# Genome-wide DNA methylation in relation to *ARID1A* deficiency in ovarian clear cell carcinoma

**DOI:** 10.1186/s12967-024-05311-7

**Published:** 2024-06-10

**Authors:** Shang Li, Gert Jan Meersma, Jolanta Kupryjanczyk, Steven de Jong, G. Bea A. Wisman

**Affiliations:** 1 Department of Medical Oncology, Cancer Research Center Groningen, University Medical Center Groningen, University of Groningen, Hanzeplein 1, 9713 GZ Groningen, The Netherlands; 2 Department of Gynecologic Oncology, Cancer Research Center Groningen, University Medical Center Groningen, University of Groningen, Hanzeplein 1, 9713 GZ Groningen, The Netherlands; 3 Department of Pathology, Maria Sklodowska-Curie National Research Institute of Oncology, Roentgena 5, 02-781 Warsaw, Poland

**Keywords:** Ovarian clear cell carcinoma, ARID1A, DNA methylation, EZH2

## Abstract

**Background:**

The poor chemo-response and high DNA methylation of ovarian clear cell carcinoma (OCCC) have attracted extensive attentions. Recently, we revealed the mutational landscape of the human kinome and additional cancer-related genes and found deleterious mutations in *ARID1A*, a component of the SWI/SNF chromatin-remodeling complex, in 46% of OCCC patients. The present study aims to comprehensively investigate whether ARID1A loss and genome-wide DNA methylation are co-regulated in OCCC and identify putative therapeutic targets epigenetically regulated by ARID1A.

**Methods:**

DNA methylation of *ARID1A*mt/ko and *ARID1A*wt OCCC tumors and cell lines were analyzed by Infinium MethylationEPIC BeadChip. The clustering of OCCC tumors in relation to clinical and mutational status of tumors were analyzed by hierarchical clustering analysis of genome-wide methylation. GEO expression profiles were used to identify differentially methylated (DM) genes and their expression level in *ARID1A*mt/ko vs *ARID1A*wt OCCCs. Combining three pre-ranked GSEAs, pathways and leading-edge genes epigenetically regulated by ARID1A were revealed. The leading-edge genes that passed the in-silico validation and showed consistent *ARID1A*-related methylation change in tumors and cell lines were regarded as candidate genes and finally verified by bisulfite sequencing and RT-qPCR.

**Results:**

Hierarchical clustering analysis of genome-wide methylation showed two clusters of OCCC tumors. Tumor stage, *ARID1A/PIK3CA* mutations and *TP53* mutations were significantly different between the two clusters. *ARID1A* mutations in OCCC did not cause global DNA methylation changes but were related to DM promoter or gene-body CpG islands of 2004 genes. Three pre-ranked GSEAs collectively revealed the significant enrichment of EZH2- and H3K27me3-related gene-sets by the *ARID1A*-related DM genes. 13 Leading-edge DM genes extracted from the enriched gene-sets passed the expression-based in-silico validation and showed consistent *ARID1A*-related methylation change in tumors and cell lines. Bisulfite sequencing and RT-qPCR analysis showed promoter hypermethylation and lower expression of *IRX1*, *TMEM101* and *TRIP6* in *ARID1A*mt compared to *ARID1A*wt OCCC cells, which was reversed by 5-aza-2′-deoxycytidine treatment.

**Conclusions:**

Our study shows that ARID1A loss is related to the differential methylation of a number of genes in OCCC. *ARID1A*-dependent DM genes have been identified as key genes of many cancer-related pathways that may provide new candidates for OCCC targeted treatment.

**Supplementary Information:**

The online version contains supplementary material available at 10.1186/s12967-024-05311-7.

## Background

Ovarian cancer is the 8th most common form of cancer in women worldwide [1]. The second most common epithelial subtype of ovarian cancer is ovarian clear cell cancer (OCCC) comprising 5–10% of all ovarian cancers in the western world and 25% in Asia, respectively [2, 3]. OCCC has much worse survival rate relatively to the other subtypes when diagnosed at an advanced stage. This poor clinical outcome is possibly due to resistance to platinum-based chemotherapy [4, 5]. Over the last decade it has become increasingly clear that besides the genetic changes, pathogenesis and chemo-resistance of ovarian cancer are also closely related to epigenetic changes, which involves, amongst others, chromatin remodeling and DNA methylation [6, 7].

AT-Rich Interaction Domain 1A (ARID1A) is an essential subunit of SWItch/Sucrose Non-Fermentable (SWI/SNF) chromatin remodeling complex. *ARID1A* mutations are found in 46–62% OCCC patients [8–11] and may relate to short progression-free survival and chemoresistance [11], but the association between *ARID1A* mutation and overall survival of OCCC remains controversial [8, 12]. Most *ARID1A* mutations are heterozygous nonsense mutations or frameshifts all resulting in ARID1A loss, which is supposed to be an early event during the transformation from precursor lesions, such as endometriosis and benign clear-cell adenofibroma, into OCCC [10, 13].

A recent multi-cancer study revealed that ARID1A loss can cause CpG island methylation phenotype of endometrial cancer, which is known to be associated with patient prognosis and diagnosis of various types of cancers [14]. In addition, methylation clustering analysis including 271 OCCC tumors identified a cluster significantly enriched for OCCCs with multiple *ARID1A* mutations [15]. All this accumulating evidence has implied an underlying association between ARID1A and DNA methylation. DNA methylation in the mammal genome occurs at CpG (deoxycytidine-phosphate-deoxyguanosine) sites that are often enriched in CpG islands (CGIs). Aberrant gaining and losing DNA methylation usually results in silencing of tumor-suppressor genes and activation of oncogenes, respectively, both contributing to tumorigenesis and metastasis [16–18]. In the recent years, the underlying diagnostic and therapeutic value of DNA methylation in cancer treatments, especially in OCCC, starts to gain attention, because of the unique methylation profile of OCCC when compared to other subtypes of ovarian cancer [19]. So far, methylation signatures of specific genes (*HNF-1B, WT1, WTI-AS, HIN-1* and *SFRP5*) have been identified as potential diagnostic or prognostic markers for OCCC patients [20–23]. In addition, it has been reported that enhancer of zeste homolog 2 (EZH2), a key component of poly-comb repressive complex (PRC2), is essential for recruitment of DNA methyltransferases (DNMTs) to EZH2-target genes, which results in promoter DNA methylation and repressed expression of the target genes [24]. EZH2 is essential for the viability of *ARID1A*mt OCCC cell lines as reflected in the in vitro and vivo responses to EZH2 inhibition, which is significantly correlated with *ARID1A* mutational status of OCCC cell lines and xenograft models [25, 26]. Of note, the expression of EZH2 is upregulated in *ARID1A*mt OCCCs compared to *ARID1A*wt OCCCs [26], which may lead to altered DNA methylation. However, whether *ARID1A* mutations and genome-wide DNA methylation are interconnected and how it is related to EZH2 activity in OCCC still needs to be unveiled.

Therefore, in this study we aim to extensively investigate whether ARID1A loss and genome-wide DNA methylation are co-regulated in OCCC, and to identify crucial genes that are not only epigenetically regulated by ARID1A but also have possible therapeutic values in OCCC. Utilizing OCCC tumor specimens and cell lines, the relation between ARID1A loss and DNA methylation has been comprehensively investigated. In addition, gene dependency data was used to identify potential gene candidates that were specifically essential for *ARID1A* mutant (mt) or *ARID1A* wildtype (wt) OCCC. After expression-based in-silico validation, 13 genes with putative clinical relevance, of which DNA methylation is closely related to the presence of mutant *ARID1A* in OCCC, were identified as potential targets. Finally, we validated the methylation status and RNA expression of some of these genes and demonstrated that these genes were epigenetically regulated.

## Materials and methods

### OCCC tumor samples and cell lines

To analyze the methylation status of OCCC, we used 24 *ARID1A*wt/11 *ARID1A*mt OCCC patient tumors, 13 OCCC cell lines (3 *ARID1A*wt/10 *ARID1A*mt), and 2 isogenic *ARID1A* knock out (ko) OCCC cell line models. Primary OCCC tumor samples were collected in Poland and the Netherlands. The OCCC patient tumors were obtained as previously described [8]. In brief, all patients gave written informed consent for data storage and tumor collection, and studies were conducted in accordance with the Declaration of Helsinki principles and the corresponding ethical review boards approved the study. Tumor samples which contained > 40% tumor cells were defined as tumor samples and when 70% of that tumoral area was OCCC, this tumor sample was confirmed as an OCCC tumor by an experienced gynecologic oncology pathologist. We obtained 13 human OCCC cell lines: TOV21G (ATCC); RMG1, RMG2, OVMANA, HAC2, and OVTOKO (JCRB Cell Bank); OVCA429 (Cell Biolabs); OVSAYO, TUOC1, OVAS, SMOV2, and KOC7C (Dr. Hiroaki Itamochi, Tottori University School of Medicine, Tottori, Japan); ES2 (Dr. Els Berns, Erasmus MC, Rotterdam, the Netherlands). Stable *ARID1Ako* clones of ES2 (ES2^*ARID1A−/−*^) and OVCA429 (OVCA429^*ARID1A−/−*^) were generated as described before [27] (Dr. Katrien Berns, The Netherlands Cancer Institute, Amsterdam). Duplicates of ES2, ES2^*ARID1A−/−*^, OVCA429 and OVCA429^*ARID1A−/−*^ were used for the genome-wide DNA methylation analysis. All cell lines were maintained in RPMI supplemented with 10% FCS, 100 mg/mL penicillin/streptomycin and 2 mM l-glutamine. All cell lines were tested by short tandem repeat profiling and were mycoplasma free. All cell lines were kept in culture for a maximum of 50 passages. Clinical data and genetic mutations (*ARID1A*, *PIK3CA*, *TP53*, *ATM* and *KRAS*) of all samples were obtained as described [8] (Supplementary Table 1–2).

### Bioinformatic analysis

An overview of the approach to select the methylated genes is summarized in Supplementary Fig. 1.

#### Genome-wide DNA methylation profiling

DNA of all samples was isolated using standard salt-chloroform extraction and isopropanol precipitation. Precipitated DNA was resuspended in Tris–EDTA buffer (10 mM Tris, 1 mM EDTA, pH 8.0). The quality control of genomic DNA and subsequently the Infinium MethylationEPIC BeadChip arrays (865859 CpG probes, Illumina, San Diego, CA, USA) were performed by GenomeScan (Leiden, the Netherlands) according to the GenomeScan protocol which is adapted from the “Illumina II Methylation Assay Manual protocol”. In brief, to assess the quality of samples, Thermo Fisher's Quant-IT analysis was used to determine the DNA concentration. Gel-electrophoresis was performed to assess the quality of the DNA sample and only those that passed quality control were analyzed in this study. Subsequently, genomic DNA (130–500 ng of each sample) was bisulfite-converted using the EZ DNA Methylation Gold Kit (Zymo Research) and used for microarray-based DNA methylation analysis. The bisulfite-converted DNA was then processed and hybridized to the MethylationEPIC BeadChip arrays according to the manufacturer's instructions. BeadChip images were scanned on the iScan system and the data quality was assessed using the R script MethyIAid [28] using default analysis settings. Detailed data processing of Infinium MethylationEPIC BeadChip arrays is described in Supplementary Methods.

#### Differential dependency between ARID1Amt and ARID1Awt OCCC

In order to identify *ARID1A*mt or *ARID1A*wt specific gene candidates in OCCC, CRISPR (DepMap 21Q3 Public + Score Chronos) and RNAi (Achilles + DRIVE + Marcotte DEMETER2) dependency of 12 OCCC cell lines (Supplementary Table 2) were obtained from DepMap website [29]. A negative dependency score from DepMap indicates that a gene is essential for cell growth and a positive dependency score indicates that inhibition of a gene will benefit cell growth [30, 31]. Average dependency scores of gene candidates in *ARID1A*mt and *ARID1A*wt OCCC were calculated.

#### Additional bioinformatic analysis

The hierarchical clustering, multiple linear regression analysis, identification of differential methylated (DM) CpGs located in gene promoters or gene-bodies, batch effect correction of GEO expression profiles (Supplementary Fig. 2), determining expression of DM CpGs targeting genes, pre-ranked gene-set enrichment analysis (pre-ranked GSEA), expression-based in-silico validation and potential clinical relevance of identified genes and visualization of candidate genes and the enriched gene-sets are described in Supplementary Methods.

### Validation of ARID1A-related DM gene candidates

A panel of human OCCC cell lines (RMG1, ES2, ES2^*ARID1A−/−*^, OVCA429, OVCA429^*ARID1A−/−*^, SMOV2, TOV21G and TUOC1) was used for in vitro validation and functional analysis. Bisulfite sequencing PCR (BSP) and quantitative reverse transcription PCR (RT-qPCR) were used to validate the methylation and expression alteration of *ARID1A*-related DM gene candidate in OCCC cell lines. The visualization of the focused region of each *ARID1A*-related DM gene candidates were done using UCSC Genome Browser on Human (GRCh37/hg19). The detailed description of these methods and the processing of the obtained data are described in Supplementary Methods.

## Results

### Whole genome methylation signatures of OCCC tumors and cell lines

**Fig. 1:**
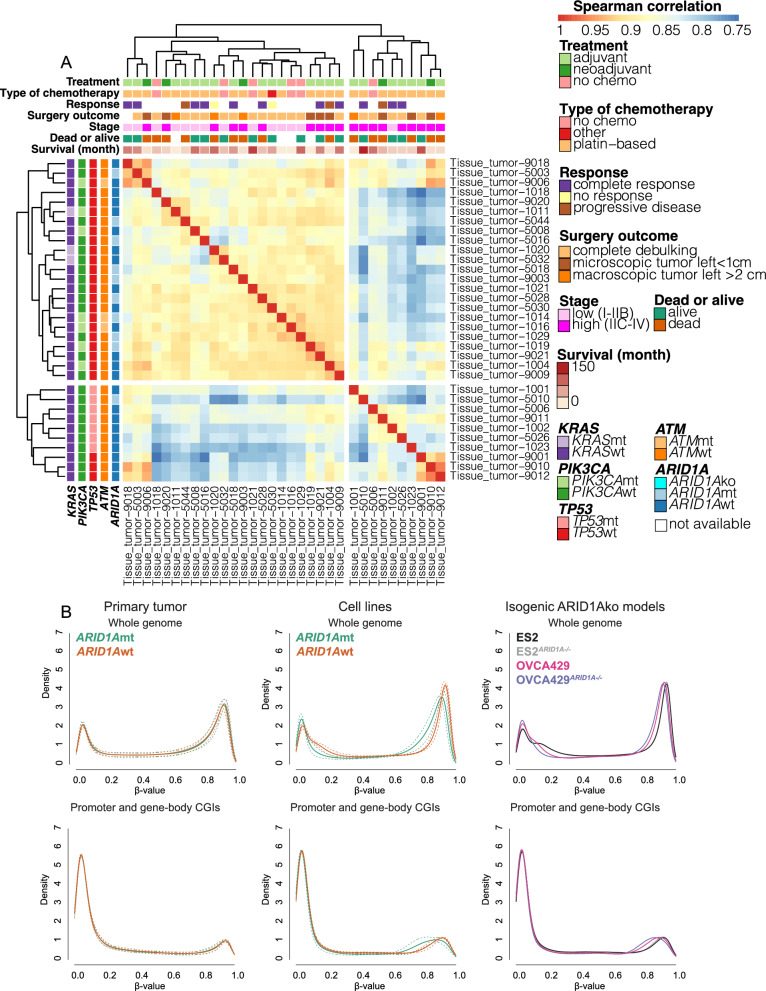
Methylation of OCCC tumors and cell lines. **A** Unsupervised two-dimensional hierarchical clustering of OCCC tumors based on β-values of 692,994 CpGs. The clinical data and genetic mutations of OCCC tumors are indicated. **B** Density distribution of methylation β-values of whole genome (up, 692,994 CpGs) and promoter and gene-body CGIs (down) are measured in *ARID1A*mt/ko vs *ARID1A*wt OCCC. Solid lines indicate the mean β-values, while dashed lines indicate the mean ± standard deviation of β-values

Genetic mutations of all samples and clinical characters of primary tumor used in this study are provided in Supplementary Table 1 and 2. Out of the ~ 850,000 methylation CpG probes in the Infinium MethylationEPIC BeadChip arrays, 692,994 CpGs remained after quality control using multi-step filtration. Based on the β-values of these 692,994 CpGs, hierarchical clustering analysis of genome-wide methylation of OCCC primary tumors (Fig. 1A) and cell lines (Supplementary Fig. 3A) was performed. *ARID1A*/*PIK3CA* mutations* (*Fisher exact test, *p* = 0.01), tumor stage (Fisher exact test, *p* = 0.02) and *TP53* mutations (Fisher exact test, *p* < 0.001) were significantly related to the methylation-based clustering of OCCC primary tumors. Tumor stage was not related to mutational status. Furthermore, based on multiple linear regression analysis, there was a trend for primary OCCC tumors with more mutations in either *ARID1A* or *PIK3CA* (*Estimate* = 0.19, *p* = 0.054) and a lower tumor stage (*Estimate* = −1.95, *p* = 0.08) in methylation cluster 1. OCCC tumors with mutant *TP53* were significantly enriched in cluster 2 (*Estimate* = −0.74, *p* = 1.67e-06). Methylation-based clustering of OCCC cell lines revealed a separation between *ARID1A*mt and *ARID1A*wt cell lines (Fisher exact test, *p* = 0.04). Hierarchical clustering of genome-wide methylation of both OCCC primary tumors and cell lines (Supplementary Fig. 3B) showed that tumor methylomes were more similar to each other than to cell lines, and vice versa. Additionally, ES2^*ARID1A−/−*^ and OVCA429^*ARID1A−/−*^ (the isogenic *ARID1A*ko cell lines) did not cluster with the *ARID1A*mt cell lines, instead they were strongest associated with their parental ES2 and OVCA429 cell lines, respectively.

The distribution of genome-wide DNA methylation (β-value) of both tumors and cell lines showed a clear bi-modal distribution, as shown in Fig. 1B. A shift in distribution was observed when comparing *ARID1A* deficient (*ARID1A*mt/ko) and *ARID1A*wt OCCC cell lines, which was most evident at the highly methylated sites (0.8 < β ≤ 1) and low methylated sites (0 < β ≤ 0.2). The shift in β-value distribution at the highly methylated sites (0.8 < β ≤ 1) was also observed when only promoter and gene-body CGIs were analyzed. No differences in β-value distribution of global and CGI methylation were observed between *ARID1A*mt and *ARID1A*wt primary tumors.

To gain more detailed insight in DNA methylation per CpG, we performed one to one comparison of 692,994 CpGs in *ARID1A*mt/ko OCCC vs *ARID1A*wt OCCC (Supplementary Fig. 4). CpGs showing methylation changes in *ARID1A*mt OCCC vs *ARID1A*wt OCCCs were equally distributed over the chromosomes. In total, methylation of ~ 10% of the CpGs in *ARID1A*mt primary tumors, ~ 40% of the CpGs in *ARID1A*mt cell lines and ~ 20% of the CpGs in *ARID1A*ko models increased or decreased more than 0.1 β-value compared to matched CpGs in *ARID1A*wt OCCC. In addition, methylation of ~ 5% of the CpGs in *ARID1A*mt cell lines and ~ 1% of the CpGs in OVCA429 *ARID1A*ko increased or decreased more than 0.4 β-value. There were almost no CpGs (less than 1%) that showed a change in methylation of more than 0.4 β-value in *ARID1A*mt primary tumors and ES2 *ARID1A*ko.

Thus, *ARID1A* deficiency was related to methylation changes in global CpGs and in promoter and gene body CpGs and was most evident in OCCC cell lines.

### Identification of differential methylated (DM) CpGs in promotor and gene body between *ARID1Amt/ko* and *ARID1Awt* OCCC

**Fig. 2:**
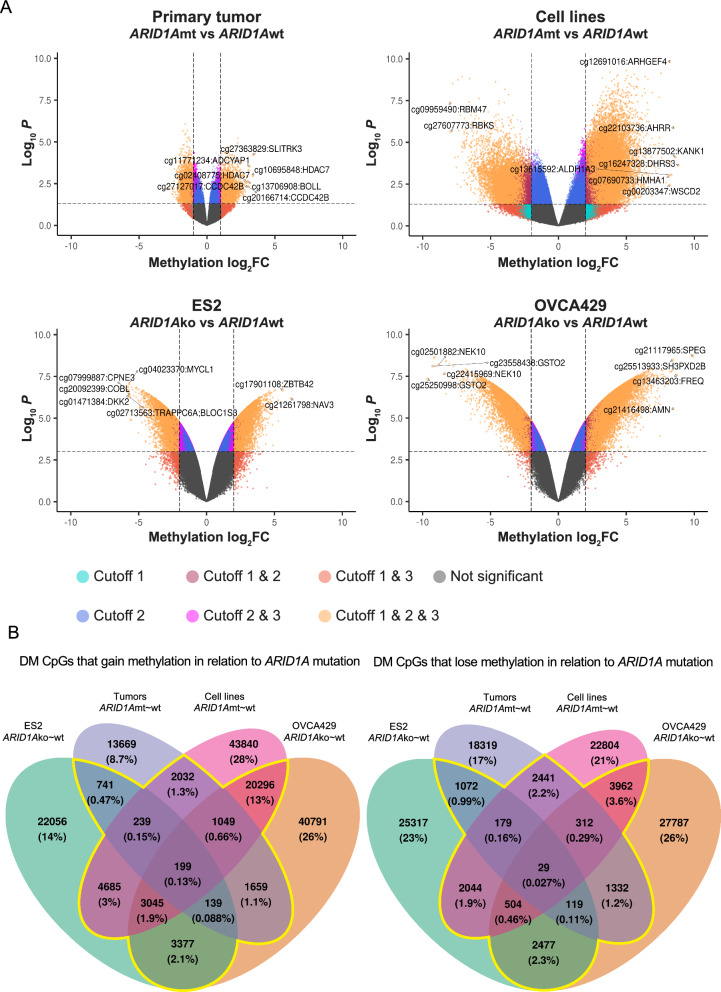
Identification of DM CpGs between *ARID1A*mt/ko vs *ARID1A*wt OCCC. **A** Volcano plot showing the identified DM CpGs in OCCC tumor, cell lines and isogenic *ARID1Ako* models. Scattered dots represent CpGs. The x-axis is the methylation log_2_fold change based on M-value, whereas the y-axis is -log10 transformed significance p-value of differential methylation obtained from” limma” method. Dots are colored based on the cut-offs they satisfy. The top altered CpGs based on M-value log_2_fold change were specified. The names of the target genes of specified CpGs are adjacent to the corresponding CpGs. **B** Venn diagram showing the number and corresponding percentage of DM CpGs in the 4 sample sets (OCCC tumors, cell lines and isogenic *ARID1Ako* models). Common DM CpGs which identified in at least 2 out of 4 sample sets are marked by the yellow line

To detect methylation changes that are associated with the *ARID1A* mutational status, DM CpGs identified in 4 sample sets (“primary tumor”, “cell lines”, “ES2 vs ES2^*ARID1A−/−*^”, “OVCA429 vs OVCA429^*ARID1A−/−*^”) were compared (Fig. 2A, Supplementary Table 3). Only DM CpGs that were commonly identified in at least two out of four sample sets were selected, comprising 39,859 unambiguous DM CpGs (Fig. 2B). After annotation of these DM CpGs to the human genome, we found that 3627 DM CpGs were located in promoter or gene-body CGIs of 2004 genes (Table 1).

**Table 1:** Identified common DM CpGs located in gene promoter or gene-body CGI

	CpGs gained methylation	CpGs lost methylation	Genes targeted by CpGs gained methylation	Genes targeted by CpGs lost methylation
Promoter CGIs	1259	898	758	638
Gene body CGIs	1141	241	632	204
Alternative promoter CGIs	84	58	63	44

**Fig. 3:**
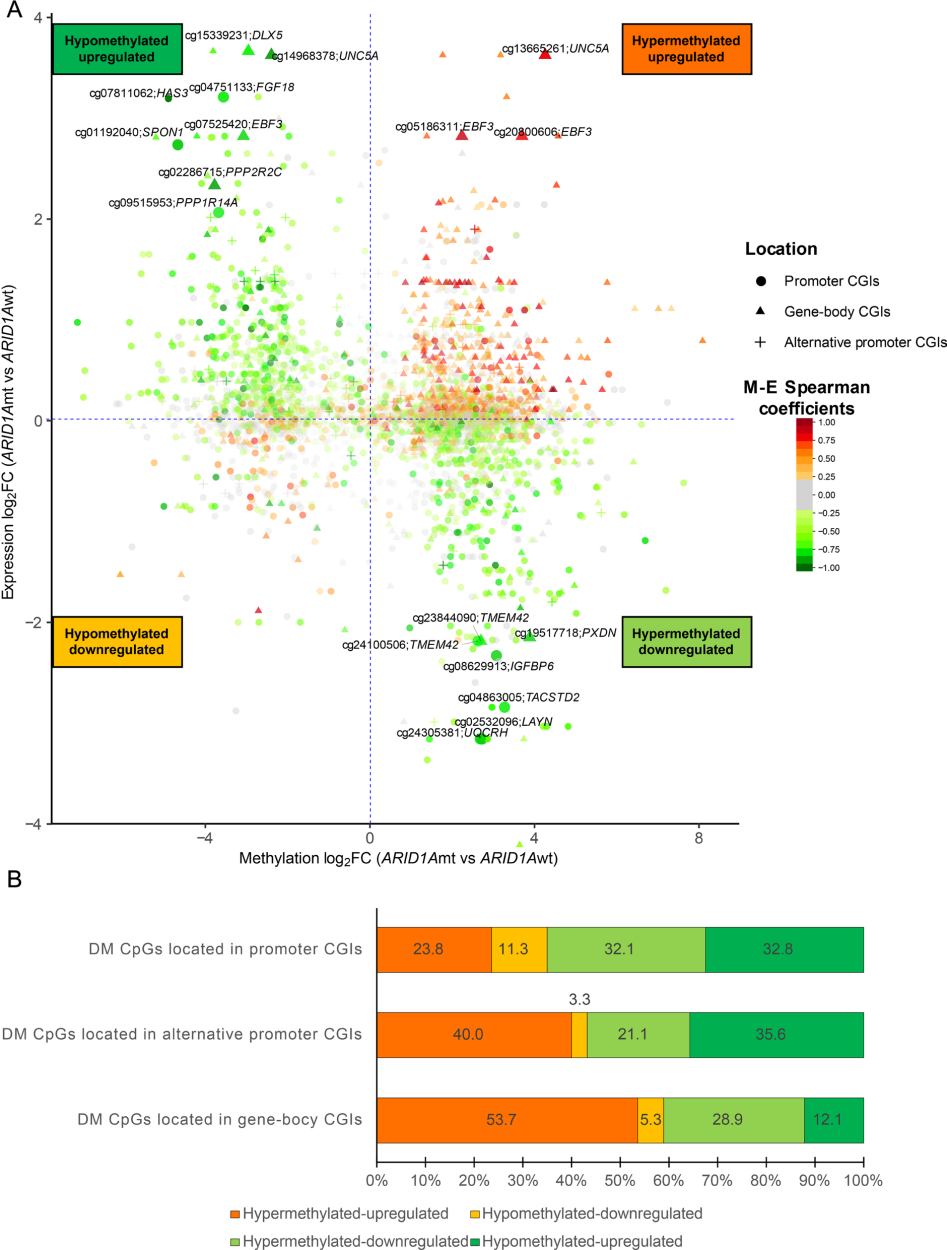
Methylation of DM CpGs and expression of the target genes based on OCCC cell lines. **A** The x-axis is the methylation log_2_FC of DM CpGs. The y-axis is expression log_2_FC of their corresponding target genes. The shape of each CpG point is based on the transcriptional regulatory element it is located in, whereas the color of each CpG dot is based on the Spearman coefficient between its M-value and expression of its target gene (M-E Spearman coefficient). The enlarged points or triangles specify the CpGs that satisfy all of the 3 conditions: (1) methylation log_2_FC of the CpG is > 2; (2) expression log_2_FC of the target genes is > 2; (3) the absolute M-E Spearman coefficients between their methylation and expression of their target genes is > 0.75. The names of CpGs and their target genes are adjacent to the corresponding enlarged points. **B** The methylation changes of DM CpGs and the expression alteration of their target genes. DM CpGs and their target genes are divided based on the pattern of their methylation and expression alteration between *ARID1A*mt cell lines vs *ARID1A*wt OCCC cell lines: hypermethylated-upregulated (orange), hypomethylated-downregulated (yellow), hypermethylated-downregulated (light green) and hypomethylated-upregulated (green) genes. The percentage of each group in promoter, alternative promoter and gene-body DM CpGs are labeled on the corresponding bar

Next, the M-E Spearman coefficients (indicated in green and red) were calculated for the 2004 genes using the β-value for each of the 3627 DM CpGs located in gene promoter or gene-body CGIs, and the expression level of their respective target genes using data from 11 *ARID1A*mt*/*wt cell lines. As shown in Fig. 3A, an inverse relation between fold changes in DM CpGs and fold changes in expression of their target genes comparing *ARID1A*mt and *ARID1A*wt cell lines, was often observed (hypermethylated-downregulated and hypomethylated-upregulated). A positive relation between the fold changes of DM CpGs and fold changes in expression of their target genes comparing *ARID1A*mt and *ARID1A*wt cell lines was also observed (hypermethylated-upregulated and hypomethylated-downregulated). Genes with the largest promoter methylation and expression changes between *ARID1A*mt vs *ARID1A*wt cell lines were depicted, such as *PPP1R14A* and *UQCRH*.

The methylation and expression alterations of the genes with absolute M-E Spearman coefficients ≥ 0.25 in association with the genomic location of the DM CpGs were summarized in Fig. 3B. In total, around 65% of the gene promoter DM genes were found to have hypermethylated-downregulated (light green) and hypomethylated-upregulated (green) patterns, indicating that majority of changes in promoter methylation in *ARID1A*mt cells were inversely related to the changes in expression of target genes. Around 60% of gene-body DM CpGs were found to be hypermethylated-upregulated (orange) and hypomethylated-downregulated (yellow), indicating that changes in methylation of most gene bodies were positively correlated to changes in target gene expression in *ARID1A*mt cells.

Taken together, ARID1A-dependent changes in promoter methylation correlated negatively with gene expression, while ARID1A-dependent changes in gene-body methylation were mostly positively correlated with gene expression, which is in line with the classical theory how DNA methylation regulates gene expression [32].

### EZH2 related gene-set was enriched in ARID1A-related DM genes

**Fig. 4:**
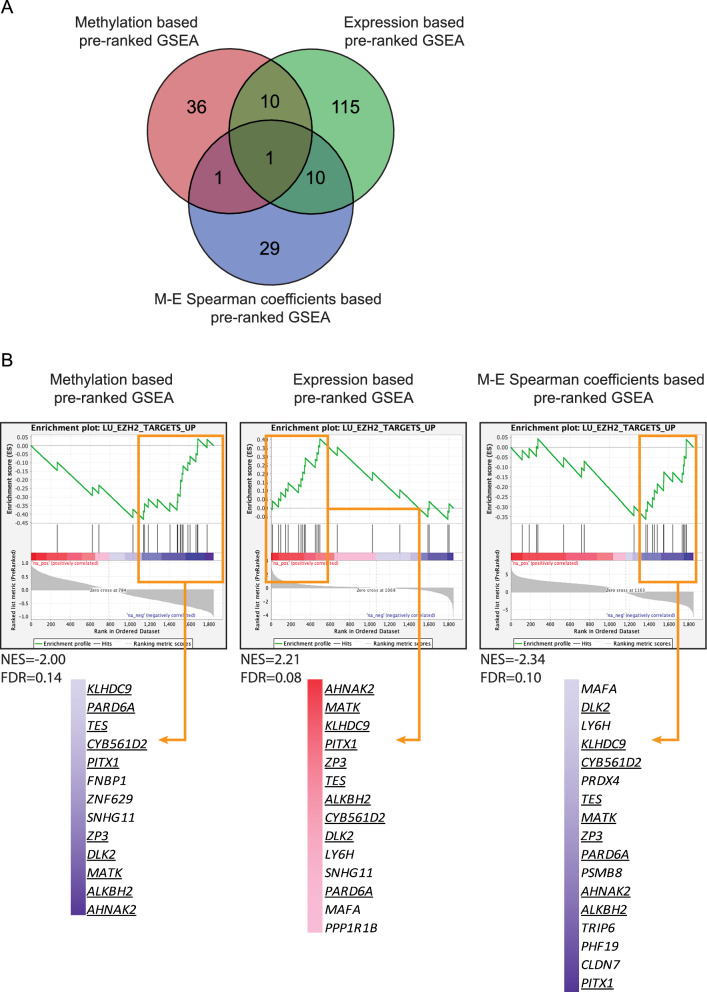
Significant gene-sets identified by the 3 different pre-ranked GSEA analyses. **A** Venn plot of significant gene-sets derived from methylation log_2_FC based pre-ranked GSEA, expression log_2_FC based pre-ranked GSEA and M-E Spearman coefficient based pre-ranked GSEA. **B** Enrichment curve of “LU EZH2 TARGETS UP” derived from methylation log_2_FC based pre-ranked GSEA, expression log_2_FC based pre-ranked GSEA and M-E Spearman coefficient based pre-ranked GSEA. The corresponding normalized enrichment scores (NES) and FDRs of “LU EZH2 TARGETS UP” are annotated. Orange boxes and arrows marked the leading-edge genes of “LU EZH2 TARGETS UP” derived from methylation log_2_FC based pre-ranked GSEA, expression log_2_FC based pre-ranked GSEA and M-E Spearman coefficient based pre-ranked GSEA. The corresponding locations of leading-edge genes inside the enrichment curve were indicated by the color bar next to them. Leading-edge genes of “LU EZH2 TARGETS UP” commonly identified by all three pre-ranked GSEA were underlined

We further investigated which genes and related pathways were mostly affected by *ARID1A*mt related methylation. The effects of *ARID1A*mt-related methylation on 2004 genes (DM in promoter or gene-body) were evaluated in data obtained from OCCC cell lines using 3 parameters: alterations on the methylation level, alterations on the expression level, and the correlation between DNA methylation and expression. According to the pre-ranked GSEA based on each of the 3 parameters, in total 202 significantly enriched gene-sets were identified (FDR ≤ 0.25, |NES|≥ 2, Supplementary Table 5). Noticeably, “LU EZH2 TARGETS UP” was the only commonly identified gene-set by all 3 pre-ranked GSEA methods (Fig. 4A, Supplementary Table 6). Its negative methylation NES (−2.00), positive expression NES (2.21) and negative M-E spearman coefficient NES (−2.34) followed the classic pattern of DNA methylation regulated gene expression. These results suggest that the methylation and expression of leading-edge genes of this EZH2 related gene-set (indicated by orange arrows) are strongly depending on the *ARID1A* status in OCCC cells (Fig. 4B). These findings are in line with previous studies, demonstrating the importance of EZH2 in *ARID1A*mt OCCC cells [26], and gives confidence to the approach taken here.

### Dependency analysis of ARID1A-related DM genes

**Fig. 5:**
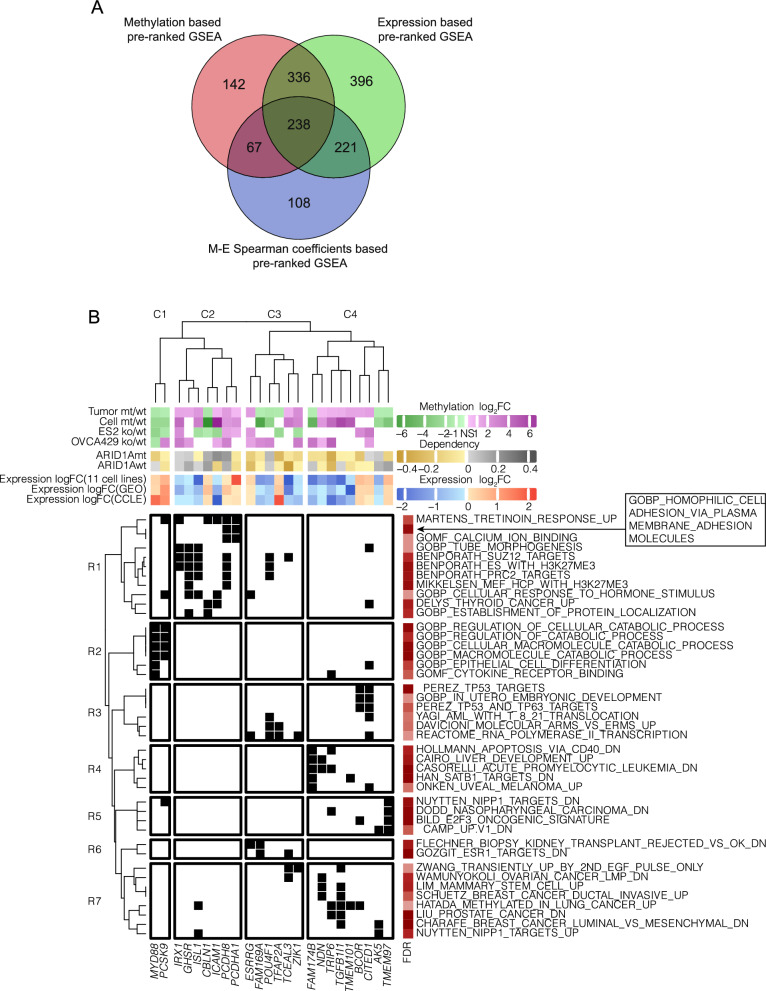
*ARID1A* dependency and expression alteration of leading-edge genes. **A** Venn plot of leading-edge genes derived from methylation log_2_FC based pre-ranked GSEA, expression log_2_FC based pre-ranked GSEA and M-E Spearman coefficient based pre-ranked GSEA. **B** Dependency and expression alteration of leading-edge genes with consistent differential methylation in *ARID1A*mt OCCC tumor and cell lines. Leading-edge genes with differential methylation in both OCCC tumor and cell lines are shown in the columns of the heatmap, while the corresponding enriched gene-sets that shared more than 2 DM leading-edge genes are shown in the rows of the heatmap. The distance between every two components in columns (genes) or rows (gene-sets) was calculated based on Spearman coefficients. “ward.D2” method was used to cluster columns (genes) and rows (gene-sets) of the heatmap according to the corresponding Spearman coefficients. A black-colored cell depicts whether a certain gene is presented in a given gene-set. Methylation log_2_FCs of the DM CpG are indicated in the first panel of row annotations; Average dependency of corresponding leading-edge genes in *ARID1A*mt cell lines and *ARID1A*wt cell lines are indicated in the second panel of row annotations; Expression log_2_FC (9 cell lines) of leading-edge genes from GEO and expression log_2_FC from CCLE (12 cell lines) are indicated in the third panel of row annotations. The most significant FDR value of each enriched gene-set from three pre-ranked GSEA is indicated in the column annotation

To identify DM genes that play a central role in various pathways, so-called hubs, we selected the leading-edge genes of significant gene-sets for each pre-ranked GSEA. We found 238 leading-edge genes that were common between the three used GSEA methods (Fig. 5A, Supplementary Table 6 and Supplementary Table 7). The average dependency score for 234 out of 238 genes in *ARID1A*mt and *ARID1A*wt OCCC cell lines were calculated using the DepMap dataset (Supplementary Table 8, 4 genes were not present in the OCCC samples from DepMap).

In total, 24 *ARID1A*-related DM genes showed consistent *ARID1A*-related methylation alterations in primary tumor and cell lines and were present in 42 gene-sets. Considering that related genes may function in shared pathways and vice versa, the 24 genes and 42 gene-sets were further clustered into modules (Fig. 5B). R1–R7 and C1–C4 were used as horizonal and vertical coordinates of a certain module in the clustering heatmap. Noticeable, the most altered genes depicted in Fig. 3 were excluded from the analysis, since they were not commonly identified by three separate pre-ranked GSEA analyses. The leading-edge genes of “LU EZH2 TARGETS UP” (Fig. 4B), except *TRIP6,* did not pass the expression-based in-silico validation, because of inconsistent methylation changes between primary tumor and cell lines (Supplementary Table 9).

Possible functions of genes presented in modules were revealed. Tumor suppressor gene *IRX1* was present in 3 EZH2 and H3K27me3 related gene-sets, indicating the possible association between *IRX1* and EZH2 in OCCC. Additionally, members of module R7-C4 (*TRIP6*, *TMEM101* and *BCOR*) shown in “HATADA METHYLATED IN LUNG CANCER UP” were detected as promoter hypermethylated in *ARID1A* deficient OCCC tumor and cell lines. Moreover, compared to *ARID1A*wt cell lines, *TRIP6* and *TMEM101* showed relatively low expression in *ARID1A*mt cell lines. For *BCOR*, methylation and expression were generally inversely correlated, except in TOV21G and KOC7C cells (Additional File 1). Interestingly, *BCOR* was also present in 2 *TP53* related gene-sets ("PEREZ TP53 TARGETS" and "PEREZ TP53 AND TP63 TARGETS") of module R3-C4, suggesting a role of *BCOR* in p53 signaling as well.

### Validation of ARID1A-related DM genes

At last, to have an in-depth view on the methylation status of the *ARID1A-*related DM genes, the methylation of the promoter or gene-body CGIs of these 24 genes in primary OCCC and cell line sample sets were visualized using UCSC genome browser online tool. Based on the UCSC visualization, 13 from these 24 genes showed a relatively high CpG level consistency between primary OCCC and cell line sample (Table 2, Supplementary Table 10). Therefore, these 13 DM genes were selected for further validation via BSP and/or RT-qPCR. For 7 genes (*AK5, CBLN1, ESRRG, MYD88, NDN, PCDH8* and *TCEAL3*), we encountered difficulties to design and optimize BSP assays due to the extreme large size and high CpG density of the region of interest. For the other 6 genes (*BCOR, IRX1*, *PCDHA1, TMEM101, TRIP6* and *ZIK1*), BSP was performed.

**Table 2:** Methylation, expression change, M-E Spearman coefficients and gene dependency of 13 *ARID1A*-related DM genes

Gene	Methylation log_2_FC	Expression log_2_FC	M-E Spearman coefficient	Location of methylation change	Dependency score	
	*ARID1A*mt vs wt	*ARID1A*mt vs wt			*ARID1A*mt	*ARID1A*wt
*AK5*	−2.49	−0.92	−0.26	Promoter CGI	0.05	0.10
*BCOR*	1.49	0.59	−0.33	Promoter CGI	−0.21	0.00
*CBLN1*	−6.83	0.22	−0.62	Promoter CGI	0.12	0.17
*ESRRG*	0.54	0.28	−0.30	Promoter CGI	−0.17	−0.24
*IRX1*	3.86	-0.28	−0.64	Gene-body CGI	0.02	0.03
*MYD88*	−1.70	0.32	−0.65	Promoter CGI	−0.17	0.01
*NDN*	1.91	−1.12	−0.39	Promoter CGI	−0.09	−0.03
*PCDH8*	2.08	0.05	0.64	Gene-body CGI	0.18	0.12
*PCDHA1*	2.76	1.88	0.74	Gene-body CGI	−0.05	−0.09
*TCEAL3*	3.68	−2.19	−0.55	Promoter CGI	−0.06	−0.38
*TMEM101*	5.50	−0.22	−0.57	Promoter and gene-body CGI	−0.01	−0.08
*TRIP6*	4.02	−1.53	−0.68	Promoter CGI	−0.32	−0.27
*ZIK1*	2.69	−0.15	−0.60	Promoter CGI	−0.12	−0.01

**Fig. 6:**
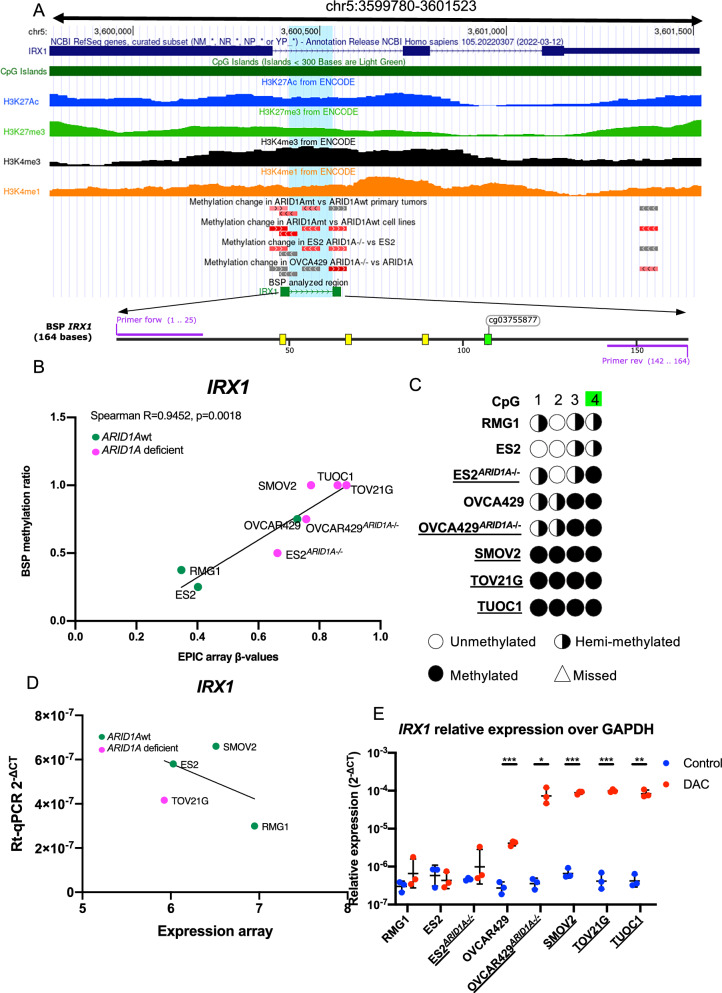
DNA methylation and gene expression of *IRX1* in *ARID1A* deficient OCCCs vs *ARID1A*wt OCCCs. **A** DNA methylation of *IRX1* promoter in OCCC cell lines. UCSC genome browser (GRCh37/hg19) representation of the genomic organization of *IRX1*. The thick solid blocks indicate the coding regions, the thinner blocks indicate the 5' and 3'UTRs, blue lines indicate introns and arrows indicate the direction of gene transcription. The CGIs are represented as horizontal green bars. H3K27me3 (green), H3K27Ac (blue), H3K4me3 (black), H3K4me1 (orange) data from ENCODE project depict histone modification status as peaks. CpGs gaining methylation (red), losing methylation (blue), insignificant (gray) in *ARID1A* deficient vs *ARID1A*wt OCCC are represented as horizontal solid bars. BSP PCR product is indicated by solid boxes (primers) and green line (analyzed sequence). The BSP-analyzed region shaded in light blue is presented below with CpG located in the BSP-analyzed region depicted as yellow bars. The labeled and green CpGs are mutually analyzed by Infinium MethylationEPIC BeadChip arrays and BSP. **B** *IRX1* BSP methylation ratio vs average β-value from Infinium MethylationEPIC BeadChip array in *ARID1A*mt (pink) and *ARID1A*wt (green) OCCC cells. The black solid line represents the regression line. **C** BSP result of *IRX1* in OCCC cells. CpG sites located in the BSP-analyzed region are numbered and showed. CpG mutually analyzed by Infinium MethylationEPIC BeadChip arrays and BSP are specified with green color. Empty circles represent unmethylated CpGs, black circles represent methylated CpGs, half black circles represent hemi-methylated CpGs and empty triangles represent missed CpGs. *ARID1A* deficient cells are underlined. **D** *IRX1* relative gene expression based on RT-qPCR vs publicly available expression profiles of *ARID1A*mt (pink) and *ARID1A*wt (green) OCCC cells. The black solid line represents the regression line. **E** RT-qPCR result of *IRX1* in OCCC cells with (red) or without (blue) DAC treatment. *ARID1A* deficient cells are underlined. Statistical significance of Student T-test is notified as *p < 0.05; **p < 0.01; ***p < 0. 001

The location of the BSP products for all 6 gene promoters were visualized using the UCSC genome browser together with the tested CpG probes from Infinium MethylationEPIC BeadChip arrays, presence of CGIs and signals of the histone marks H3K27me3, H3K27Ac, H3K4me3 and H3K4me1 (Fig. 6A, Supplementary Fig. 5A, 6A, 7A, 8A and 9A). The presence of the CGIs and the histone marks suggests that these genes are indeed epigenetically regulated. The significant Spearman correlation between the BSP semi-quantification and the Infinium MethylationEPIC BeadChip array demonstrated successful validation of most genes, whereas *PCDHA1* showed a trend (Fig. 6B, Supplementary Fig. 5B, 6B, 7B, 8B and 9B). For *IRX1, TRIP6, TMEM101,* and *ZIK1* differential promoter methylation was also shown in *ARID1A*mt/ko vs wt by BSP analysis (Fig. 6C, Supplementary Fig. 5C, 6C, 8C).

In order to test whether genes were indeed epigenetically silenced by methylation in *ARID1A*mt vs wt OCCC, we analyzed mRNA expression of *IRX1*, *TRIP6, TMEM101* and *BCOR* by RT-qPCR. Expression of *TRIP6* based on publicly available expression arrays could be validated by RT-qPCR (Supplementary Fig. 5D). The expression of *IRX1*, *TMEM101* and *BCOR* as determined with RT-qPCR was not significantly correlated to their expression based on publicly available expression arrays (Fig. 6D, Supplementary Fig. 6D and 7D), possibly due to the limited number of cell lines included in the expression arrays. Although *IRX1* expression was low in all cell lines, induction of expression by 5-aza-2′-deoxycytidine (DAC treatment) was especially observed in the *ARID1A*mt and *ARID1A*ko cell lines, consistent with the higher percentage of hemi or full methylated CpGs of the *IRX1* promoter in these cell lines compared to *ARID1A*wt cell lines (Fig. 6E). Our results thus indicate *ARID1A* status dependent epigenetic regulation of *IRX1*. High *TRIP6* and *TMEM101* promoter methylation was demonstrated in all three *ARID1A*mt cell lines and one of the *ARID1A*wt cell lines (RMG1). Low *TRIP6* expression and induction of expression after DAC treatment was, however, only found in *ARID1A*mt TUOC1 cells (Supplementary Fig. 5E). Interestingly, low *TMEM101* expression and induction of the expression after DAC treatment was observed in another cell line (TOV21G) (Supplementary Fig. 6E). The clearly detectable *BCOR* mRNA levels were not elevated in any cell line by DAC treatment (Supplementary Fig. 7E).

Taken together, the results from the MethylationEPIC BeadChip arrays were successfully validated. Our observations indicate that frequent hypermethylation and epigenetic regulation of *IRX1* expression occur especially in *ARID1A*mt and *ARID1A*ko OCCC cell lines.

## Discussion

Here, we studied the effect of *ARID1A* mutational status on genome-wide methylation in OCCC. The overall genome-wide methylation was different between *ARID1A*mt and *ARID1A*wt tumors when *ARID1A*mt status was combined with other frequently occurring mutations in OCCC, while in cell lines *ARID1A* mutational status was sufficient to show a difference in global methylation. More in-depth analysis revealed that for 2004 genes the *ARID1A* status was associated with differential promoter or gene-body DNA methylation. These *ARID1A-*related DM genes were mostly enriched in pathways related to PRC2/EZH2 activity. Leading-edge DM genes were extracted from the enriched gene-sets, and several *ARID1A-*dependent DM genes were validated. The potential clinical relevance of these genes for OCCC treatment warrants further investigation.

In the present study, we found that whole genome methylation-based clustering separated tumors with *ARID1A*mt*/PIK3CA*mt and *TP53*wt from tumors with *ARID1A*wt, *PIK3CA*wt and *TP53*mt, which is in line with previous data [15]. Furthermore, in a previous study we showed that *ARID1A* mutations were mutually exclusive with *TP53* mutations [8], which is in agreement with the current observation that DNA methylation is also distinctive between tumors with an *ARID1A* mutation and a *TP53* mutation. Nevertheless, the relation between clustering *TP53* mutations was not seen in cell lines, possible due to the small number of *TP53*mt cell lines included in the study. In addition, our data indicated that *ARID1A* deficiency in OCCC predominantly caused differential promoter and gene-body methylation of a specific set of genes rather than global DNA methylation alterations. Overall, more and larger *ARID1A*-related methylation changes were detected in cell lines than in primary tumor samples. These differences have also been observed in other contexts and can be explained by epigenetic heterogeneity within primary tumors, for instance, the presence of normal cells in the tumor microenvironment, and on the other hand the homogeneous epigenetics and RNA expression found in 2D cultures of established OCCC cell lines [33, 34]. Consequently, combining these data is essential to identify clinically relevant epigenetically regulated genes.

Our results with cell lines point at a possible connection between *ARID1A* mutation and DNA methylation, which may be EZH2 activity driven. Based on pre-ranked GSEA, we found that genes epigenetically regulated by loss of *ARID1A*wt in OCCC were enriched in many PRC2/EZH2 and histone methylation related gene-sets. In particular, “LU EZH2 TARGETS UP” was the only gene-set commonly identified by the 3 separate pre-ranked GSEA analysis, strongly suggesting that the mutational status of *ARID1A* is involved in EZH2 activity as well as DNA methylation. Previously, it was demonstrated that recruitment of DNMTs to DNA by EZH2 is responsible for promoter methylation and repression of target gene expression [24]. Others have shown in cancer cell lines, including OCCC, with mutations in either *ARID1A* or other components of the SWI/SNF complex, that inhibition of the EZH2 activity is an effective approach [25, 26]. Consequently, multiple clinical trials are ongoing investigating the effect of EZH2 inhibition, such as a phase 2 trial using tazemetostat in *ARID1A*mt tumors (NCT05023655).

We identified many genes whose methylation might be induced by EZH2 activity. *IRX1* was the common leading-edge gene of multiple EZH2 related gene-sets. Downregulation of *IRX1* in lung adenocarcinoma was shown to be caused by EZH2 activity and DNMT3B-induced promoter hypermethylation. Reversing *IRX1* inactivation by a DNMT inhibitor (DAC) induced expression of a proapoptotic regulator BAX [35]. Here, we found that *IRX1* was heavily methylated, especially in *ARID1A*mt cells, whereas gene expression was very low in all OCCC cell lines. Treatment of OCCC cells with DAC turned out to be a selective approach to reactivate *IRX1* expression in *ARID1A*mt and *ARID1A*ko cells. Whether the induction of *IRX1* expression has any functional consequence in OCCC needs to be further investigated. For *TMEM101* and *TRIP6*, DAC only had an effect in those *ARID1A*mt OCCC cell lines that showed a combination of high promoter methylation and very low gene expression. These results suggest that *ARID1A* status is not the sole factor associated with expression of these genes.

Previous studies have reported that EZH2 participates in the recruitment of DNMTs to the promoter of EZH2-target genes, while ARID1A loss in OCCCs can induce EZH2 expression by modulating PI3K pathway [25, 26]. Based on this knowledge, it is possible that the increased expression of EZH2 caused by ARID1A loss can induce alterations in DNA methylation in OCCC. Our findings provided supporting evidence to this assumption, but further studies are needed to reveal the specific mechanism how *ARID1A* mutation induce DNA methylation alterations and whether or not through an EZH2 dependent way.

*ARID1A*mt OCCCs are not only sensitive to the inhibition of EZH2, but also to HDAC6, BRD2, PARP and ATR inhibition. Based on this knowledge, synthetic lethal therapies for *ARID1A*mt OCCC have been designed, showing remarkable effectiveness in preclinical studies of OCCC [10, 26, 36]. In our study, some novel *ARID1A* specific gene targets have been identified and may offer synthetic lethal potentials in *ARID1A* mutant OCCC. *BCOR,* encoding an important component of noncanonical PRC1.1, may be such a gene. Expression of *BCOR* is higher in *ARID1A*mt cell lines. Moreover, in none of the OCCC cell lines DAC treatment did result in an upregulation of *BCOR* expression, indicating non-epigenetic regulation of its expression. BCOR can promote PRC2 recruitment to CpG islands [37], supporting the possible associations between *BCOR* expression and EZH2 activity. *BCOR* was identified as leading-edge gene of two p53 related gene-sets in our analysis, in line with previous findings [38]. Another interesting gene we identified was *MYD88*. High expression of *MYD88* was found in *ARID1A*mt cells and the *ARID1A*mt specific negative dependency score of *MYD88* suggests its therapeutic value as a synthetic lethal target of *ARID1A*mt OCCC. MYD88 is an essential activator of NF-κB pathway and has been demonstrated as an independent prognosis factor that correlated to poor survival of epithelial ovarian cancer patients [39]. Surprisingly, *TMEM101* and *MYD88*, both NF-κB activators [40], showed high expression and low promoter methylation in *ARID1A*wt OCCC cells. Thereby, NF-κB signaling appears to be crucial in both *ARID1A*mt and *ARID1A*wt OCCC, although the mechanism of NF-κB activation in OCCC might differ upon *ARID1A* mutational status. Moreover, it was reported that the treatment with NF-κB inhibitor (BAY 11-7082) suppress the growth of OCCC cells [41]. Based on these data, targeting NF-kB signaling alone or combined with *ARID1A* specific strategies might be a plausible treatment for OCCC.

A major strength of our study is that a large number of OCCC tumor samples and a broad panel of OCCC cell lines have been characterized on methylation level. Using publicly available expression data of OCCC cell lines, the expression of *ARID1A*-dependent DM genes in OCCC were investigated. In addition, the *ARID1A*-related epigenetic regulation of potential gene candidates for OCCC treatment were further validated in vitro. Nevertheless, methylation of only three *ARID1A*wt OCCC cell lines were analyzed, and therefore to compare the methylation of *ARID1A*mt vs *ARID1A*wt cell lines more comprehensively, more *ARID1A*wt cell lines could have been included. Besides, since there are no publicly available expression profiles of OCCC tumors with known *ARID1A* mutational status, expression analysis of OCCC primary tumors is lacking. Moreover, the sample size of patient samples is too small to perform survival analyses.

## Conclusions

Our study interrogates the potential relationship between *ARID1A* deficiency and DNA methylation in OCCC and shows that ARID1A loss is related to the differential methylation of a number of genes rather than global DNA methylation alterations in OCCC. *ARID1A*-dependent DM genes have been identified as key genes of many cancer-related pathways that may provide new candidates for OCCC targeted treatment. Future pre-clinical studies are required to determine the therapeutic value of these epigenetically *ARID1A-*regulated genes and the underlying regulatory mechanisms.

### Supplementary Information

Supplementary material 1. The expression of gene candidates and the methylation of DM CpGs located in their promoters or gene-bodies. Orange represents *ARID1A*mt OCCC cell lines and blue represents OCCC *ARID1A*wt cell lines.

Supplementary material 2.

Supplementary material 3: Figure 1: Approach to identify *ARID1A* related DM genes with potential clinical value in OCCC for treatment. Figure 2: Batch effect correction of 2 OCCC expression profiles from GEO database. Figure 3: Hierarchical clustering analysis of OCCC tumors and cell lines on methylation level. Figure 4: One to one comparison of all individual CpGs between *ARID1A* deficient OCCC vs *ARID1A*wt OCCC across all the chromosomes. Figure 5: DNA methylation and gene expression of *TRIP6* in *ARID1A* deficient OCCCs vs *ARID1A*wt OCCCs. Figure 6: DNA methylation and gene expression of *TMEM101* in *ARID1A* deficient OCCCs vs *ARID1A*wt OCCCs. Figure 7: DNA methylation and gene expression of *BCOR* in *ARID1A* deficient OCCCs vs *ARID1A*wt OCCCs. Figure 8: DNA methylation and gene expression of *ZIK1* in *ARID1A* deficient OCCCs vs *ARID1A*wt OCCCs. Figure 9: DNA methylation and gene expression of *PCDHA1* in *ARID1A* deficient OCCCs vs *ARID1A*wt OCCCs.

Supplementary material 4.

## Data Availability

The methylation data derived from Infinium MethylationEPIC BeadChip are available from the corresponding author upon reasonable request. The OCCC expression profiles used in this study are publicly available in NCBI's Gene Expression Omnibus and are accessible through GEO Series accession number GSE16570 and GSE29175.

## Abbreviations

DAC: 5-Aza-2′-deoxycytidine
ARID1A: AT-Rich Interaction Domain 1A
BSP: Bisulfite sequencing PCR
CGIs: CpG islands
CpG: Deoxycytidine-phosphate-deoxyguanosine
DM: Differentially methylated
DNMTs: DNA methyltransferases
EZH2: Enhancer of zeste homolog 2
FDR: False discovery rate
Mt: Mutant
NES: Normalized enrichment score
ko: Knock out
OCCC: Ovarian clear cell carcinoma
PRC2: Poly-comb repressive complex
pre-ranked GSEA: Pre-ranked gene-set enrichment analysis
RT-qPCR: Quantitative reverse transcription PCR
M-E Spearman coefficients: Spearman coefficients between M-value of DM CpGs and expression of their targeted genes
SWI/SNF: SWItch/Sucrose Non-Fermentable
Ct: Threshold cycles
Wt: Wildtype
