# Supplementary material for: Genome-wide DNA methylation in relation to ARID1A deficiency in ovarian clear cell carcinoma
Source: J Transl Med. 2024 Jun 10;22:556. doi: 10.1186/s12967-024-05311-7 (PMC11163774; doi:10.1186/s12967-024-05311-7)
Supplement: Supplementary file 1 — Supplementary material 1. The expression of gene candidates and the methylation of DM CpGs located in their promoters or gene-bodies. Orange represents ARID1Amt OCCC cell lines and blue represents OCCC ARID1Awt cell lines. [file 12967_2024_5311_MOESM1_ESM.pdf]

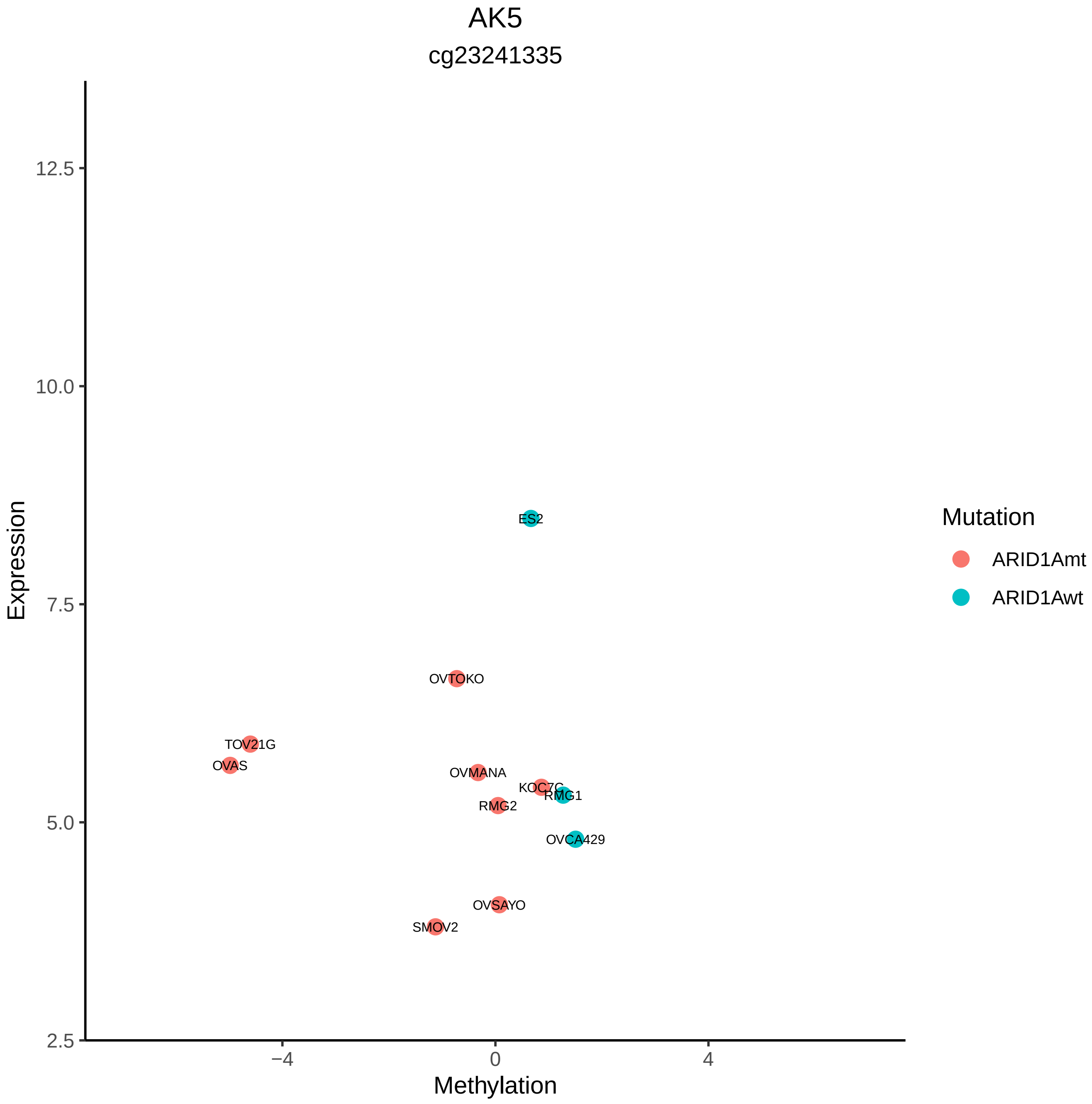
AK5
cg23241335
Expression
12.5
10.0
7.5
5.0
2.5
−4
0
4
Methylation
ES2
OVTOKO
TOV21G
OVAS
OVMANA
KOC7C
RMG1
RMG2
OVCA429
OVSAYO
SMOV2
Mutation
ARID1Amt
ARID1Awt

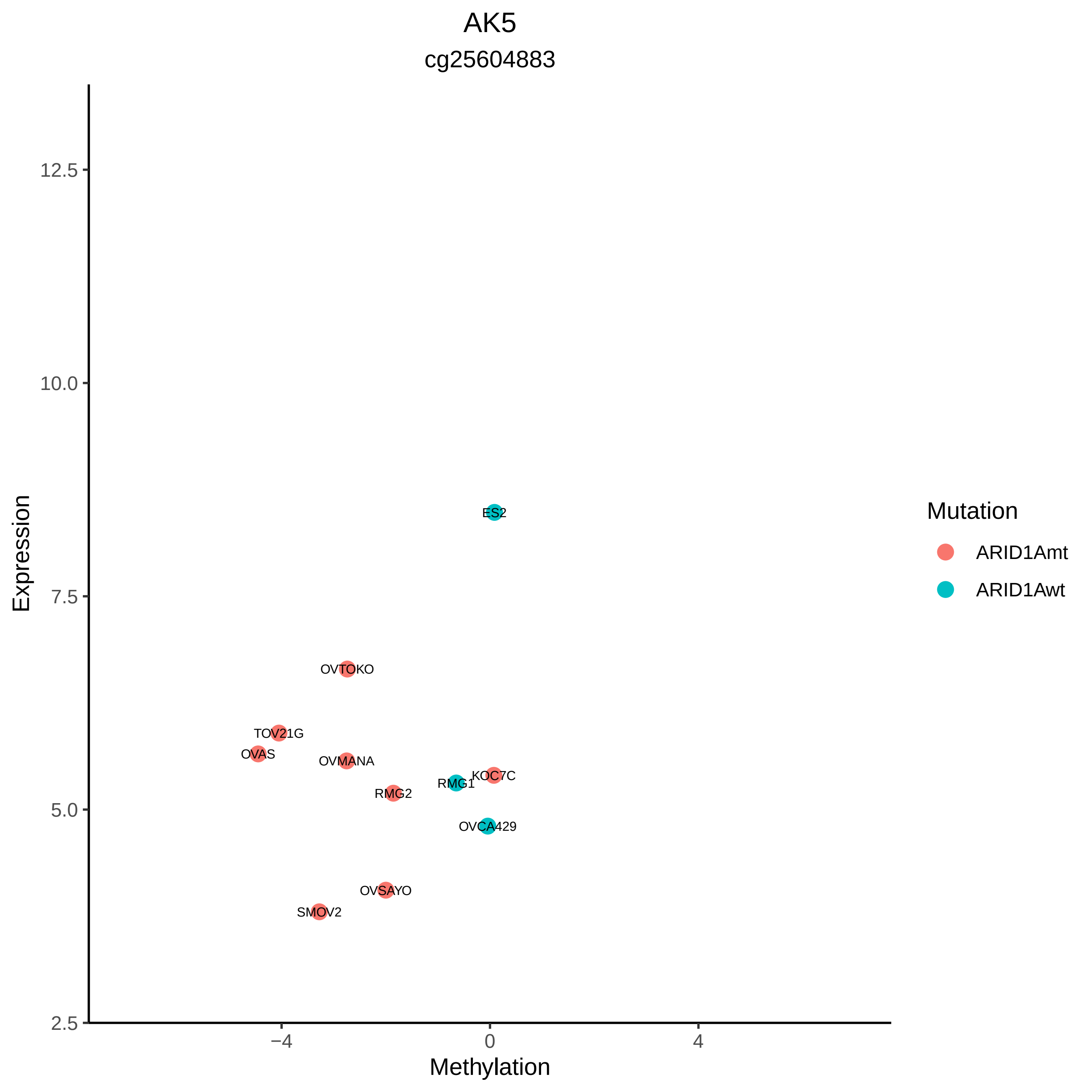

AK5
cg25604883
Expression
Methylation
12.5
10.0
7.5
5.0
2.5
−4
0
4
ES2
OVTOKO
TOV21G
OVAS
OVMANA
RMG2
RMG1
KOC7C
OVCA429
OVSAYO
SMOV2
Mutation
ARID1Amt
ARID1Awt

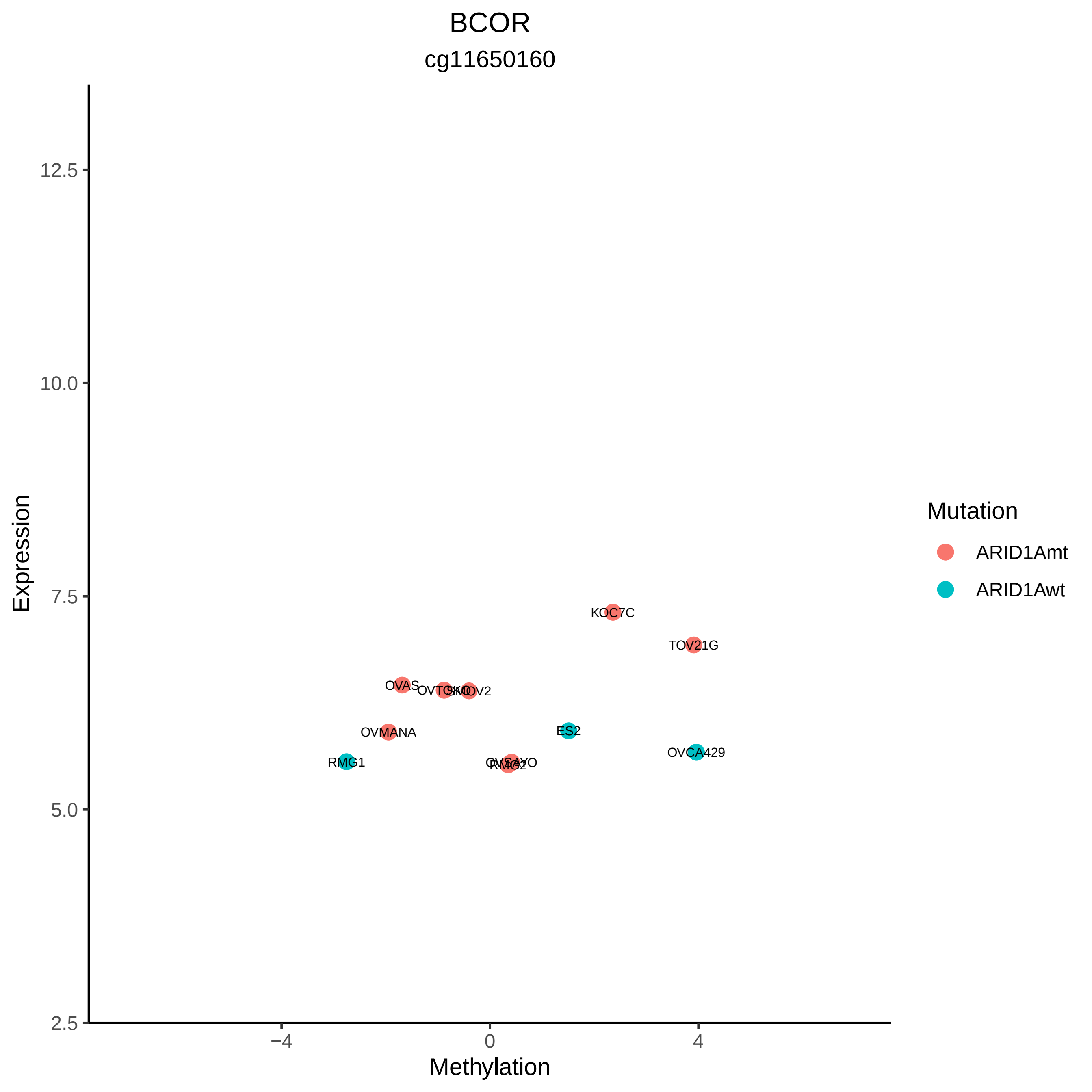

BCOR
cg11650160
Expression
Methylation
12.5
10.0
7.5
5.0
2.5
−4
0
4
KOC7C
TOV21G
OVAS
SMOV2
OVMANA
ES2
OVCA429
RMG1
RMG2
Mutation
ARID1Amt
ARID1Awt

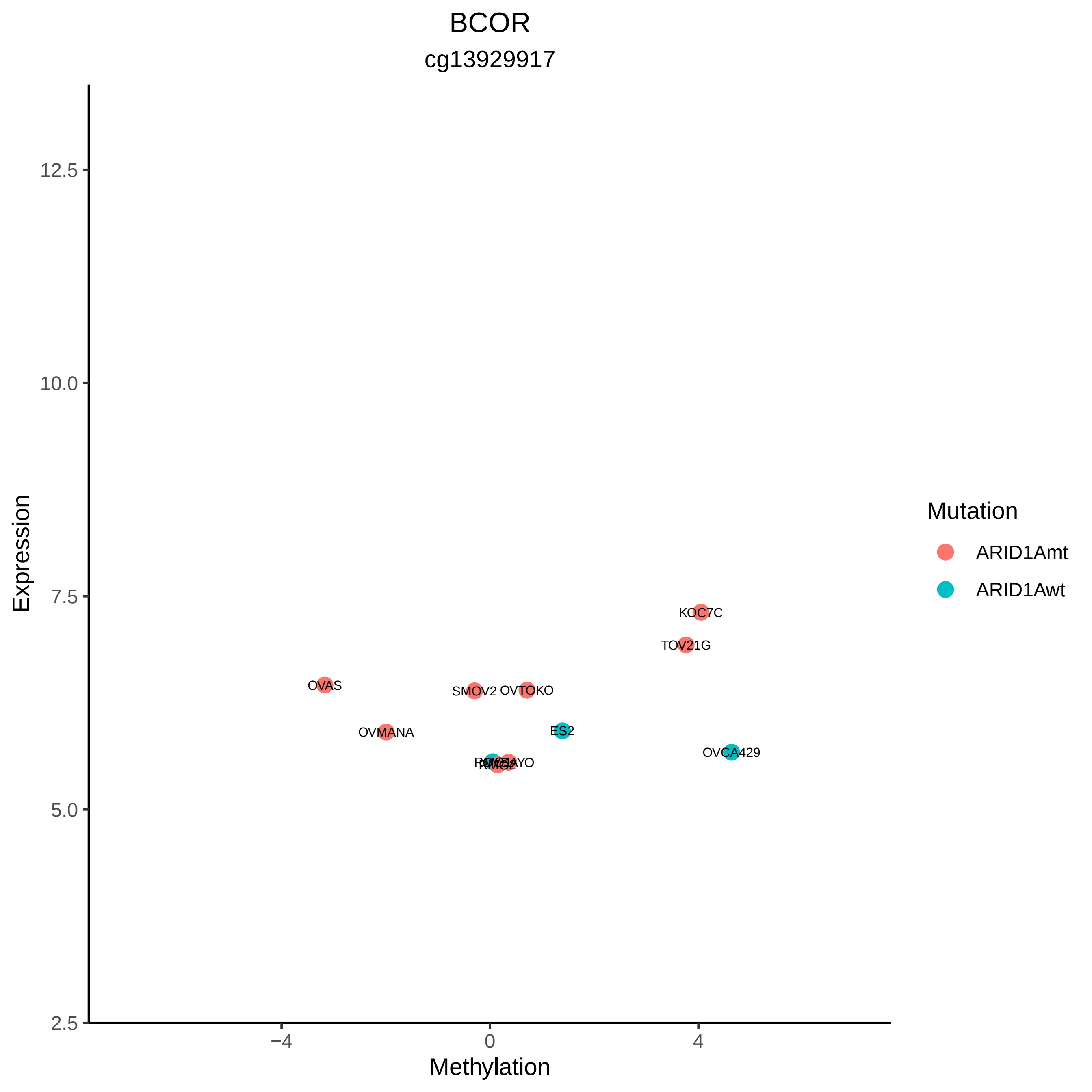

BCOR
cg13929917
Expression
Methylation
12.5
10.0
7.5
5.0
2.5
−4
0
4
KOC7C
TOV21G
OVAS
SMOV2
OVTOKO
OVMANA
ES2
OVCA429
Mutation
ARID1Amt
ARID1Awt

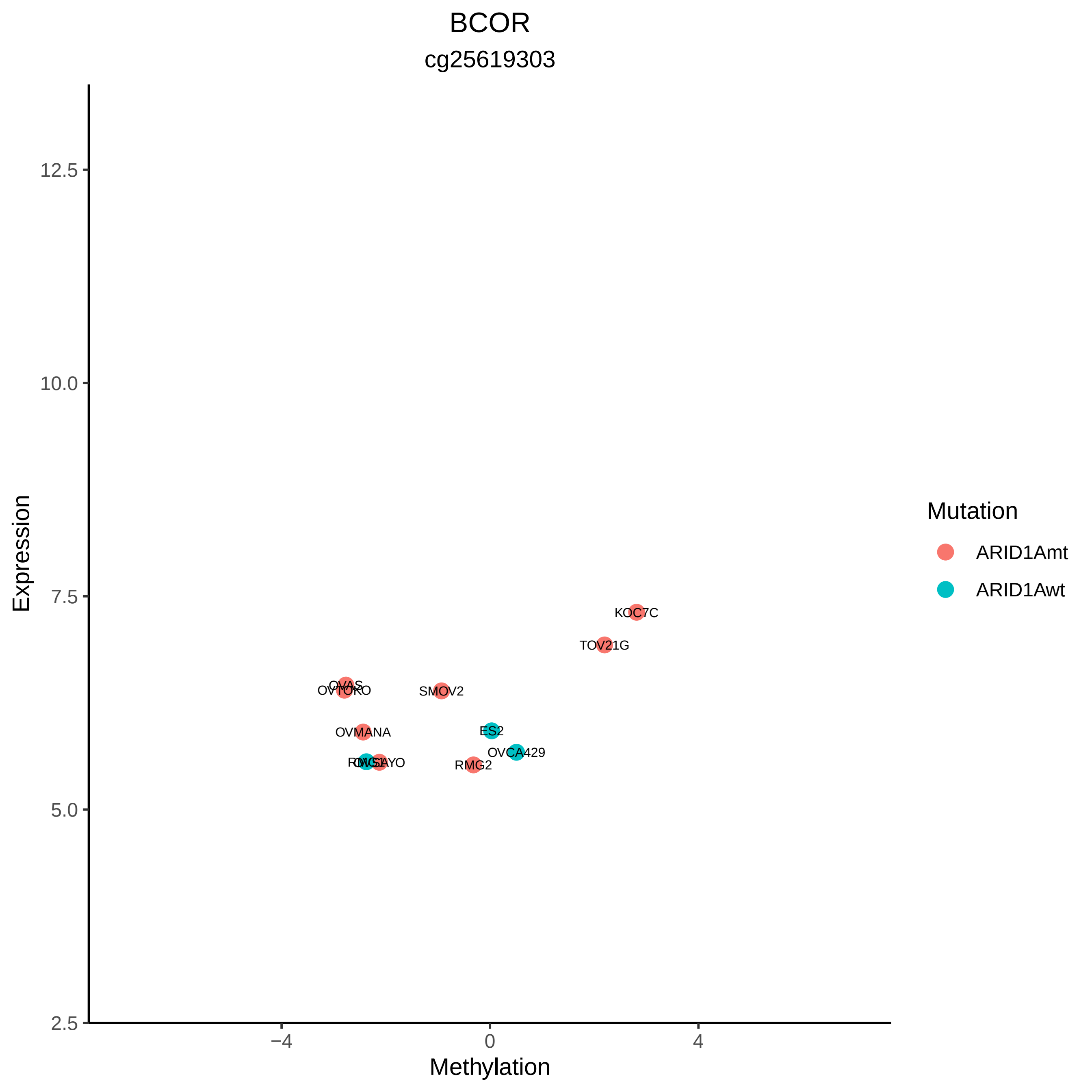

BCOR
cg25619303
Expression
Methylation
12.5
10.0
7.5
5.0
2.5
−4
0
4
KOC7C
TOV21G
OVAS
OVTOKO
SMOV2
OVMANA
ES2
OVCA429
RMG2
Mutation
ARID1Amt
ARID1Awt

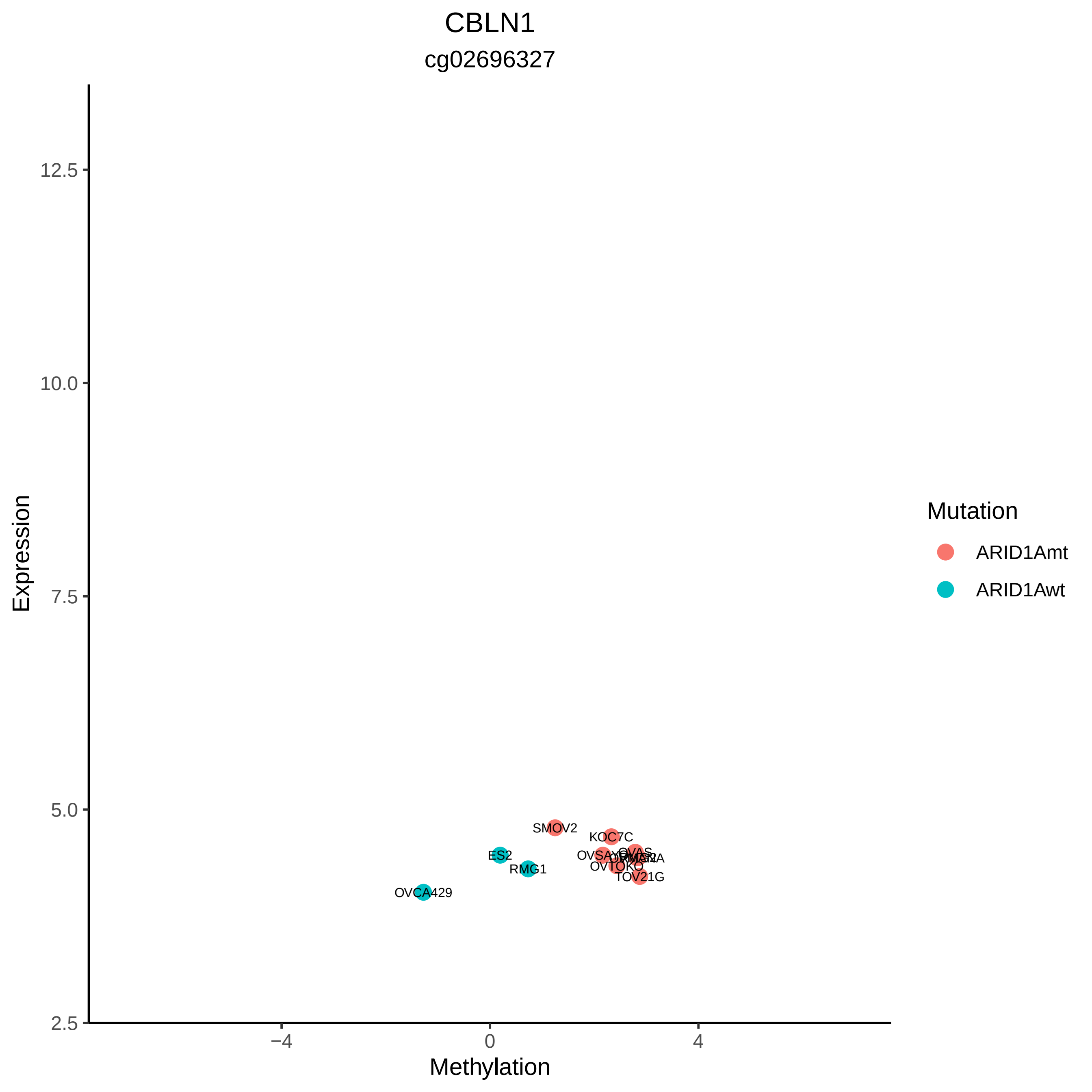

CBLN1
cg02696327
Expression
Methylation
12.5
10.0
7.5
5.0
2.5
−4
0
4
SMOV2
KOC7C
ES2
RMG1
OVTOKO
TOV21G
OVCA429
Mutation
ARID1Amt
ARID1Awt

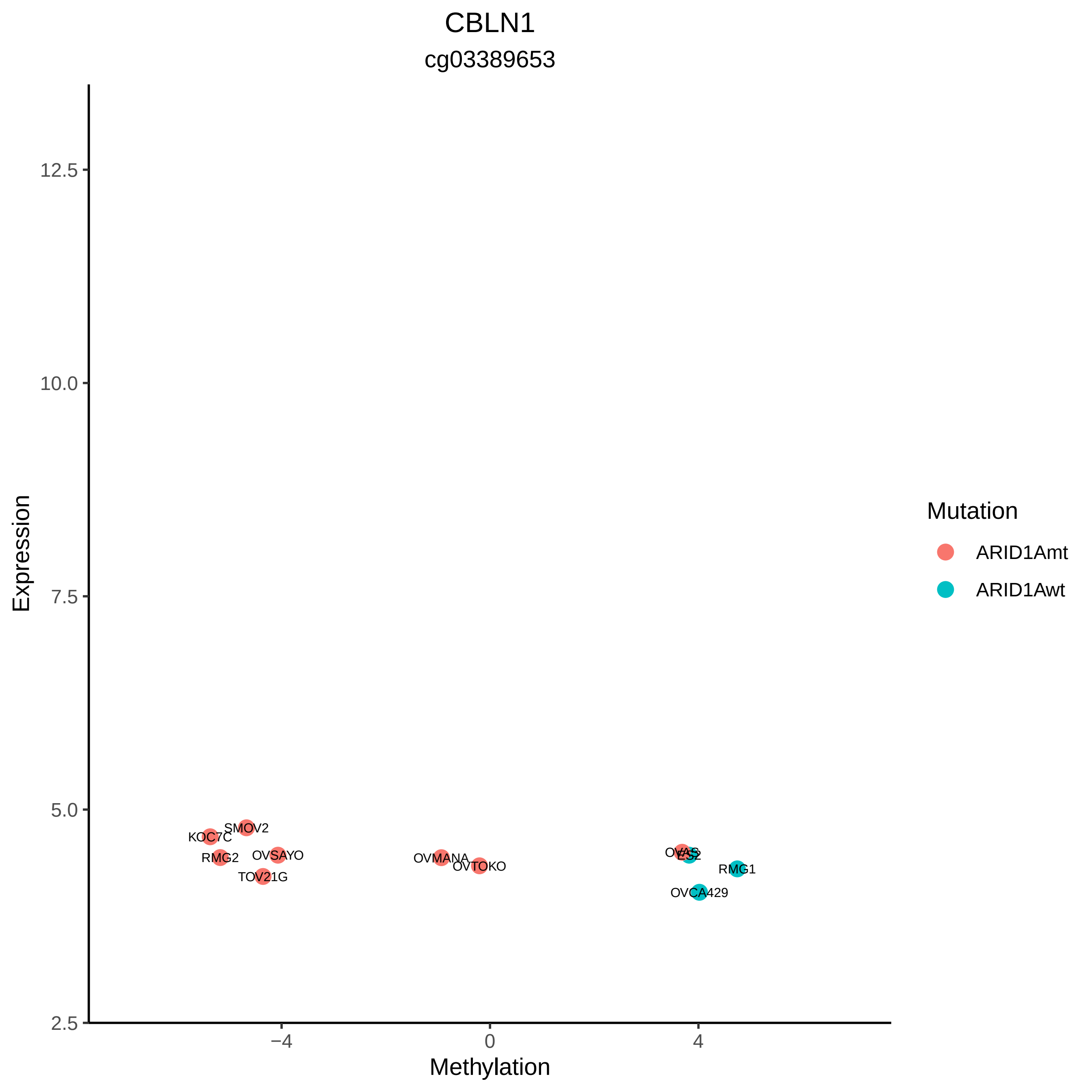
CBLN1
cg03389653
Expression
Methylation
12.5
10.0
7.5
5.0
2.5
−4
0
4
SMOV2
KOC7C
RMG2
OVSAYO
TOV21G
OVMANA
OVTOKO
OVAS
ES2
RMG1
OVCA429
Mutation
ARID1Amt
ARID1Awt

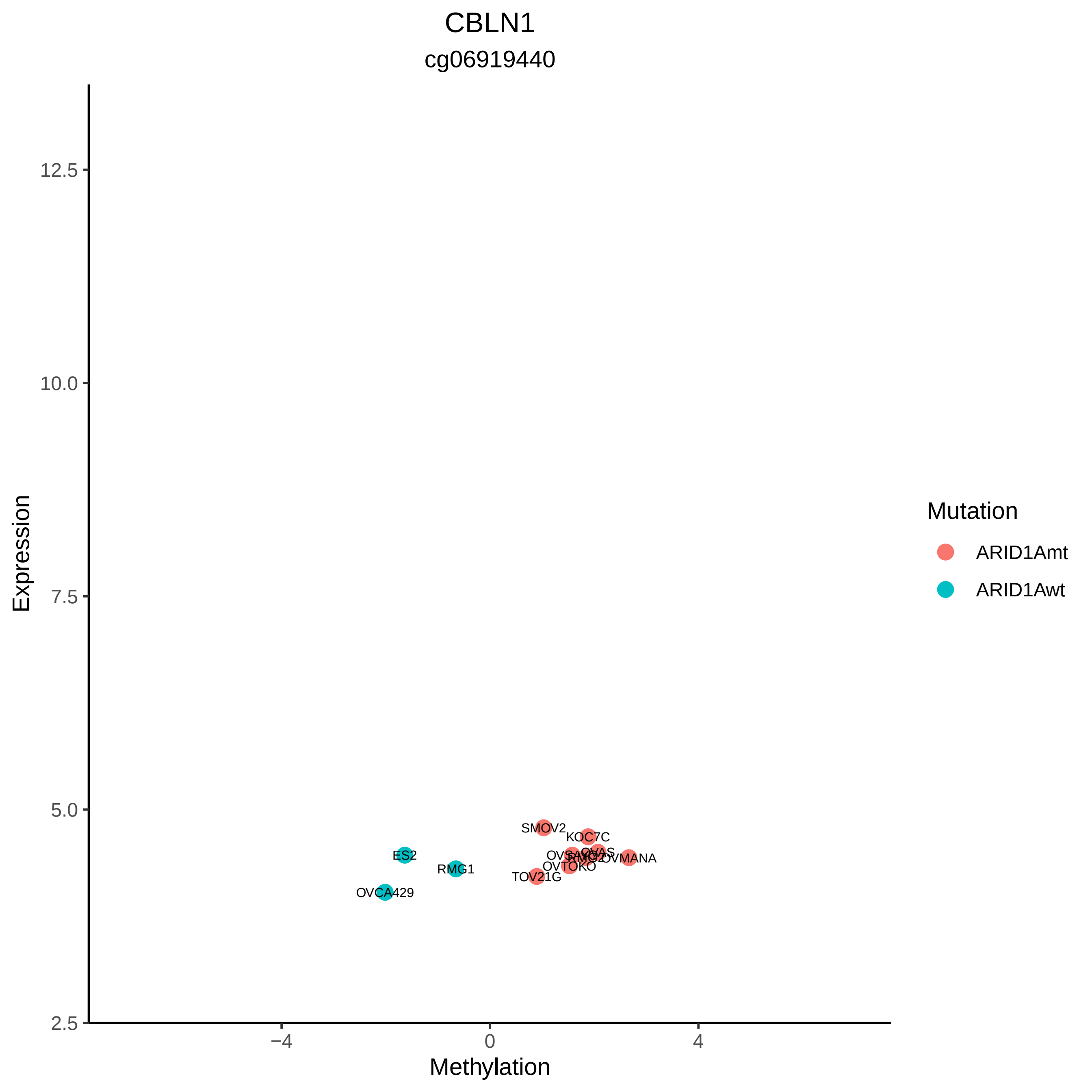
CBLN1
cg06919440
Expression
Methylation
12.5
10.0
7.5
5.0
2.5
−4
0
4
SMOV2
KOC7C
ES2
RMG1
OVTOKO
TOV21G
OVCA429
Mutation
ARID1Amt
ARID1Awt

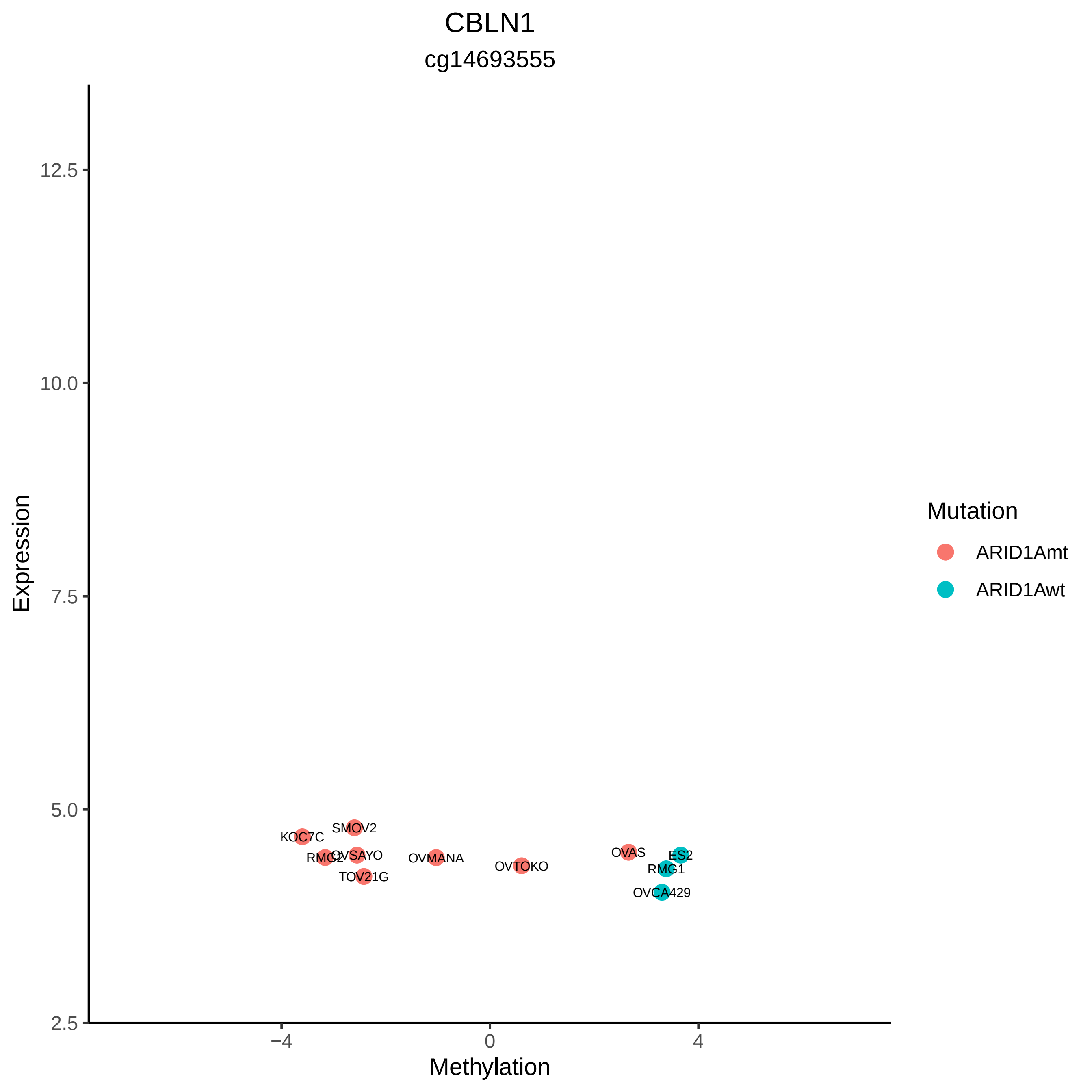

CBLN1
cg14693555
Expression
12.5
10.0
7.5
5.0
2.5
−4
0
4
Methylation
KOC7C
SMOV2
RMG2
OVSAYO
TOV21G
OVMANA
OVTOKO
OVAS
ES2
RMG1
OVCA429
Mutation
ARID1Amt
ARID1Awt

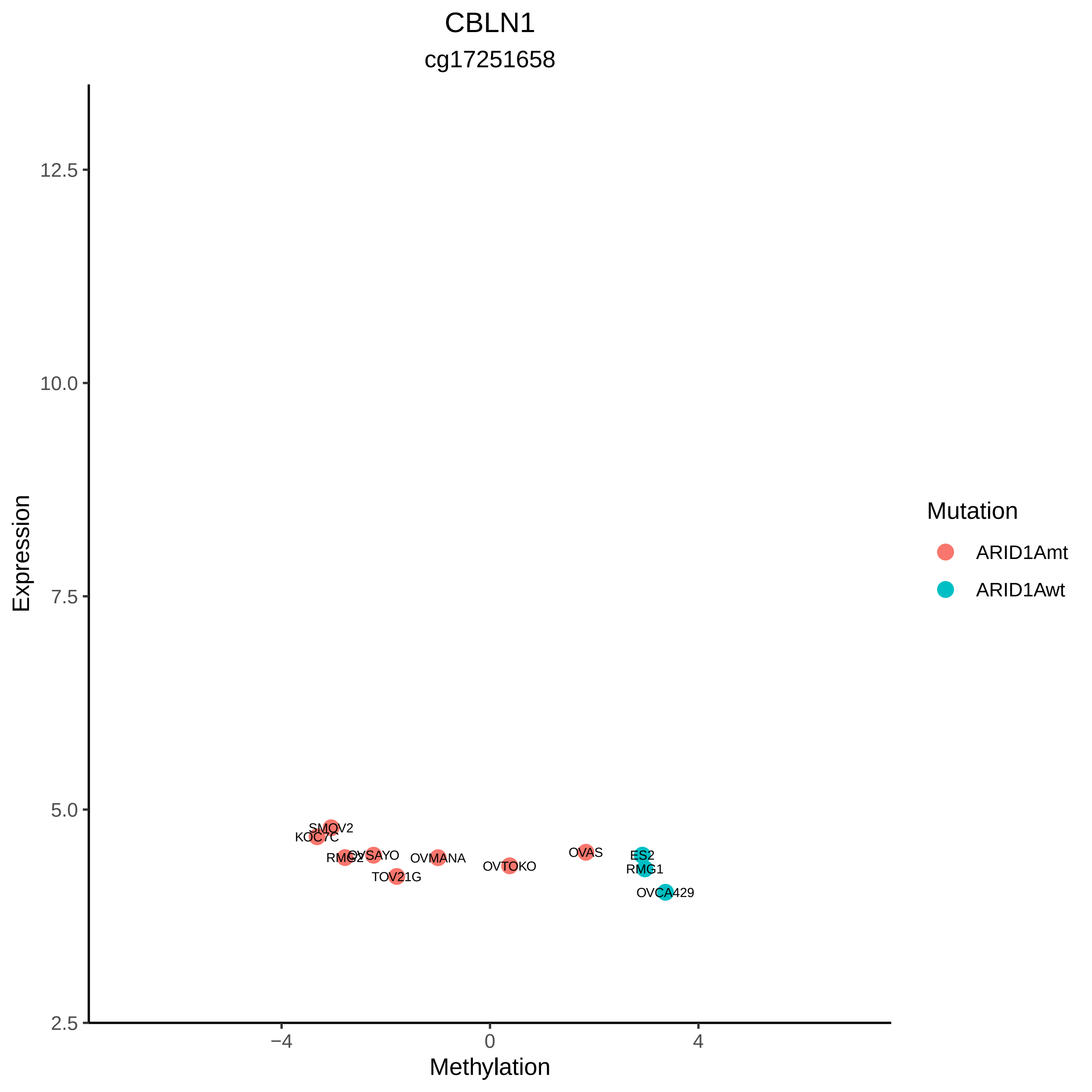

CBLN1
cg17251658
Expression
Methylation
12.5
10.0
7.5
5.0
2.5
−4
0
4
SMOV2
KOC7C
RMG2
OVSAYO
OVMANA
TOV21G
OVTOKO
OVAS
ES2
RMG1
OVCA429
Mutation
ARID1Amt
ARID1Awt

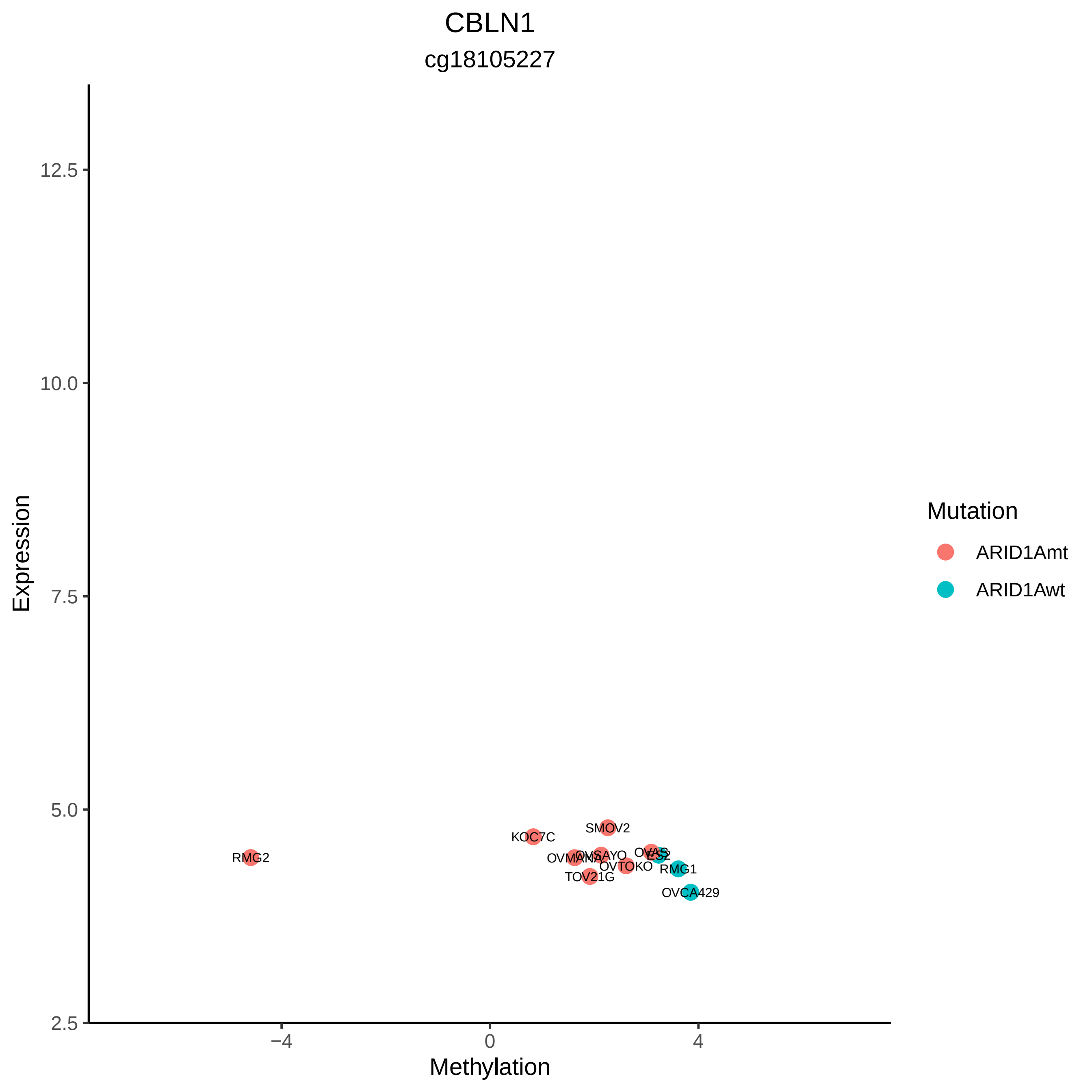

CBLN1
cg18105227
Expression
12.5
10.0
7.5
5.0
2.5
Methylation
−4
0
4
RMG2
KOC7C
SMOV2
OVMANA
OVSAYO
OVTOKO
TOV21G
OVAS
ES2
RMG1
OVCA429
Mutation
ARID1Amt
ARID1Awt

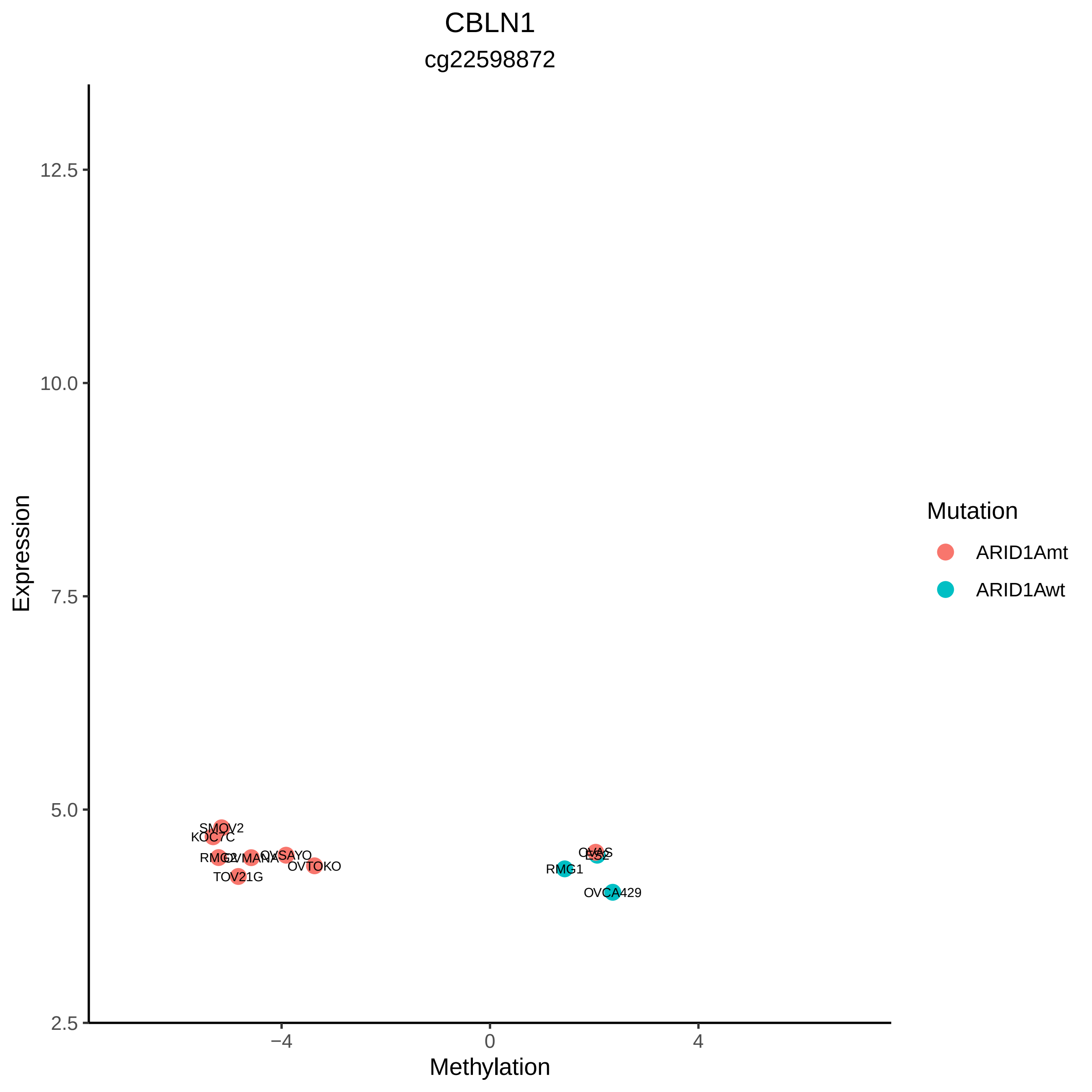
CBLN1
cg22598872
Expression
Methylation
12.5
10.0
7.5
5.0
2.5
−4
0
4
SMOV2
KOC7C
RMG2
OVMANA
OVSAYO
OVTOKO
TOV21G
OVAS
ES2
RMG1
OVCA429
Mutation
ARID1Amt
ARID1Awt

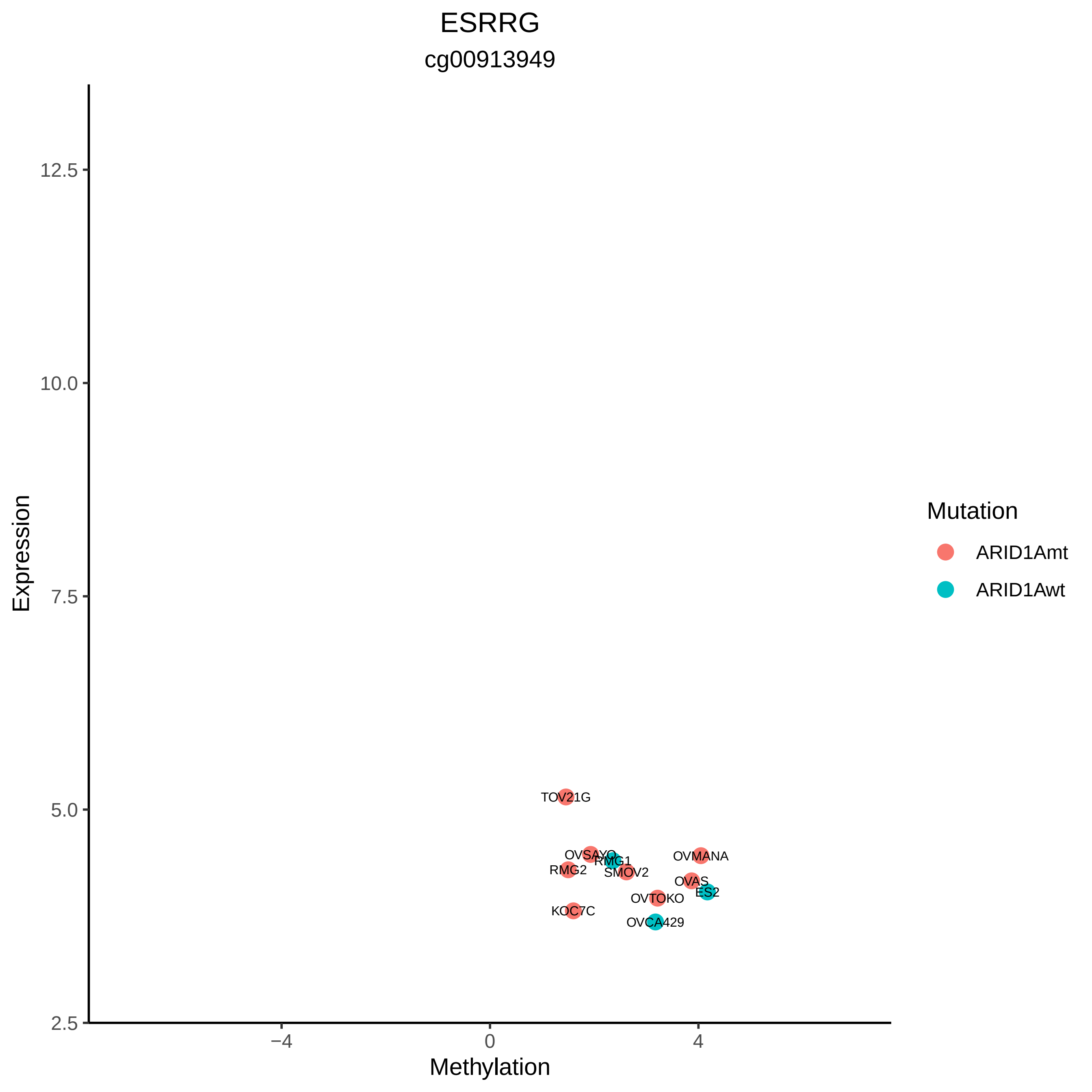

ESRRG
cg00913949
Expression
Methylation
12.5
10.0
7.5
5.0
2.5
−4
0
4
TOV21G
OVSAYO
RMG1
RMG2
SMOV2
OVMANA
OVAS
ES2
OVTOKO
KOC7C
OVCA429
Mutation
ARID1Amt
ARID1Awt

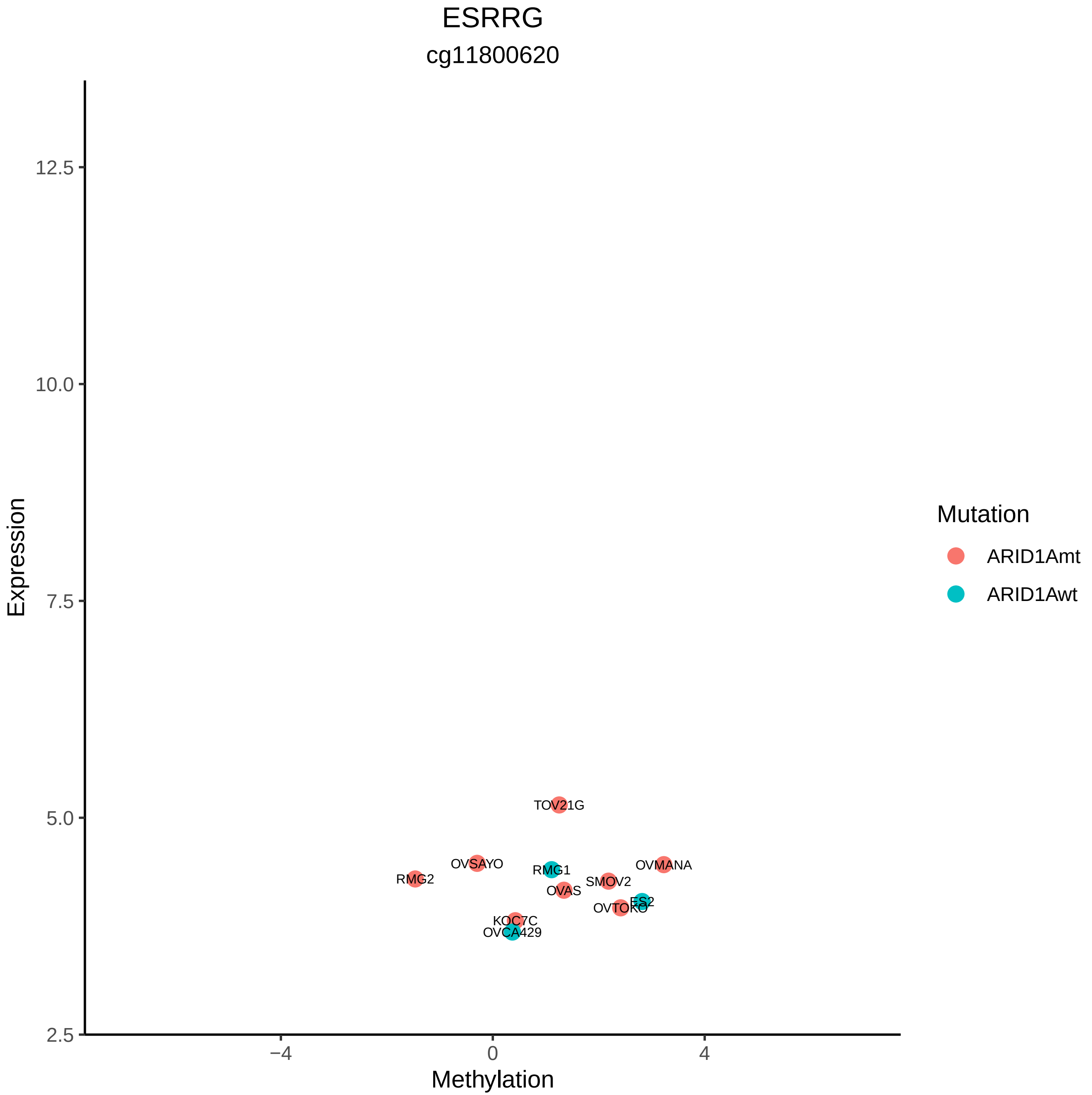

ESRRG
cg11800620
Expression
Methylation
12.5
10.0
7.5
5.0
2.5
−4
0
4
TOV21G
OVSAYO
RMG2
RMG1
OVMANA
SMOV2
OVAS
ES2
OVTOKO
KOC7C
OVCA429
Mutation
ARID1Amt
ARID1Awt

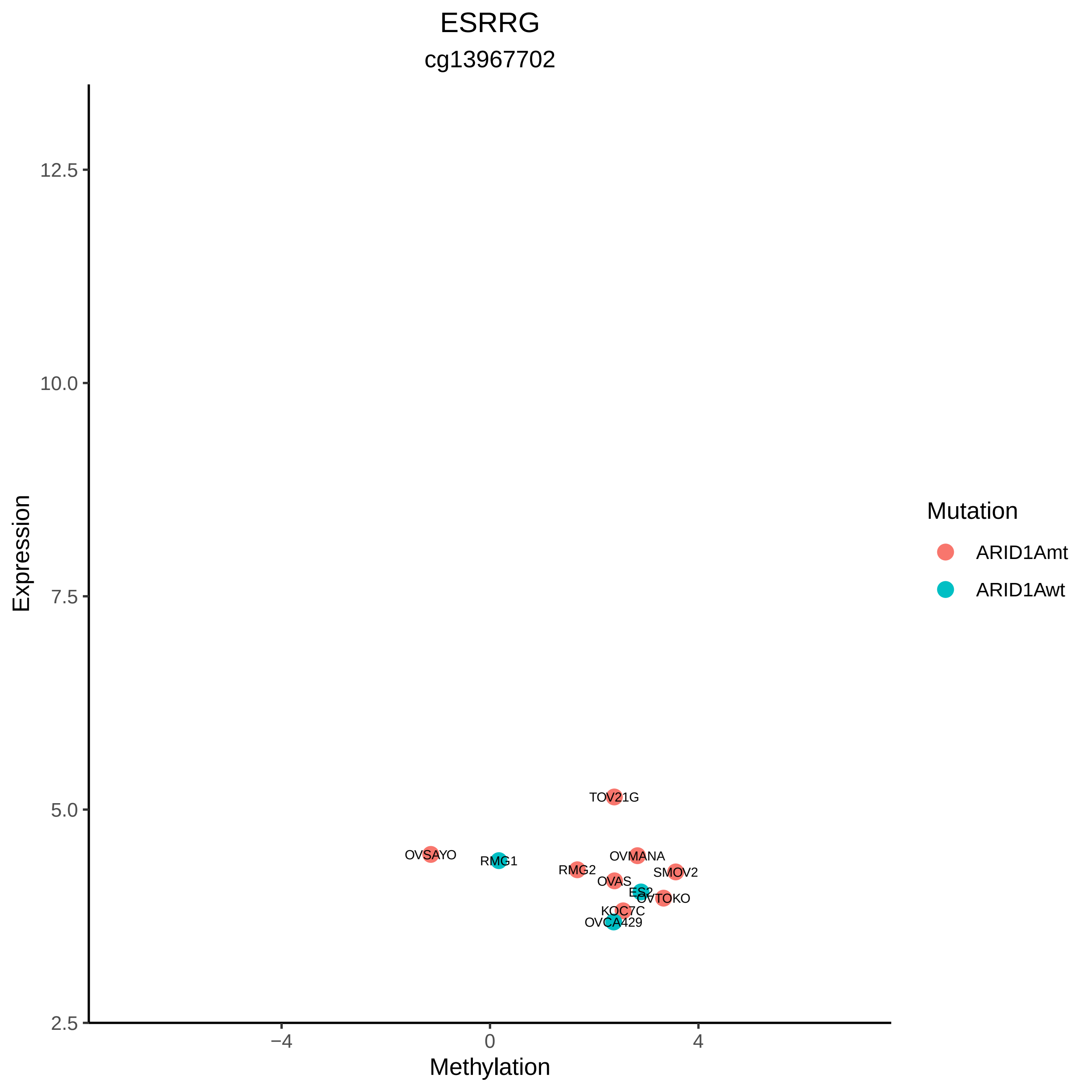
ESRRG
cg13967702
Expression
12.5
10.0
7.5
5.0
2.5
Methylation
−4
0
4
TOV21G
OVSAYO
RMG1
OVMANA
RMG2
SMOV2
OVAS
ES2
OVTOKO
KOC7C
OVCA429
Mutation
ARID1Amt
ARID1Awt

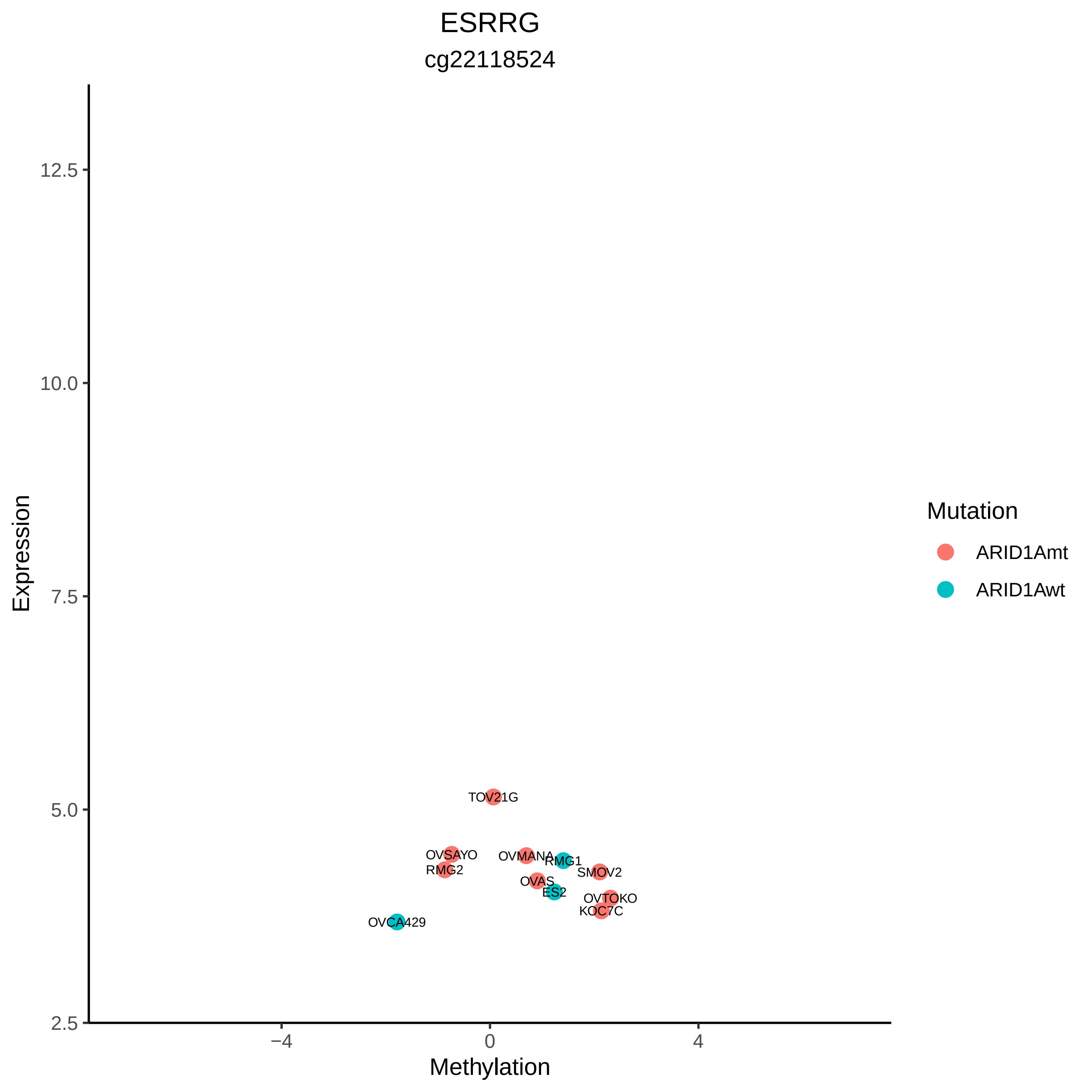

ESRRG
cg22118524
Expression
Methylation
12.5
10.0
7.5
5.0
2.5
−4
0
4
TOV21G
OVSAYO
RMG2
OVMANA
RMG1
SMOV2
OVAS
ES2
OVTOKO
KOC7C
OVCA429
Mutation
ARID1Amt
ARID1Awt

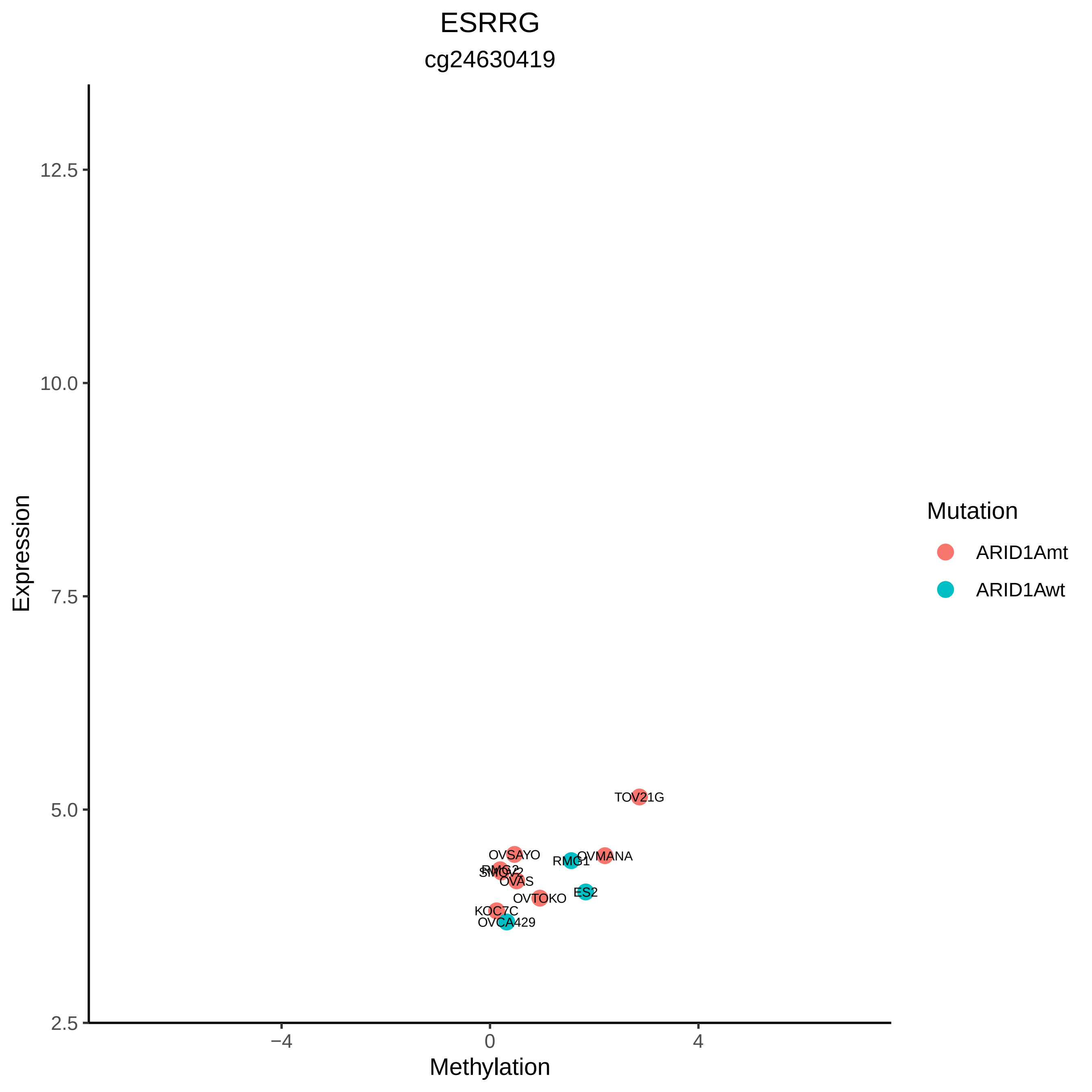
ESRRG
cg24630419
Expression
12.5
10.0
7.5
5.0
2.5
Methylation
−4
0
4
TOV21G
OVSAYO
OVMANA
RMG1
RMG2
SMOV2
OVAS
ES2
OVTOKO
KOC7C
OVCA429
Mutation
ARID1Amt
ARID1Awt

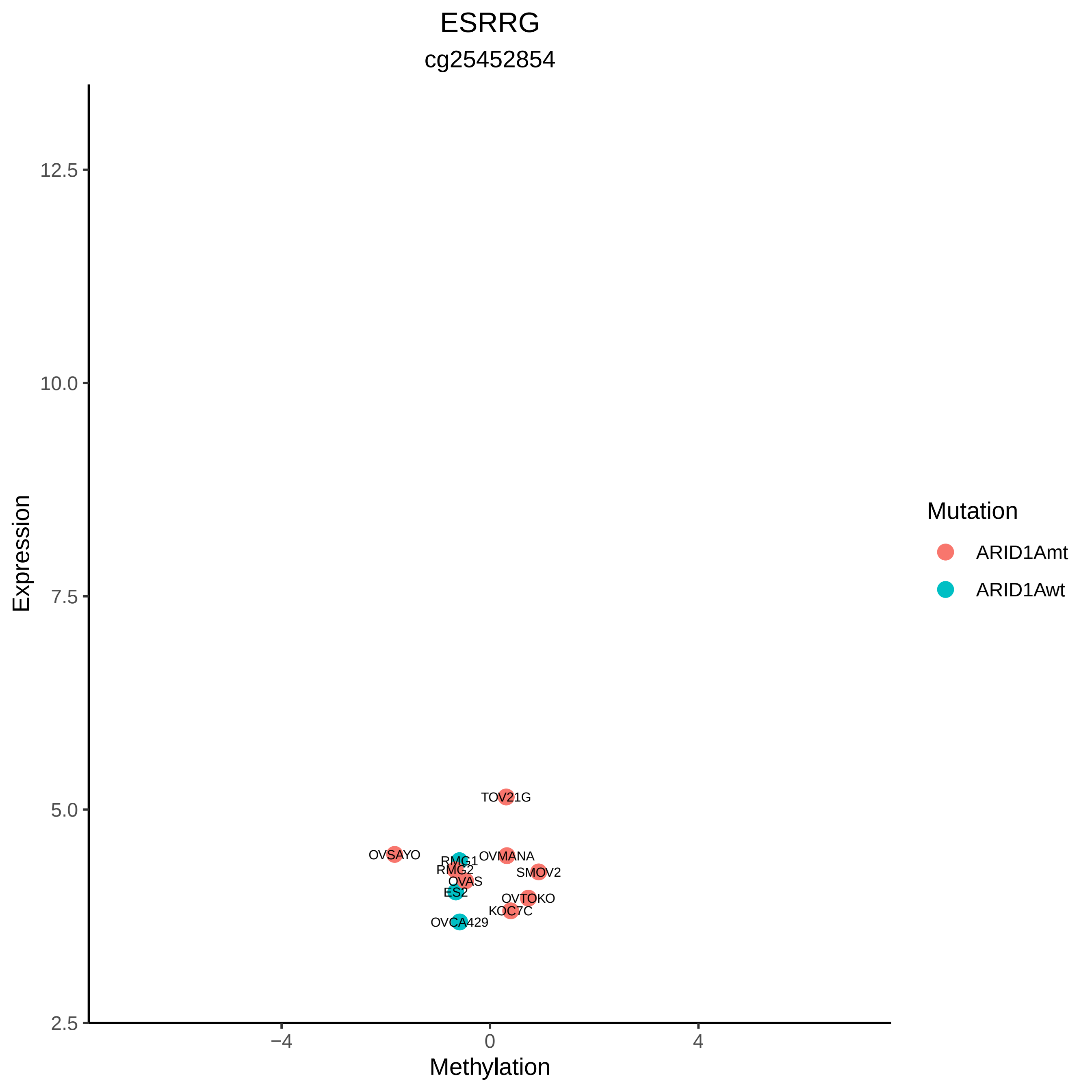
ESRRG
cg25452854
Expression
12.5
10.0
7.5
5.0
2.5
Methylation
−4
0
4
TOV21G
OVSAYO
RMG1
OVMANA
RMG2
SMOV2
OVAS
ES2
OVTOKO
KOC7C
OVCA429
Mutation
ARID1Amt
ARID1Awt

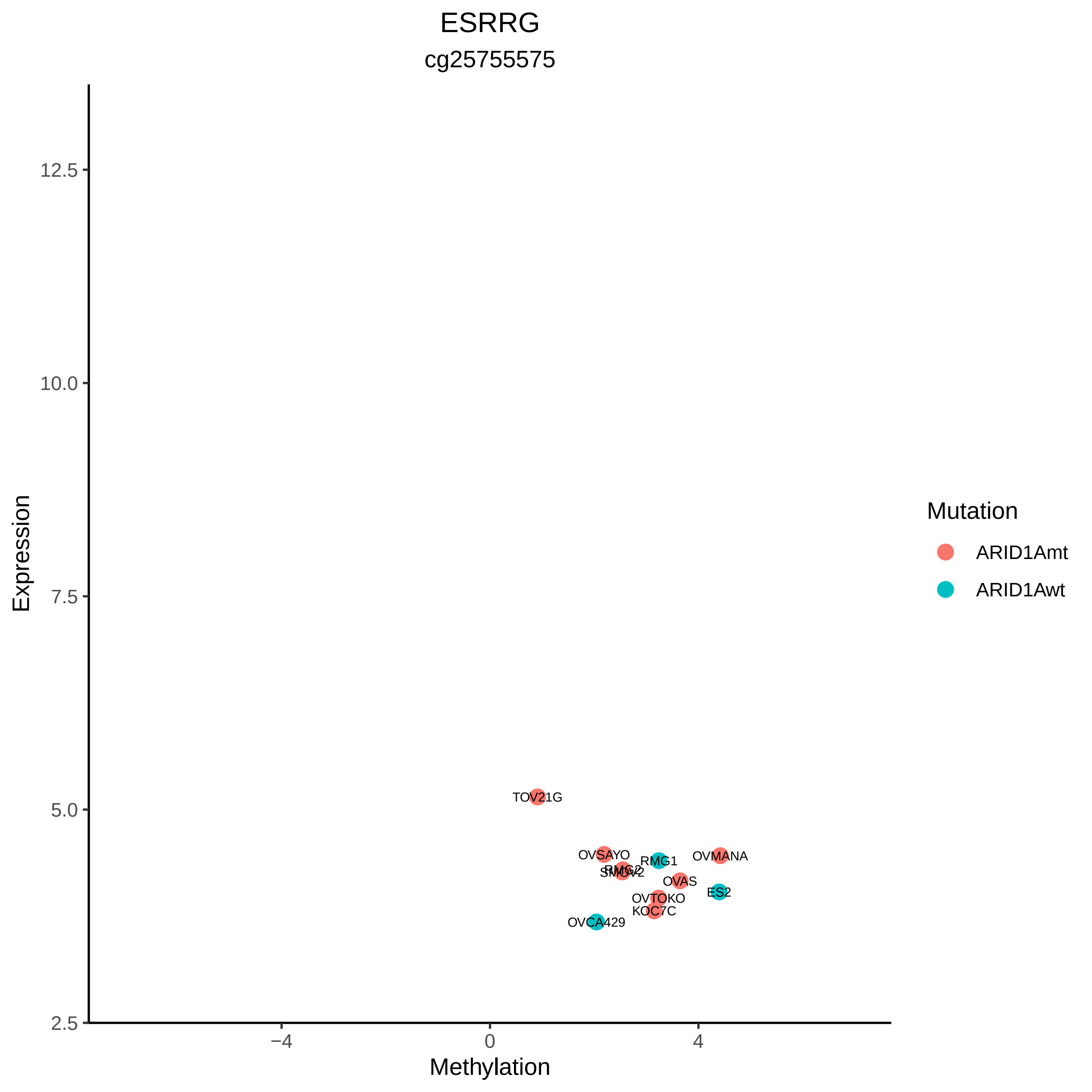

ESRRG
cg25755575
Expression
Methylation
12.5
10.0
7.5
5.0
2.5
−4
0
4
TOV21G
OVSAYO
RMG2
SMOV2
RMG1
OVMANA
OVAS
ES2
OVTOKO
KOC7C
OVCA429
Mutation
ARID1Amt
ARID1Awt

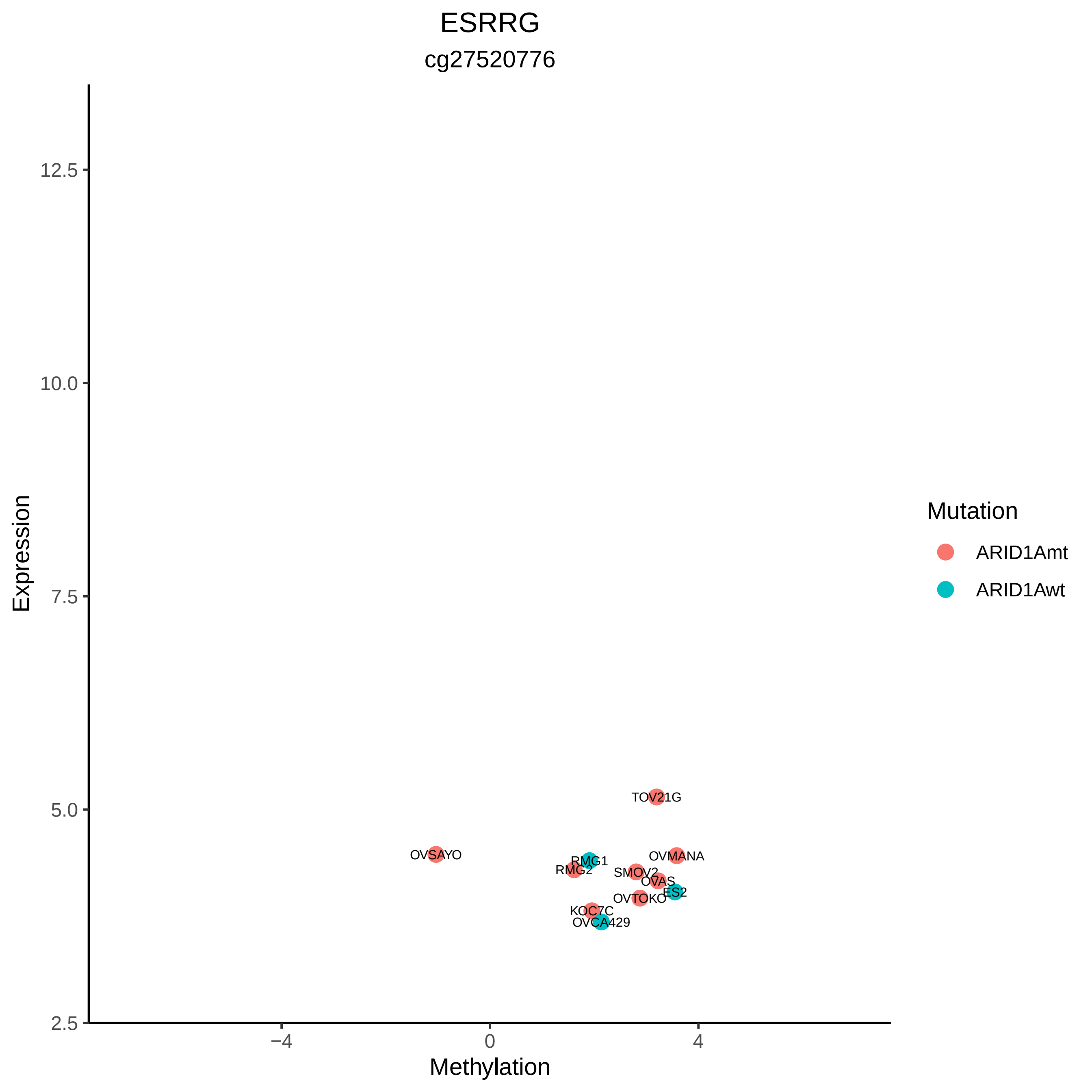
ESRRG
cg27520776
Expression
Methylation
12.5
10.0
7.5
5.0
2.5
−4
0
4
TOV21G
OVSAYO
OVMANA
RMG1
RMG2
SMOV2
OVAS
ES2
OVTOKO
KOC7C
OVCA429
Mutation
ARID1Amt
ARID1Awt

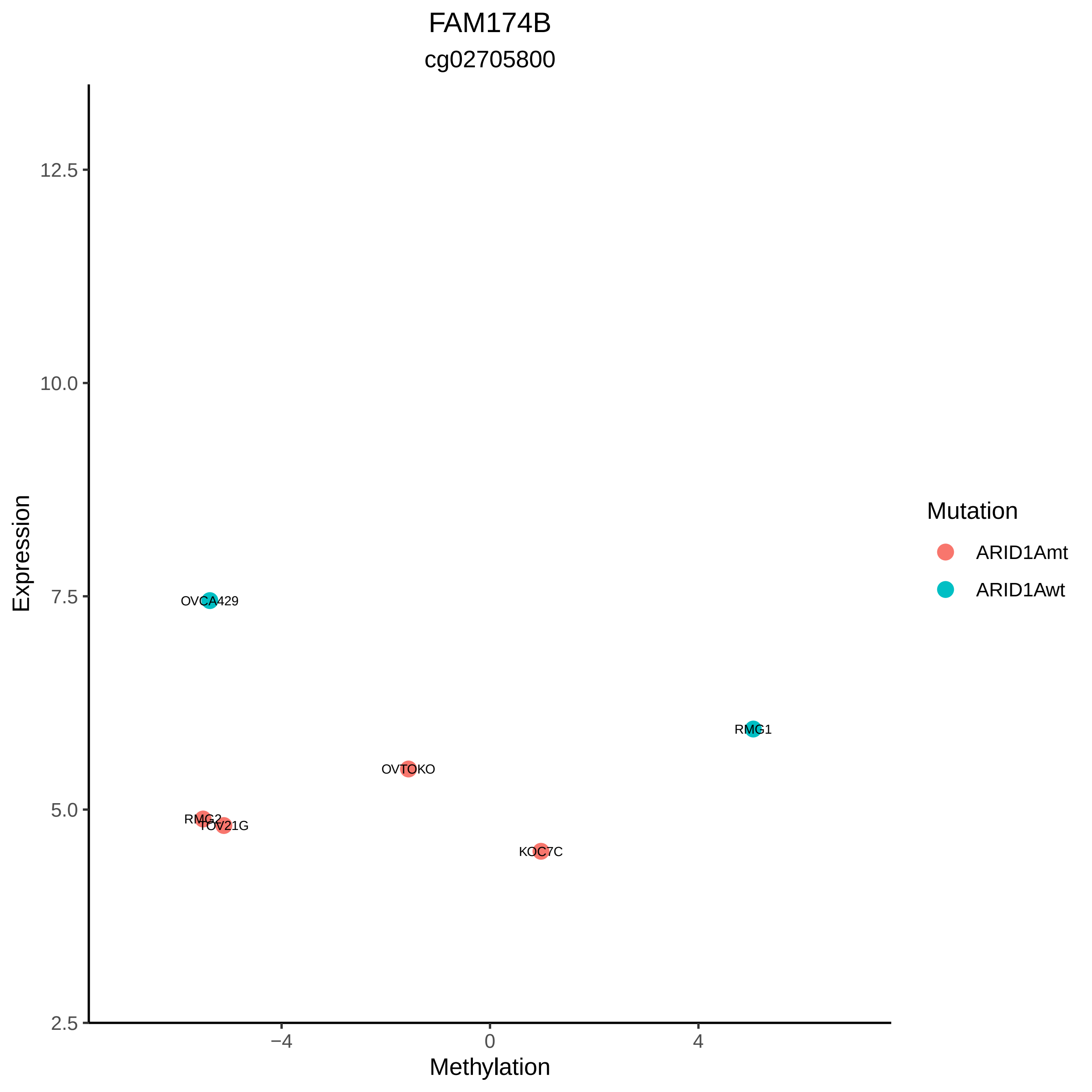
FAM174B
cg02705800
Expression
Methylation
12.5
10.0
7.5
5.0
2.5
−4
0
4
OVCA429
RMG1
OVTOKO
RMG2
TOV21G
KOC7C
Mutation
ARID1Amt
ARID1Awt

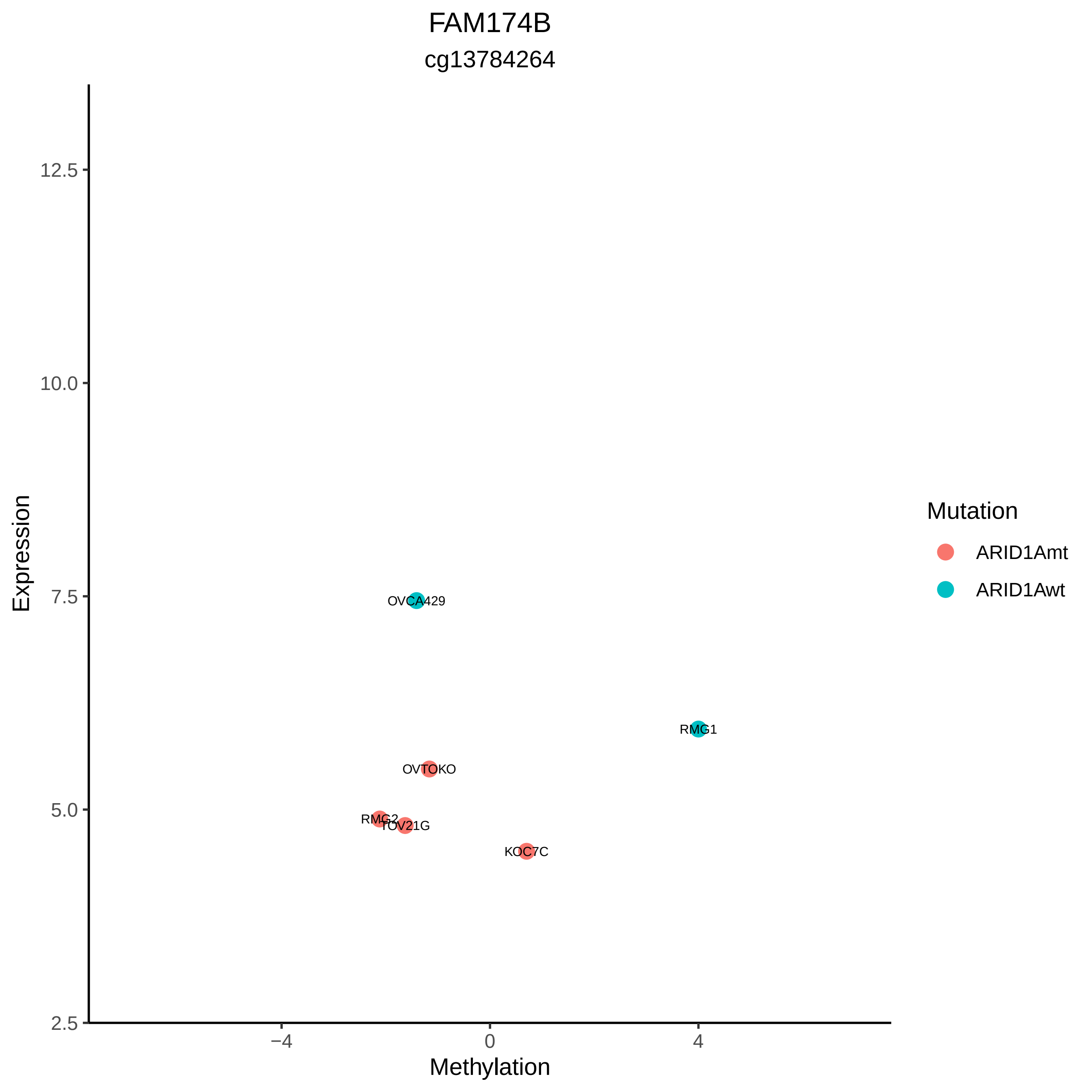
FAM174B
cg13784264
Expression
Methylation
12.5
10.0
7.5
5.0
2.5
−4
0
4
OVCA429
RMG1
OVTOKO
RMG2
TOV21G
KOC7C
Mutation
ARID1Amt
ARID1Awt

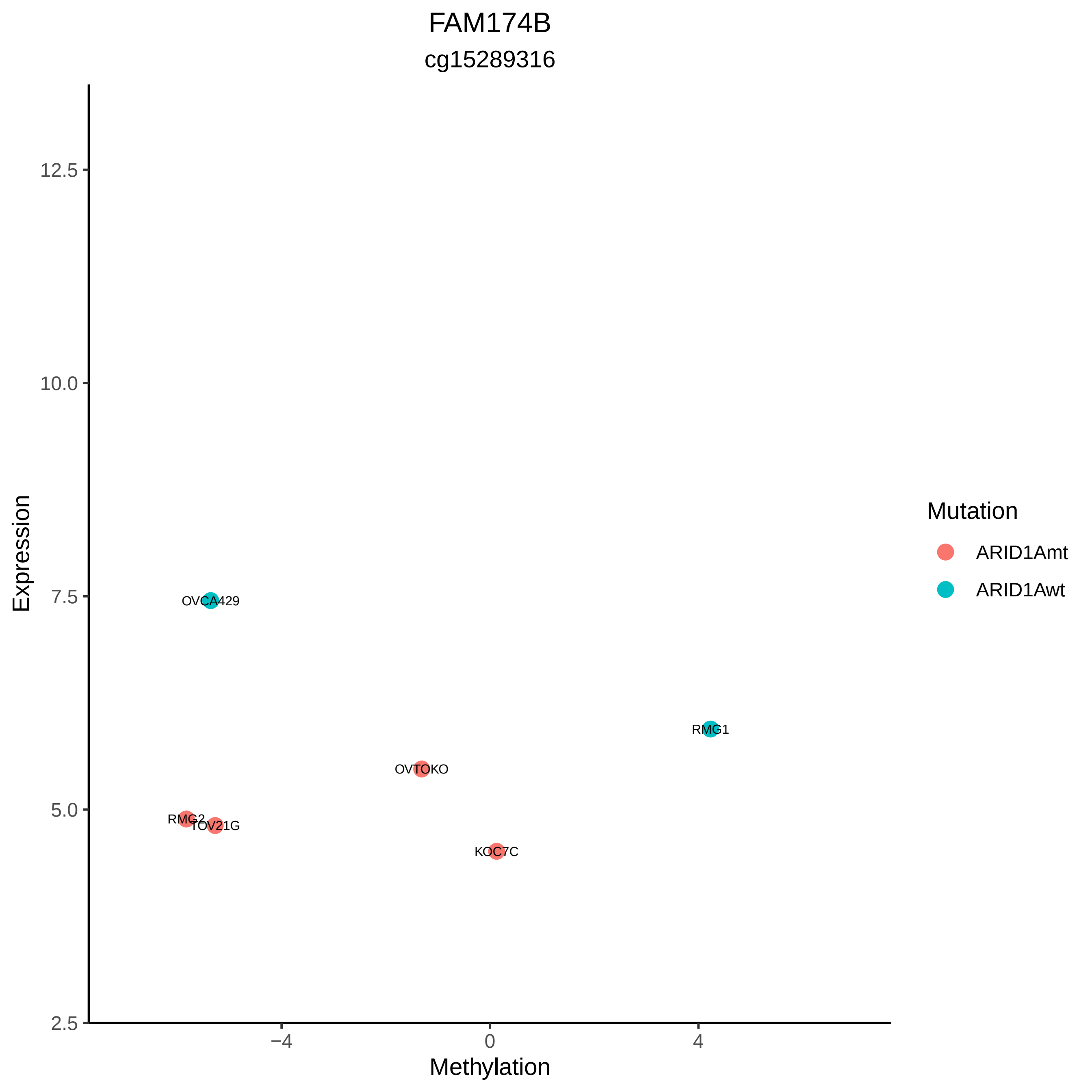

FAM174B
cg15289316
Expression
12.5
10.0
7.5
5.0
2.5
Methylation
−4
0
4
OVCA429
RMG1
OVTOKO
RMG2
TOV21G
KOC7C
Mutation
ARID1Amt
ARID1Awt

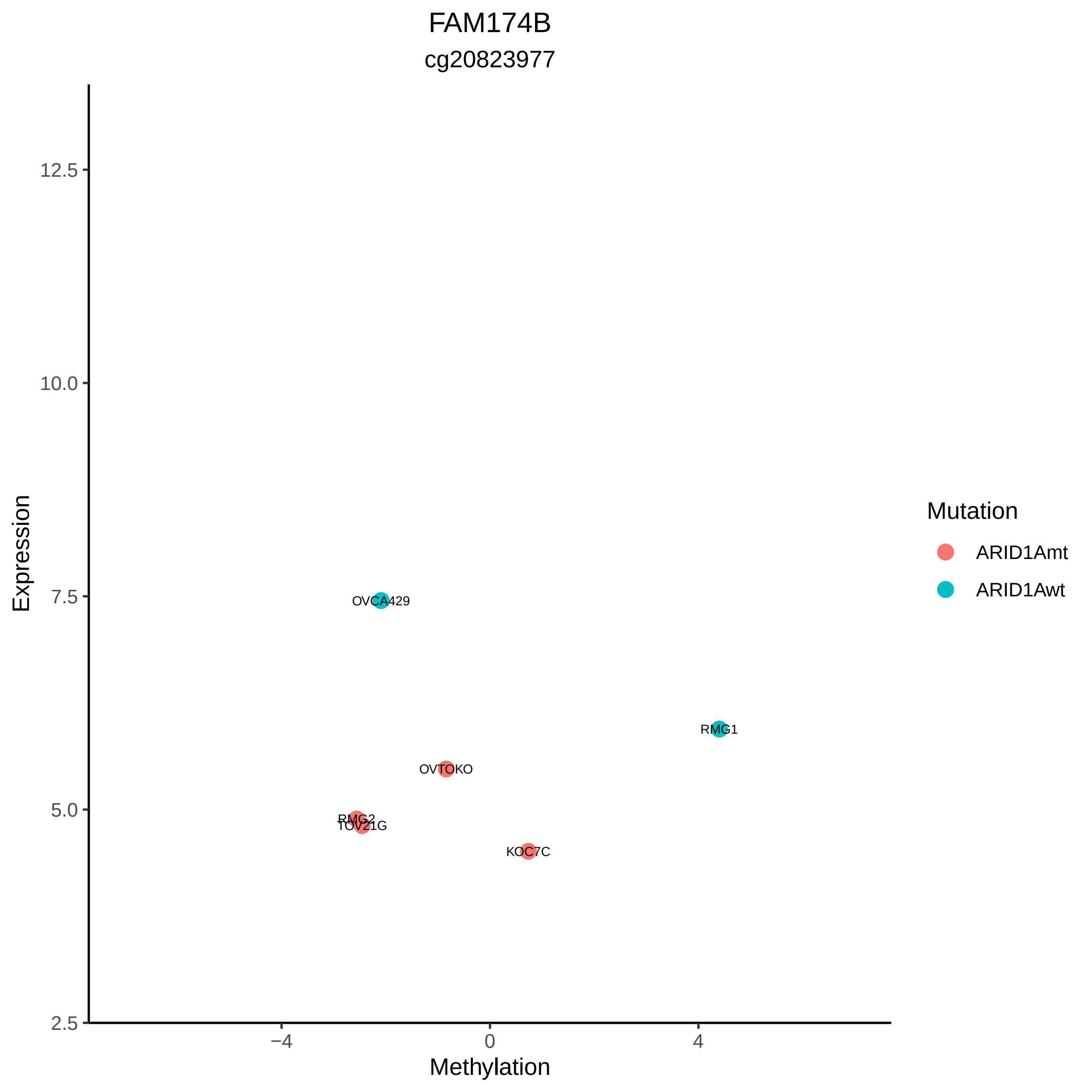
FAM174B
cg20823977
Expression
12.5
10.0
7.5
5.0
2.5
Methylation
−4
0
4
OVCA429
RMG1
OVTOKO
RMG2
TOV21G
KOC7C
Mutation
ARID1Amt
ARID1Awt

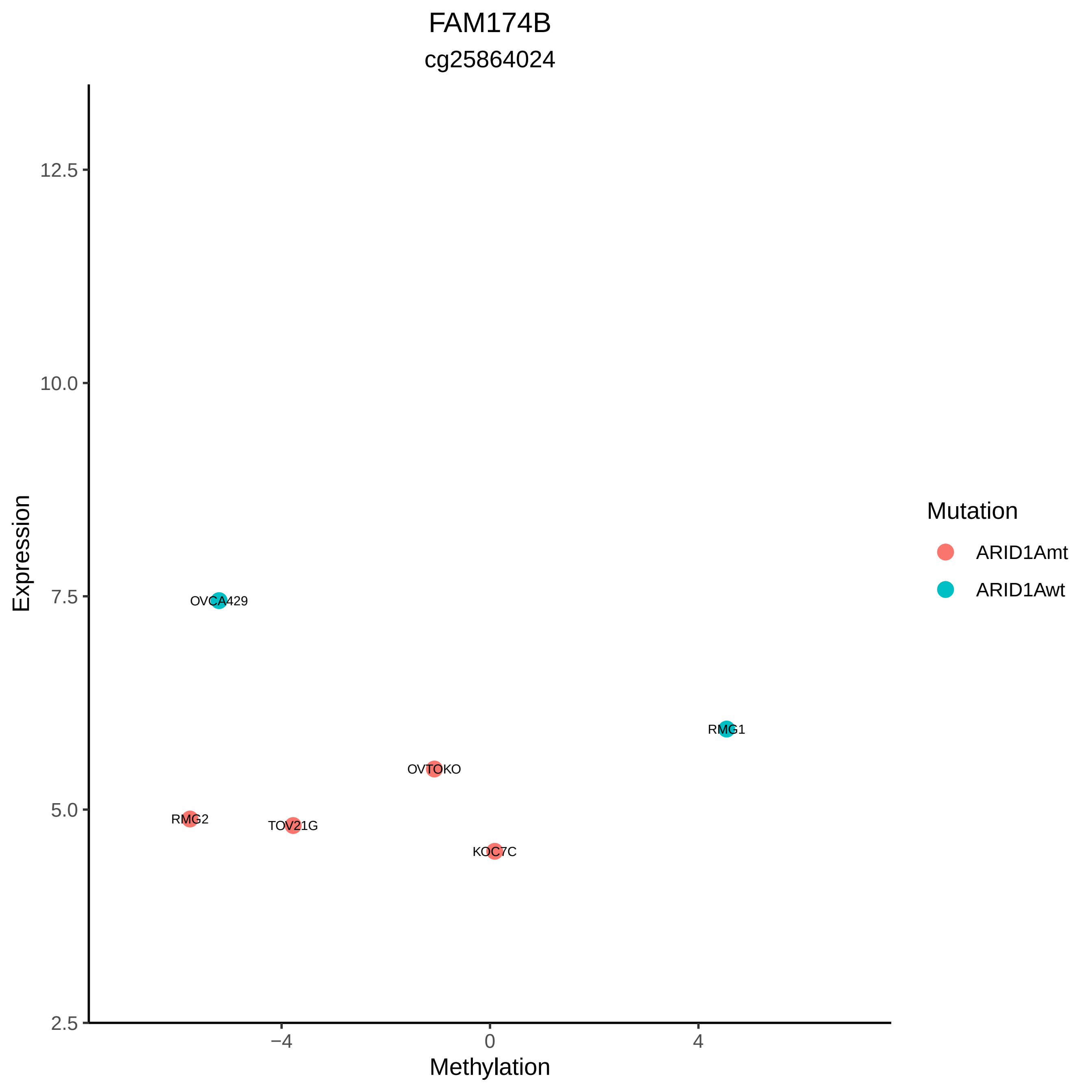

FAM174B
cg25864024
Expression
Methylation
12.5
10.0
7.5
5.0
2.5
−4
0
4
OVCA429
RMG1
OVTOKO
RMG2
TOV21G
KOC7C
Mutation
ARID1Amt
ARID1Awt

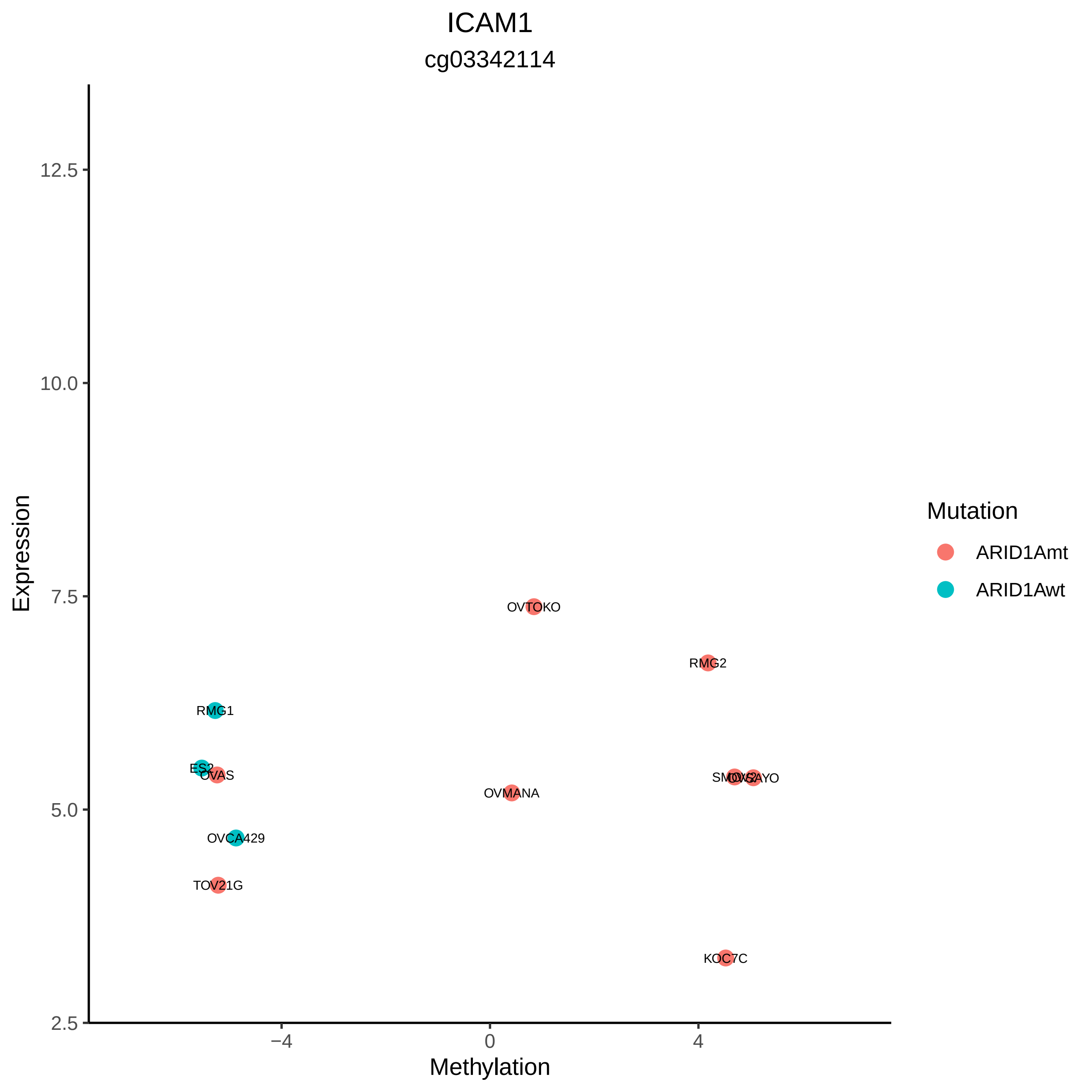

ICAM1
cg03342114
Expression
Methylation
12.5
10.0
7.5
5.0
2.5
−4
0
4
OVTOKO
RMG2
RMG1
ES2
OVAS
OVMANA
SMOV2
OVSAYO
OVCA429
TOV21G
KOC7C
Mutation
ARID1Amt
ARID1Awt

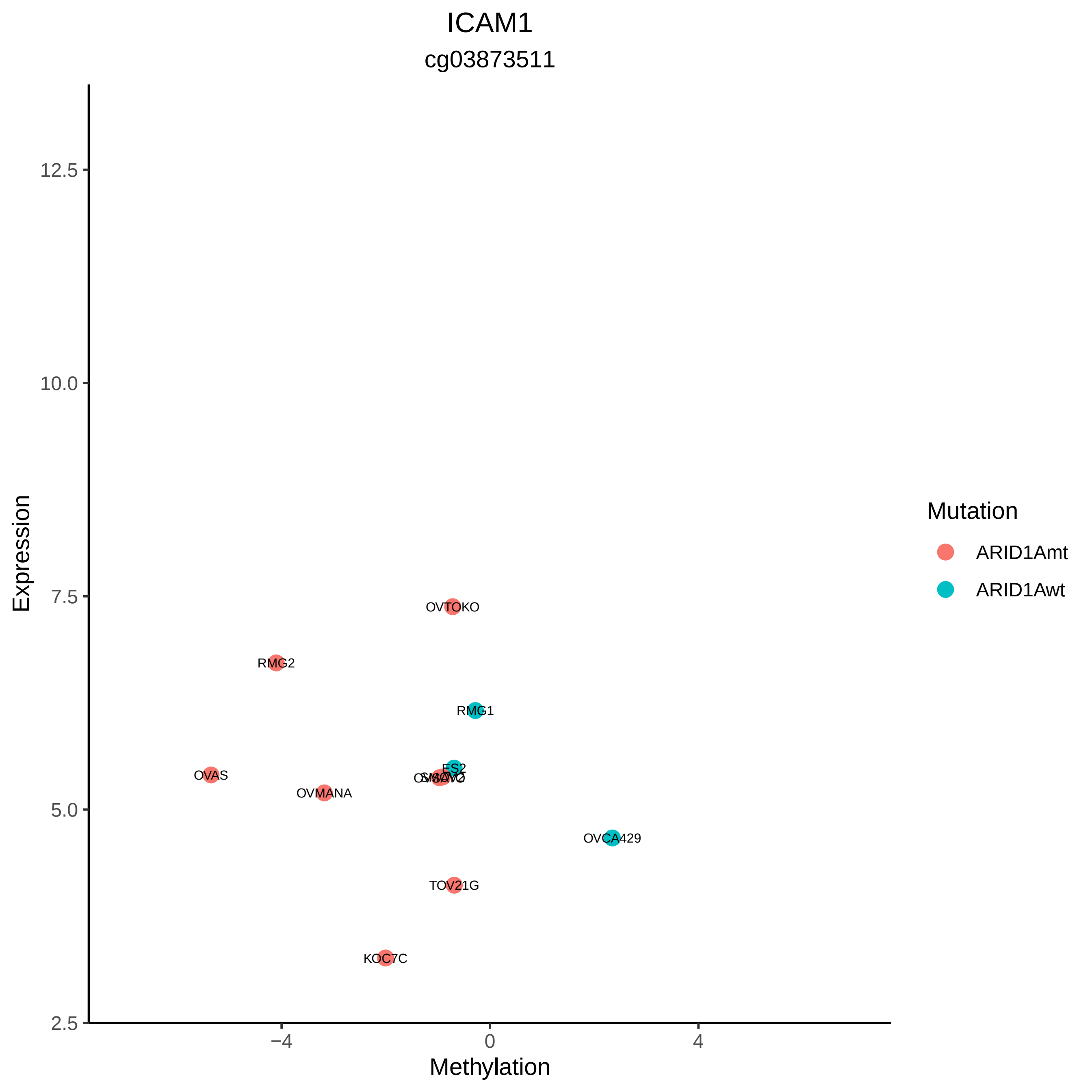
ICAM1
cg03873511
Expression
Methylation
12.5
10.0
7.5
5.0
2.5
−4
0
4
OVTOKO
RMG2
RMG1
ES2
OVAS
OVMANA
OVCA429
TOV21G
KOC7C
Mutation
ARID1Amt
ARID1Awt

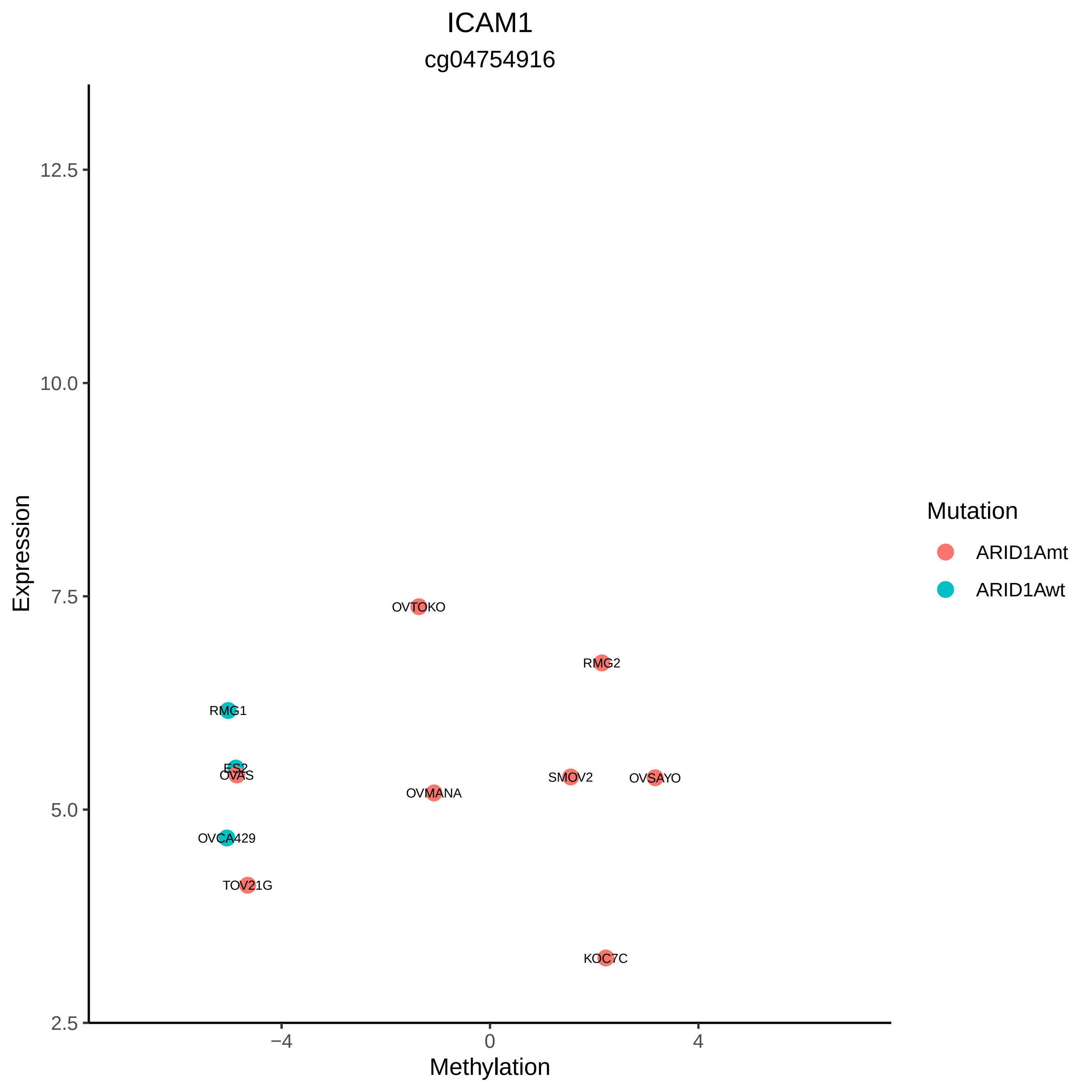
ICAM1
cg04754916
Expression
Methylation
12.5
10.0
7.5
5.0
2.5
−4
0
4
OVTOKO
RMG2
RMG1
ES2
OVAS
SMOV2
OVSAYO
OVMANA
OVCA429
TOV21G
KOC7C
Mutation
ARID1Amt
ARID1Awt

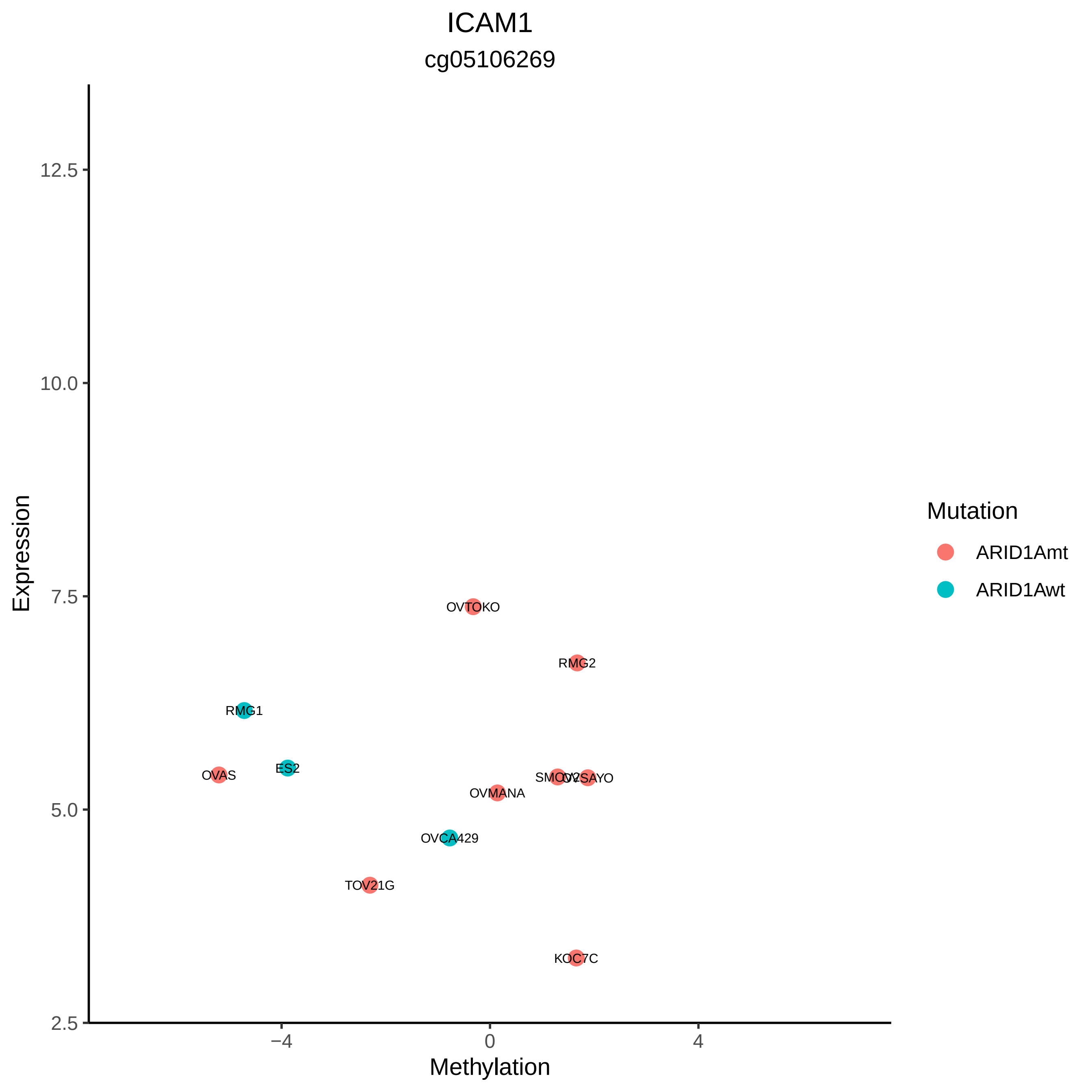

ICAM1
cg05106269
Expression
Methylation
12.5
10.0
7.5
5.0
2.5
−4
0
4
OVTOKO
RMG2
RMG1
ES2
OVAS
SMOV2
OVSAYO
OVMANA
OVCA429
TOV21G
KOC7C
Mutation
ARID1Amt
ARID1Awt

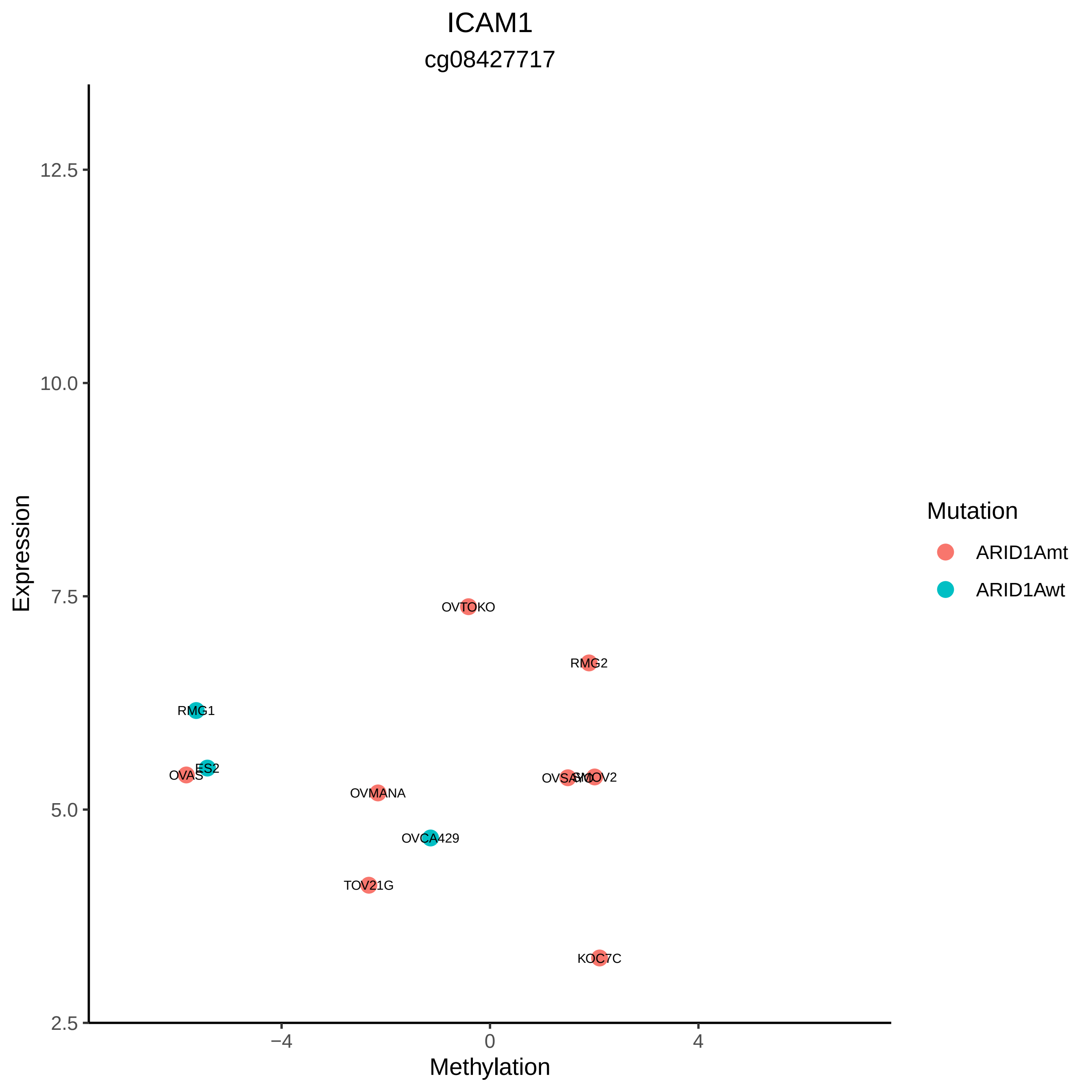

ICAM1
cg08427717
Expression
Methylation
12.5
10.0
7.5
5.0
2.5
−4
0
4
OVTOKO
RMG2
RMG1
ES2
OVAS
OVMANA
OVSAHO
SMOV2
OVCA429
TOV21G
KOC7C
Mutation
ARID1Amt
ARID1Awt

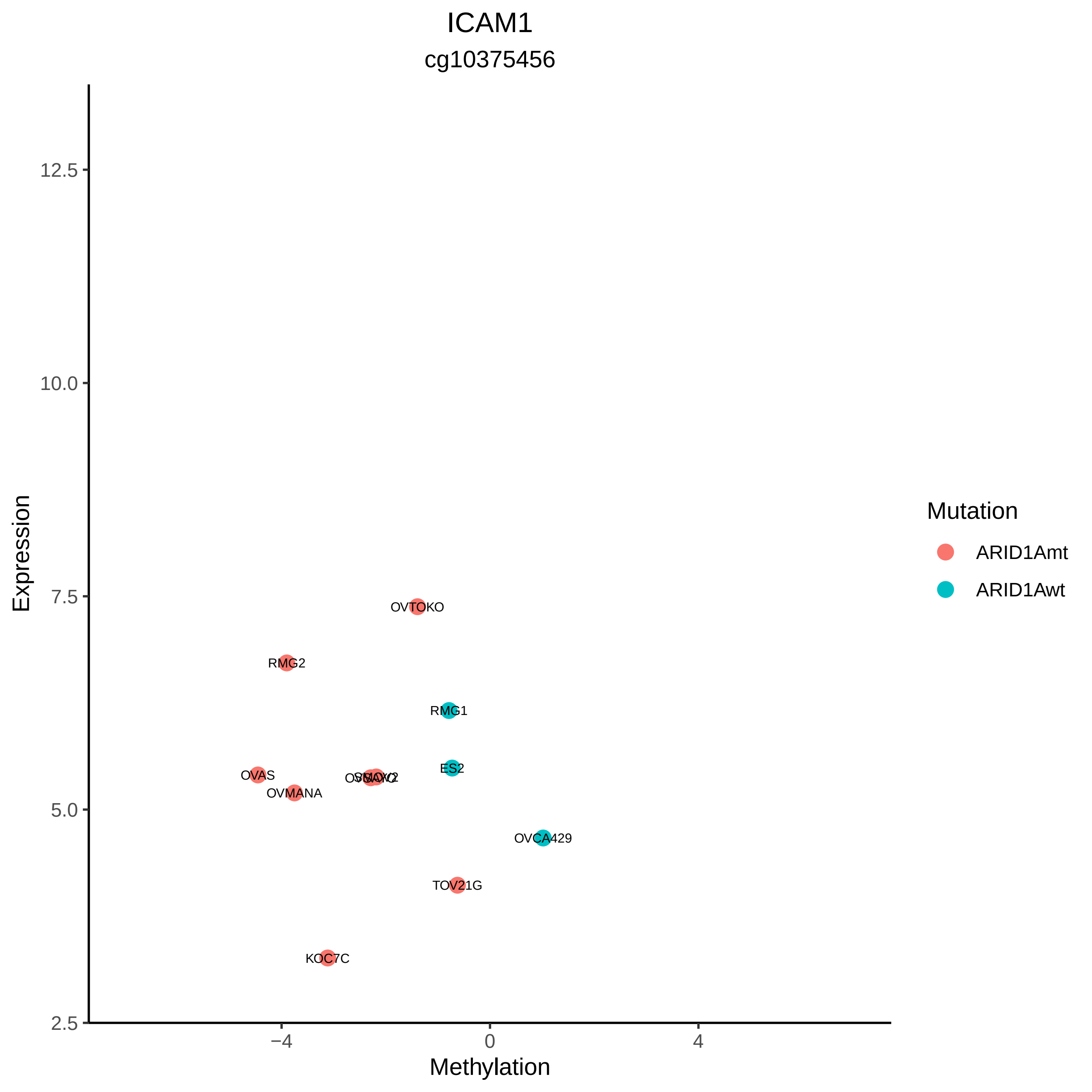
ICAM1
cg10375456
Expression
Methylation
12.5
10.0
7.5
5.0
2.5
−4
0
4
OVTOKO
RMG2
RMG1
ES2
OVAS
OVMANA
OVCA429
TOV21G
KOC7C
Mutation
ARID1Amt
ARID1Awt

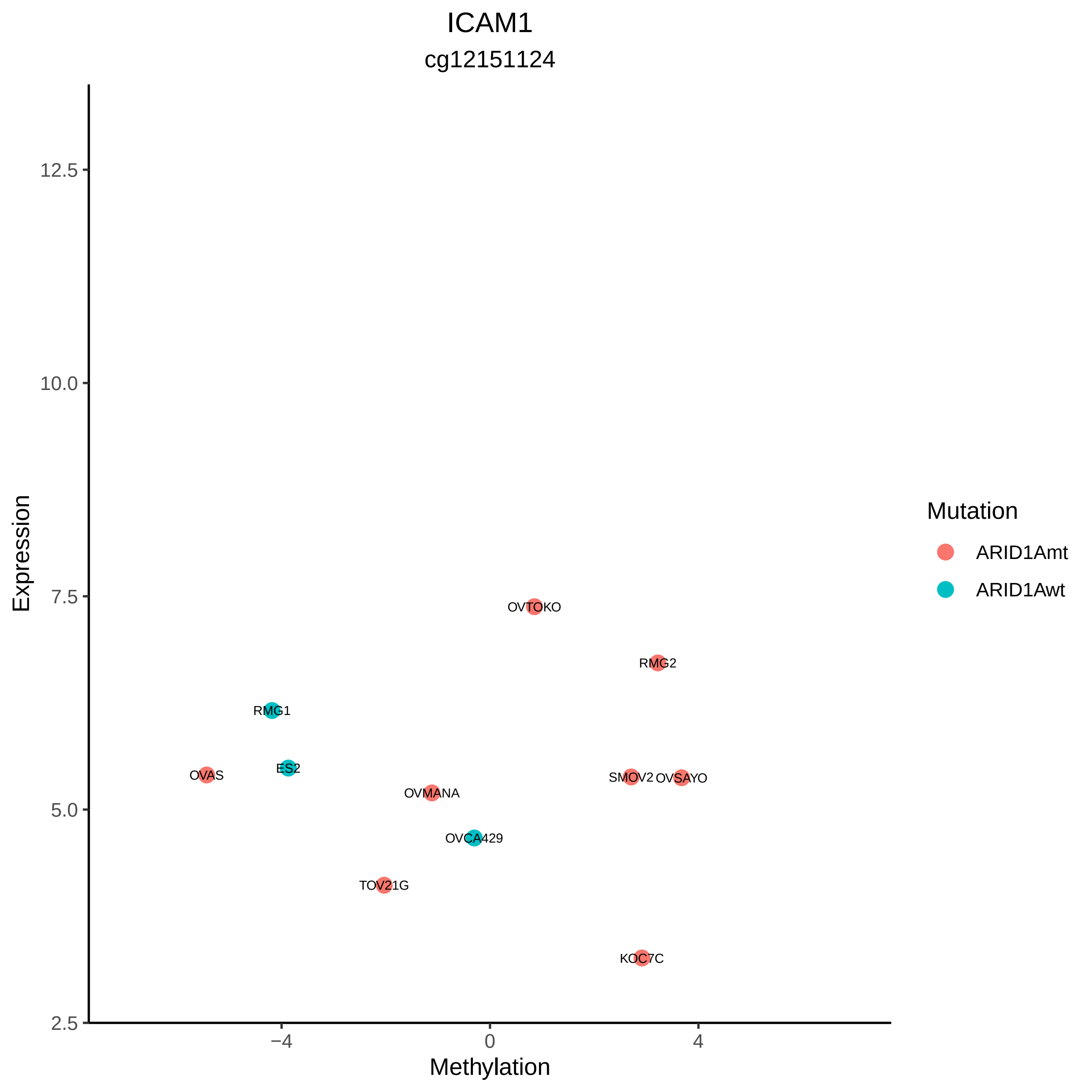

ICAM1
cg12151124
Expression
12.5
10.0
7.5
5.0
2.5
−4
0
4
Methylation
OVTOKO
RMG2
RMG1
OVAS
ES2
OVMANA
SMOV2
OVSAYO
OVCA429
TOV21G
KOC7C
Mutation
ARID1Amt
ARID1Awt

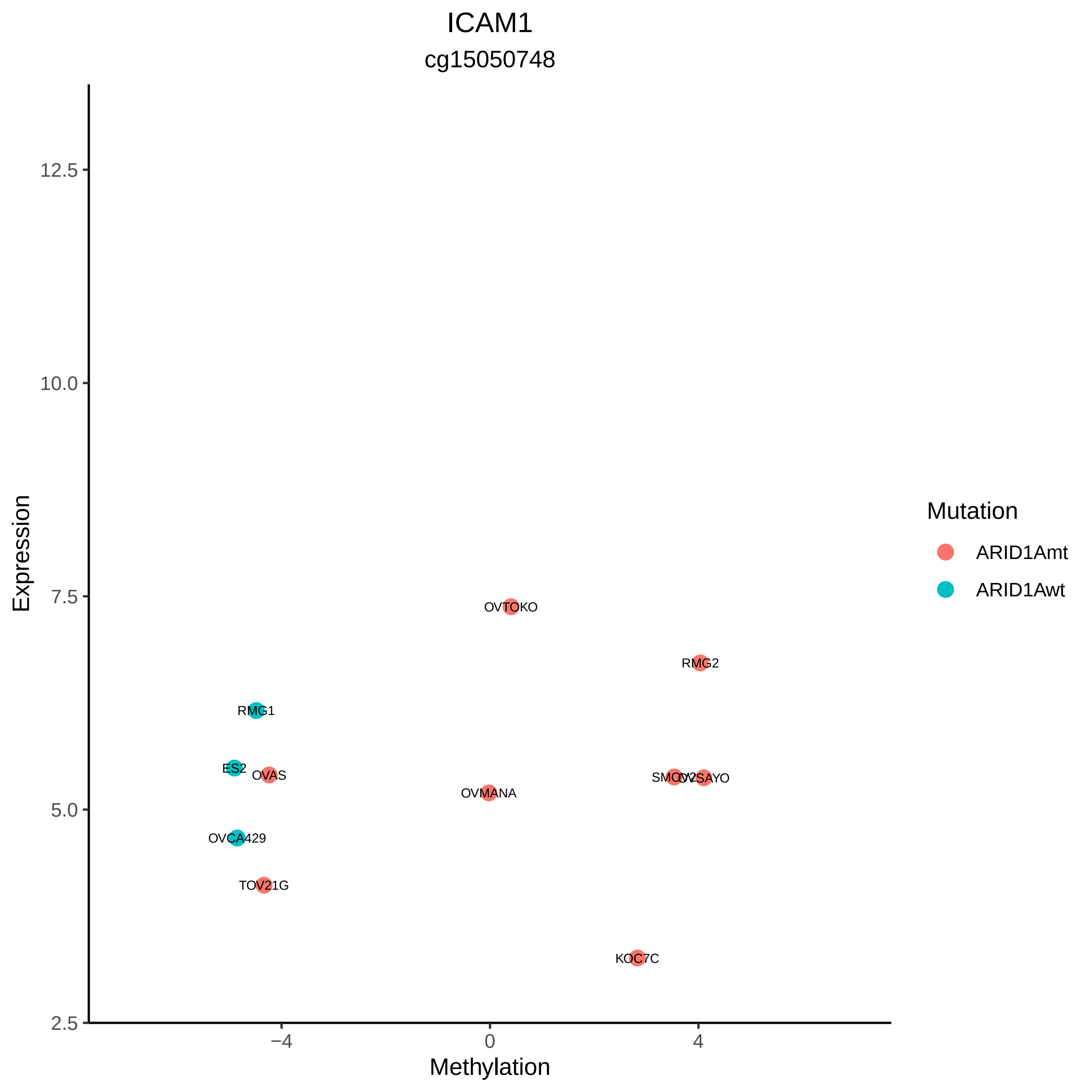

ICAM1
cg15050748
Expression
12.5
10.0
7.5
5.0
2.5
-4
0
4
Methylation
OVTOKO
RMG2
RMG1
ES2
OVAS
OVMANA
SMOV2
OVSAYO
OVCA429
TOV21G
KOC7C
Mutation
ARID1Amt
ARID1Awt

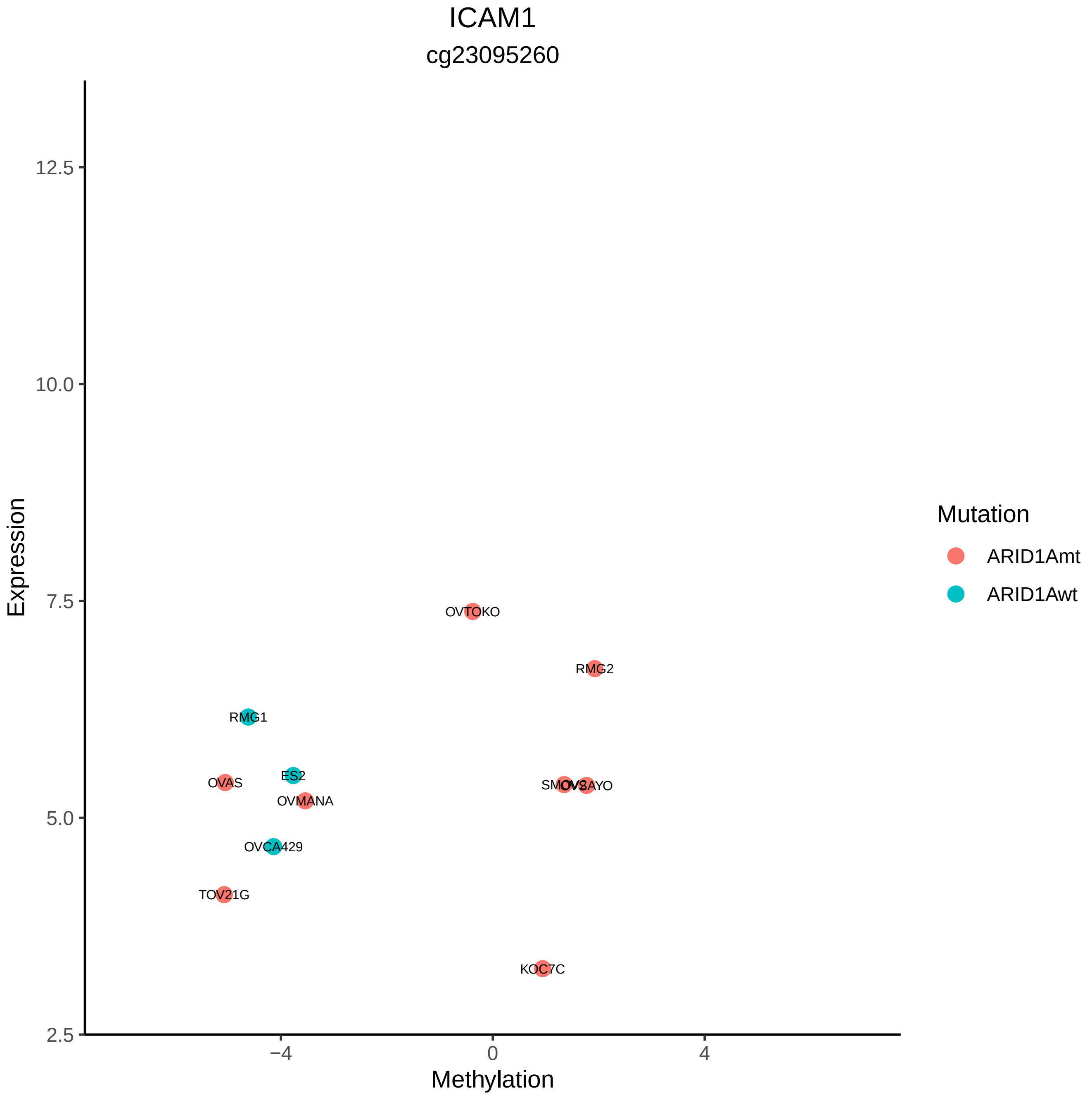
ICAM1
cg23095260
Expression
Methylation
12.5
10.0
7.5
5.0
2.5
−4
0
4
OVTOKO
RMG2
RMG1
ES2
OVAS
OVMANA
SMOV2
OVSAYO
OVCA429
TOV21G
KOC7C
Mutation
ARID1Amt
ARID1Awt

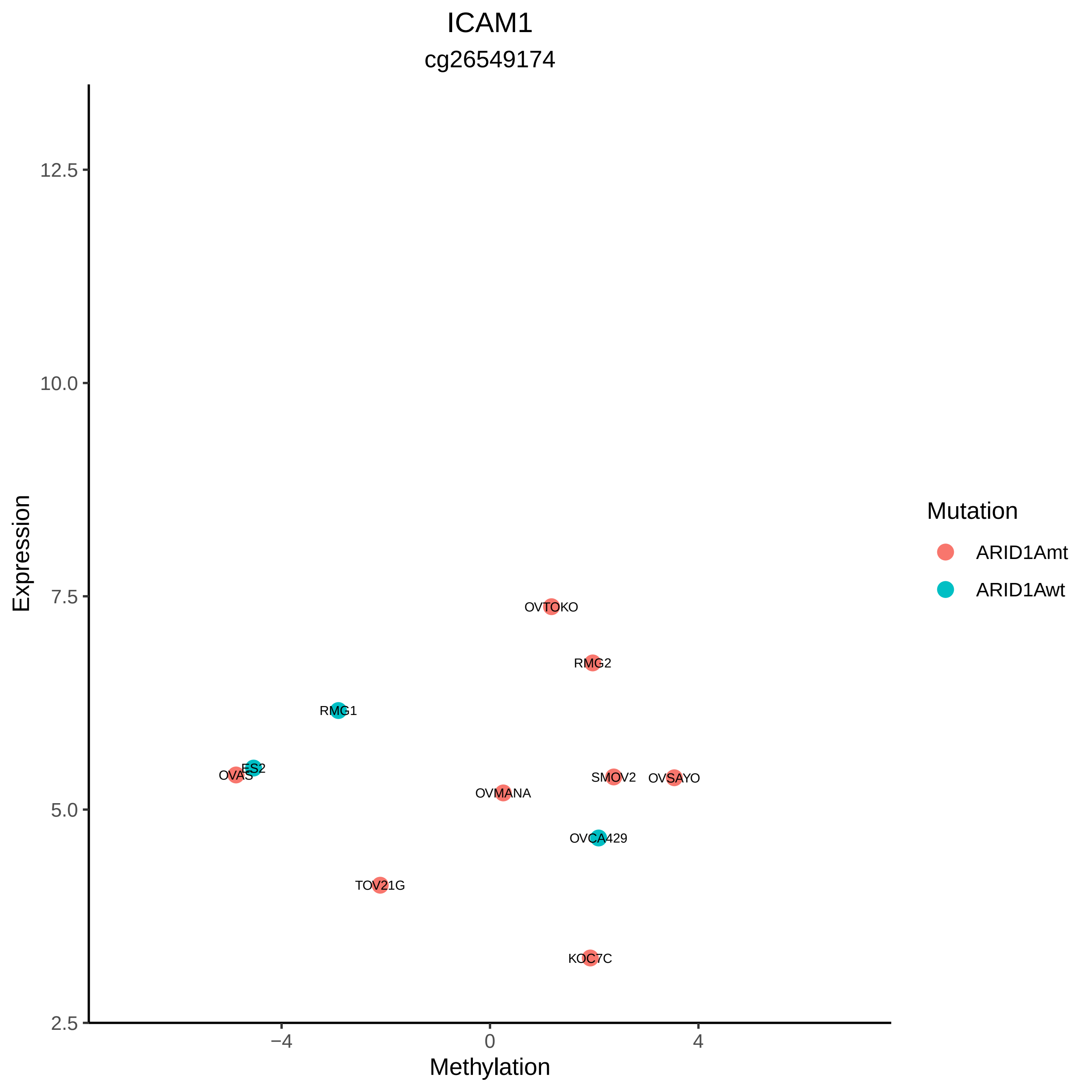

ICAM1
cg26549174
Expression
Methylation
12.5
10.0
7.5
5.0
2.5
−4
0
4
OVTOKO
RMG2
RMG1
ES2
OVAS
SMOV2
OVSAYO
OVMANA
OVCA429
TOV21G
KOC7C
Mutation
ARID1Amt
ARID1Awt

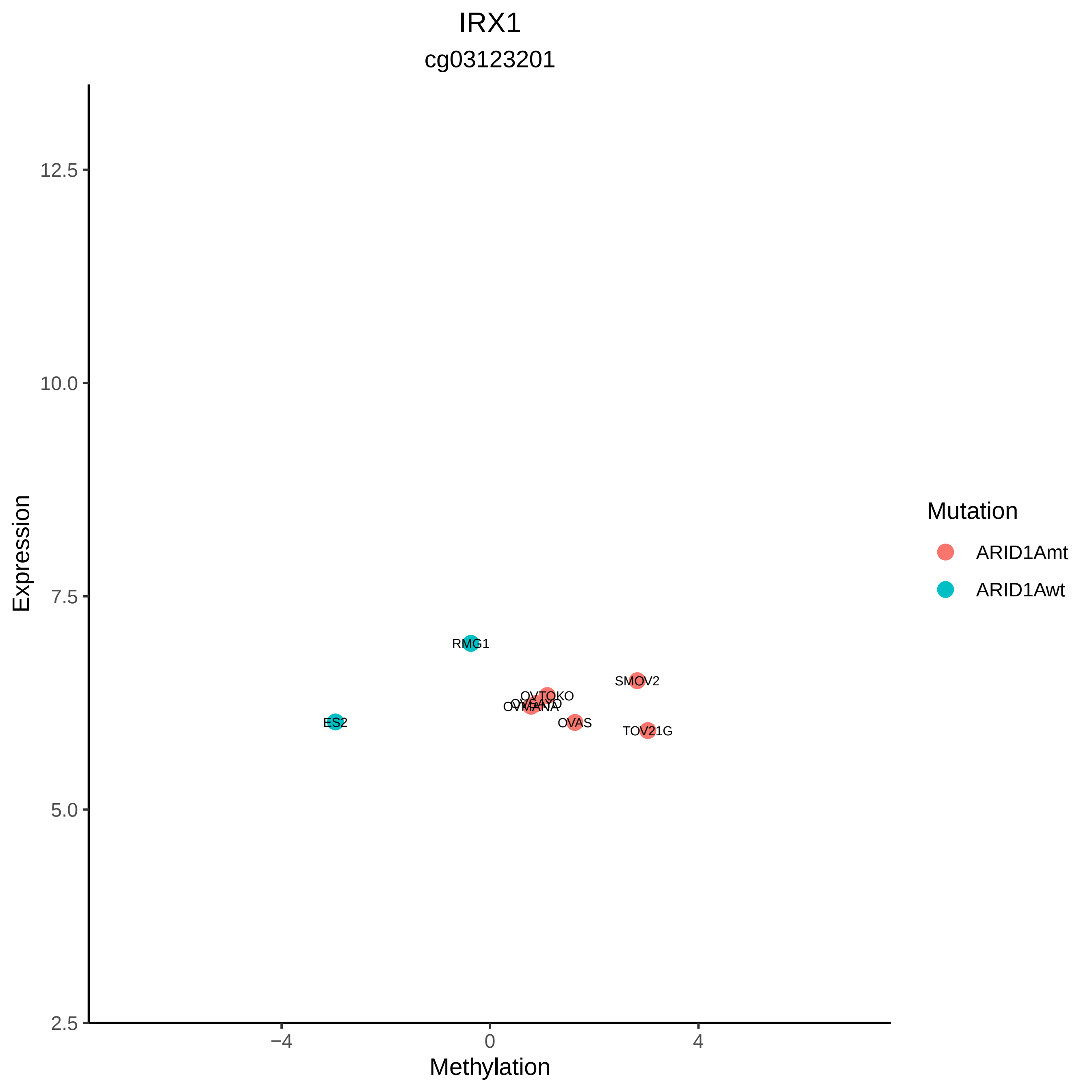

IRX1
cg03123201
Expression
Methylation
Mutation
ARID1Amt
ARID1Awt
RMG1
SMOV2
OVTOKO
OVMANA
ES2
OVAS
TOV21G
12.5
10.0
7.5
5.0
2.5
−4
0
4

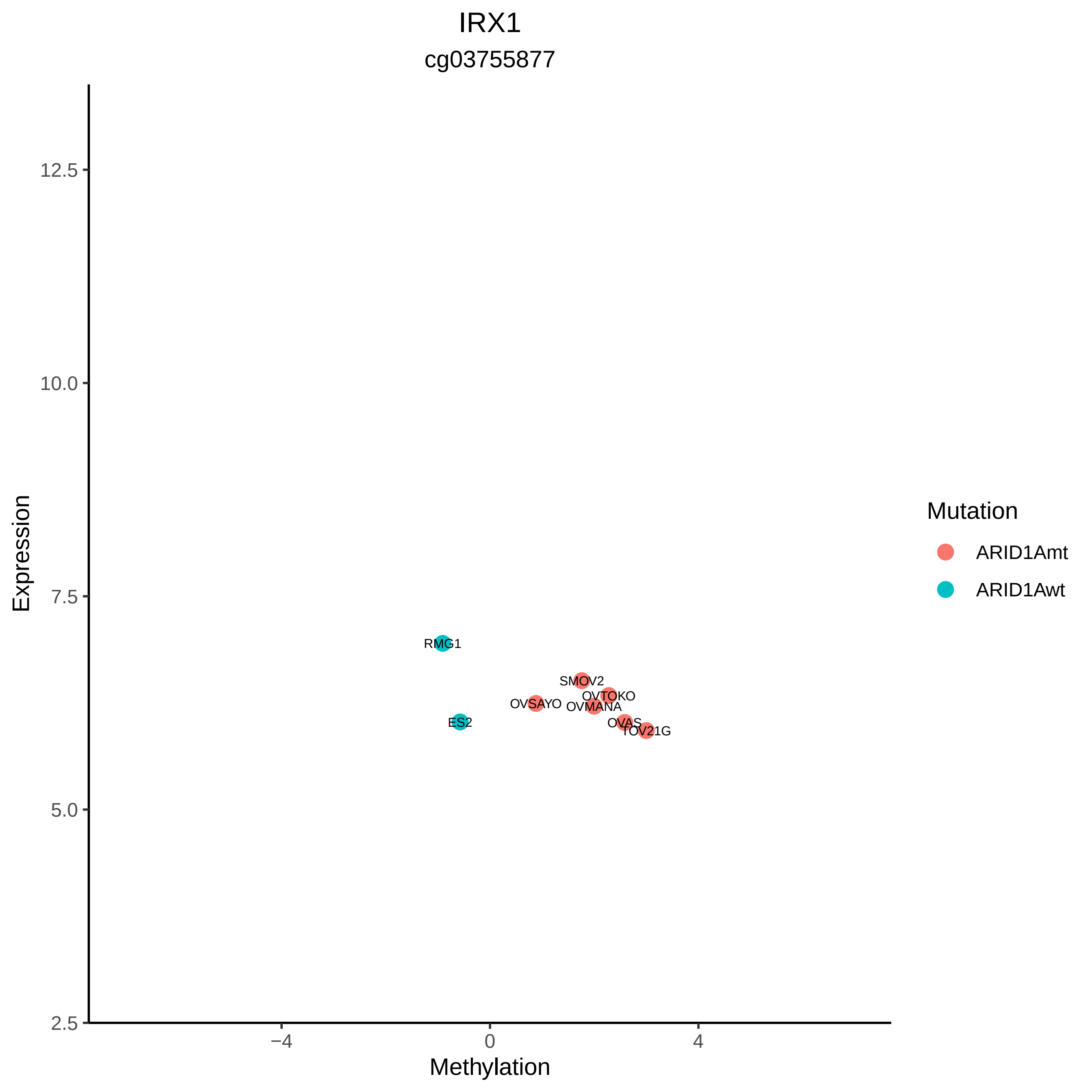

IRX1
cg03755877
Expression
Methylation
12.5
10.0
7.5
5.0
2.5
−4
0
4
RMG1
SMOV2
OVTOKO
OVSAYO
OVMANA
ES2
OVAS
TOV21G
Mutation
ARID1Amt
ARID1Awt

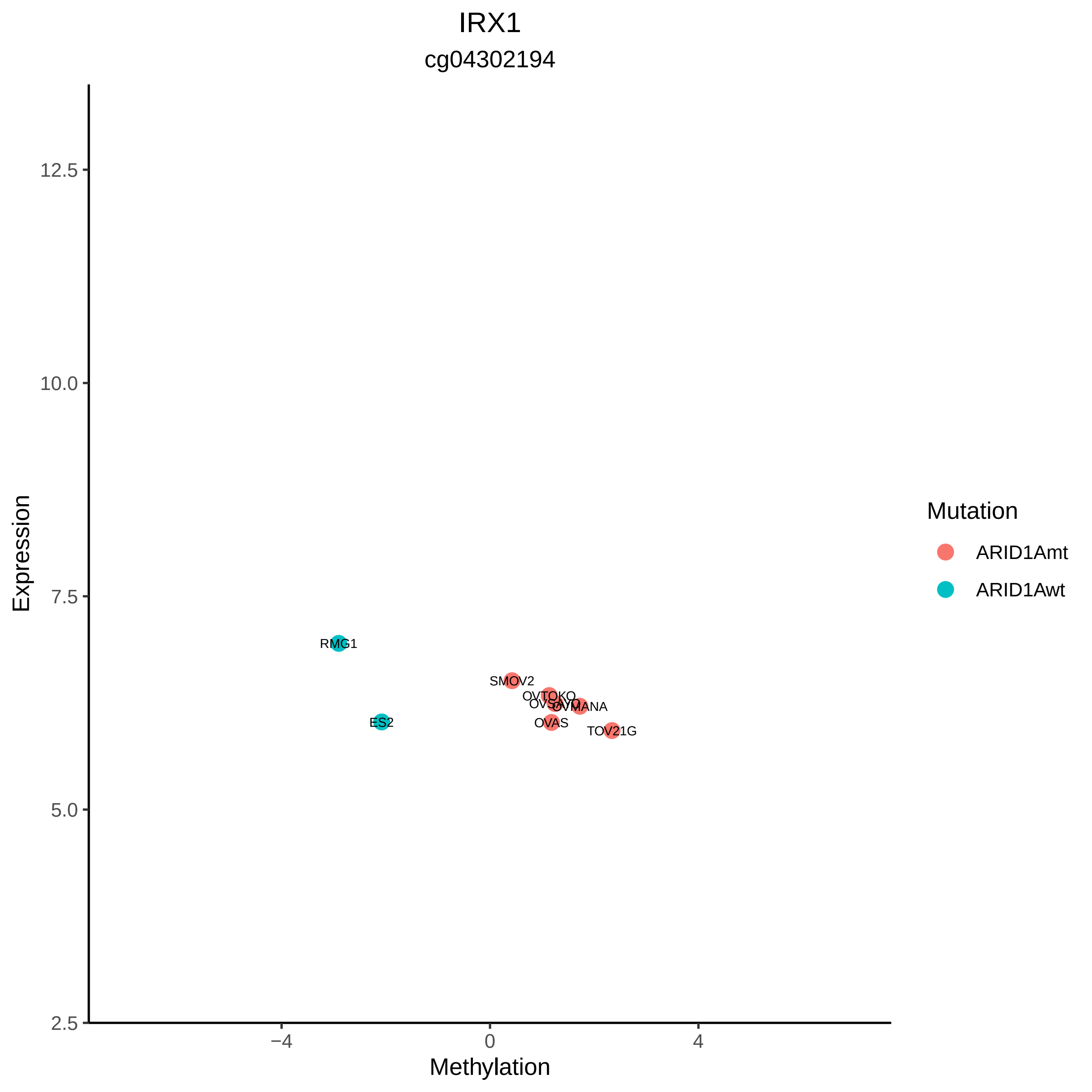
IRX1
cg04302194
Expression
12.5
10.0
7.5
5.0
2.5
−4
0
4
Methylation
RMG1
ES2
SMOV2
OVTOKO
OVSAYO
OVMANA
OVAS
TOV21G
Mutation
ARID1Amt
ARID1Awt

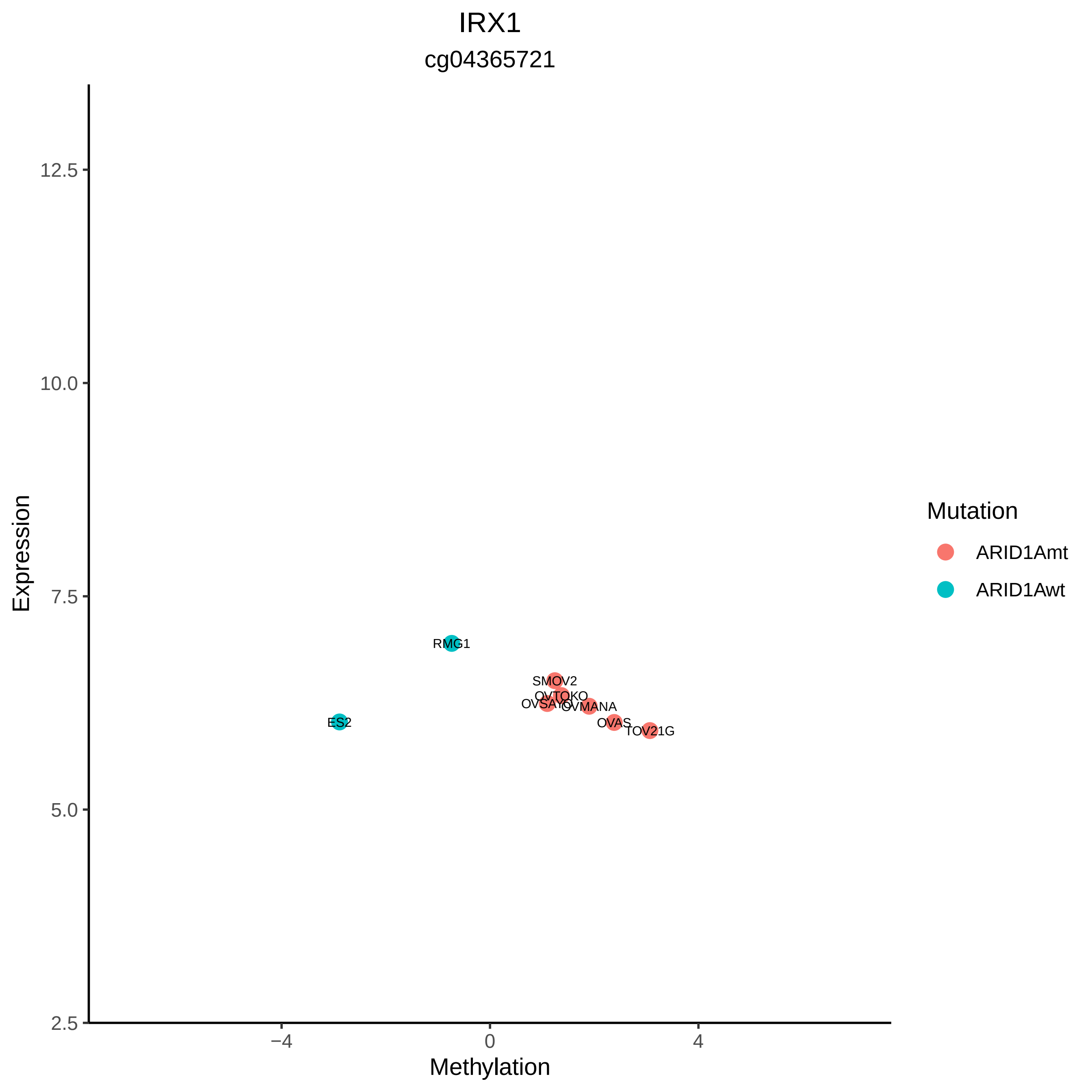

IRX1
cg04365721
Expression
12.5
10.0
7.5
5.0
2.5
Methylation
−4
0
4
RMG1
SMOV2
OVTOKO
OVSAYO
OVMANA
OVAS
TOV21G
ES2
Mutation
ARID1Amt
ARID1Awt

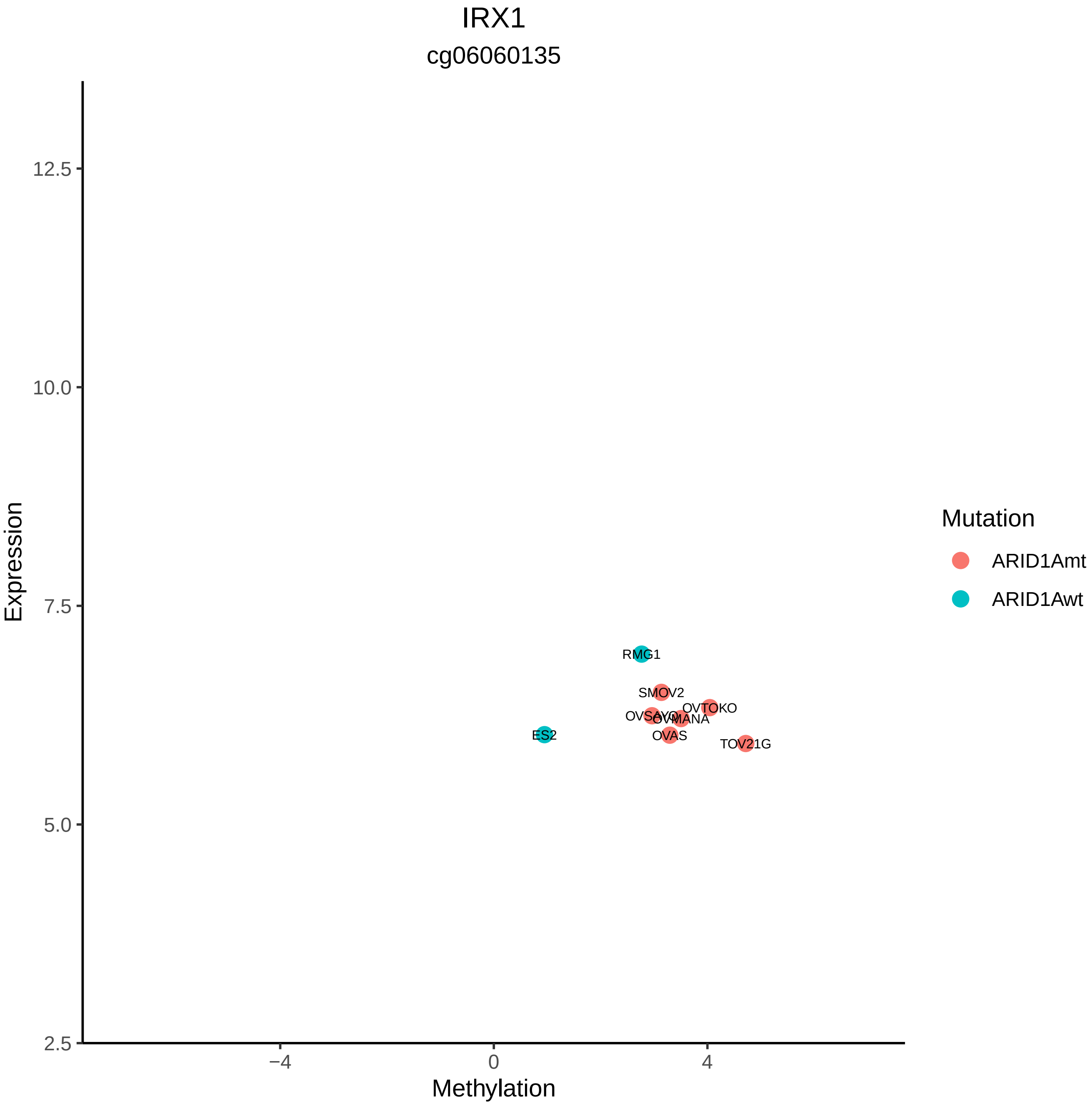
IRX1
cg06060135
Expression
Methylation
12.5
10.0
7.5
5.0
2.5
−4
0
4
RMG1
SMOV2
OVTOKO
OVSAYO
OVMANA
ES2
OVAS
TOV21G
Mutation
ARID1Amt
ARID1Awt

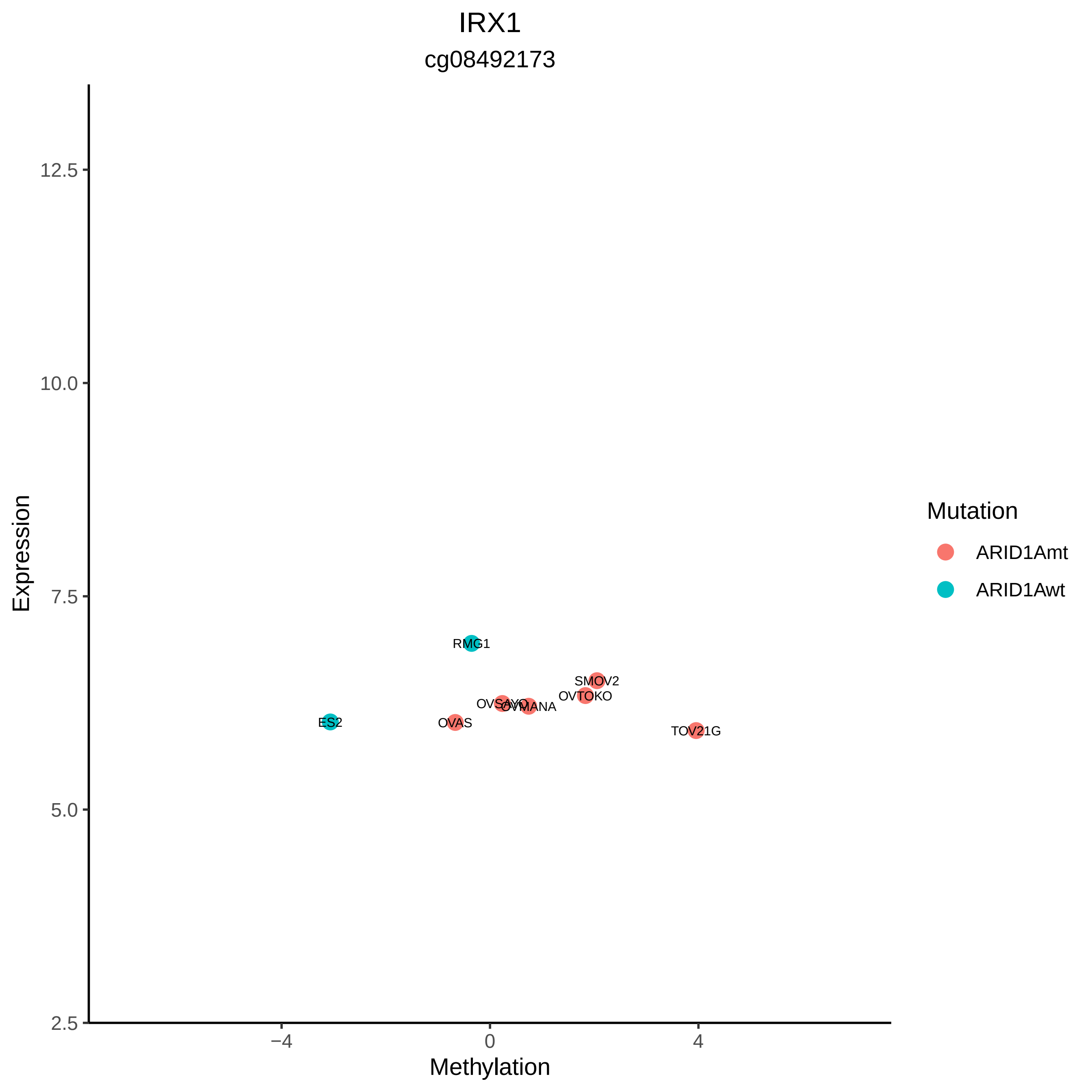

IRX1
cg08492173
Expression
12.5
10.0
7.5
5.0
2.5
−4
0
4
Methylation
RMG1
SMOV2
OVTOKO
OVSAYO
OVMANA
ES2
OVAS
TOV21G
Mutation
ARID1Amt
ARID1Awt

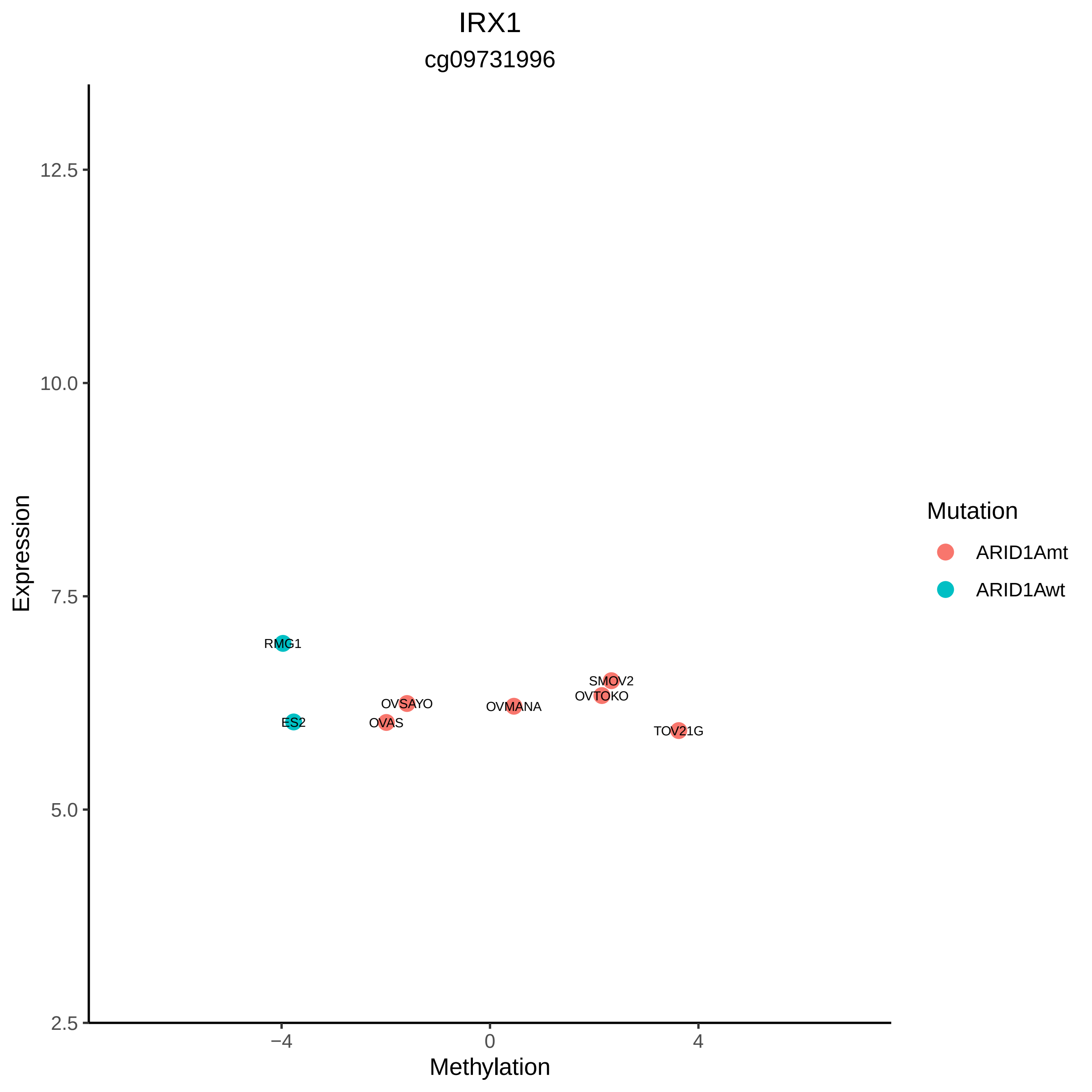
IRX1
cg09731996
Expression
12.5
10.0
7.5
5.0
2.5
−4
0
4
Methylation
RMG1
ES2
OVSAYO
OVAS
OVMANA
SMOV2
OVTOKO
TOV21G
Mutation
ARID1Amt
ARID1Awt

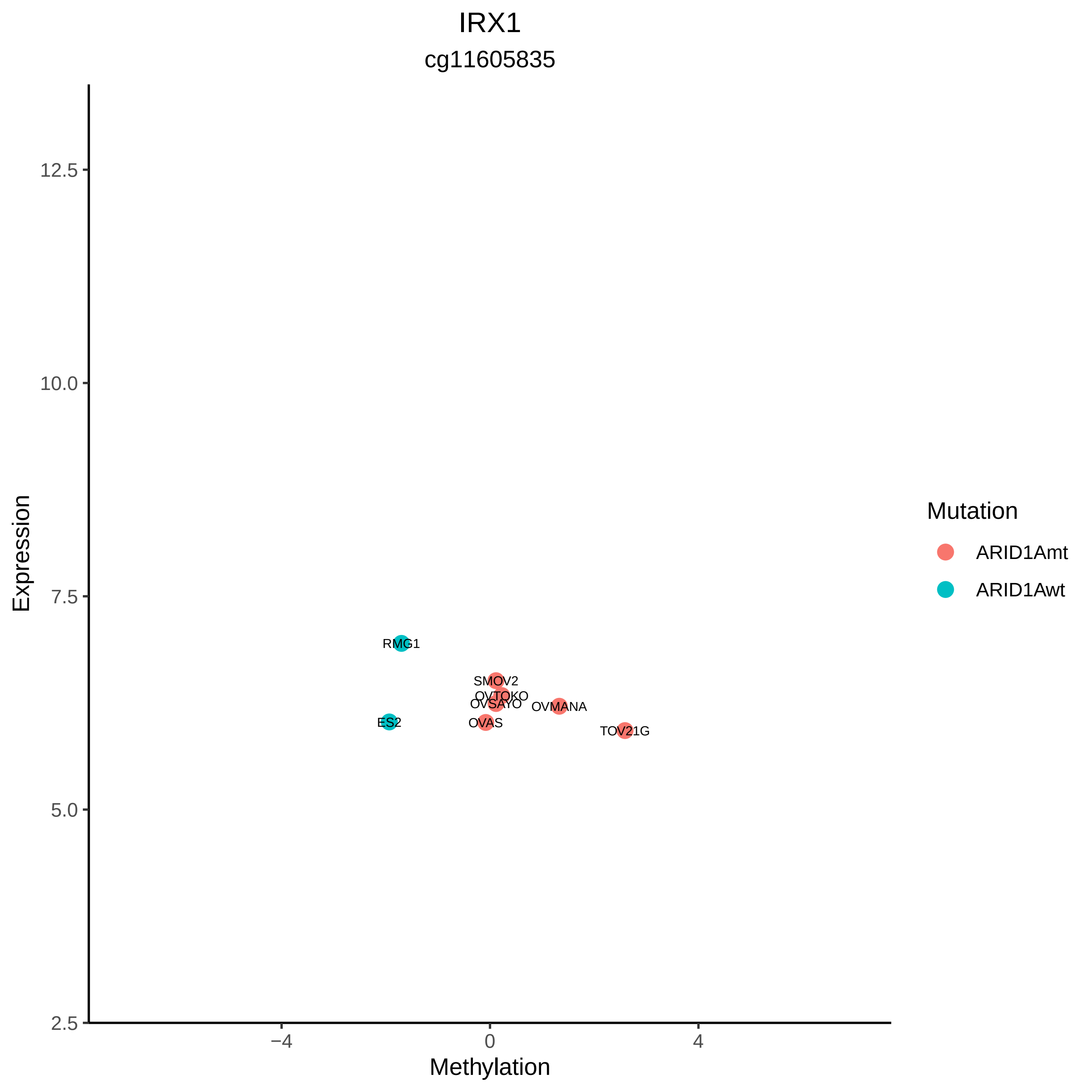

IRX1
cg11605835
Expression
12.5
10.0
7.5
5.0
2.5
−4
0
4
Methylation
RMG1
SMOV2
OVTOKO
OVSAYO
OVMANA
ES2
OVAS
TOV21G
Mutation
ARID1Amt
ARID1Awt

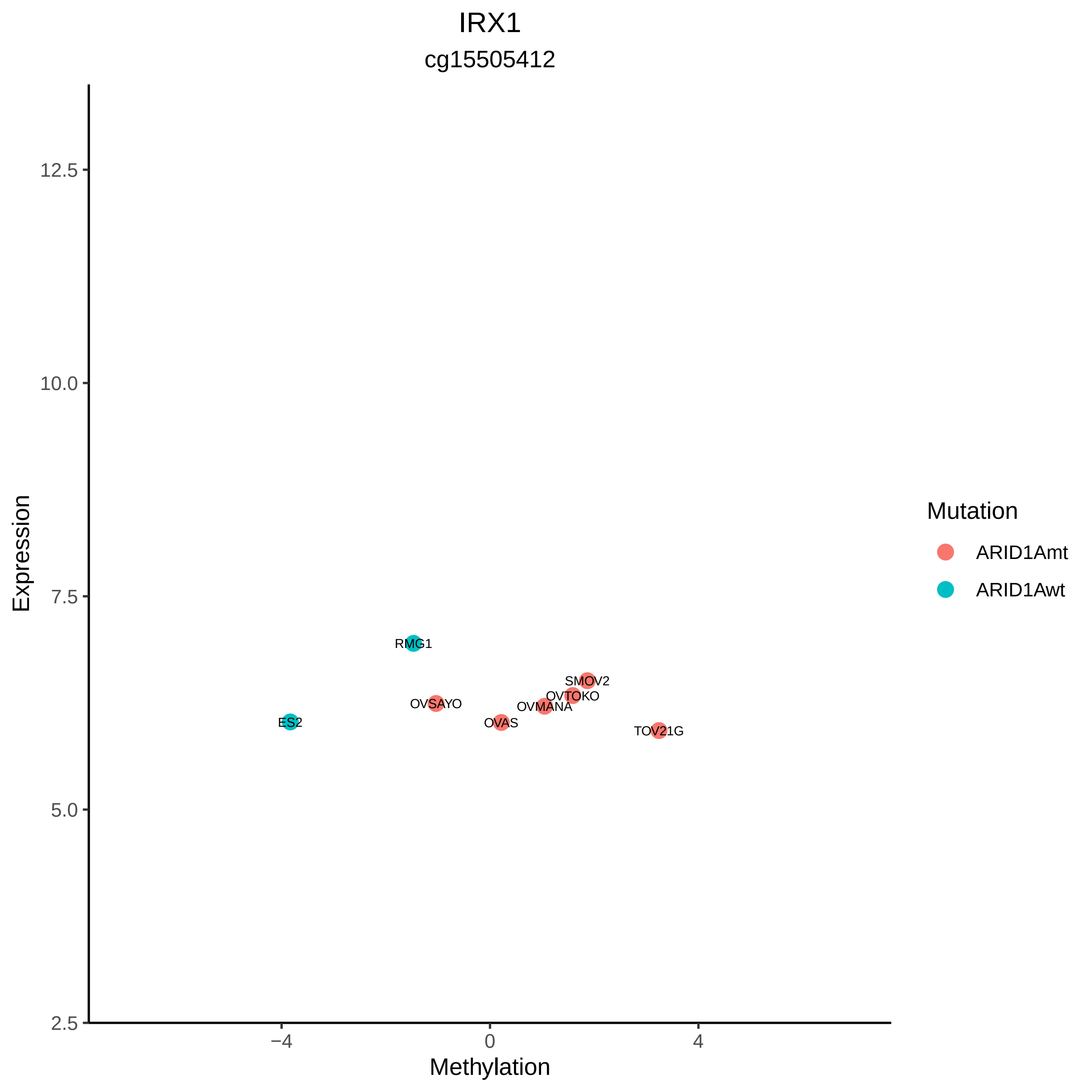
IRX1
cg15505412
Expression
12.5
10.0
7.5
5.0
2.5
−4
0
4
Methylation
RMG1
SMOV2
OVTOKO
OVSAYO
OVMANA
ES2
OVAS
TOV21G
Mutation
ARID1Amt
ARID1Awt

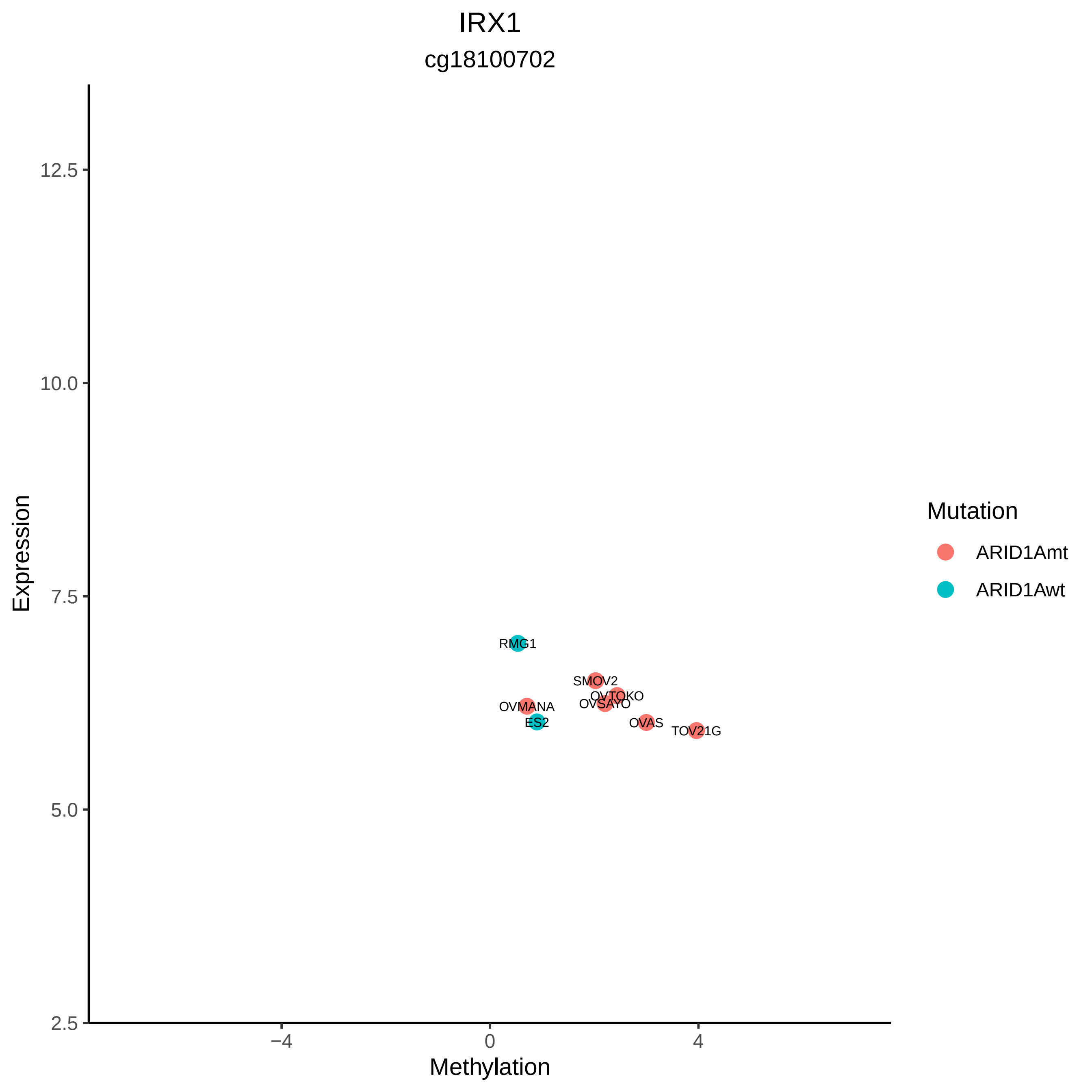

IRX1
cg18100702
Expression
Methylation
12.5
10.0
7.5
5.0
2.5
−4
0
4
RMG1
SMOV2
OVTOKO
OVSAYO
OVMANA
ES2
OVAS
TOV21G
Mutation
ARID1Amt
ARID1Awt

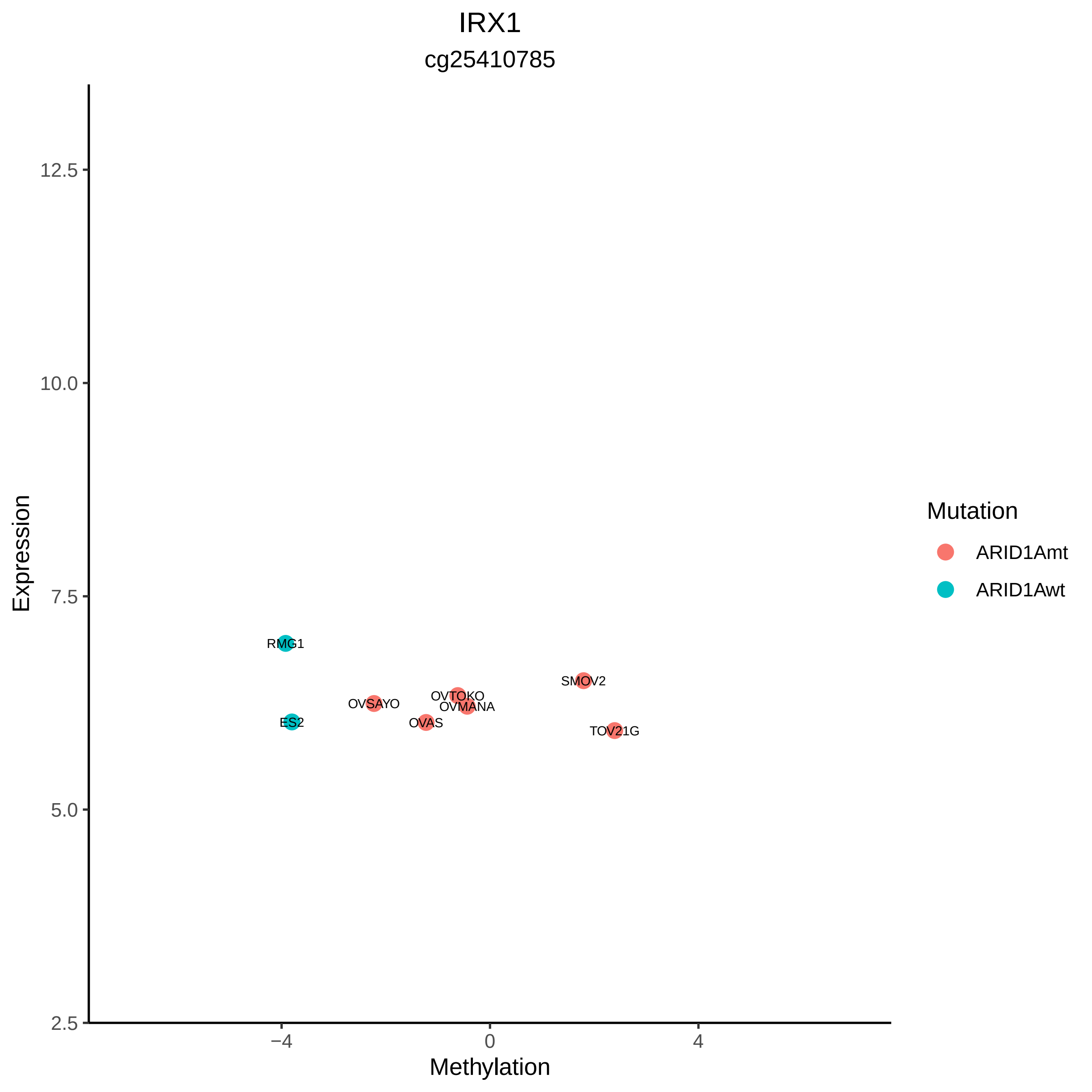
IRX1
cg25410785
Expression
12.5
10.0
7.5
5.0
2.5
−4
0
4
Methylation
RMG1
ES2
OVSAYO
OVAS
OVTOKO
OVMANA
SMOV2
TOV21G
Mutation
ARID1Amt
ARID1Awt

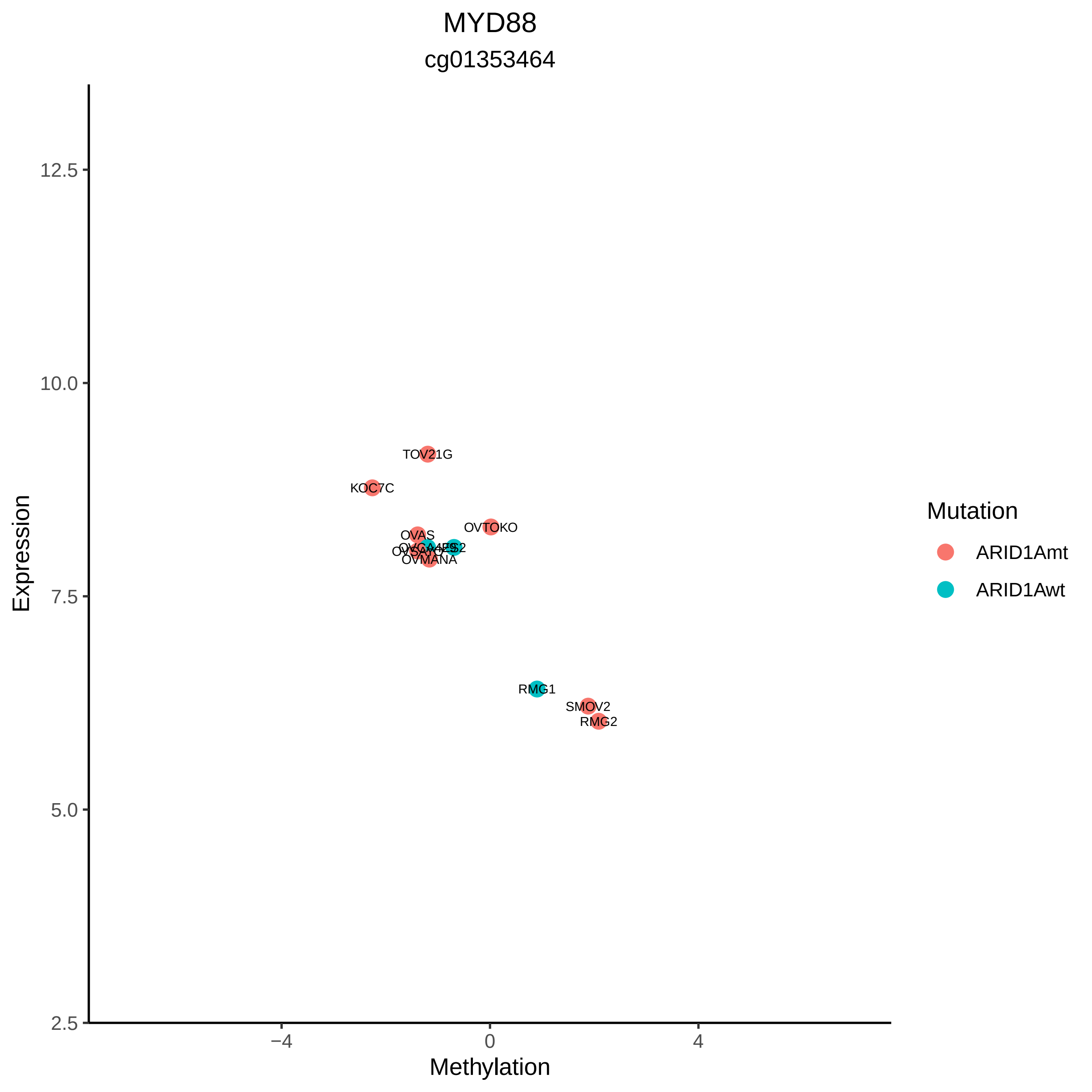

MYD88
cg01353464
Expression
Methylation
12.5
10.0
7.5
5.0
2.5
−4
0
4
TOV21G
KOC7C
OVTOKO
OVAS
OVISE
OVCA429
OVSAYO
ES2
OVMANA
RMG1
SMOV2
RMG2
Mutation
ARID1Amt
ARID1Awt

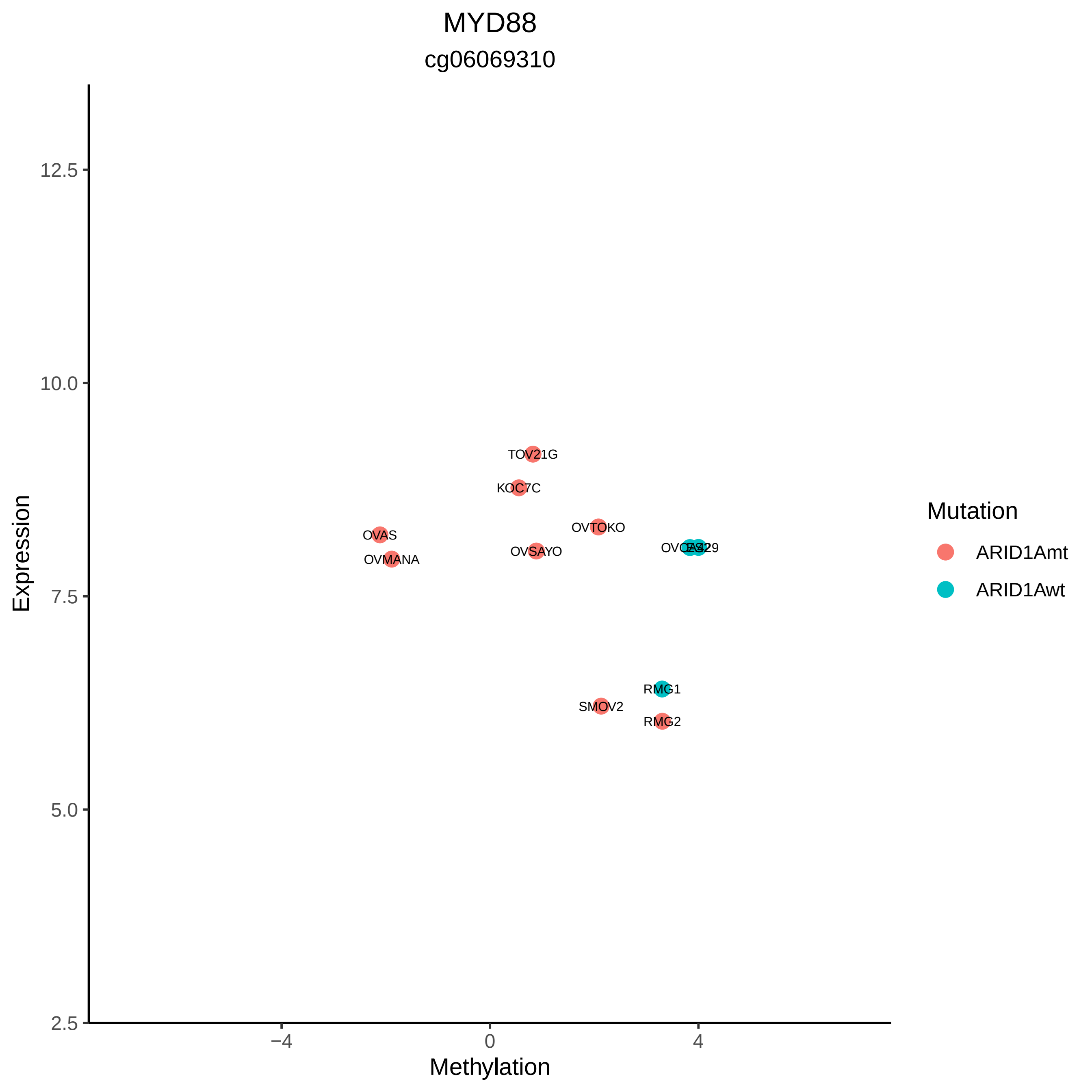

MYD88
cg06069310
Expression
Methylation
12.5
10.0
7.5
5.0
2.5
−4
0
4
TOV21G
KOC7C
OVTOKO
OVAS
OVSAYO
OVMANA
RMG1
SMOV2
RMG2
Mutation
ARID1Amt
ARID1Awt

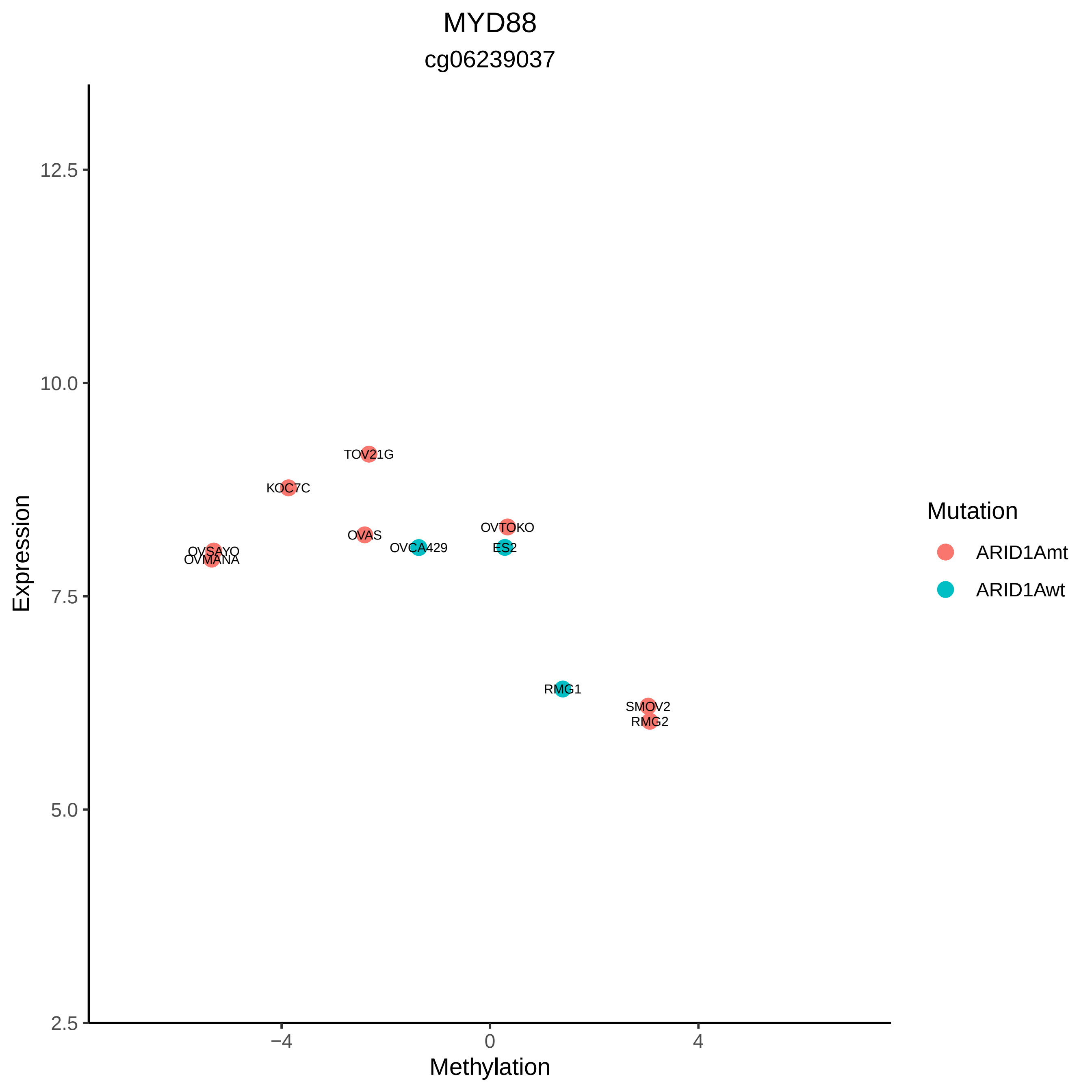
MYD88
cg06239037
Expression
Methylation
12.5
10.0
7.5
5.0
2.5
−4
0
4
TOV21G
KOC7C
OVTOKO
OVAS
OVCA429
ES2
OVSAYO
OVMANA
RMG1
SMOV2
RMG2
Mutation
ARID1Amt
ARID1Awt

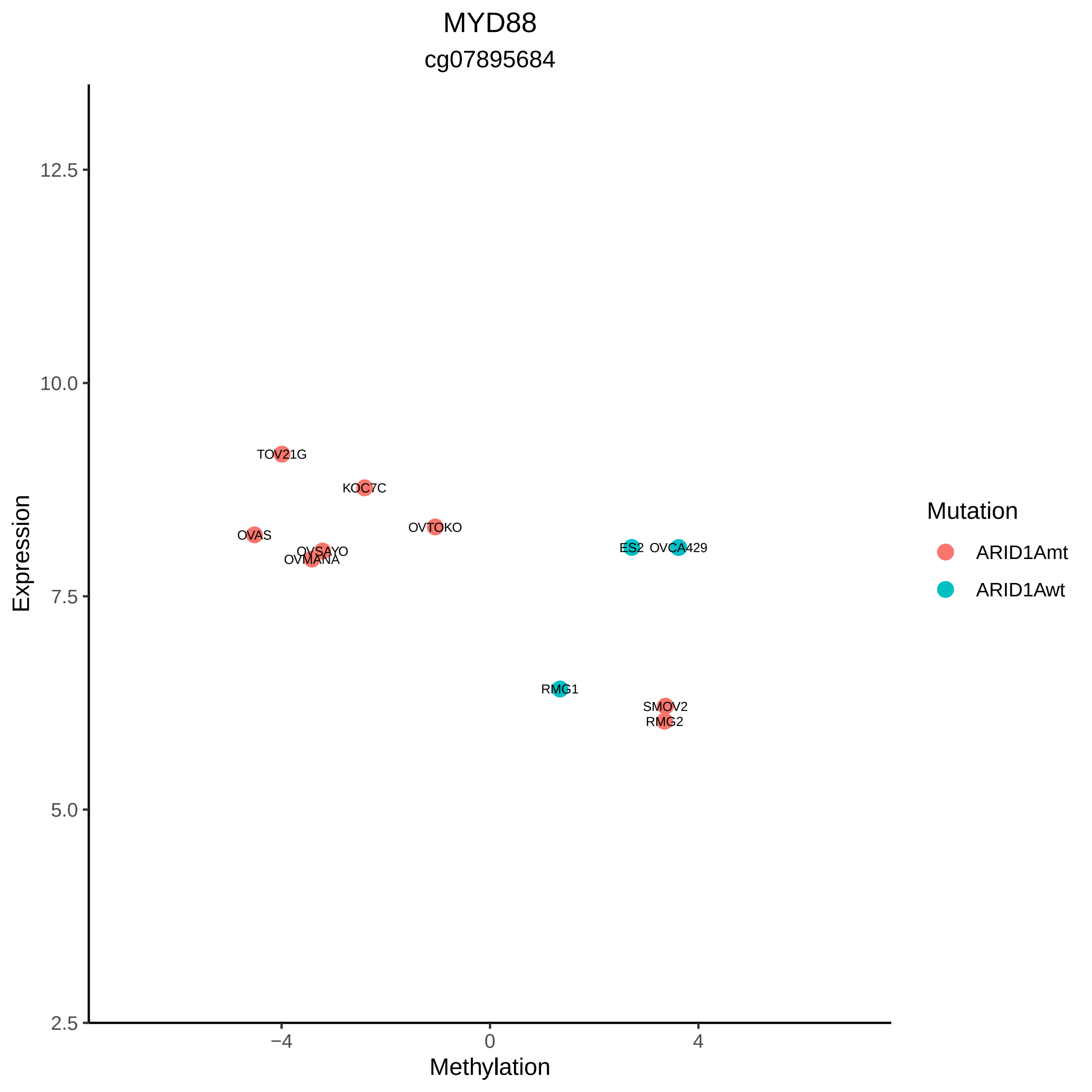
MYD88
cg07895684
Expression
Methylation
12.5
10.0
7.5
5.0
2.5
−4
0
4
TOV21G
KOC7C
OVTOKO
OVAS
OVSAYO
OVMANA
ES2
OVCA429
RMG1
SMOV2
RMG2
Mutation
ARID1Amt
ARID1Awt

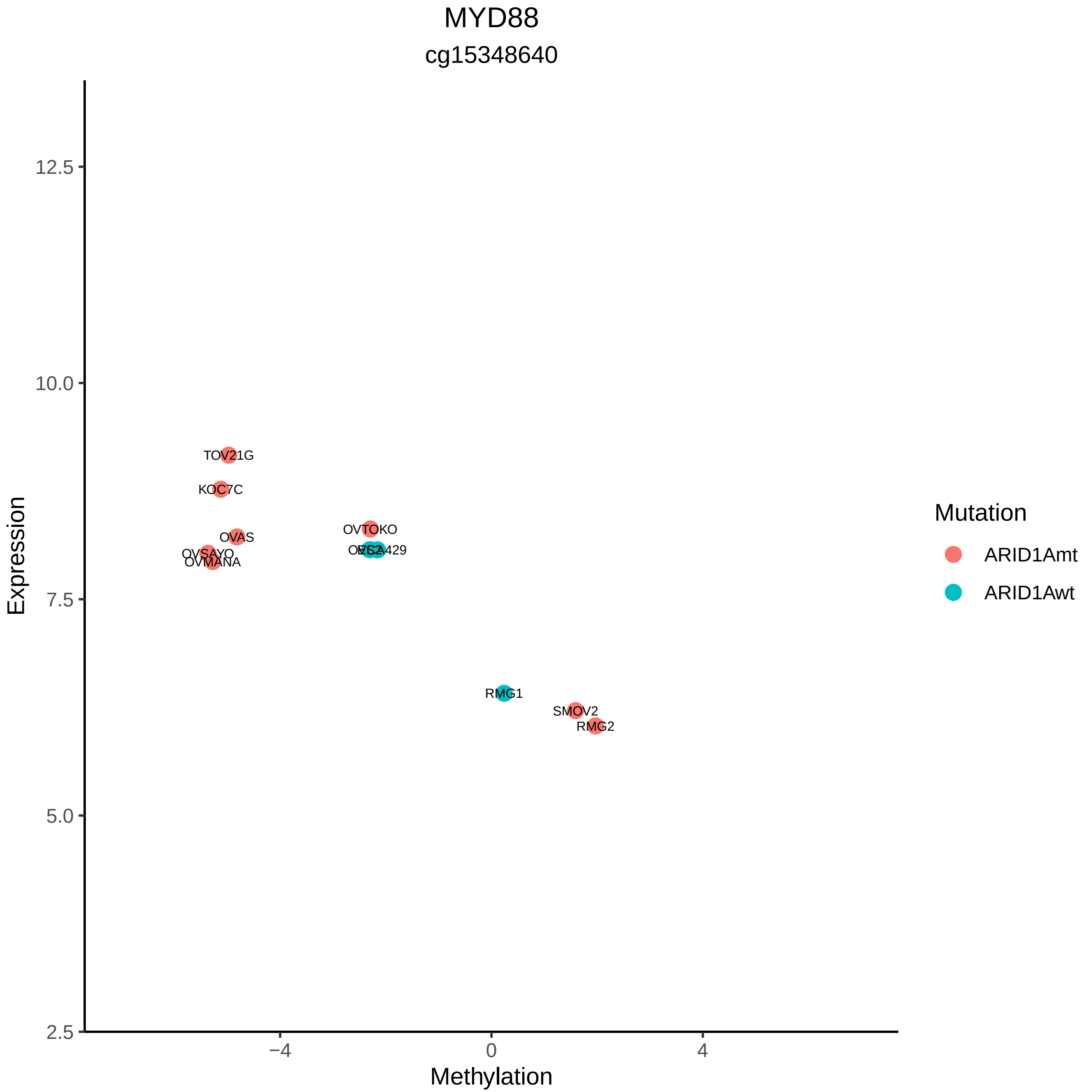
MYD88
cg15348640
Expression
Methylation
12.5
10.0
7.5
5.0
2.5
−4
0
4
TOV21G
KOC7C
OVAS
OVSAYO
OVMANA
OVTOKO
RMG1
SMOV2
RMG2
Mutation
ARID1Amt
ARID1Awt

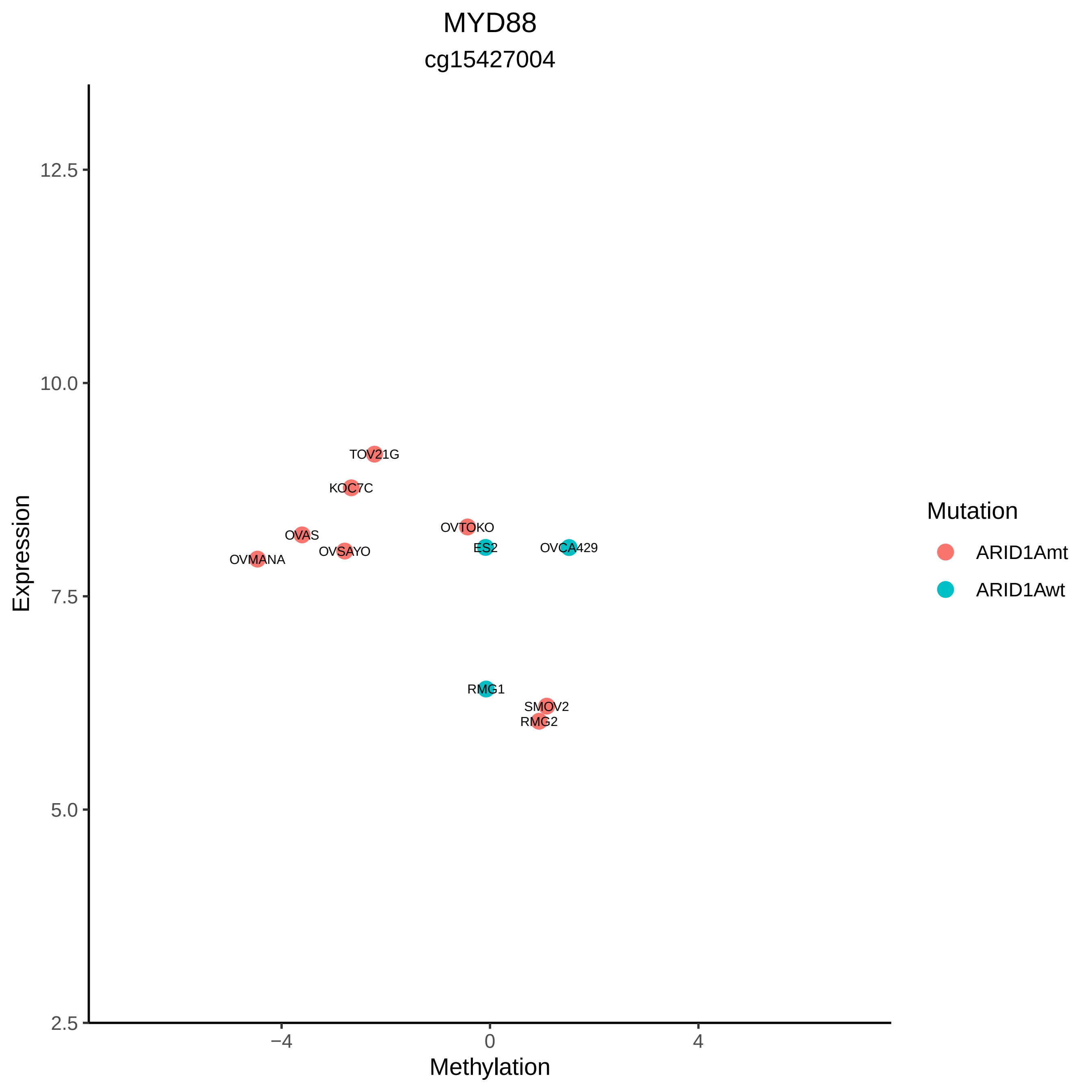
MYD88
cg15427004
Expression
12.5
10.0
7.5
5.0
2.5
Methylation
−4
0
4
TOV21G
KOC7C
OVTOKO
OVAS
OVSAYO
OVMANA
ES2
OVCA429
RMG1
SMOV2
RMG2
Mutation
ARID1Amt
ARID1Awt

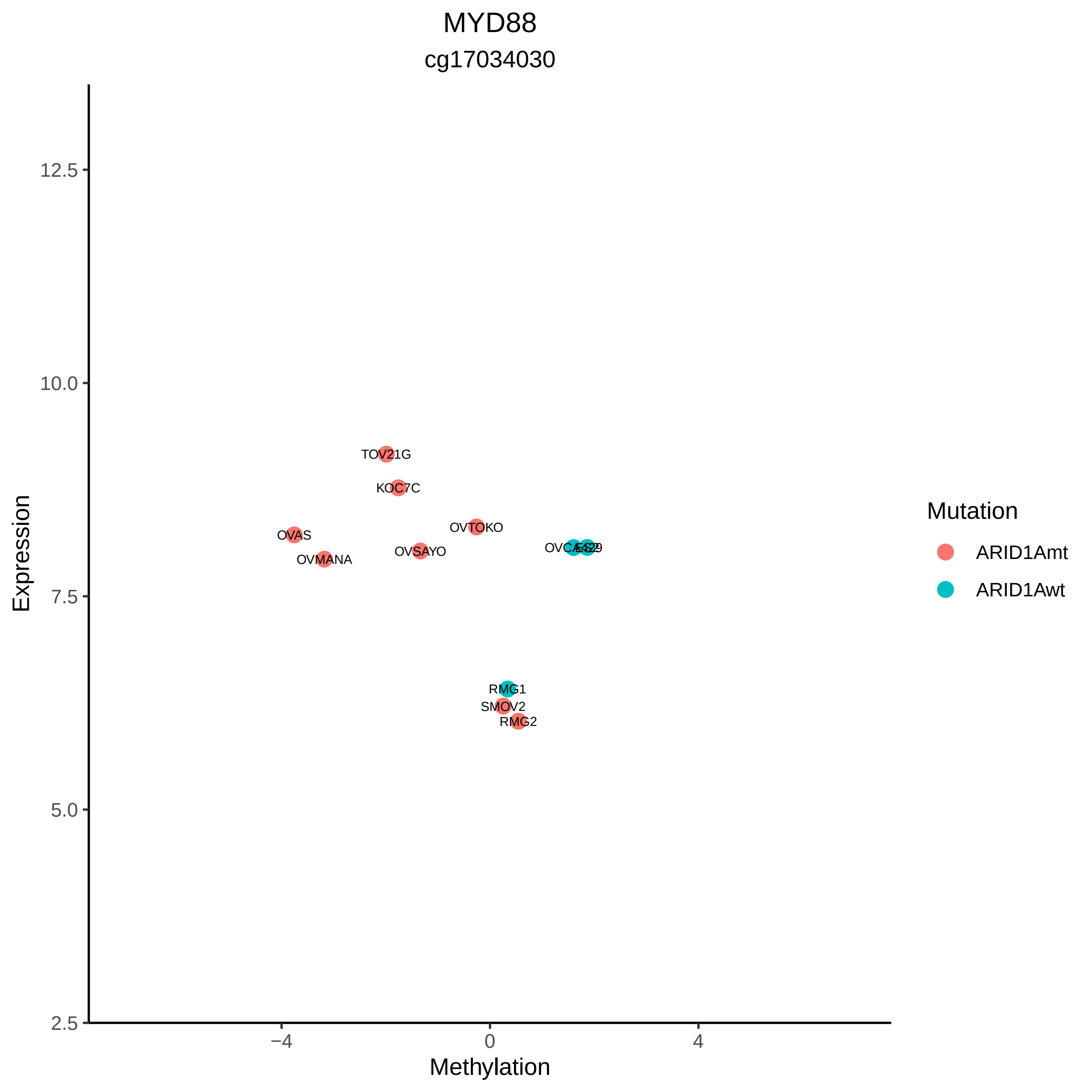
MYD88
cg17034030
Expression
12.5
10.0
7.5
5.0
2.5
−4
0
4
Methylation
TOV21G
KOC7C
OVTOKO
OVAS
OVSAYO
OVMANA
RMG1
SMOV2
RMG2
Mutation
ARID1Amt
ARID1Awt

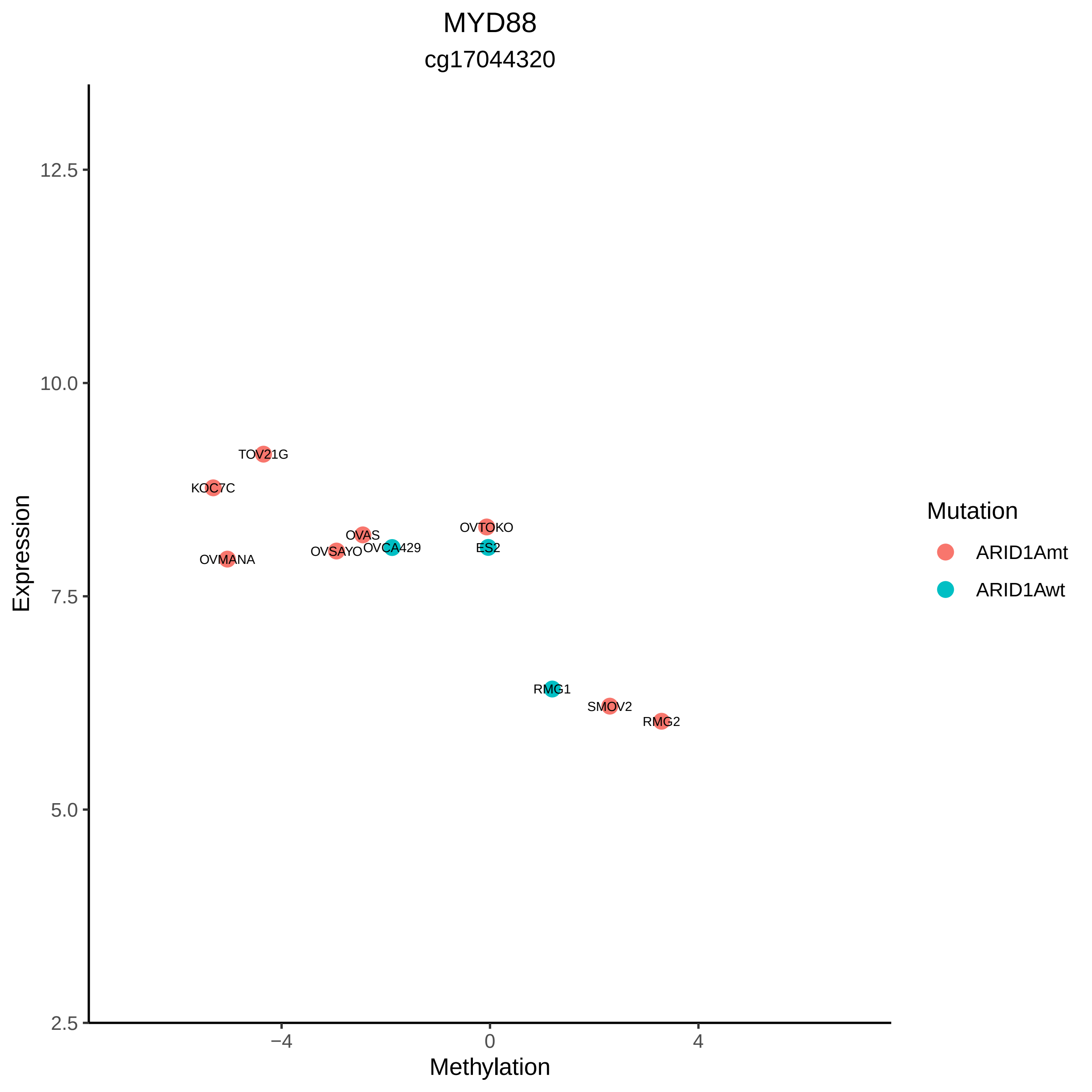
MYD88
cg17044320
Expression
Methylation
12.5
10.0
7.5
5.0
2.5
−4
0
4
TOV21G
KOC7C
OVTOKO
OVAS
OVCA429
ES2
OVSAYO
OVMANA
RMG1
SMOV2
RMG2
Mutation
ARID1Amt
ARID1Awt

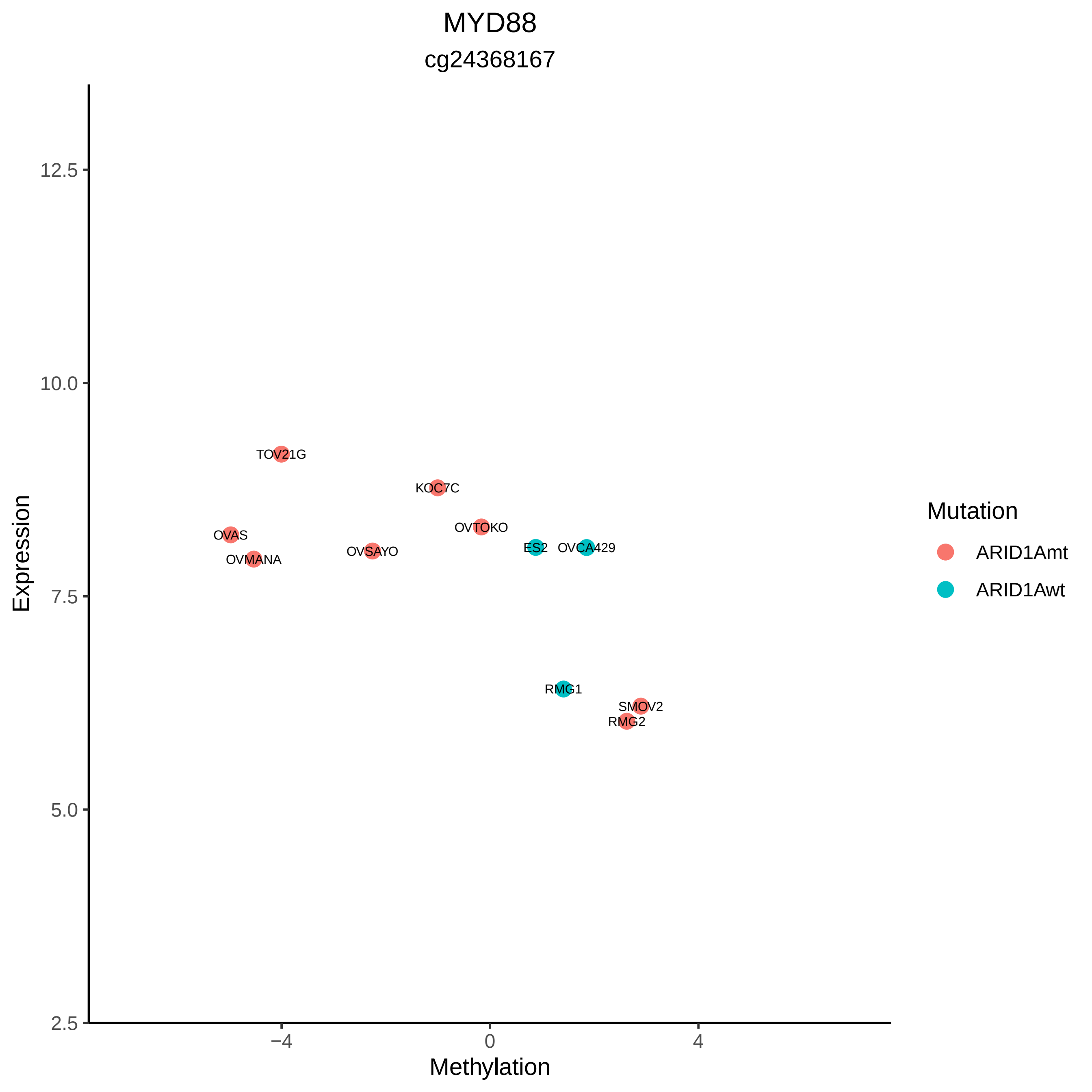

MYD88
cg24368167
12.5
10.0
7.5
5.0
2.5
Expression
−4
0
4
Methylation
TOV21G
KOC7C
OVTOKO
OVAS
OVMANA
OVSAYO
ES2
OVCA429
RMG1
SMOV2
RMG2
Mutation
ARID1Amt
ARID1Awt

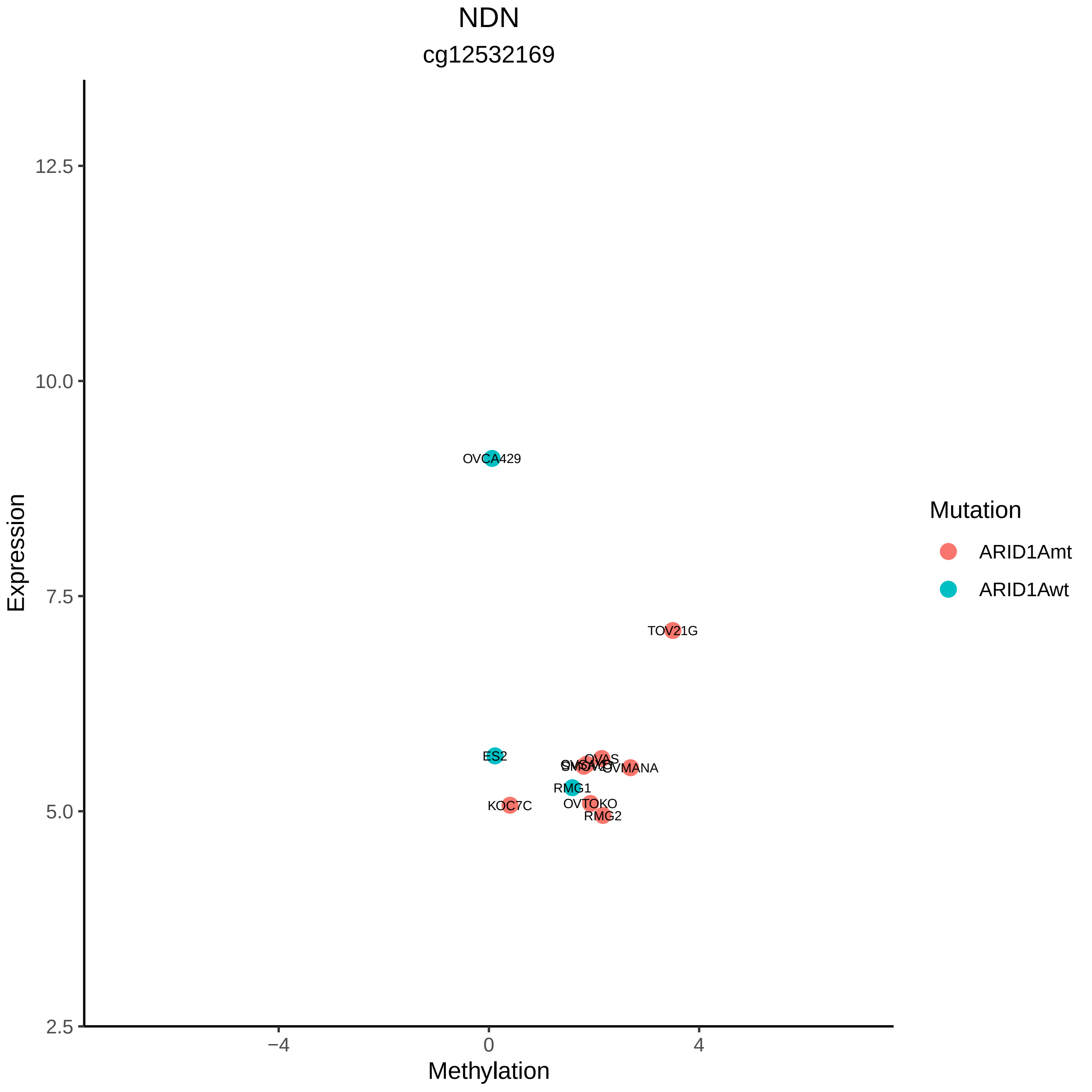
NDN
cg12532169
Expression
12.5
10.0
7.5
5.0
2.5
−4
0
4
Methylation
Mutation
ARID1Amt
ARID1Awt
OVCA429
TOV21G
ES2
OVAS
OVMANA
RMG1
KOC7C
OVTOKO
RMG2

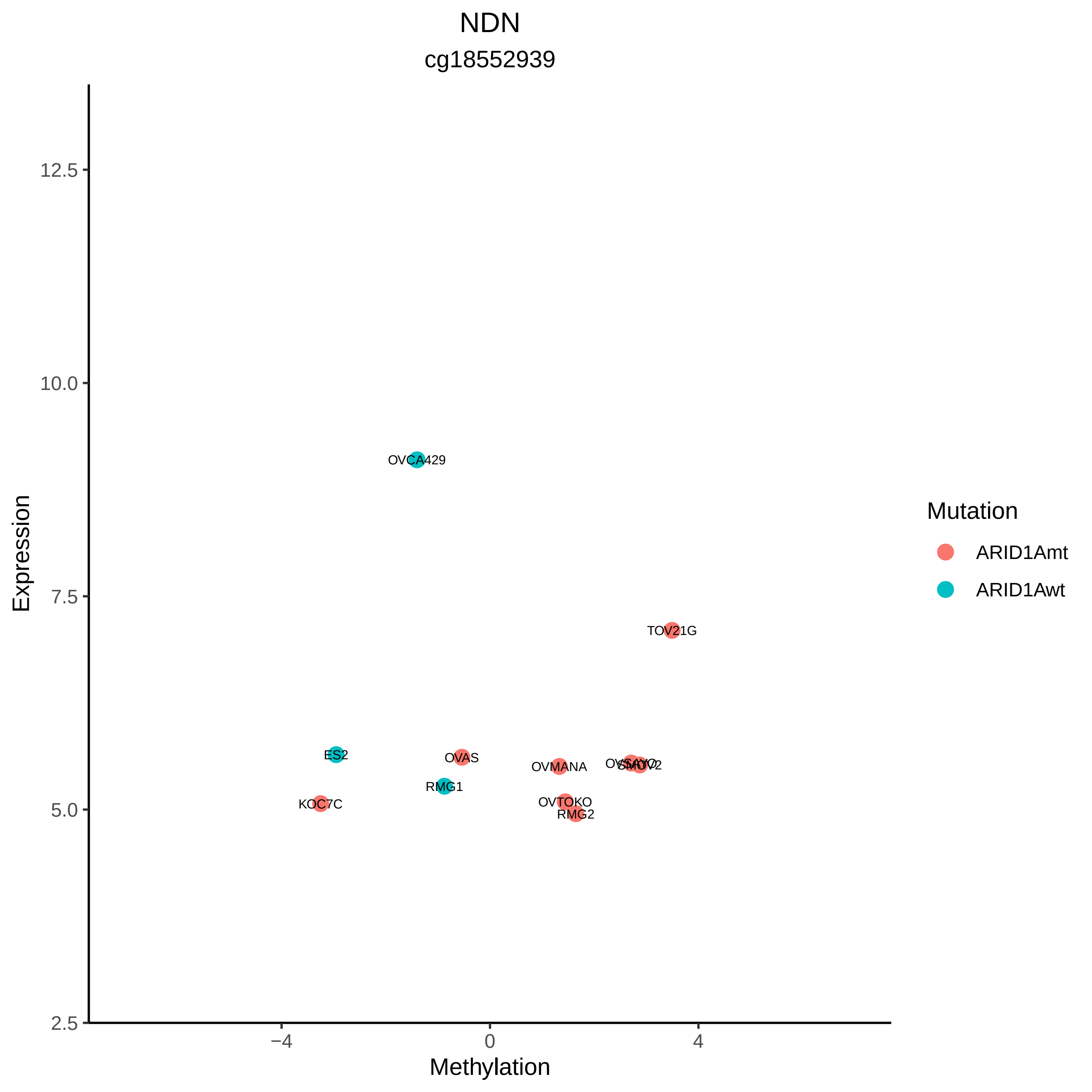

NDN
cg18552939
Expression
Methylation
12.5
10.0
7.5
5.0
2.5
−4
0
4
OVCA429
TOV21G
ES2
OVAS
OVMANA
OVSAYO
SMOV2
RMG1
KOC7C
OVTOKO
RMG2
Mutation
ARID1Amt
ARID1Awt

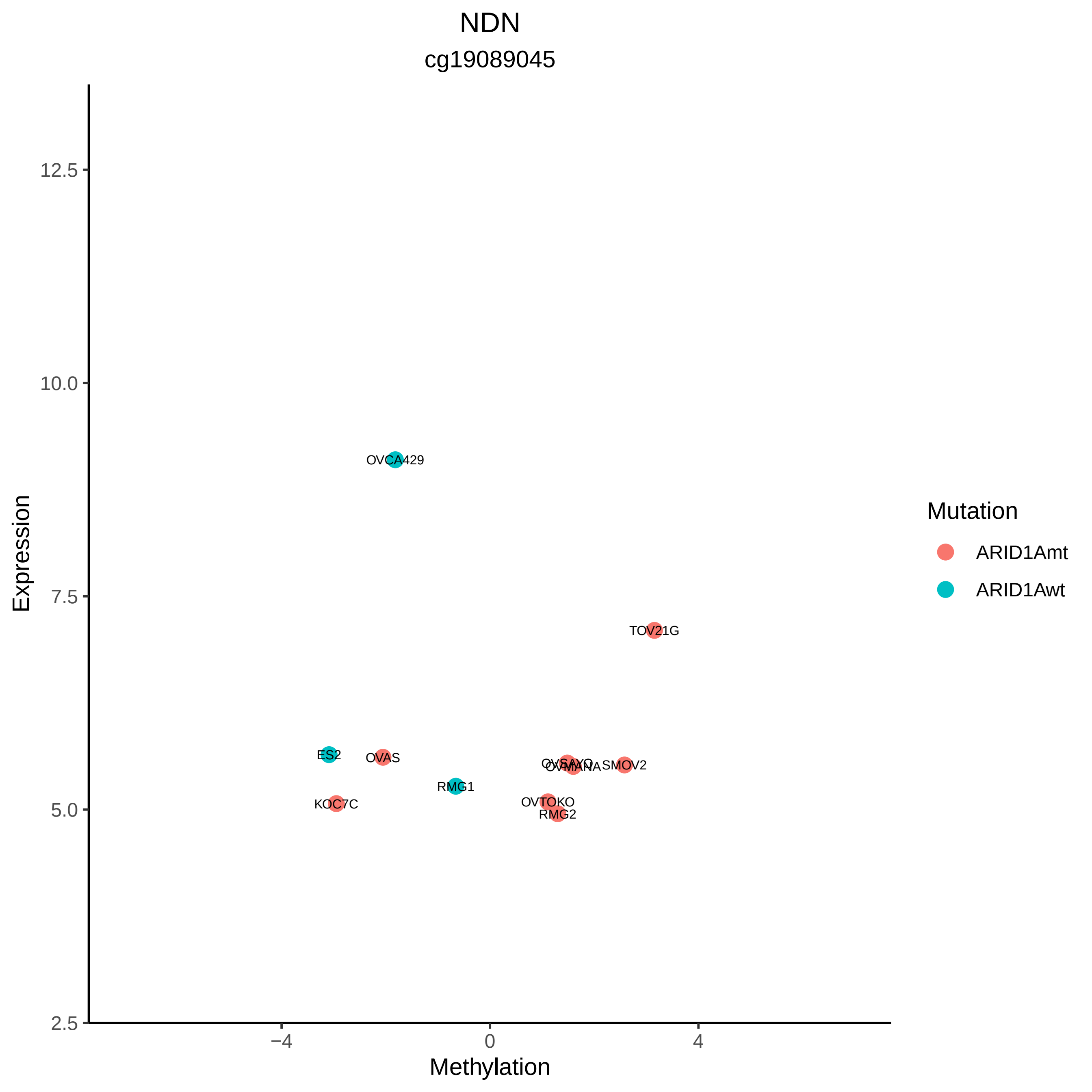

NDN
cg19089045
Expression
Methylation
12.5
10.0
7.5
5.0
2.5
−4
0
4
OVCA429
TOV21G
ES2
OVAS
OVSAYO
OVMANA
SMOV2
RMG1
KOC7C
OVTOKO
RMG2
Mutation
ARID1Amt
ARID1Awt

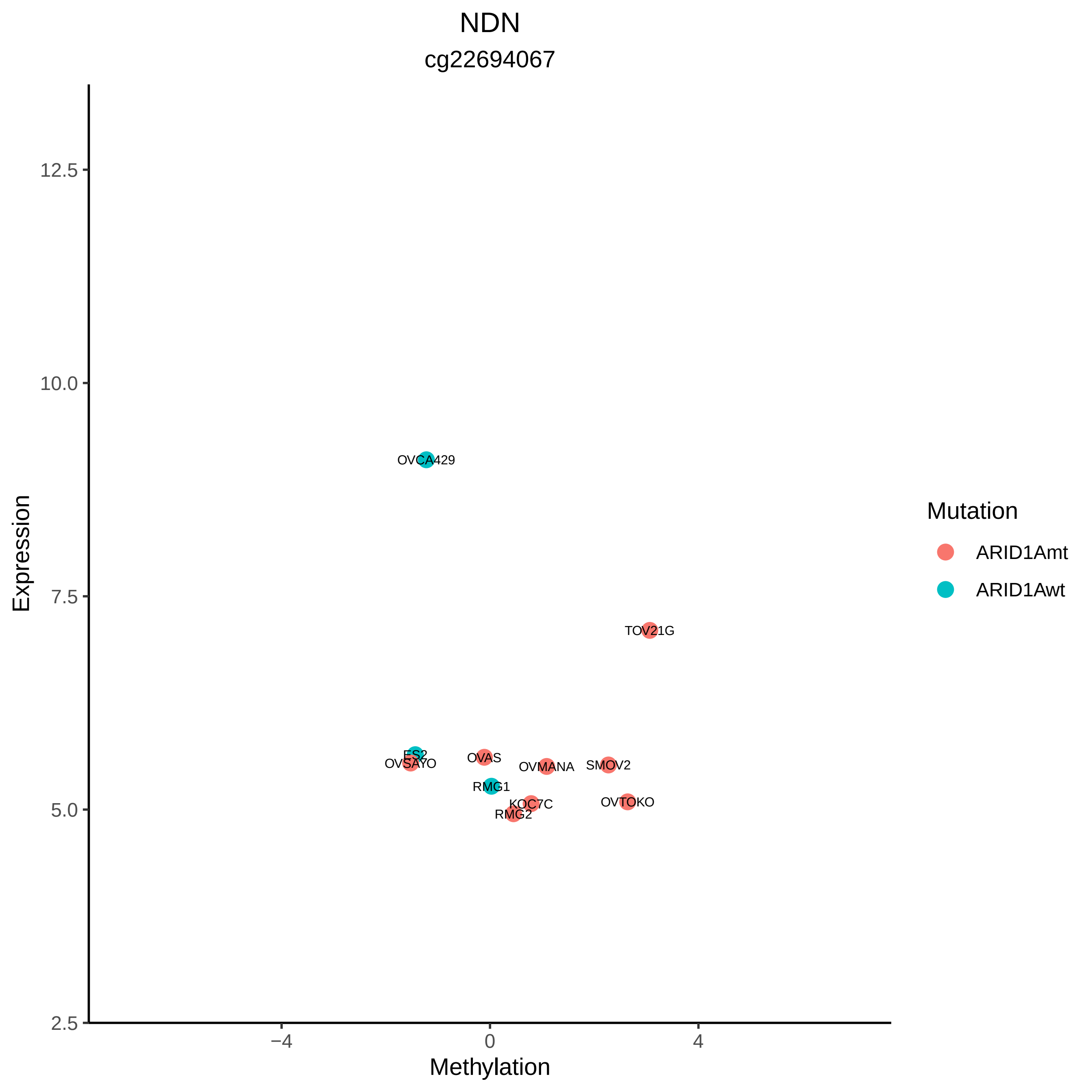
NDN
cg22694067
Expression
Methylation
12.5
10.0
7.5
5.0
2.5
−4
0
4
OVCA429
TOV21G
ES2
OVSAYO
OVAS
OVMANA
SMOV2
RMG1
KOC7C
RMG2
OVTOKO
Mutation
ARID1Amt
ARID1Awt

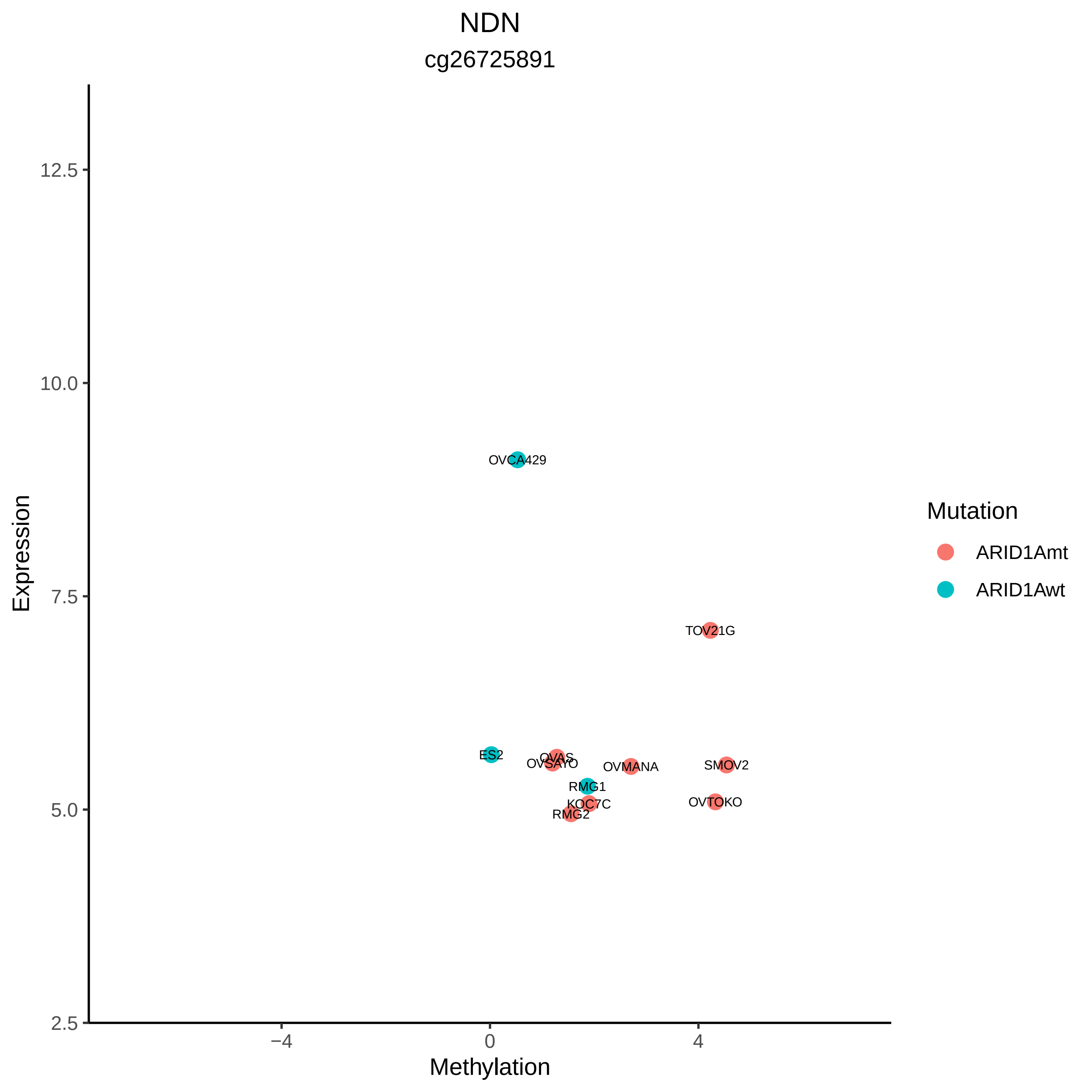
NDN
cg26725891
Expression
12.5
10.0
7.5
5.0
2.5
Methylation
−4
0
4
OVCA429
TOV21G
ES2
OVAS
OVSAYO
OVMANA
SMOV2
RMG1
KOC7C
RMG2
OVTOKO
Mutation
ARID1Amt
ARID1Awt

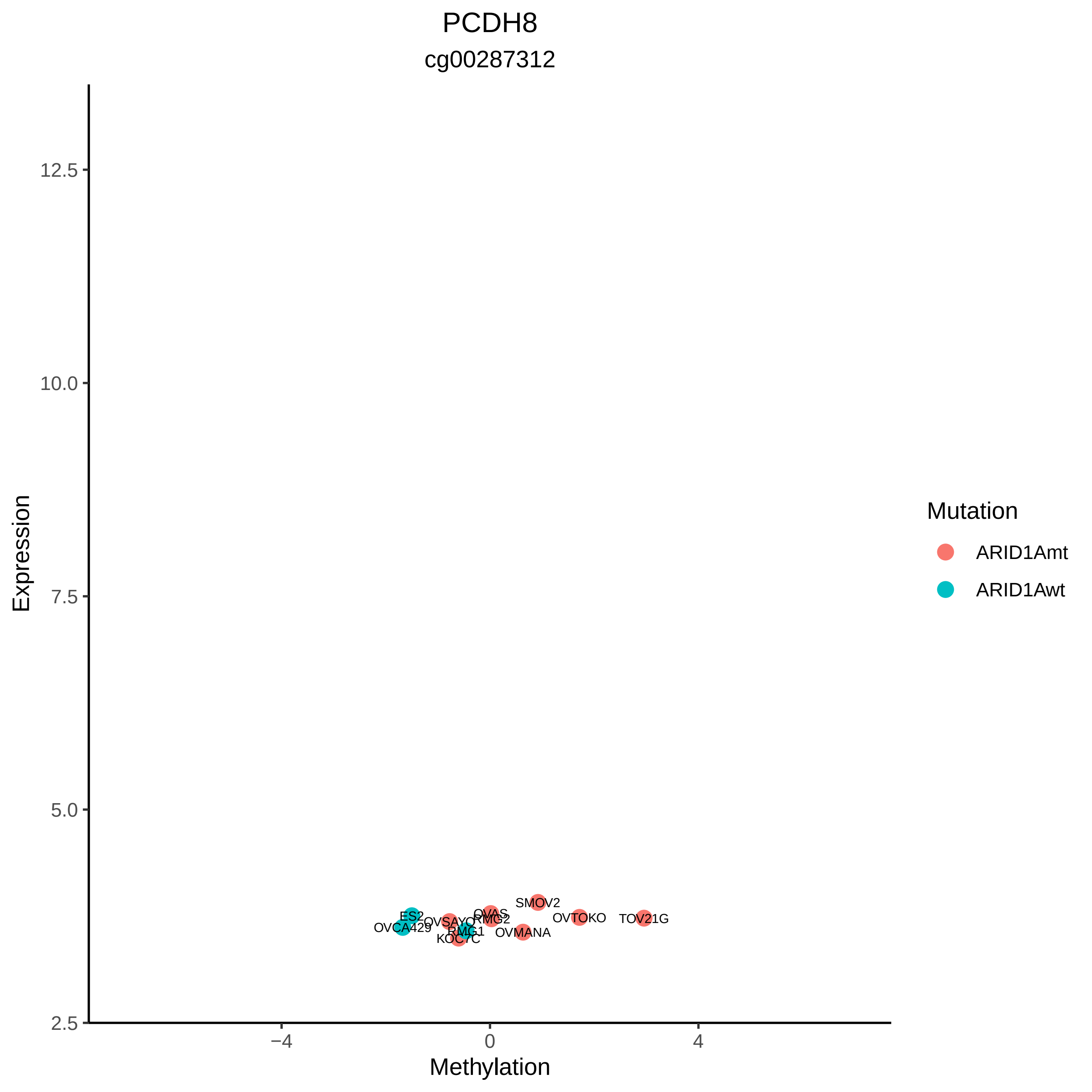
PCDH8
cg00287312
Expression
Methylation
12.5
10.0
7.5
5.0
2.5
−4
0
4
SMOV2
OVAS
RMG2
OVTOKO
TOV21G
ES2
OVSAYO
OVCA429
RMG1
KOC7C
OVMANA
Mutation
ARID1Amt
ARID1Awt

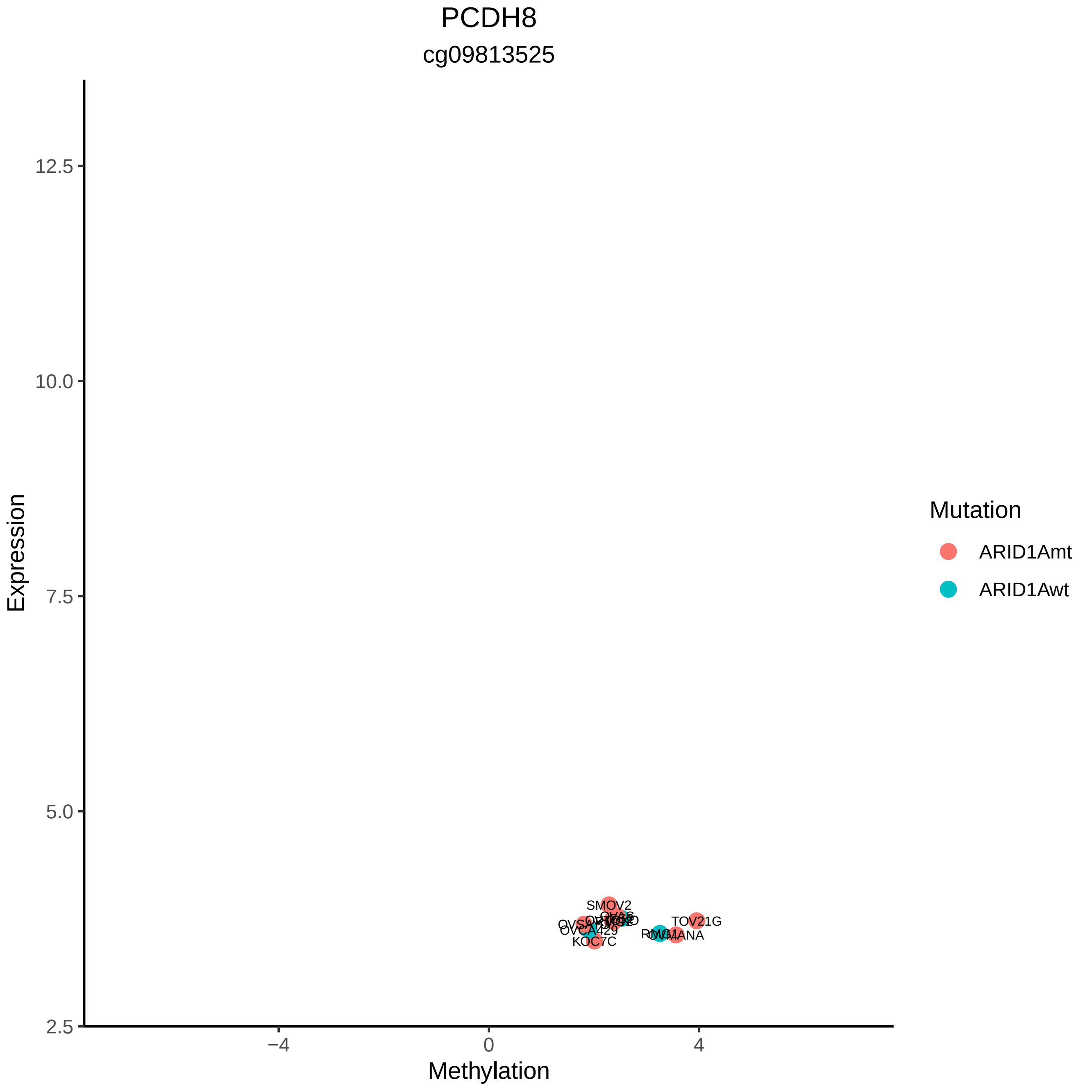
PCDH8
cg09813525
Expression
12.5
10.0
7.5
5.0
2.5
−4
0
4
Methylation
SMOV2
TOV21G
KOC7C
Mutation
ARID1Amt
ARID1Awt

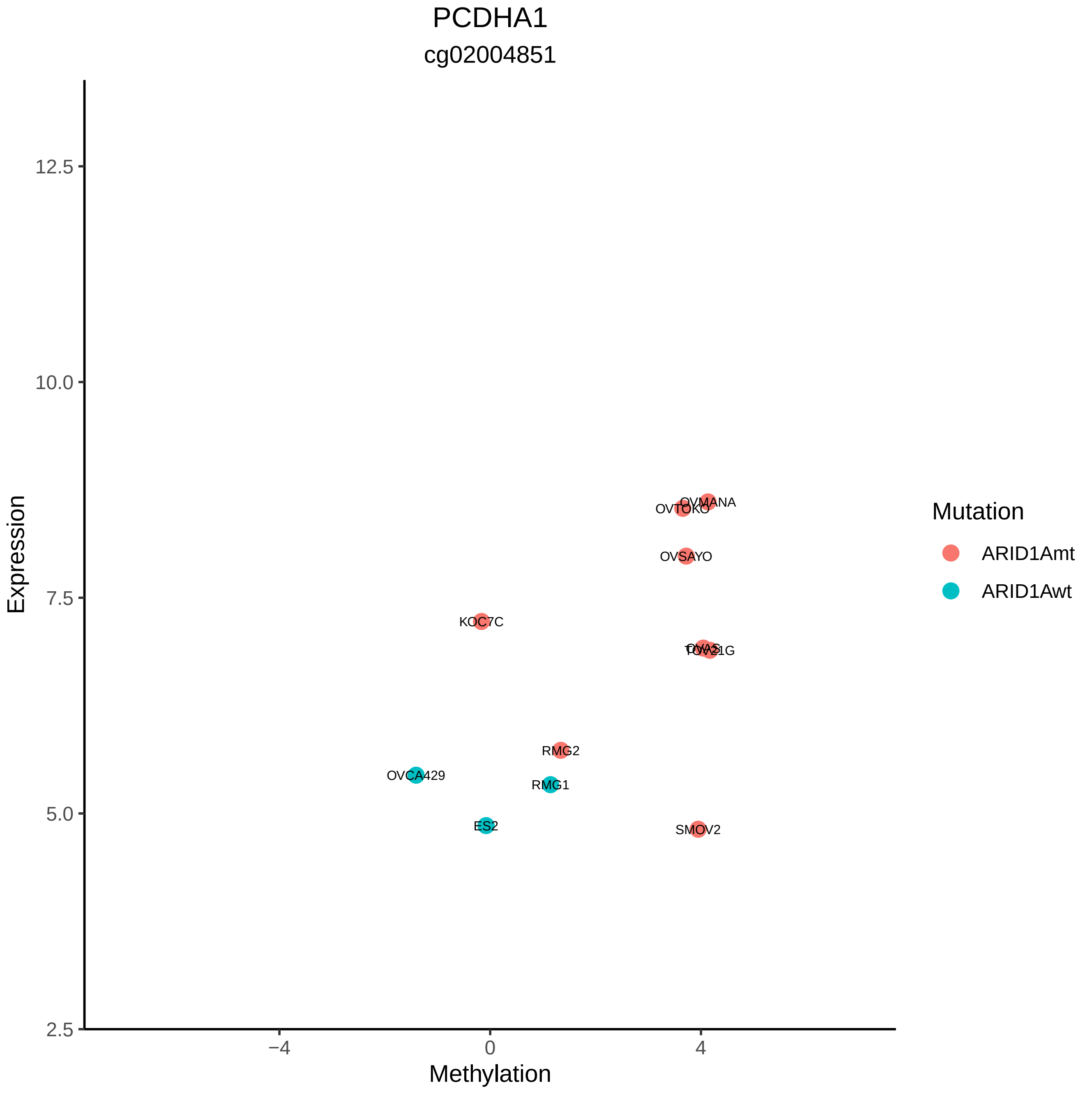
PCDHA1
cg02004851
Expression
12.5
10.0
7.5
5.0
2.5
Methylation
−4
0
4
OVMANA
OVTOKO
OVSAYO
KOC7C
OVAS
TOV21G
RMG2
OVCA429
RMG1
ES2
SMOV2
Mutation
ARID1Amt
ARID1Awt

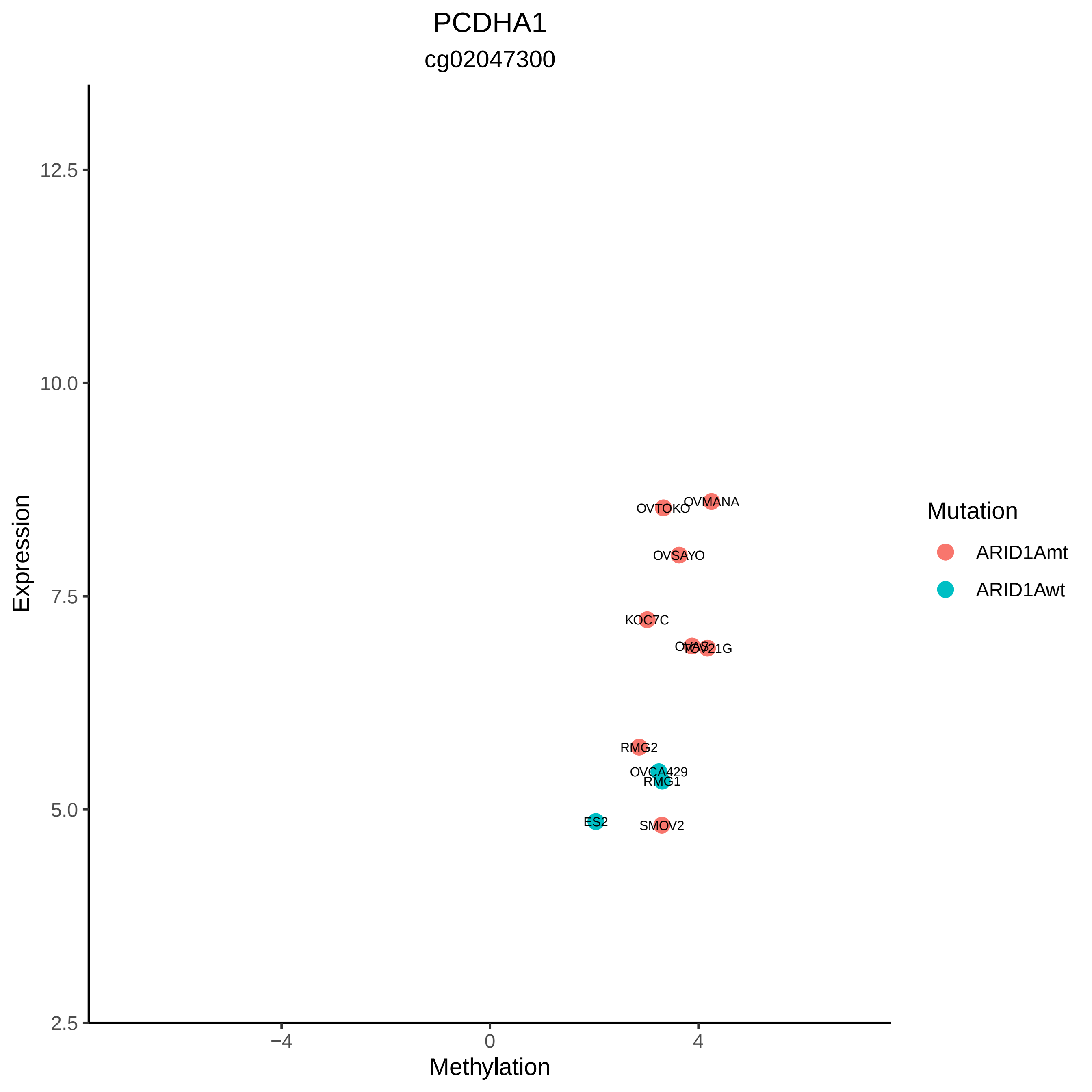
PCDHA1
cg02047300
Expression
Methylation
12.5
10.0
7.5
5.0
2.5
−4
0
4
Mutation
ARID1Amt
ARID1Awt
OVMANA
OVTOKO
OVSAYO
KOC7C
OVAS
TOV21G
RMG2
OVCA429
RMG1
ES2
SMOV2

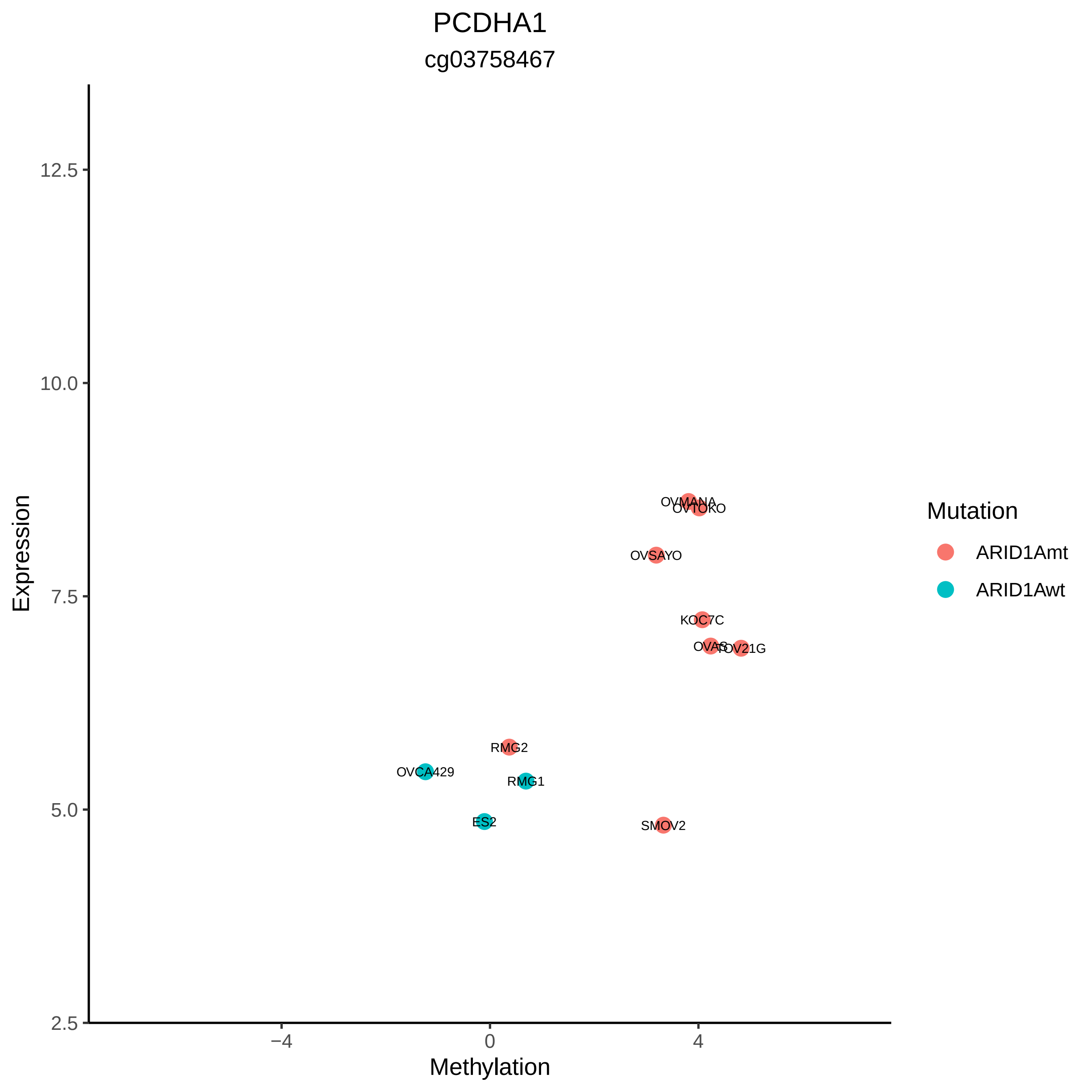

PCDHA1
cg03758467
Expression
Methylation
12.5
10.0
7.5
5.0
2.5
−4
0
4
OVMANA
OVTOKO
OVSAYO
KOC7C
OVAS
TOV21G
RMG2
OVCA429
RMG1
ES2
SMOV2
Mutation
ARID1Amt
ARID1Awt

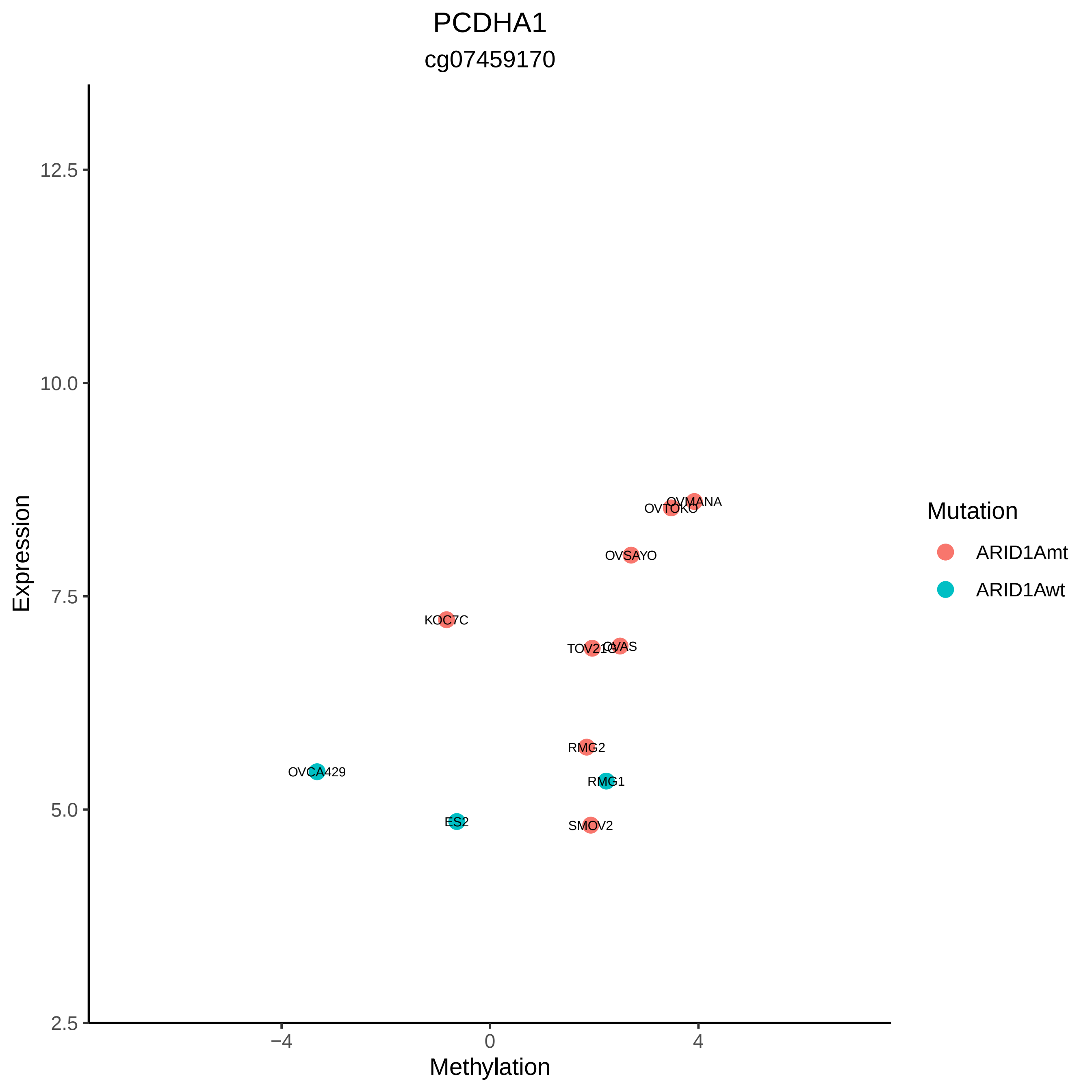
PCDHA1
cg07459170
Expression
Methylation
12.5
10.0
7.5
5.0
2.5
−4
0
4
OVMANA
OVTOKO
OVSAYO
KOC7C
TOV21G
OVAS
RMG2
OVCA429
RMG1
ES2
SMOV2
Mutation
ARID1Amt
ARID1Awt

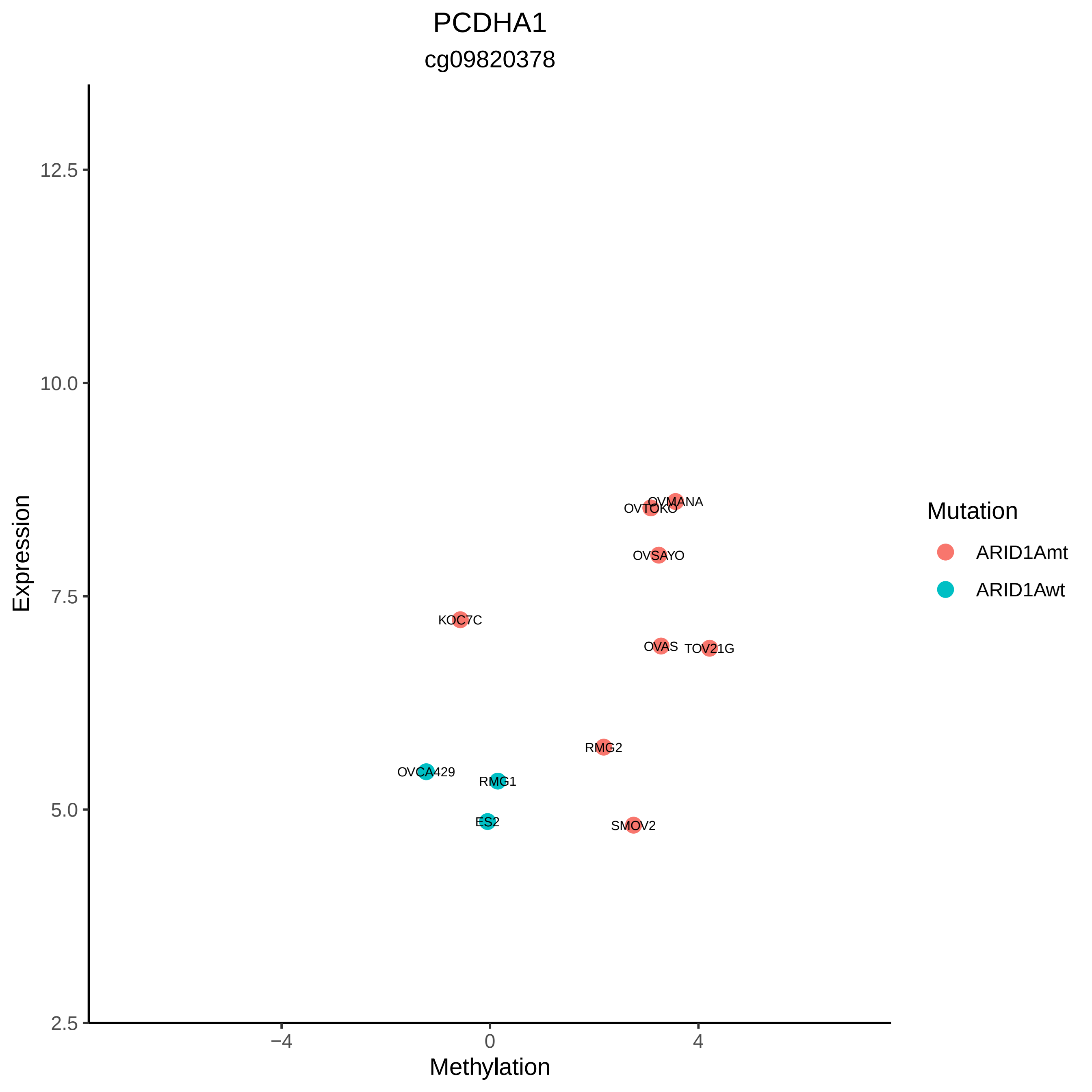
PCDHA1
cg09820378
Expression
Methylation
12.5
10.0
7.5
5.0
2.5
−4
0
4
OVMANA
OVTOKO
OVSAYO
KOC7C
OVAS
TOV21G
RMG2
OVCA429
RMG1
ES2
SMOV2
Mutation
ARID1Amt
ARID1Awt

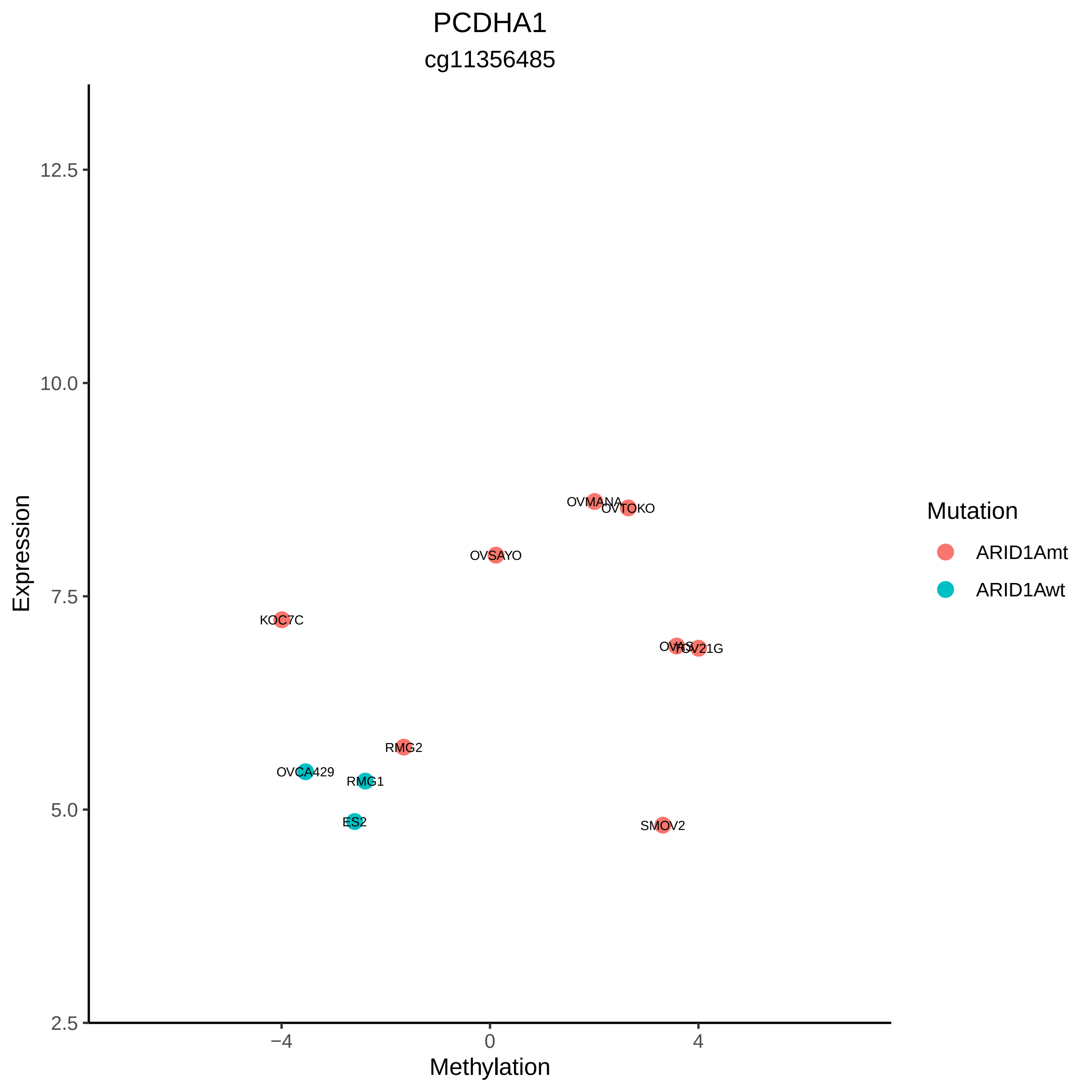
PCDHA1
cg11356485
Expression
Methylation
12.5
10.0
7.5
5.0
2.5
−4
0
4
OVMANA
OVTOKO
OVSAYO
KOC7C
OVISE
TOV21G
RMG2
OVCA429
RMG1
ES2
SMOV2
Mutation
ARID1Amt
ARID1Awt

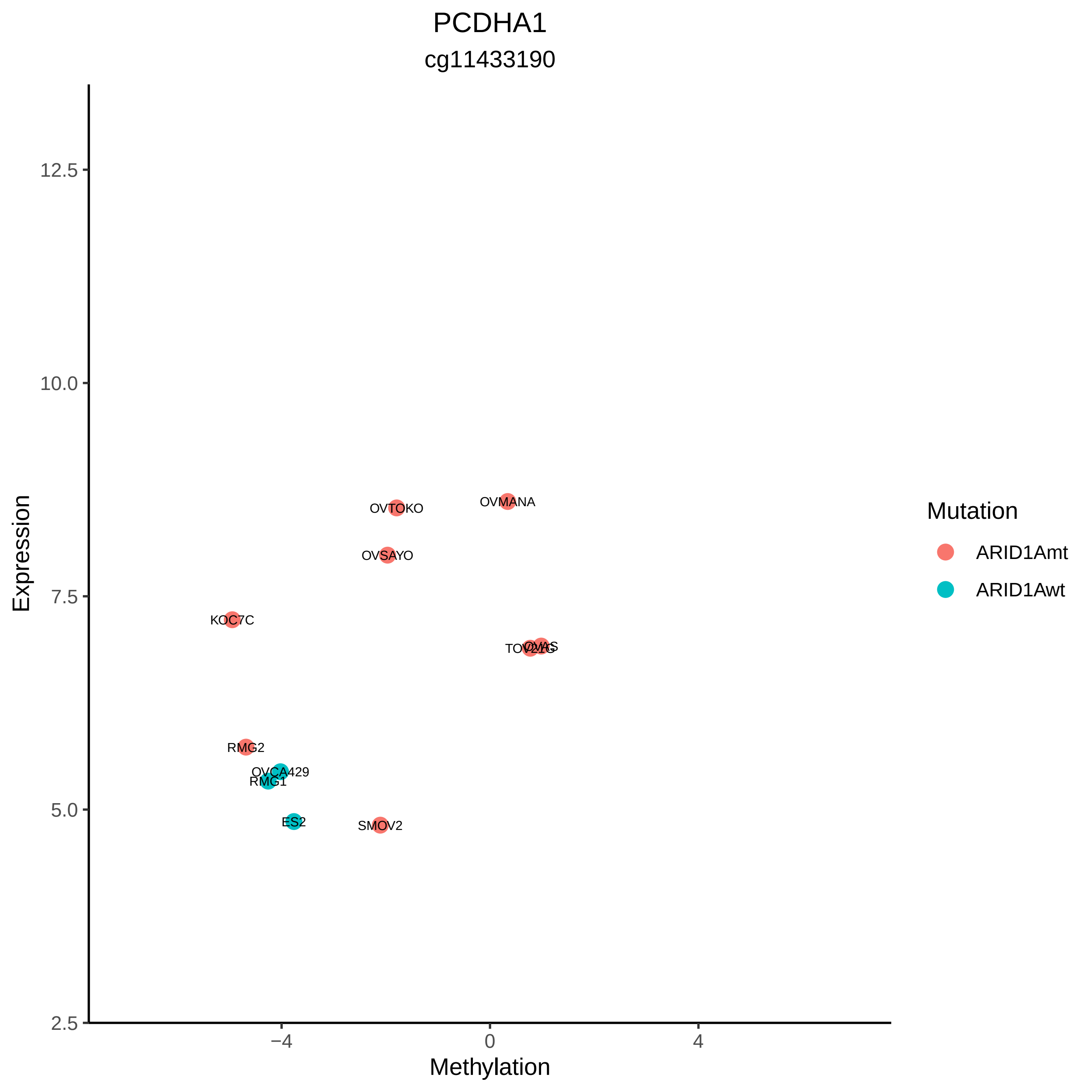

PCDHA1
cg11433190
Expression
Methylation
12.5
10.0
7.5
5.0
2.5
−4
0
4
OVTOKO
OVMANA
OVSAYO
KOC7C
TOV21G
OVAS
RMG2
OVCA429
RMG1
ES2
SMOV2
Mutation
ARID1Amt
ARID1Awt

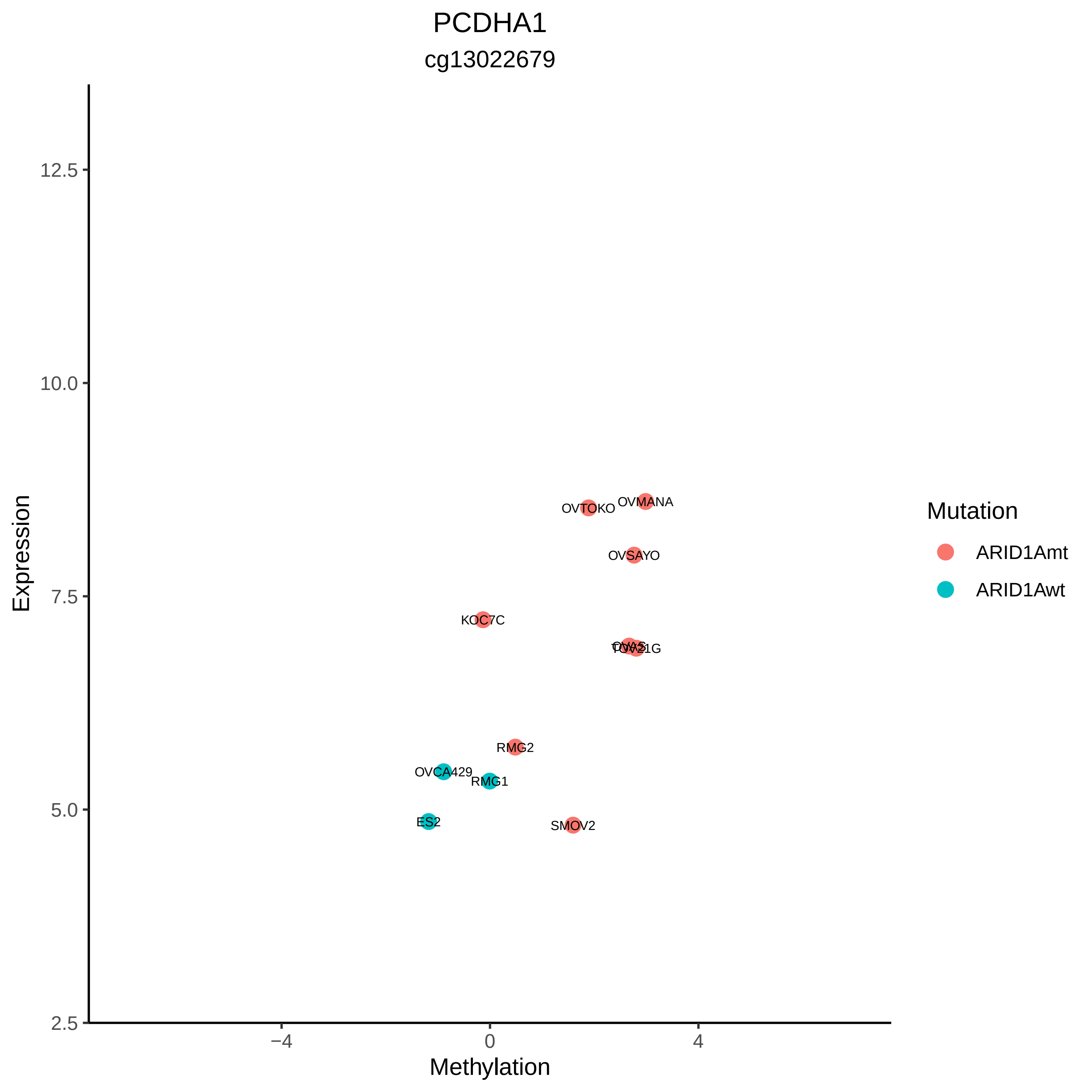

PCDHA1
cg13022679
Expression
Methylation
12.5
10.0
7.5
5.0
2.5
−4
0
4
OVTOKO
OVMANA
OVSAYO
KOC7C
OVISE
TOV21G
RMG2
OVCA429
RMG1
ES2
SMOV2
Mutation
ARID1Amt
ARID1Awt

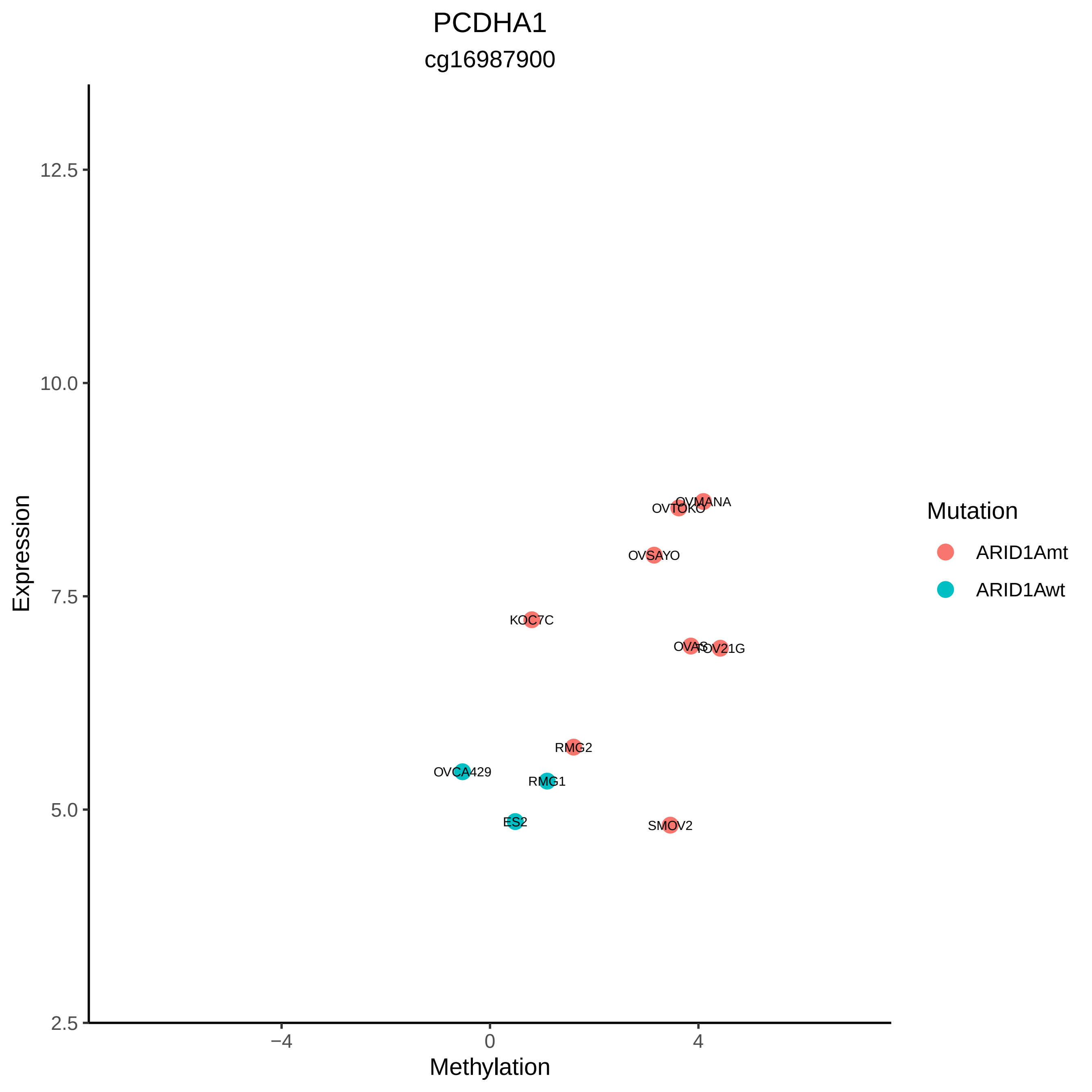
PCDHA1
cg16987900
Expression
Methylation
12.5
10.0
7.5
5.0
2.5
−4
0
4
OVMANA
OVTOKO
OVSAYO
KOC7C
OVISE
TOV21G
RMG2
OVCA429
RMG1
ES2
SMOV2
Mutation
ARID1Amt
ARID1Awt

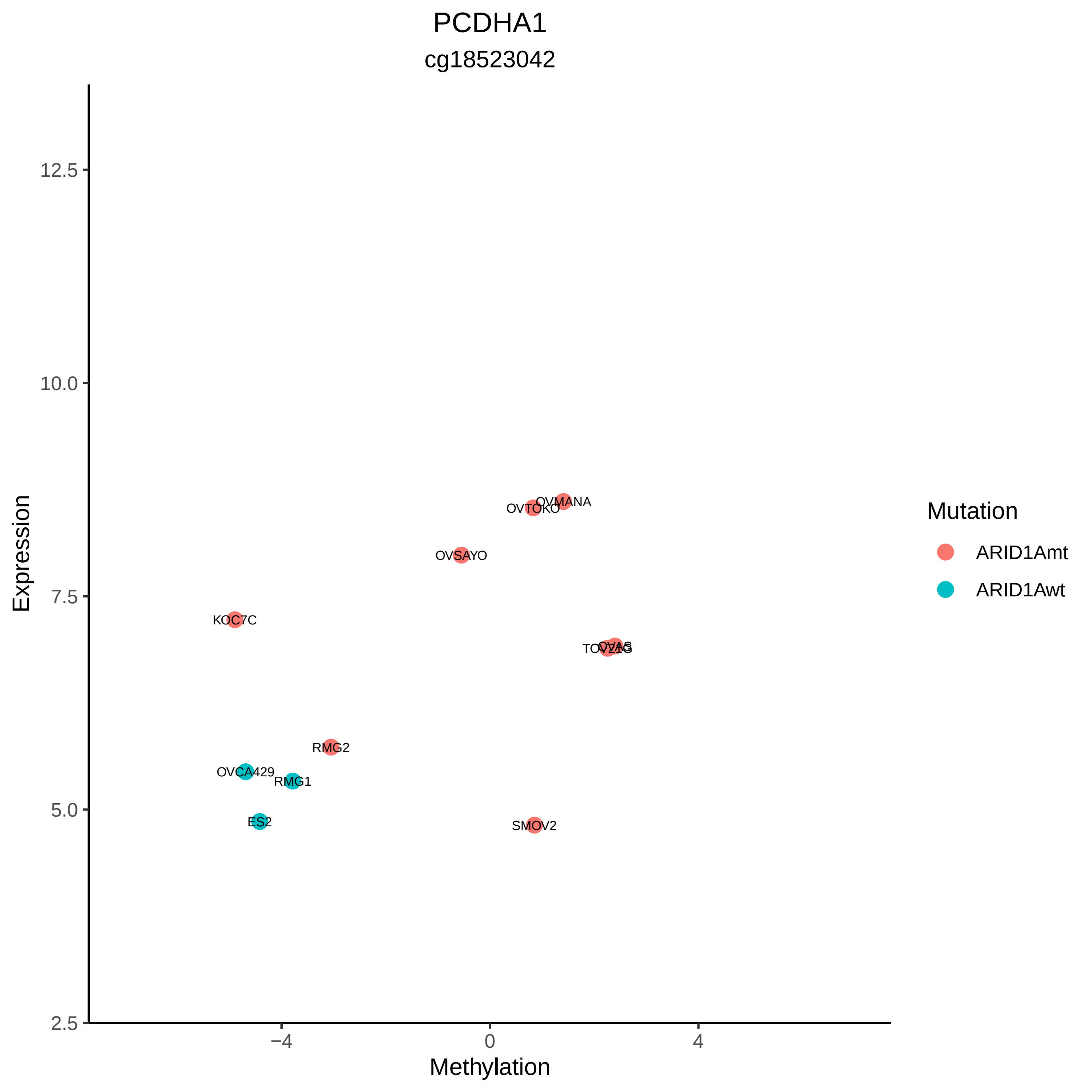

PCDHA1
cg18523042
Expression
Methylation
12.5
10.0
7.5
5.0
2.5
−4
0
4
OVMANA
OVTOKO
OVSAYO
KOC7C
TOV21G
OVAS
RMG2
OVCA429
RMG1
ES2
SMOV2
Mutation
ARID1Amt
ARID1Awt

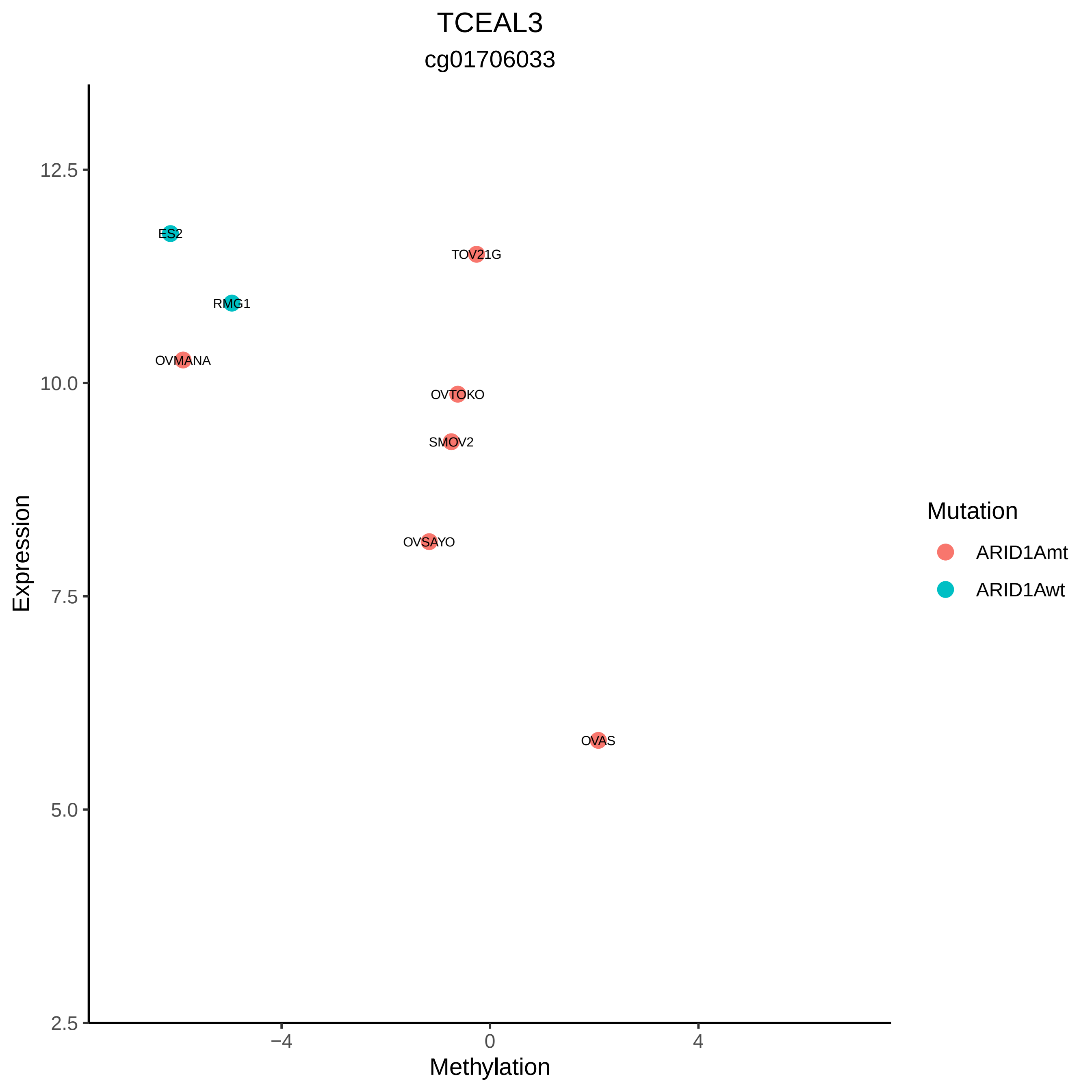
TCEAL3
cg01706033
Expression
Methylation
12.5
10.0
7.5
5.0
2.5
−4
0
4
ES2
TOV21G
RMG1
OVMANA
OVTOKO
SMOV2
OVSAYO
OVAS
Mutation
ARID1Amt
ARID1Awt

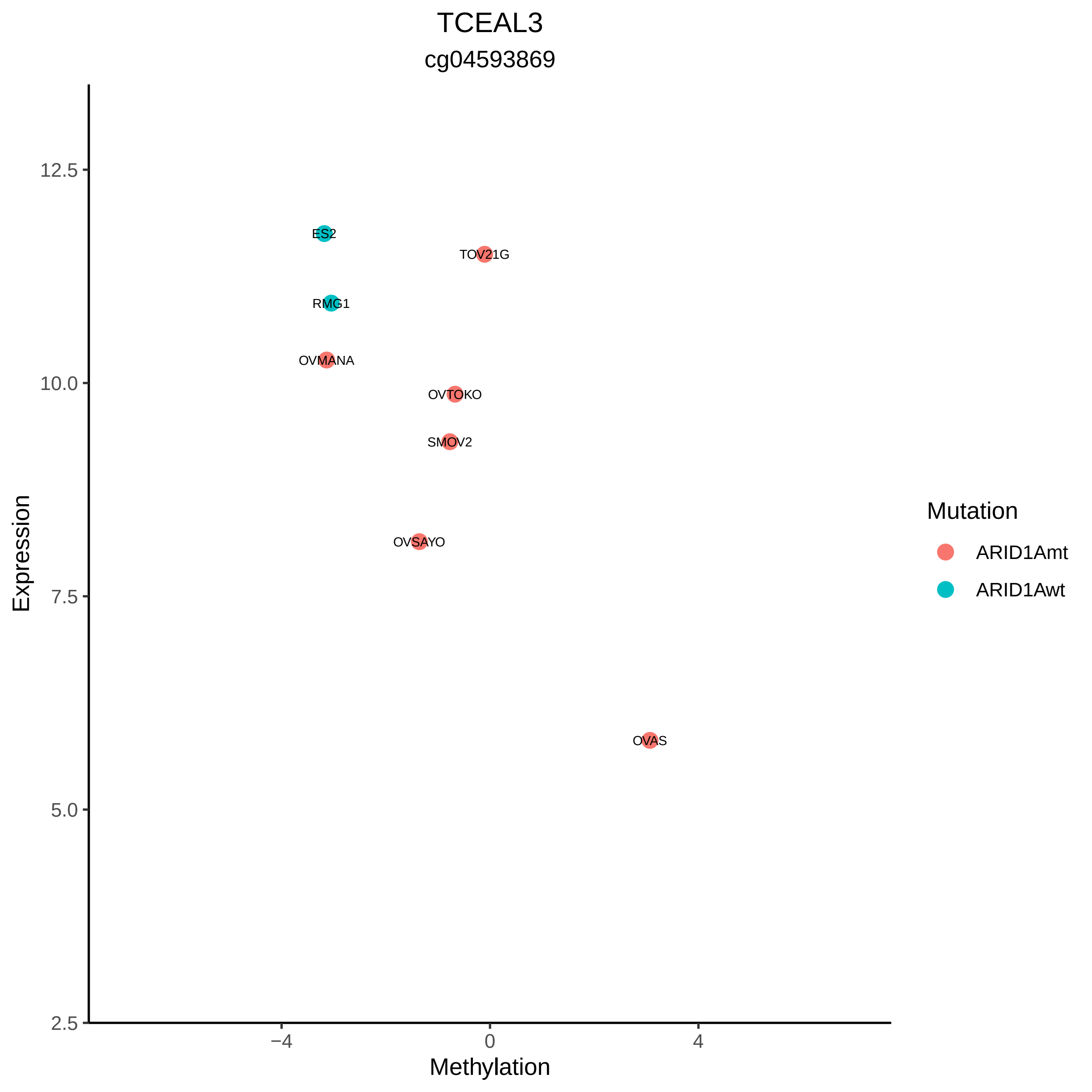
TCEAL3
cg04593869
Expression
Methylation
12.5
10.0
7.5
5.0
2.5
−4
0
4
ES2
TOV21G
RMG1
OVMANA
OVTOKO
SMOV2
OVSAYO
OVAS
Mutation
ARID1Amt
ARID1Awt

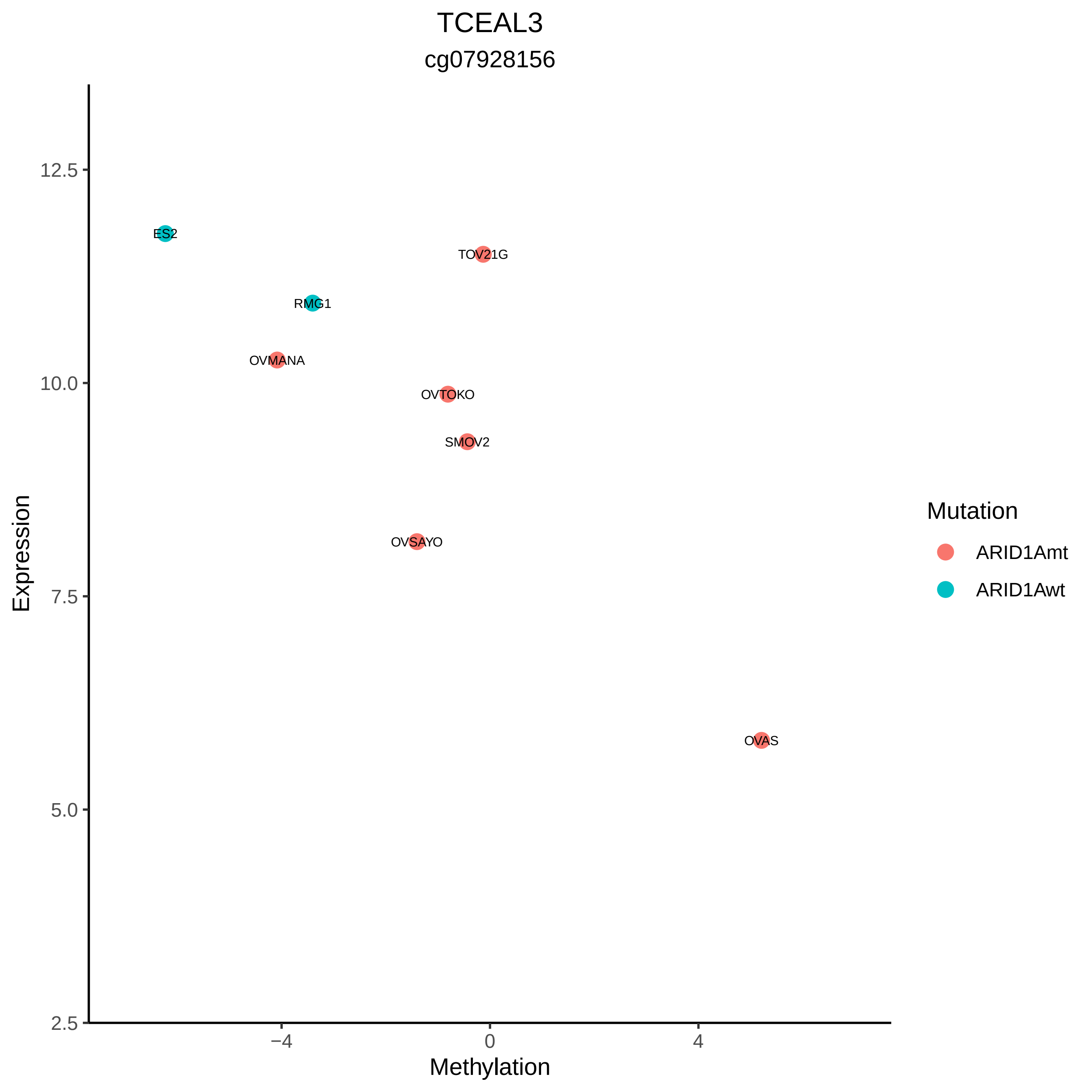

TCEAL3
cg07928156
Expression
Methylation
12.5
10.0
7.5
5.0
2.5
−4
0
4
ES2
TOV21G
RMG1
OVMANA
OVTOKO
SMOV2
OVSAYO
OVAS
Mutation
ARID1Amt
ARID1Awt

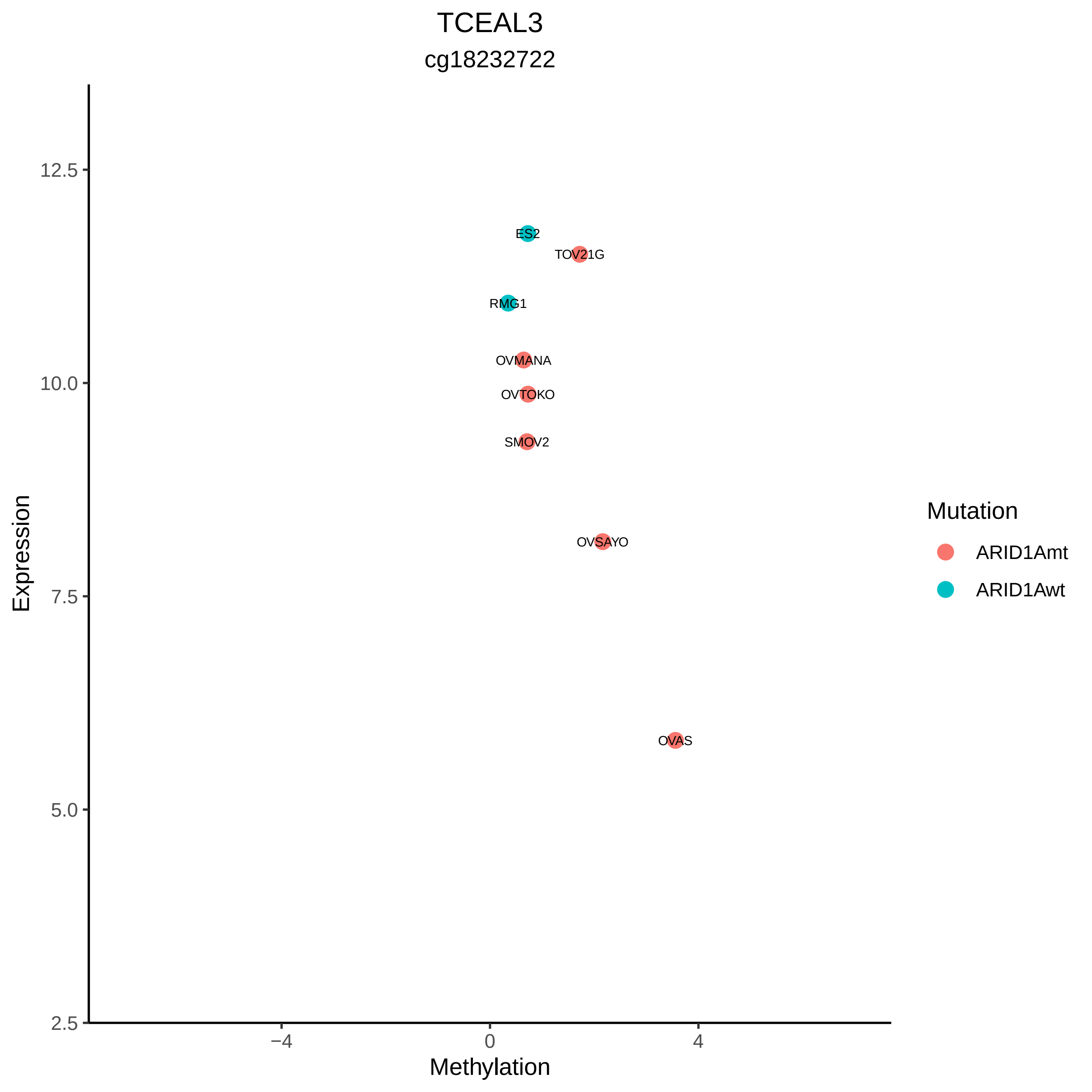

TCEAL3
cg18232722
Expression
Methylation
12.5
10.0
7.5
5.0
2.5
−4
0
4
ES2
TOV21G
RMG1
OVMANA
OVTOKO
SMOV2
OVSAYO
OVAS
Mutation
ARID1Amt
ARID1Awt

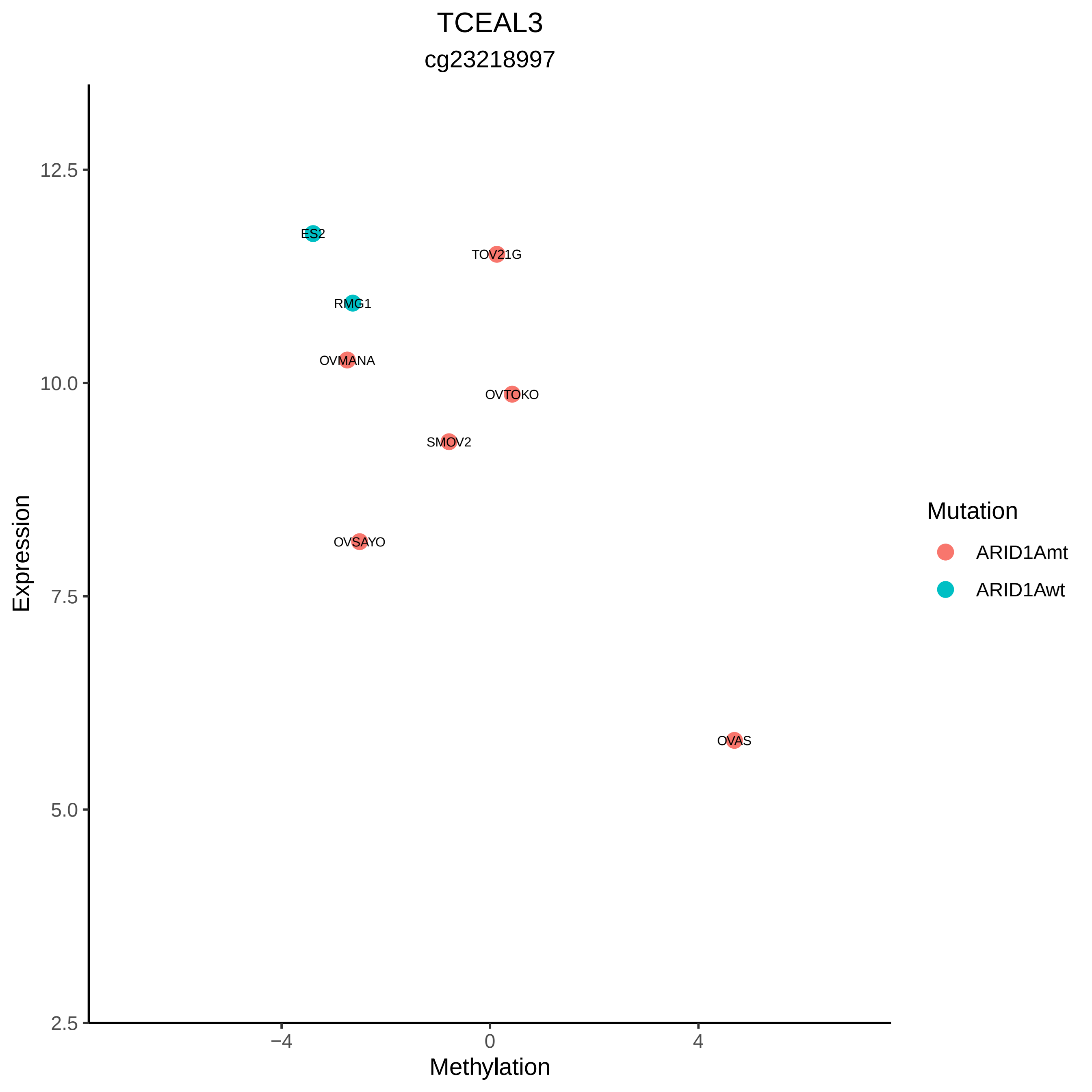
TCEAL3
cg23218997
Expression
Methylation
12.5
10.0
7.5
5.0
2.5
−4
0
4
ES2
TOV21G
RMG1
OVMANA
OVTOKO
SMOV2
OVSAYO
OVAS
Mutation
ARID1Amt
ARID1Awt

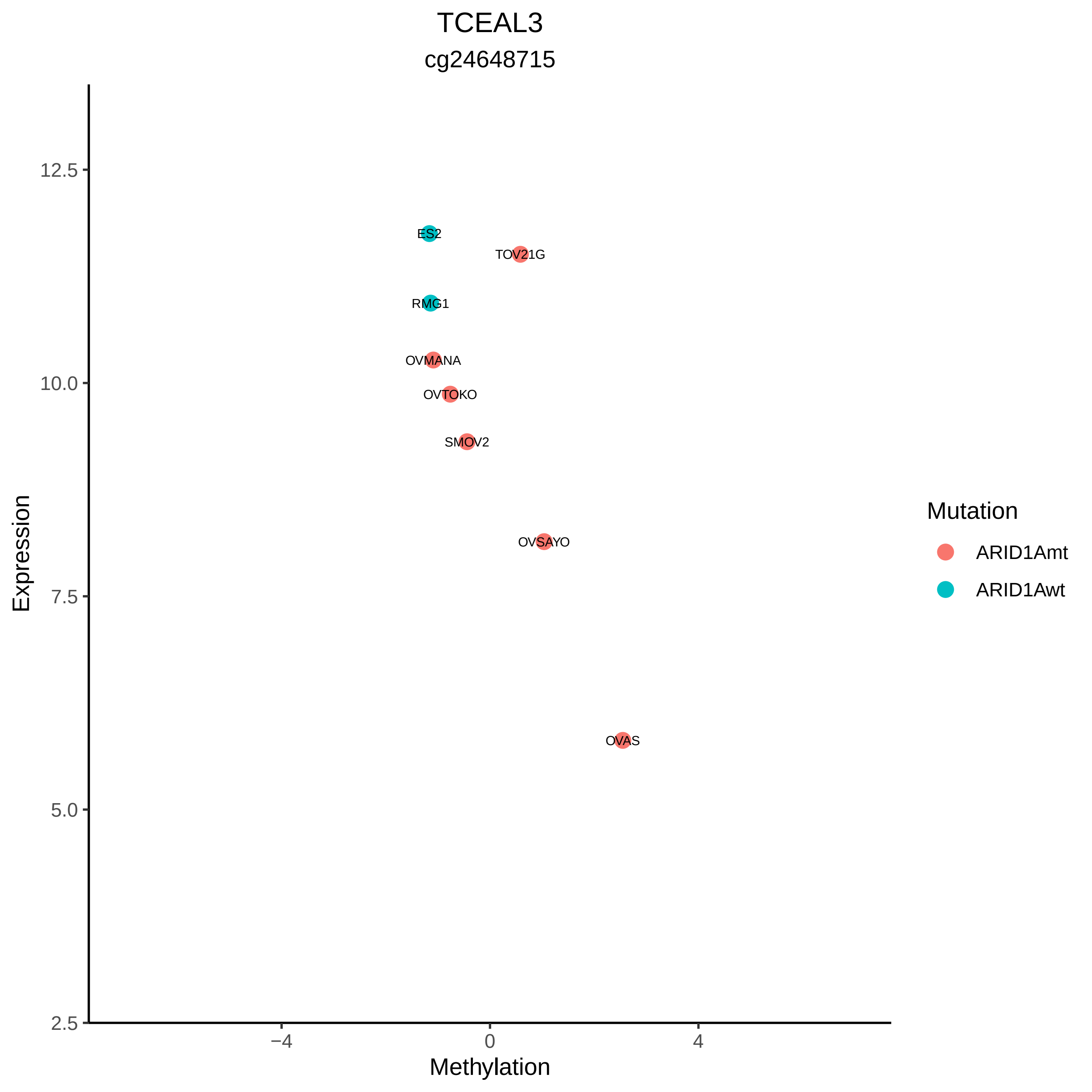

TCEAL3
cg24648715
Expression
Methylation
12.5
10.0
7.5
5.0
2.5
−4
0
4
ES2
TOV21G
RMG1
OVMANA
OVTOKO
SMOV2
OVSAYO
OVAS
Mutation
ARID1Amt
ARID1Awt

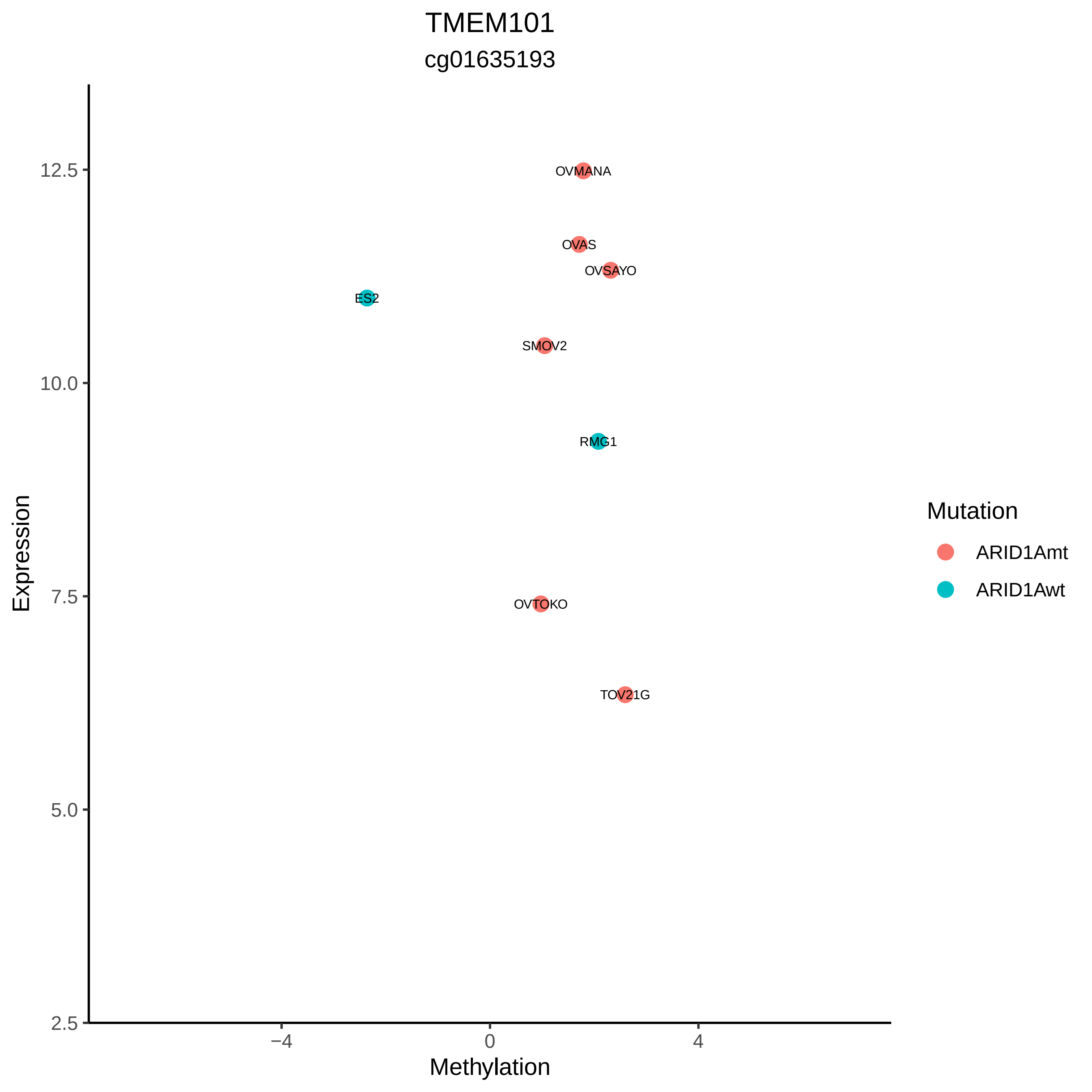
TMEM101
cg01635193
12.5
10.0
7.5
5.0
2.5
−4
0
4
Methylation
Expression
OVMANA
OVAS
OVSAYO
ES2
SMOV2
RMG1
OVTOKO
TOV21G
Mutation
ARID1Amt
ARID1Awt

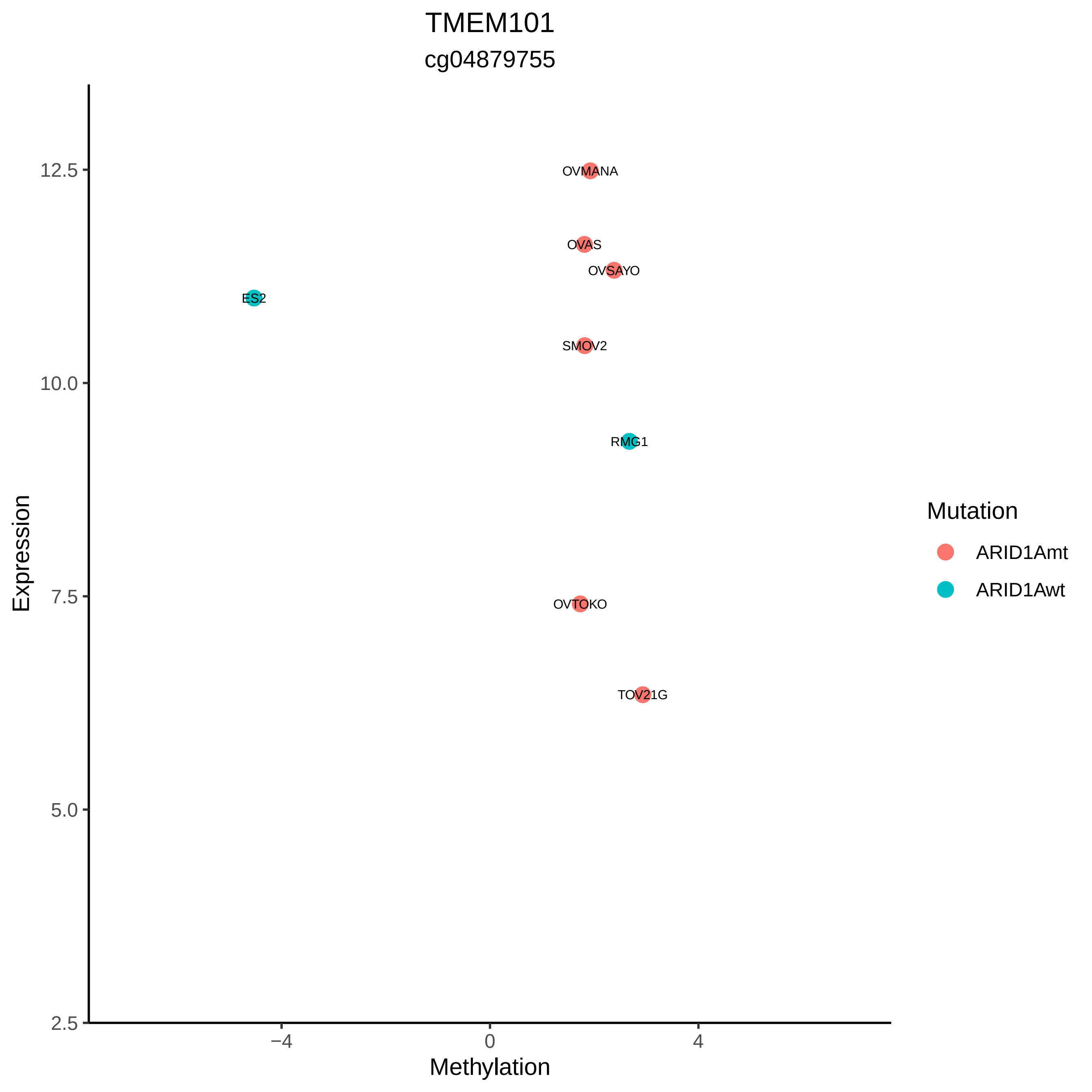
TMEM101
cg04879755
Expression
12.5
10.0
7.5
5.0
2.5
Methylation
−4
0
4
OVMANA
OVAS
OVSAYO
ES2
SMOV2
RMG1
OVTOKO
TOV21G
Mutation
ARID1Amt
ARID1Awt

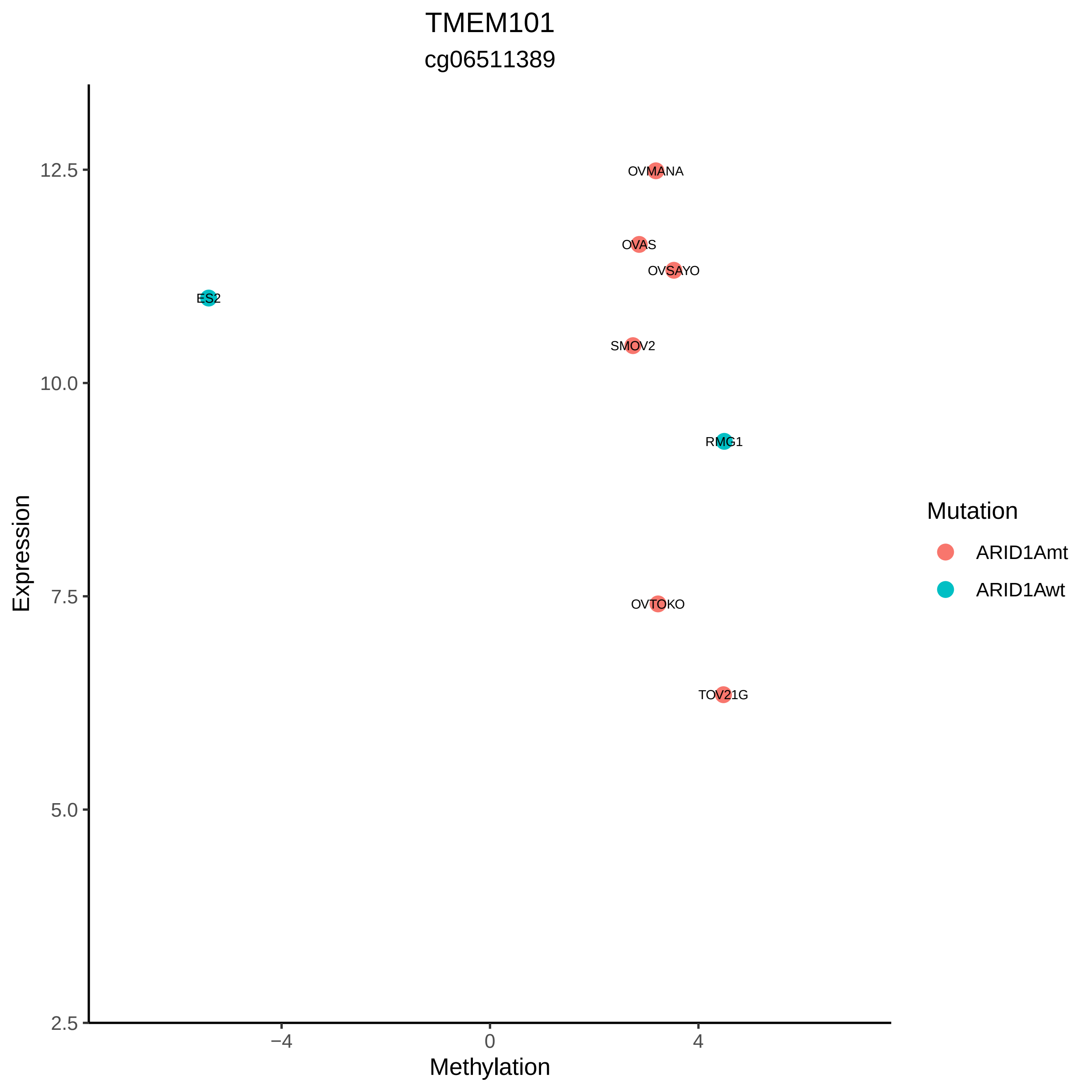

TMEM101
cg06511389
Expression
12.5
10.0
7.5
5.0
2.5
−4
0
4
Methylation
OVMANA
OVAS
OVSAYO
ES2
SMOV2
RMG1
OVTOKO
TOV21G
Mutation
ARID1Amt
ARID1Awt

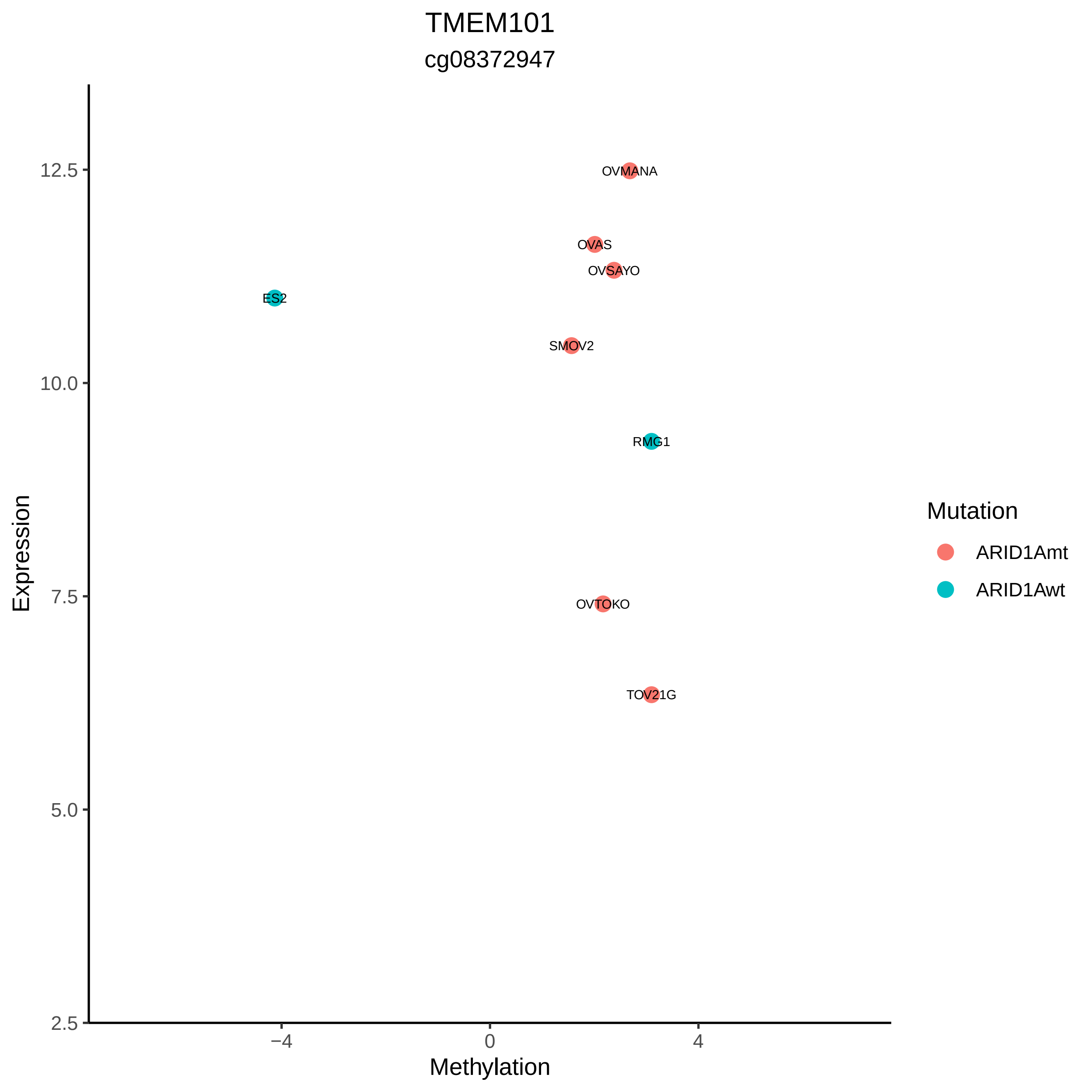
TMEM101
cg08372947
Expression
Methylation
12.5
10.0
7.5
5.0
2.5
−4
0
4
OVMANA
OVAS
OVSAYO
ES2
SMOV2
RMG1
OVTOKO
TOV21G
Mutation
ARID1Amt
ARID1Awt

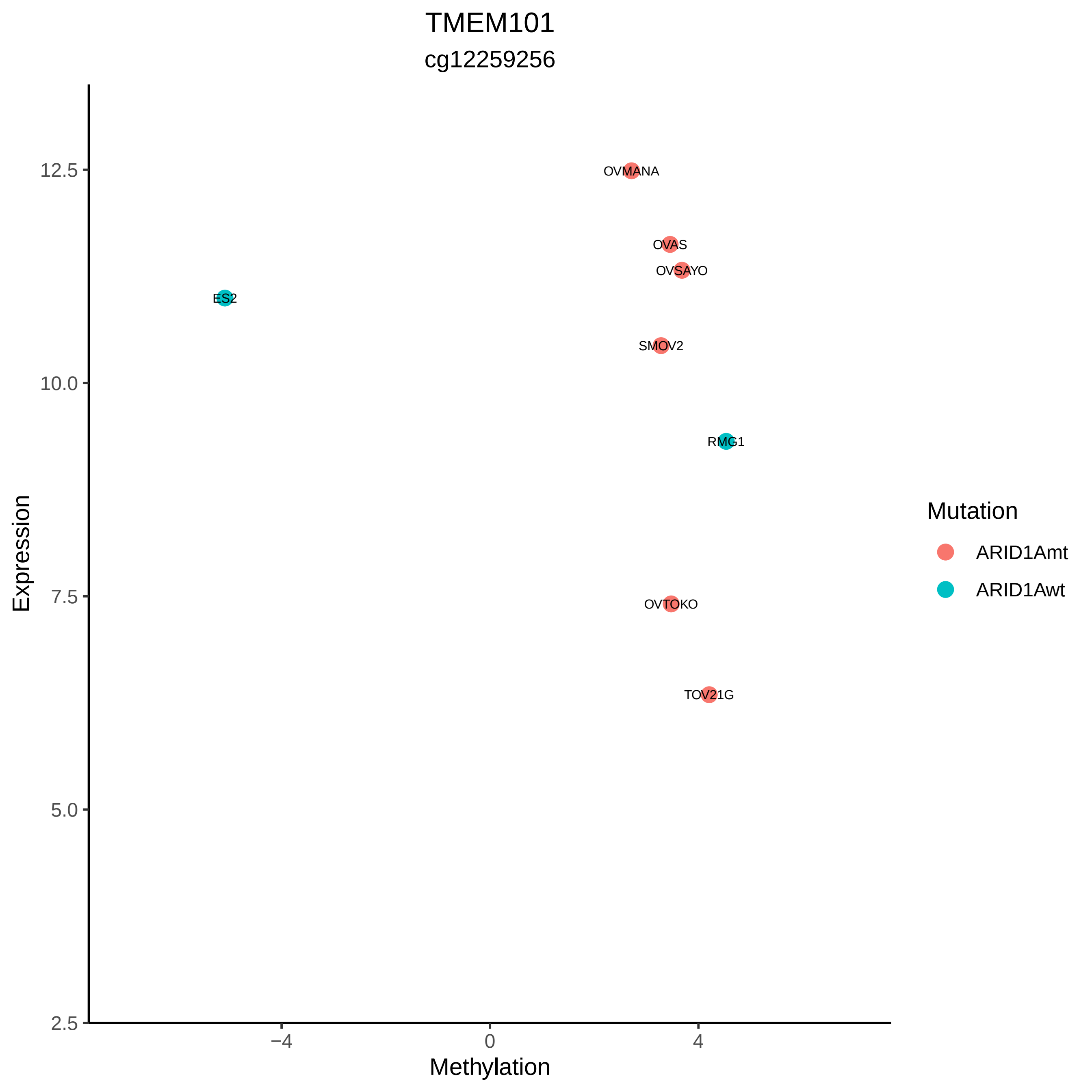

TMEM101
cg12259256
Expression
Methylation
12.5
10.0
7.5
5.0
2.5
−4
0
4
OVMANA
OVAS
OVSAYO
ES2
SMOV2
RMG1
OVTOKO
TOV21G
Mutation
ARID1Amt
ARID1Awt

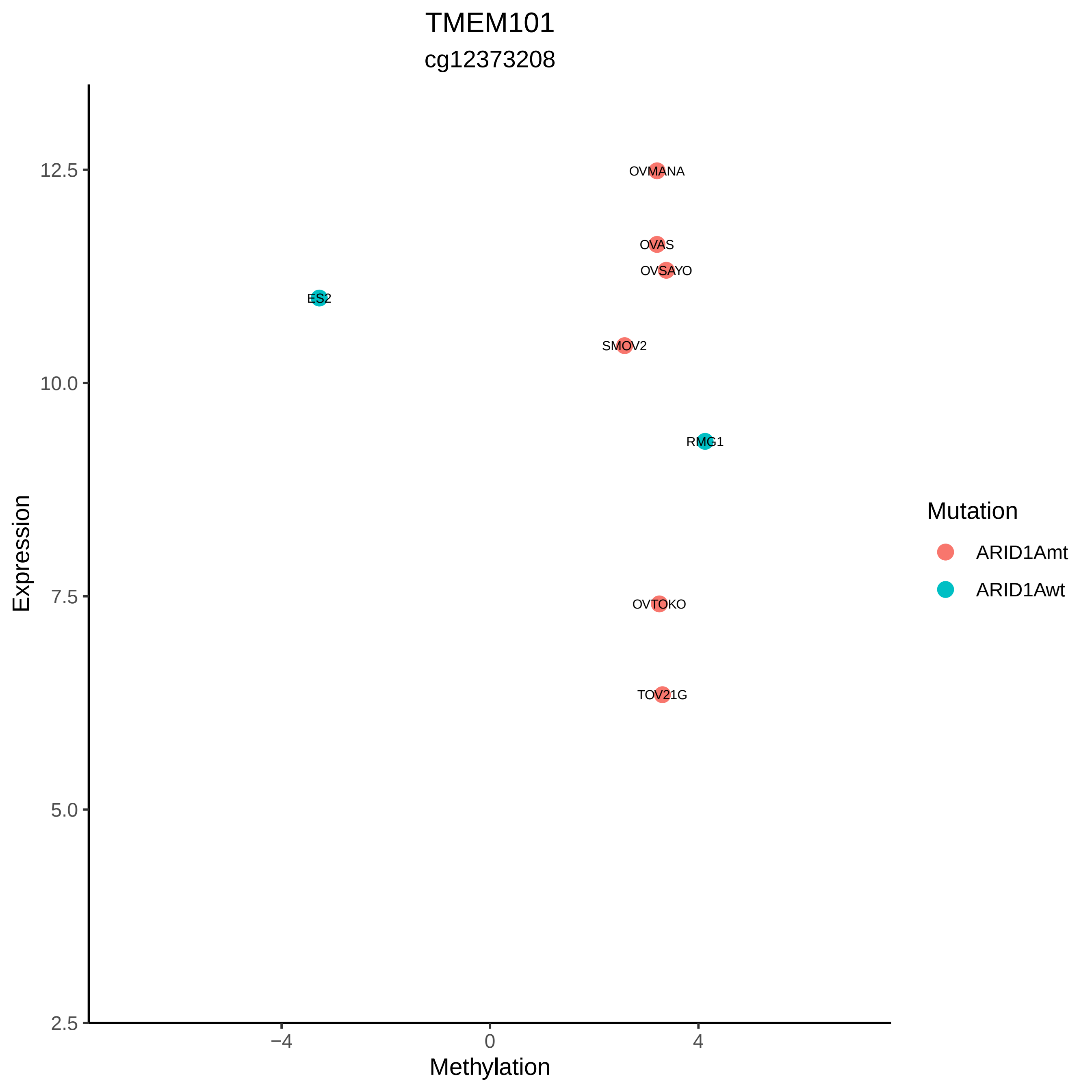
TMEM101
cg12373208
Expression
Methylation
12.5
10.0
7.5
5.0
2.5
−4
0
4
OVMANA
OVAS
OVSAYO
ES2
SMOV2
RMG1
OVTOKO
TOV21G
Mutation
ARID1Amt
ARID1Awt

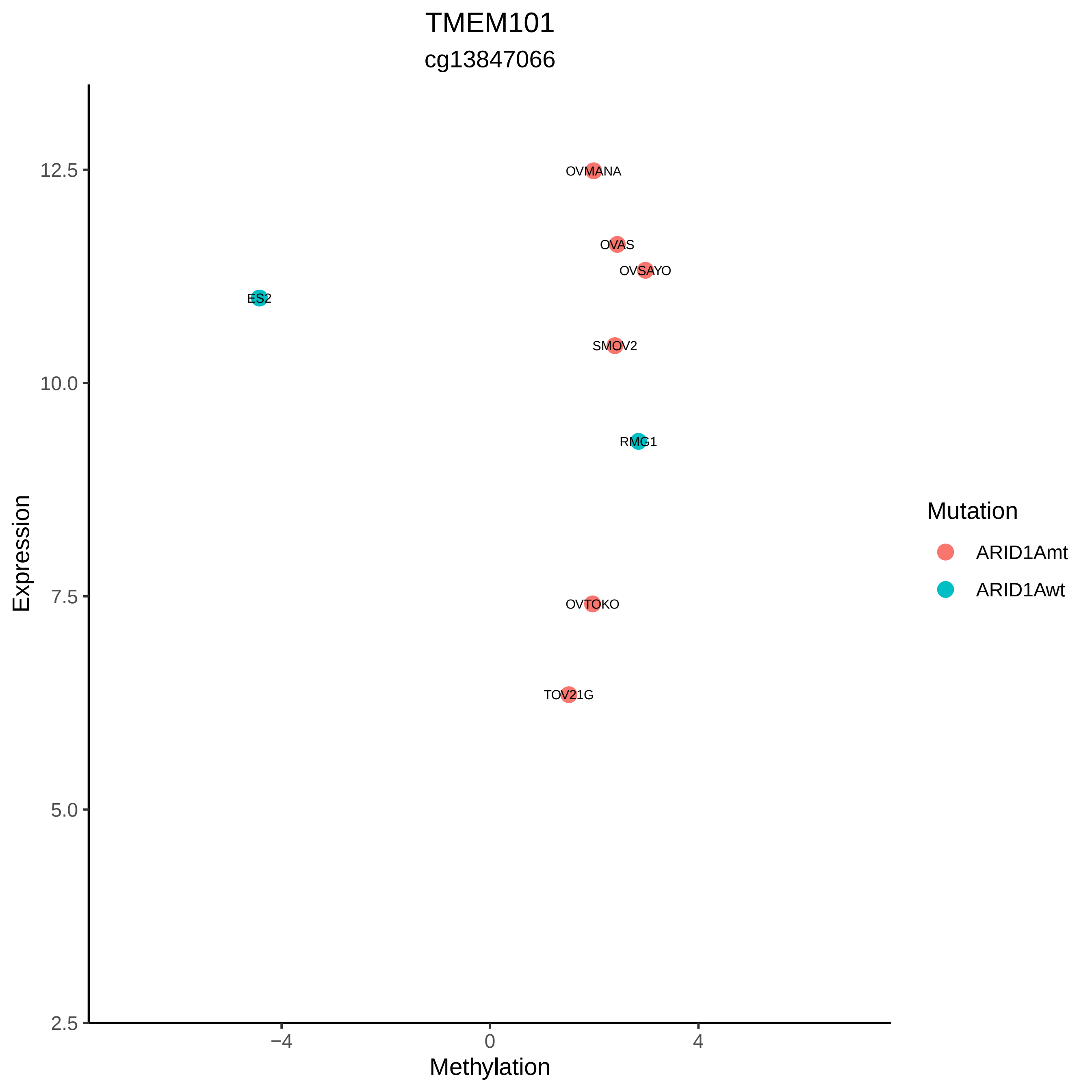
TMEM101
cg13847066
Expression
Methylation
12.5
10.0
7.5
5.0
2.5
−4
0
4
OVMANA
OVAS
OVSAYO
ES2
SMOV2
RMG1
OVTOKO
TOV21G
Mutation
ARID1Amt
ARID1Awt

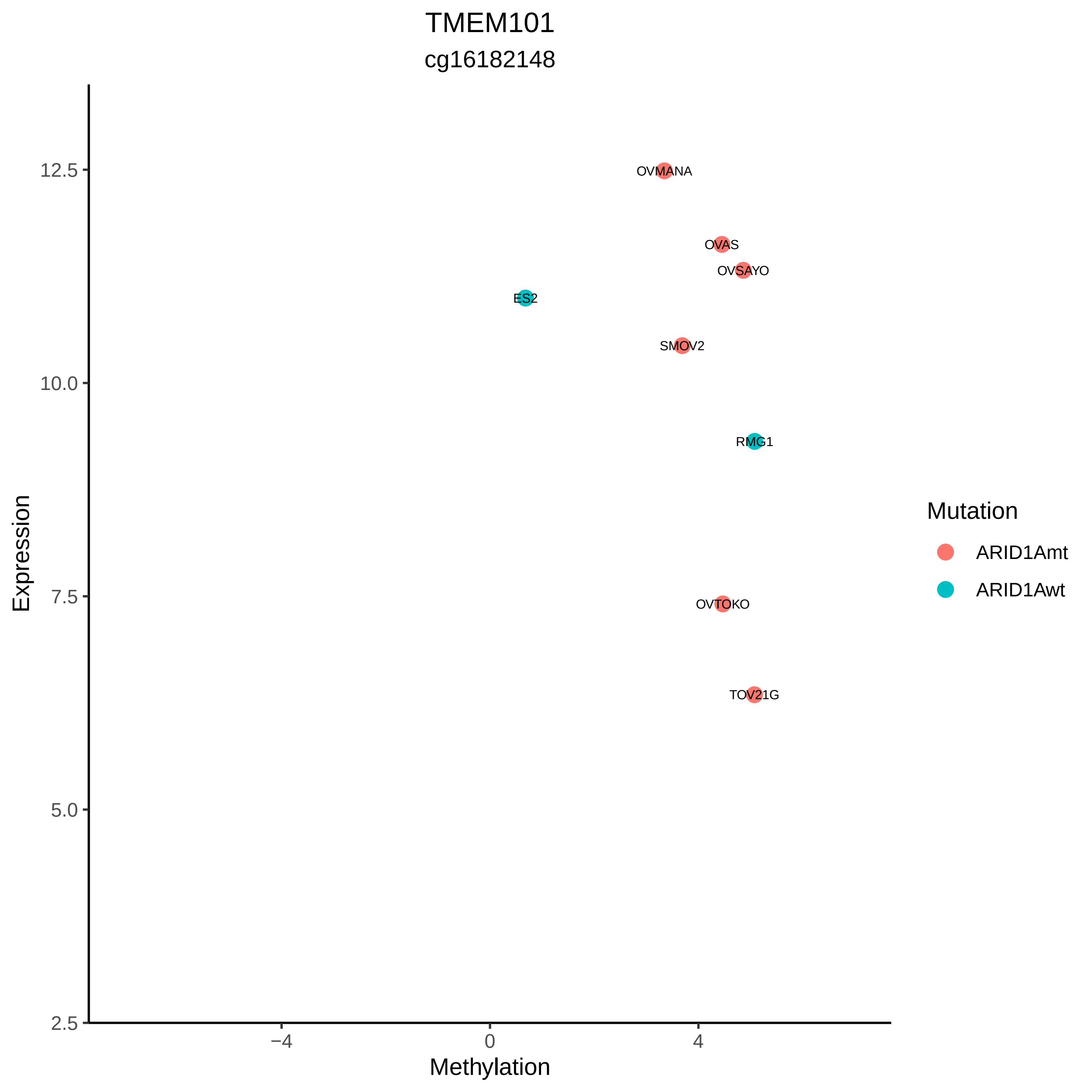

TMEM101
cg16182148
OVMANA
OVAS
OVSAYO
ES2
SMOV2
RMG1
OVTOKO
TOV21G
12.5
10.0
7.5
5.0
2.5
−4
0
4
Expression
Methylation
Mutation
ARID1Amt
ARID1Awt

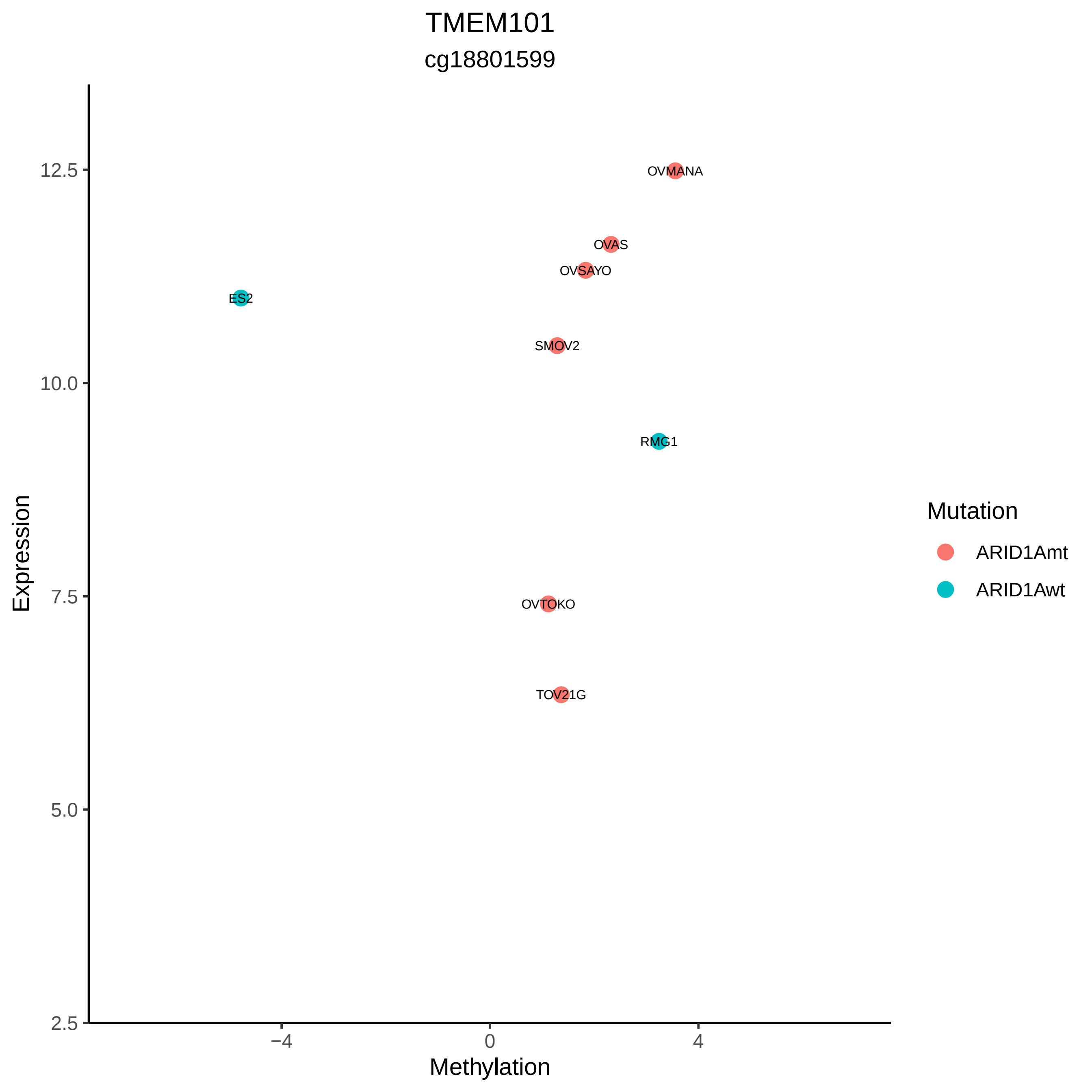

TMEM101
cg18801599
Expression
Methylation
12.5
10.0
7.5
5.0
2.5
−4
0
4
OVMANA
OVAS
OVSAYO
ES2
SMOV2
RMG1
OVTOKO
TOV21G
Mutation
ARID1Amt
ARID1Awt

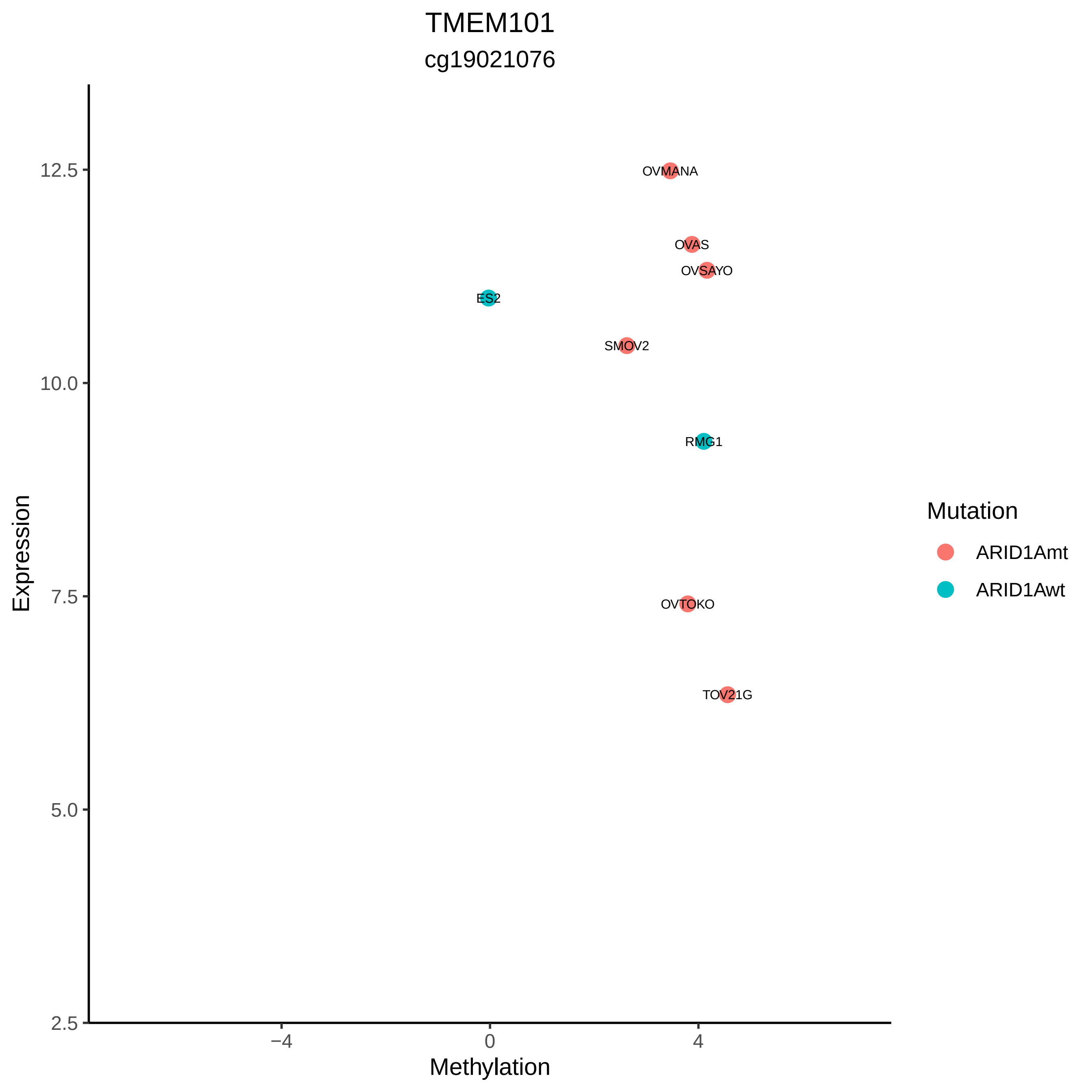
TMEM101
cg19021076
12.5
10.0
7.5
5.0
2.5
Expression
−4
0
4
Methylation
OVMANA
OVAS
OVSAYO
ES2
SMOV2
RMG1
OVTOKO
TOV21G
Mutation
ARID1Amt
ARID1Awt

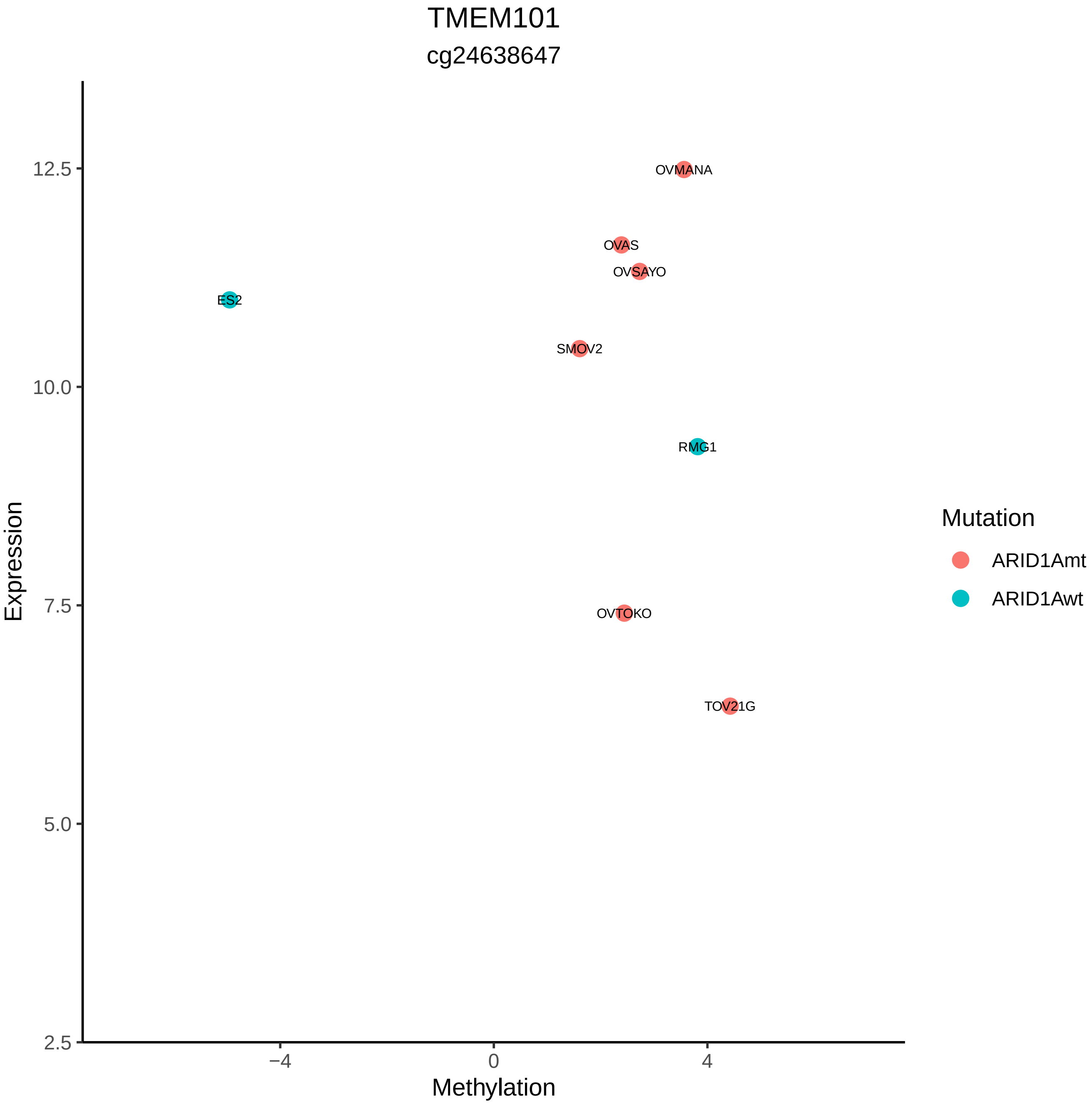
TMEM101
cg24638647
Expression
12.5
10.0
7.5
5.0
2.5
−4
0
4
Methylation
OVMANA
OVAS
OVSAYO
ES2
SMOV2
RMG1
OVTOKO
TOV21G
Mutation
ARID1Amt
ARID1Awt

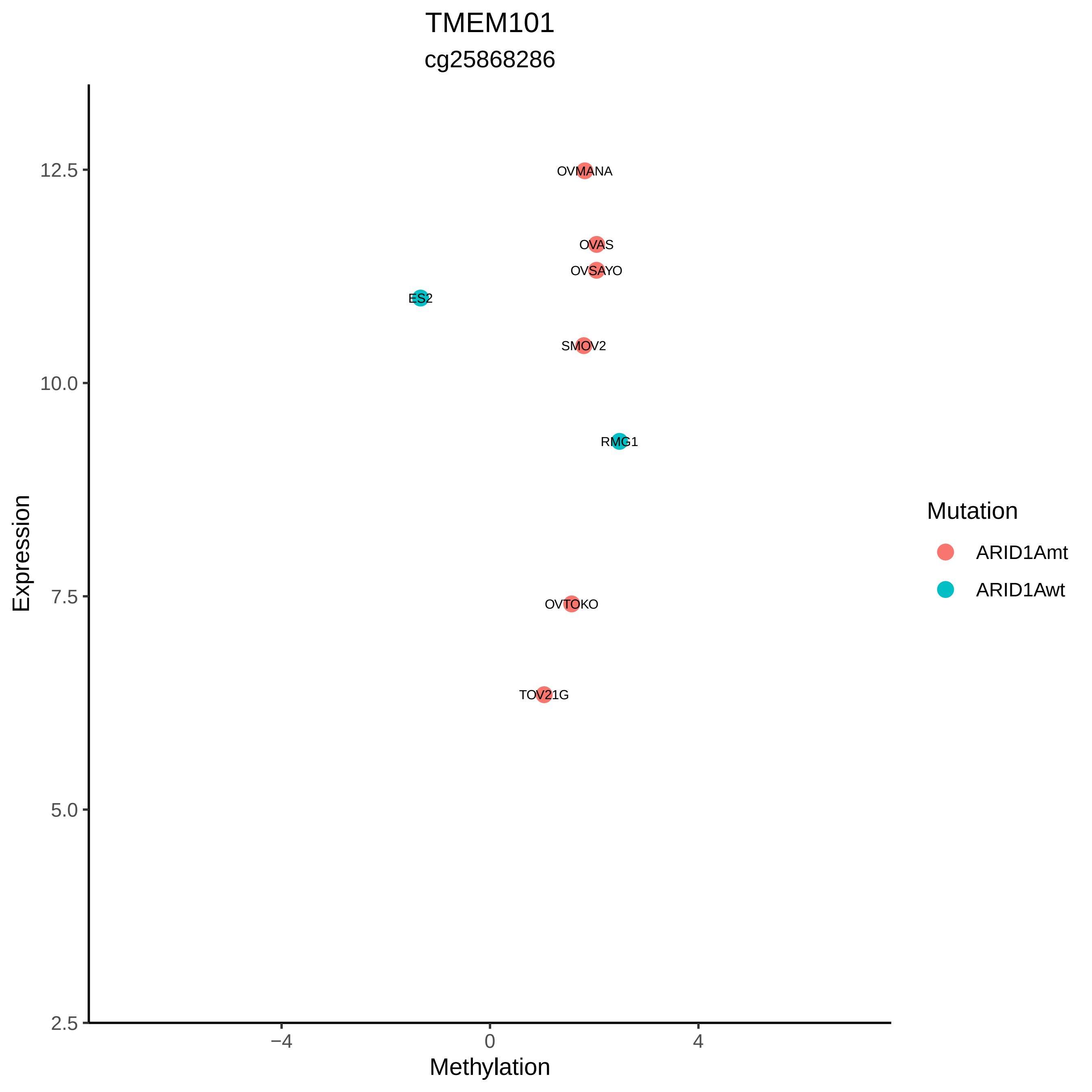
TMEM101
cg25868286
Expression
12.5
10.0
7.5
5.0
2.5
Methylation
−4
0
4
OVMANA
OVAS
OVSAYO
ES2
SMOV2
RMG1
OVTOKO
TOV21G
Mutation
ARID1Amt
ARID1Awt

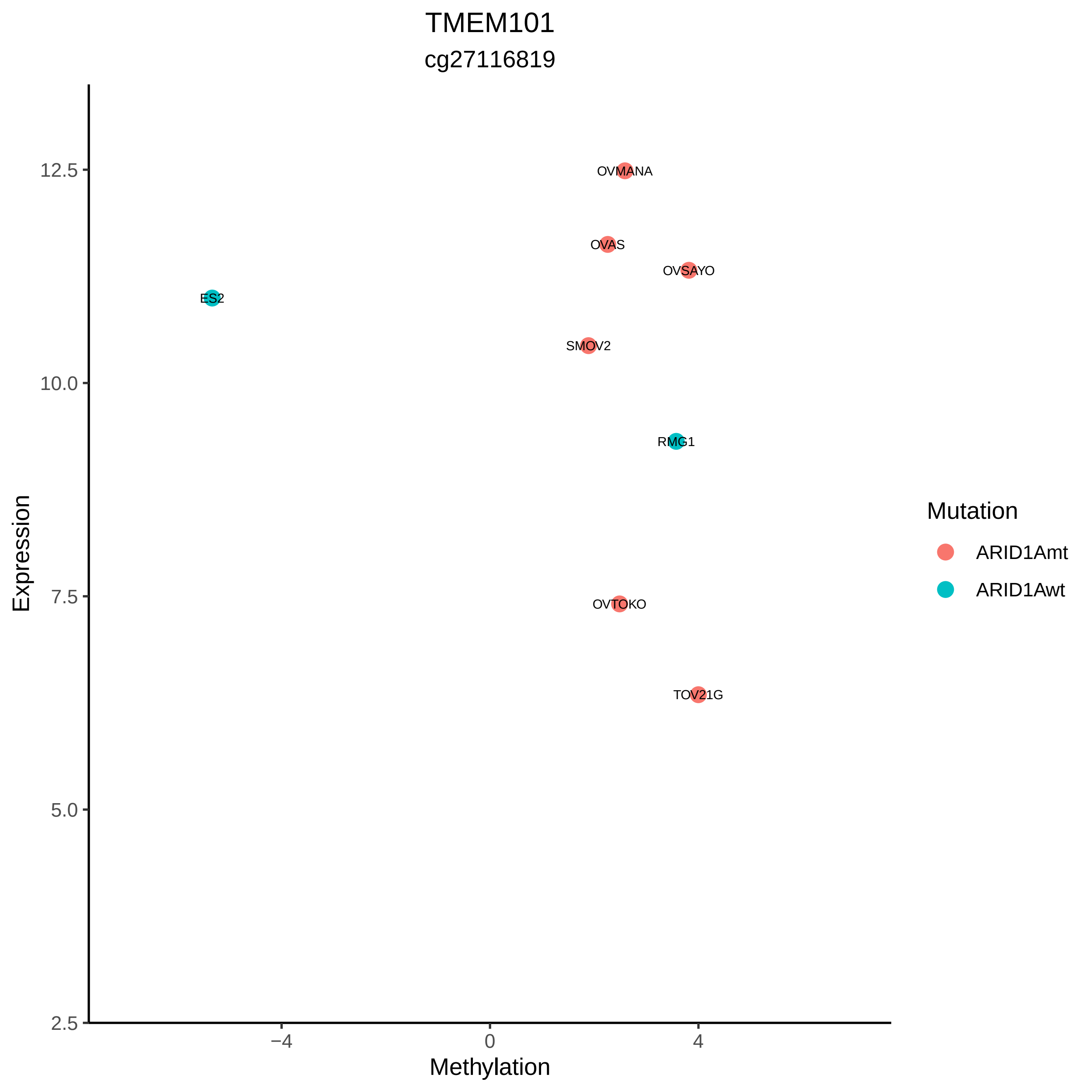

TMEM101
cg27116819
Expression
Methylation
12.5
10.0
7.5
5.0
2.5
−4
0
4
OVMANA
OVAS
OVSAYO
ES2
SMOV2
RMG1
OVTOKO
TOV21G
Mutation
ARID1Amt
ARID1Awt

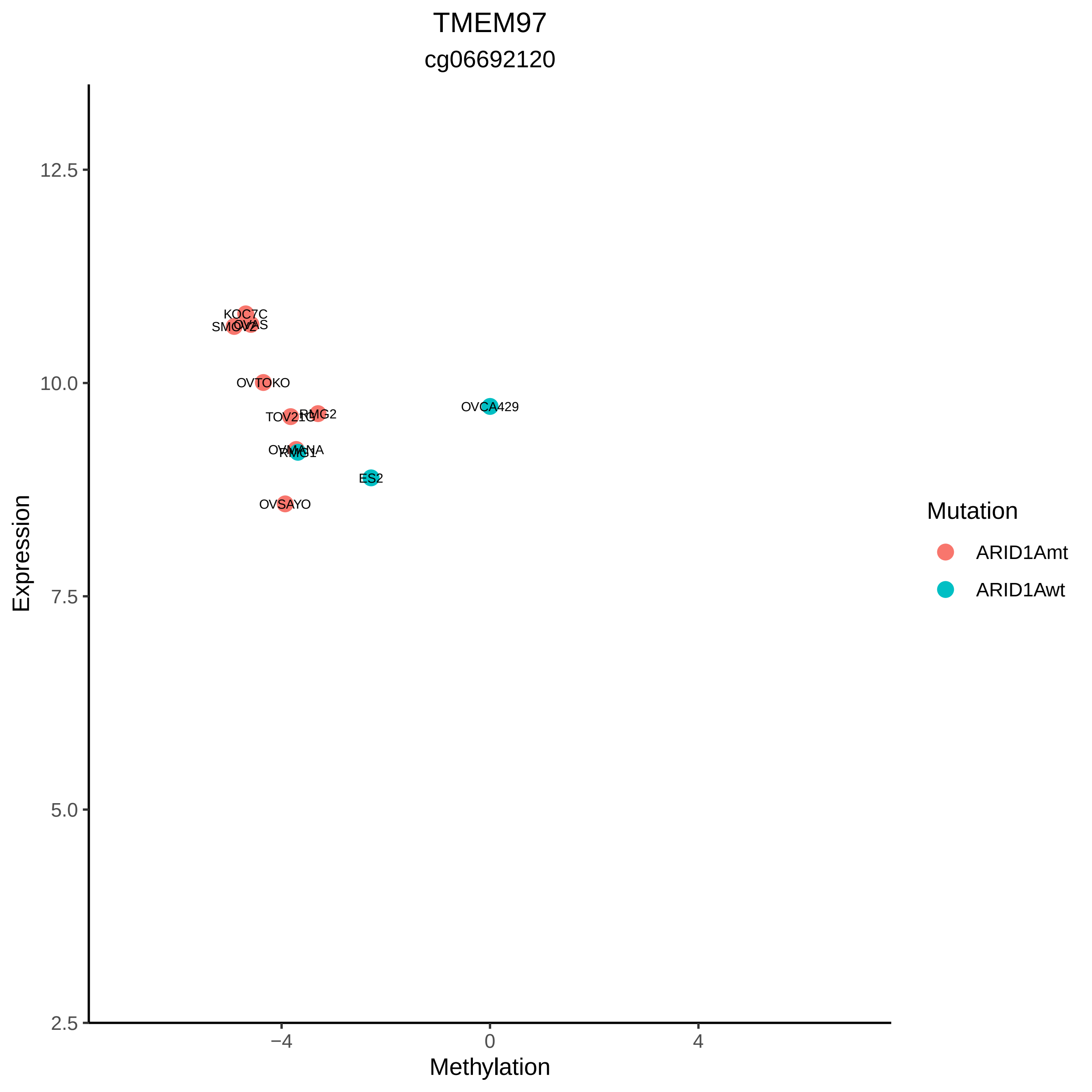

TMEM97
cg06692120
Expression
Methylation
12.5
10.0
7.5
5.0
2.5
−4
0
4
KOC7C
SMOV2
OVISE
OVTOKO
OVCA429
TOV21G
RMG2
OVMANA
RMG1
ES2
OVSAYO
Mutation
ARID1Amt
ARID1Awt

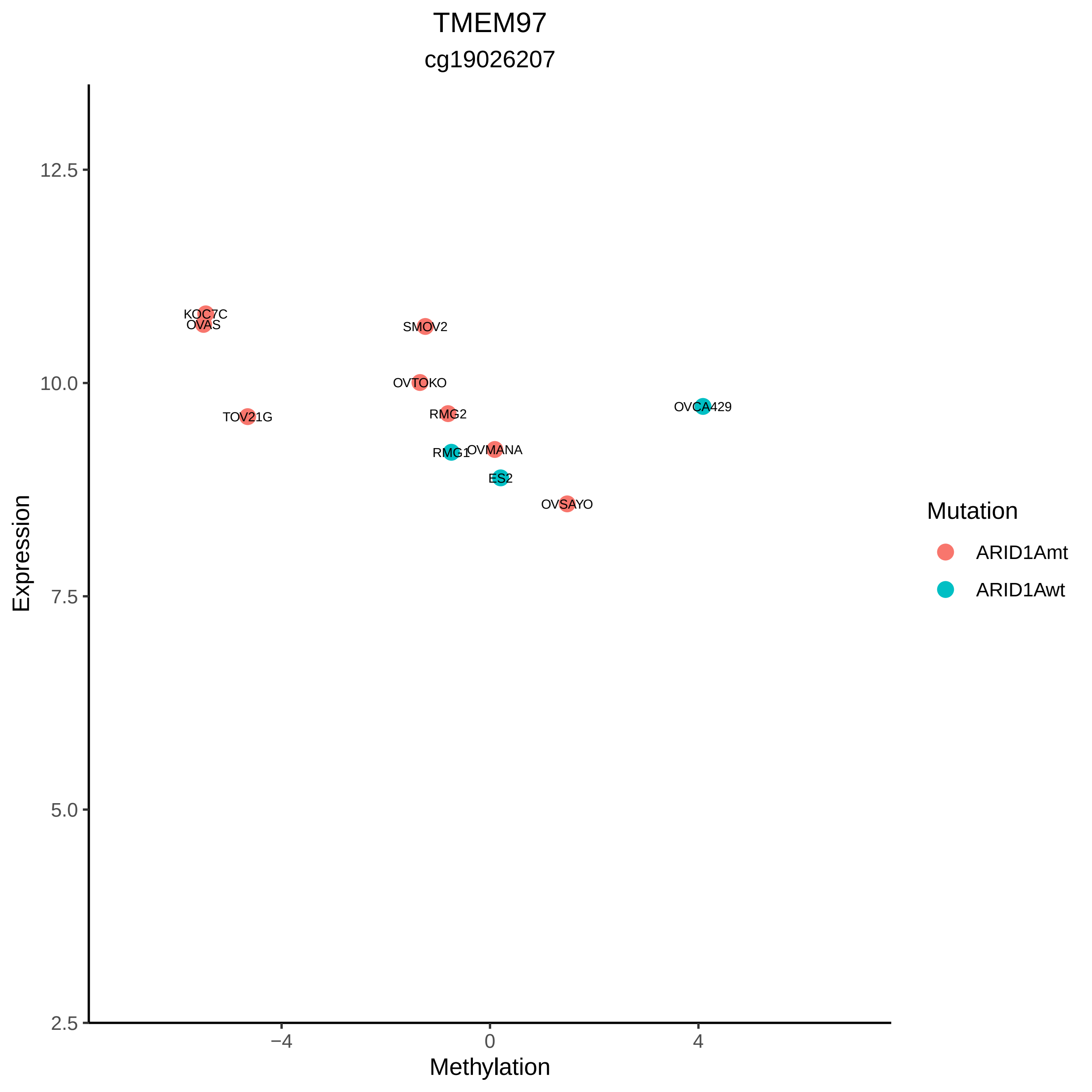
TMEM97
cg19026207
Expression
Methylation
12.5
10.0
7.5
5.0
2.5
−4
0
4
KOC7C
OVAS
SMOV2
OVTOKO
TOV21G
RMG2
OVCA429
RMG1
OVMANA
ES2
OVSAYO
Mutation
ARID1Amt
ARID1Awt

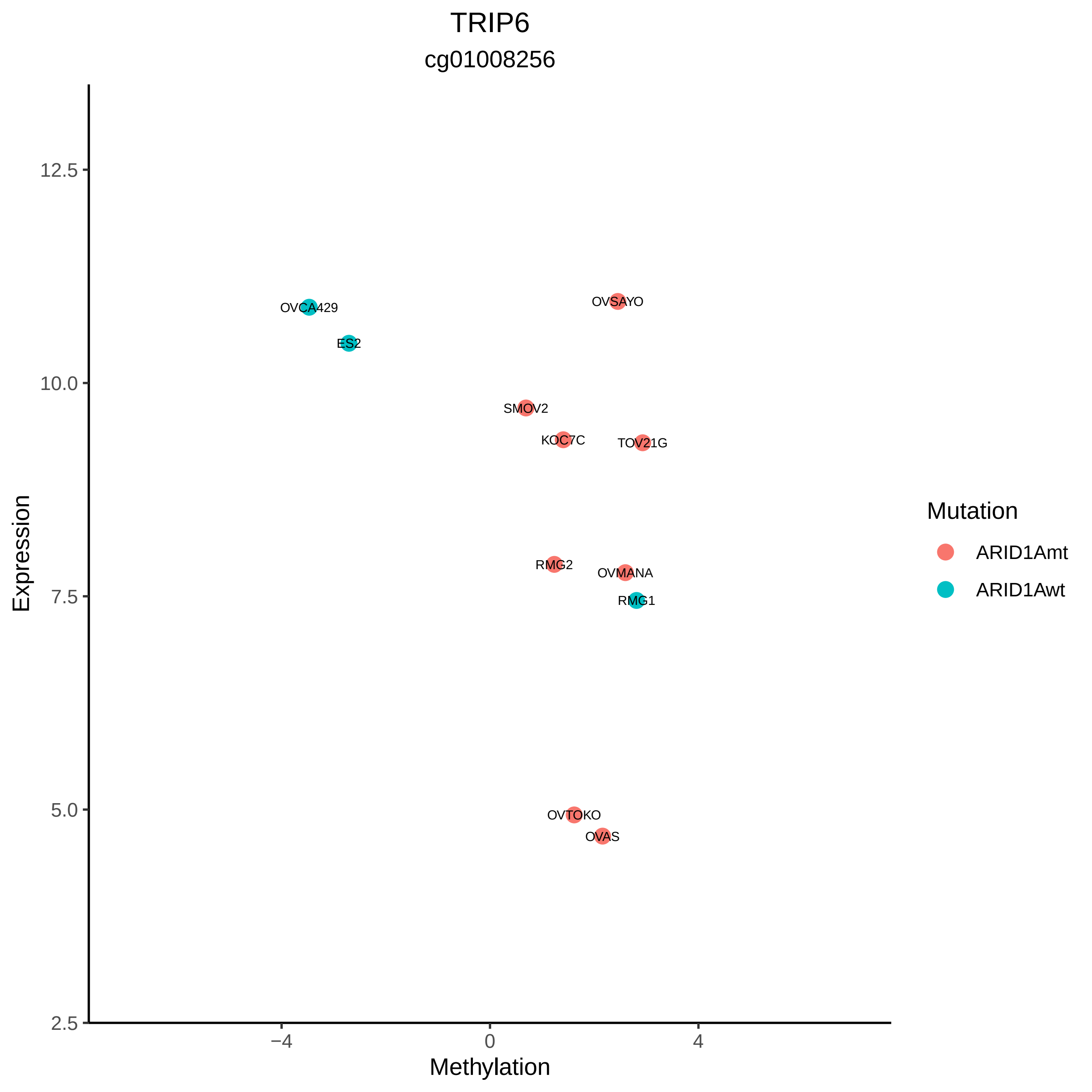
TRIP6
cg01008256
Expression
Methylation
12.5
10.0
7.5
5.0
2.5
−4
0
4
OVCA429
ES2
OVSAYO
SMOV2
KOC7C
TOV21G
RMG2
OVMANA
RMG1
OVTOKO
OVAS
Mutation
ARID1Amt
ARID1Awt

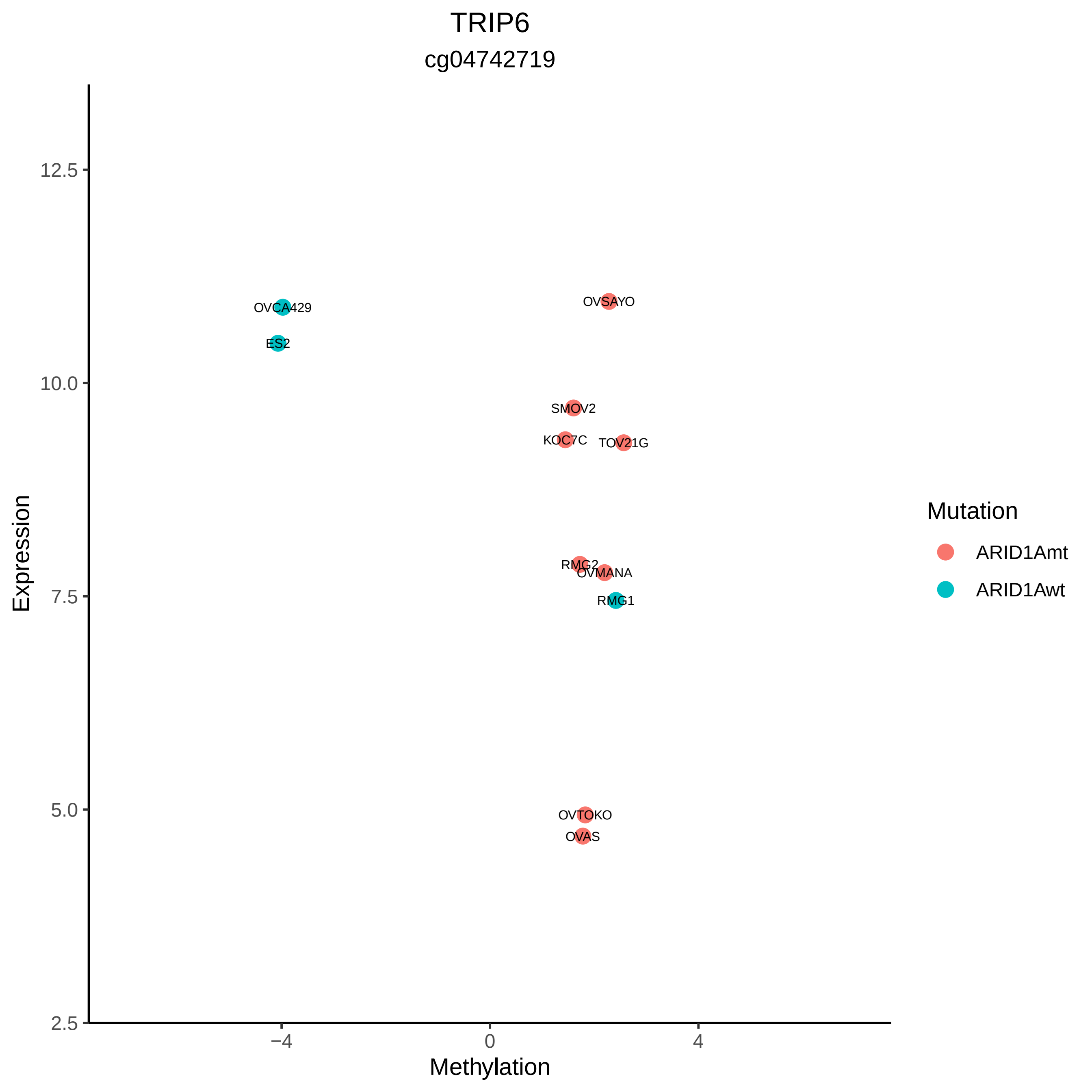

TRIP6
cg04742719
Expression
Methylation
12.5
10.0
7.5
5.0
2.5
−4
0
4
OVCA429
ES2
OVSAYO
SMOV2
KOC7C
TOV21G
RMG2
OVMANA
RMG1
OVTOKO
OVAS
Mutation
ARID1Amt
ARID1Awt

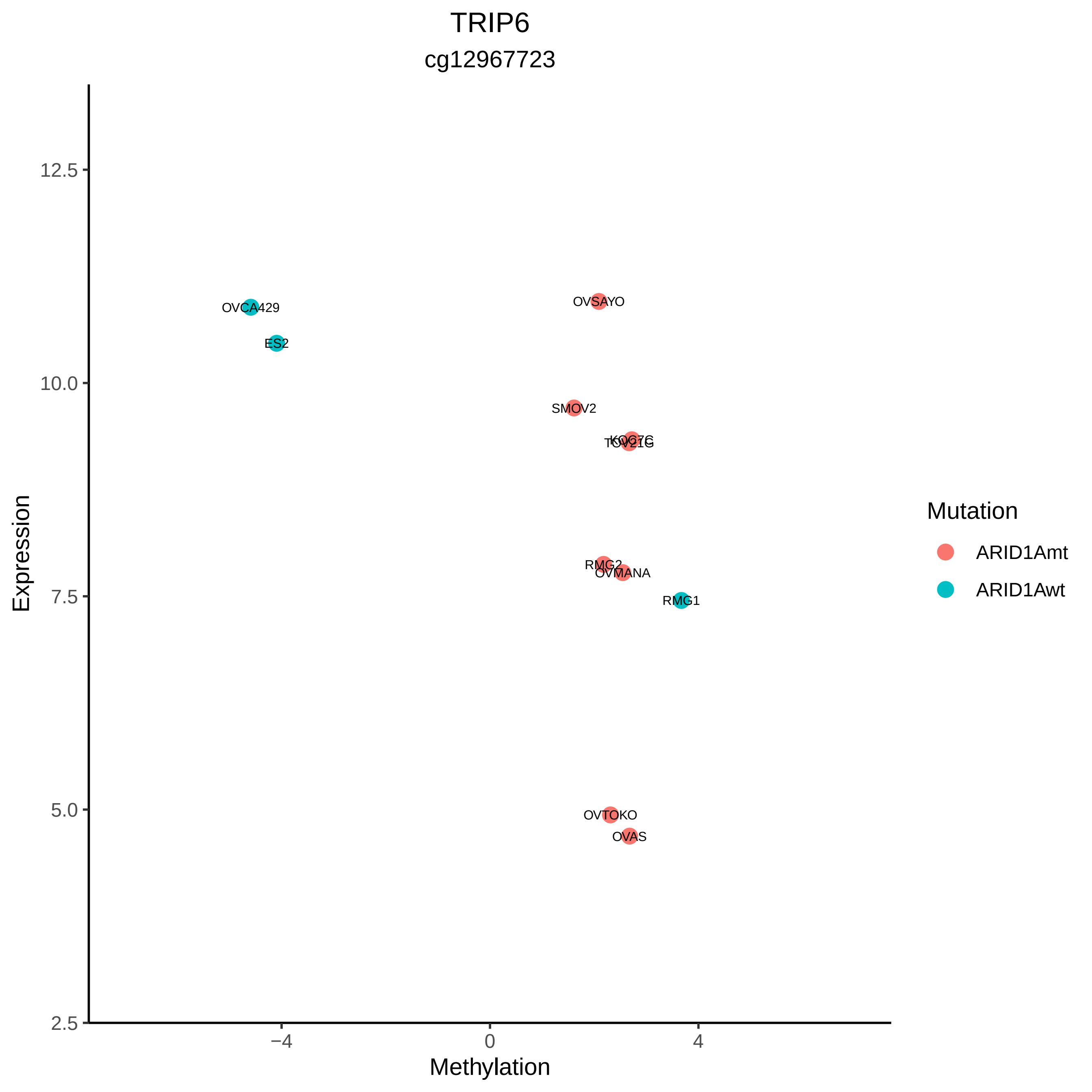

TRIP6
cg12967723
OVCA429
ES2
OVSAYO
SMOV2
KOC7C
TOV21G
RMG2
OVMANA
RMG1
OVTOKO
OVAS
12.5
10.0
7.5
5.0
2.5
−4
0
4
Expression
Methylation
Mutation
ARID1Amt
ARID1Awt

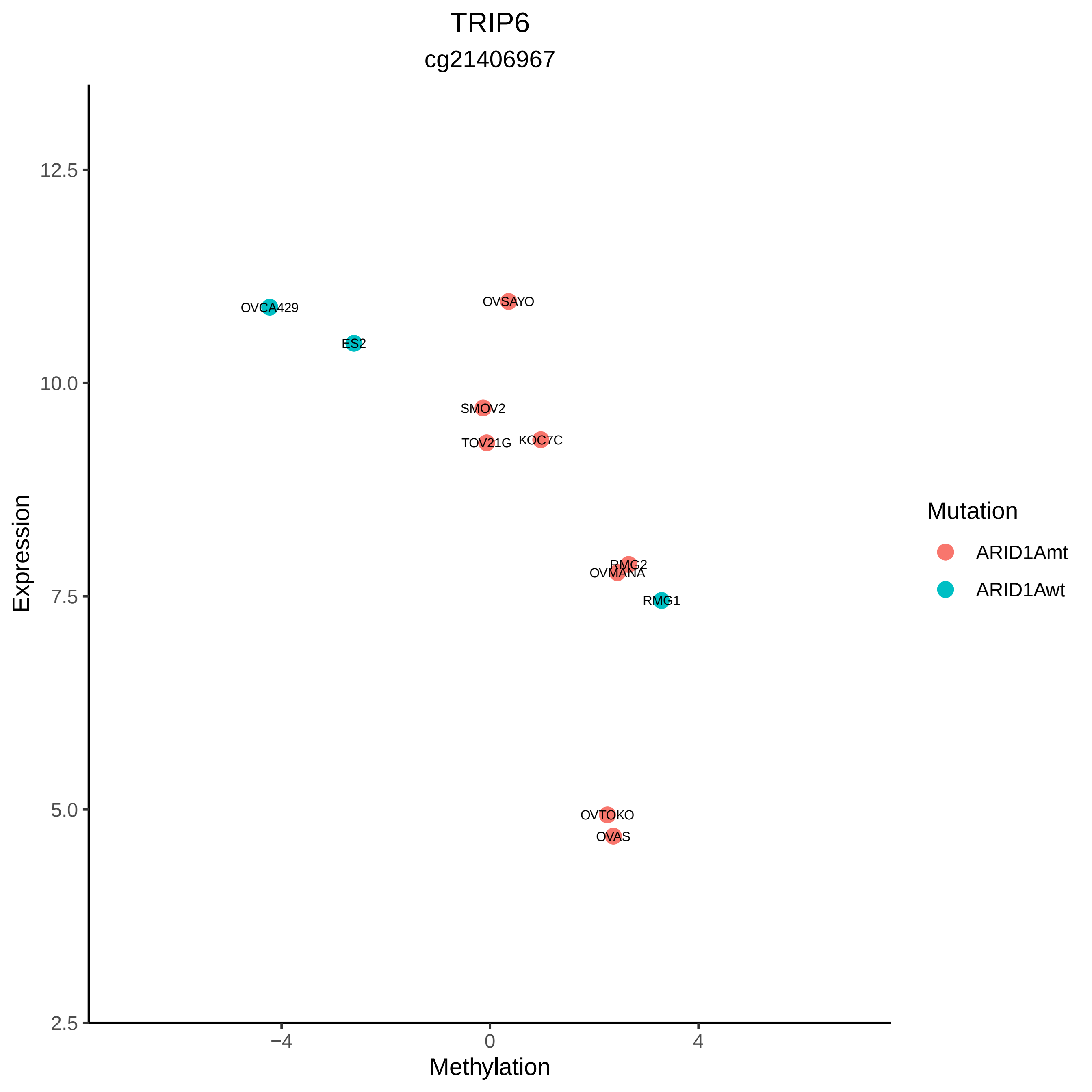
TRIP6
cg21406967
Expression
12.5
10.0
7.5
5.0
2.5
Methylation
−4
0
4
OVCA429
ES2
OVSAYO
SMOV2
TOV21G
KOC7C
RMG2
OVMANA
RMG1
OVTOKO
OVAS
Mutation
ARID1Amt
ARID1Awt

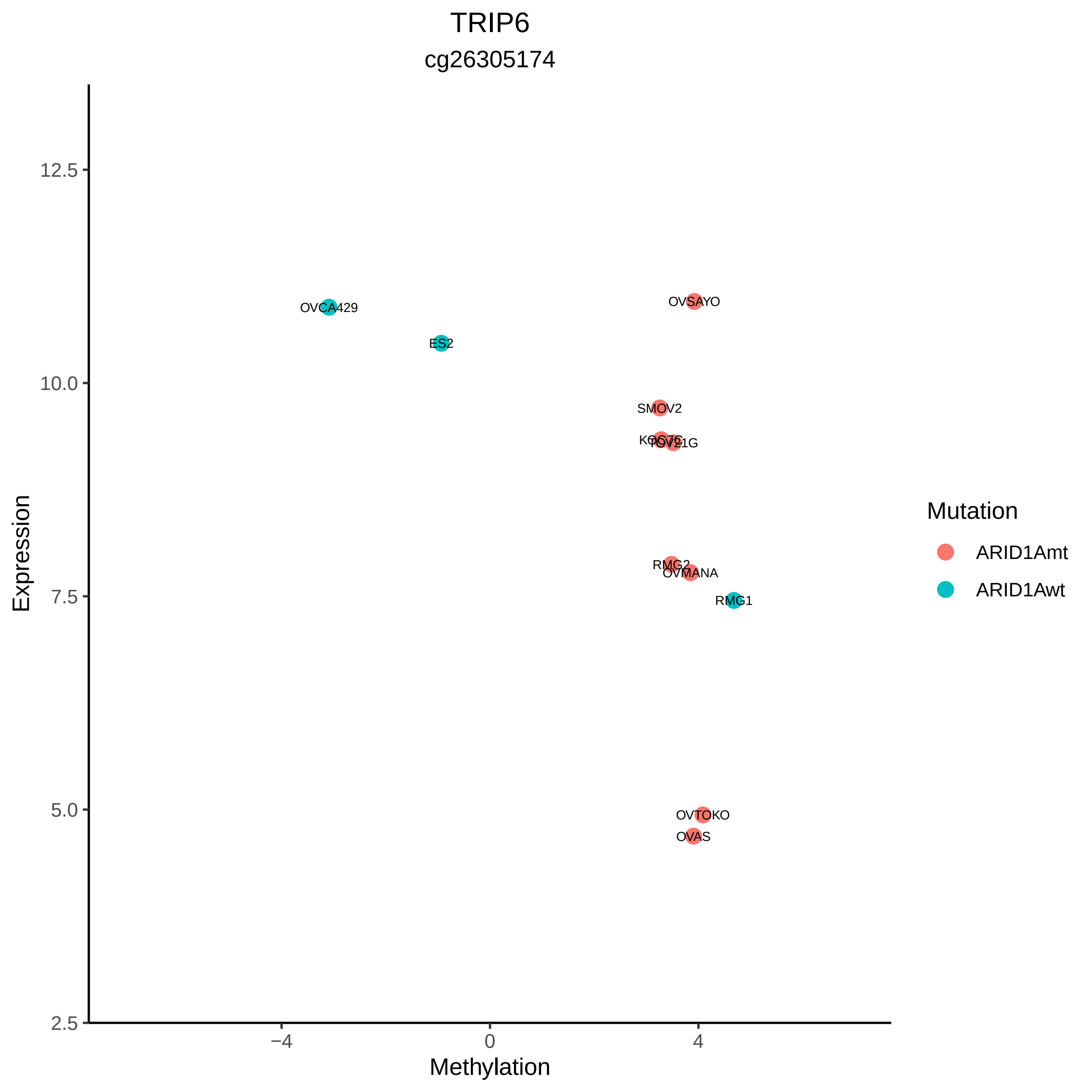

TRIP6
cg26305174
Expression
Methylation
12.5
10.0
7.5
5.0
2.5
−4
0
4
OVCA429
ES2
OVSAYO
SMOV2
KOC7C
TOV21G
RMG2
OVMANA
RMG1
OVTOKO
OVAS
Mutation
ARID1Amt
ARID1Awt

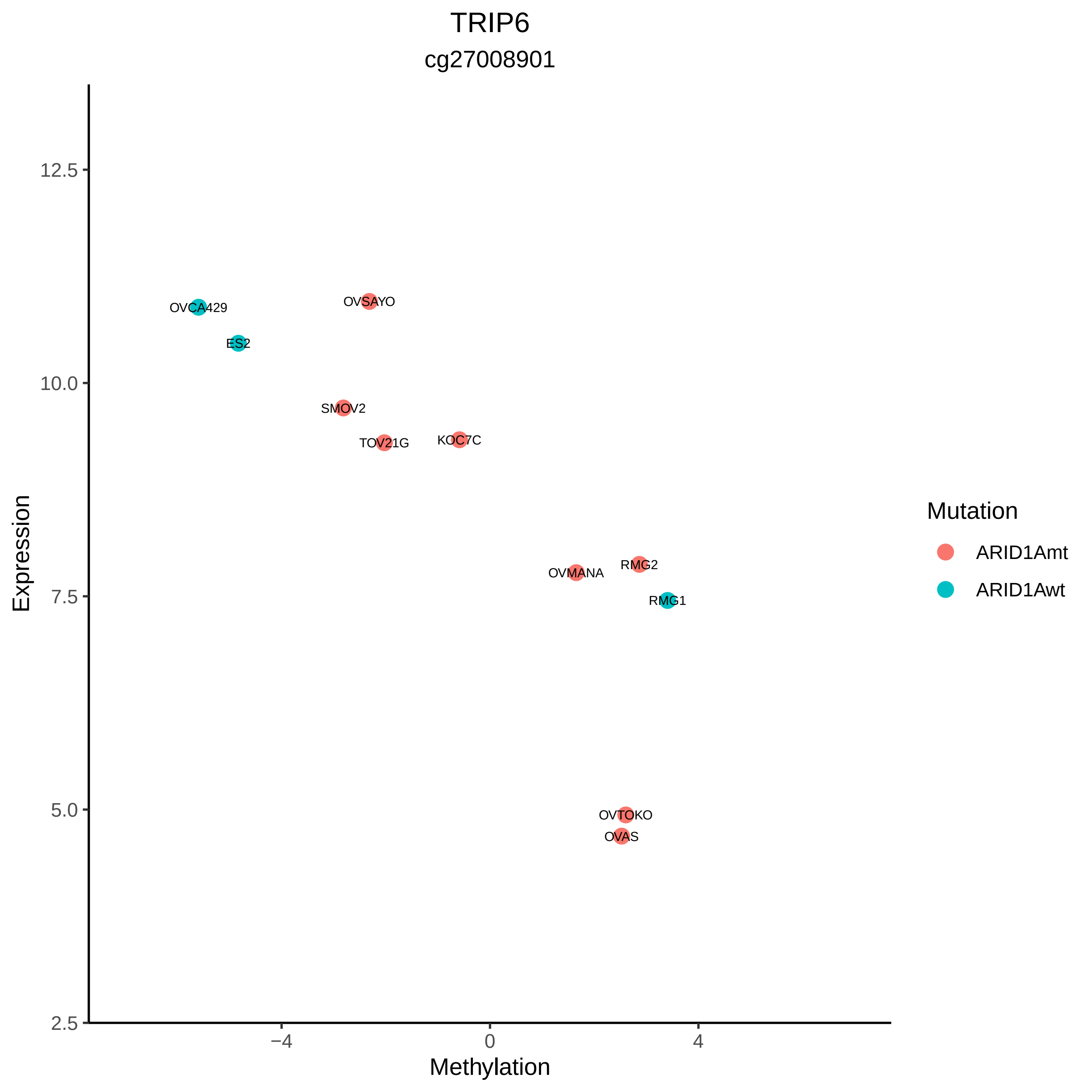
TRIP6
cg27008901
OVCA429
OVSAYO
ES2
SMOV2
TOV21G
KOC7C
OVMANA
RMG2
RMG1
OVTOKO
OVAS
12.5
10.0
7.5
5.0
2.5
−4
0
4
Expression
Methylation
Mutation
ARID1Amt
ARID1Awt

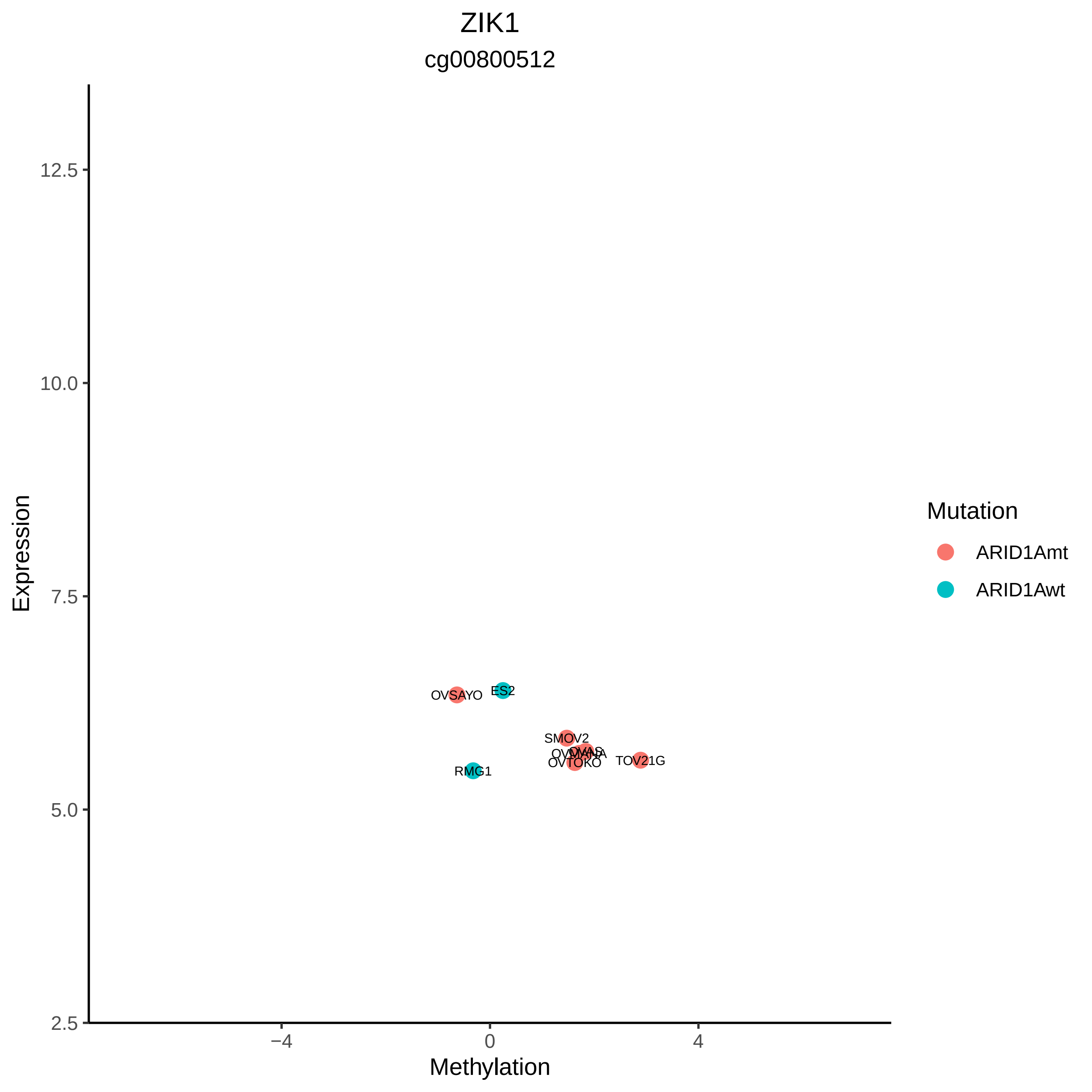
ZIK1
cg00800512
Expression
12.5
10.0
7.5
5.0
2.5
Methylation
−4
0
4
OVSAYO
ES2
SMOV2
TOV21G
OVTOKO
RMG1
Mutation
ARID1Amt
ARID1Awt

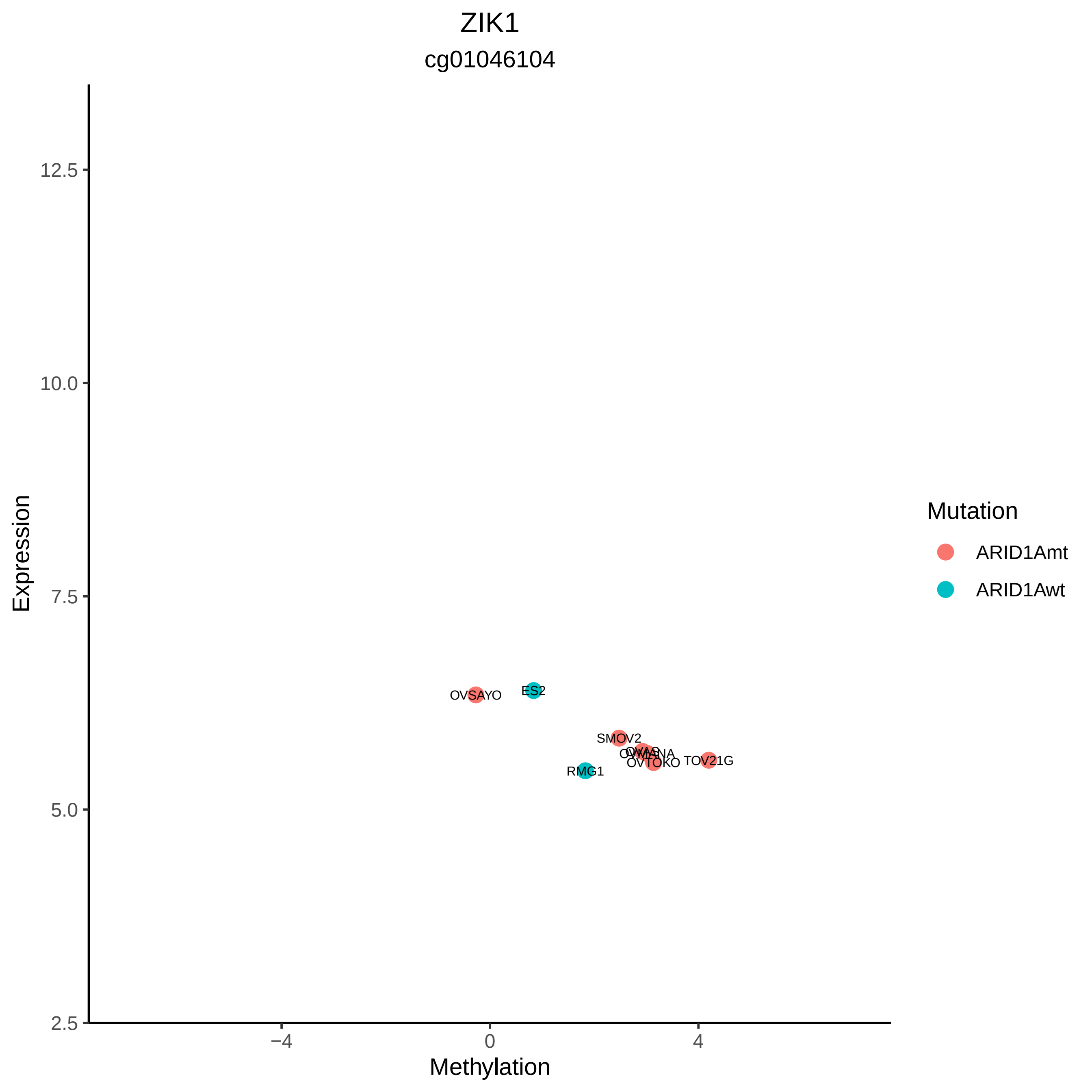
ZIK1
cg01046104
Expression
Methylation
12.5
10.0
7.5
5.0
2.5
−4
0
4
OVSAYO
ES2
SMOV2
OVTOKO
TOV21G
RMG1
Mutation
ARID1Amt
ARID1Awt

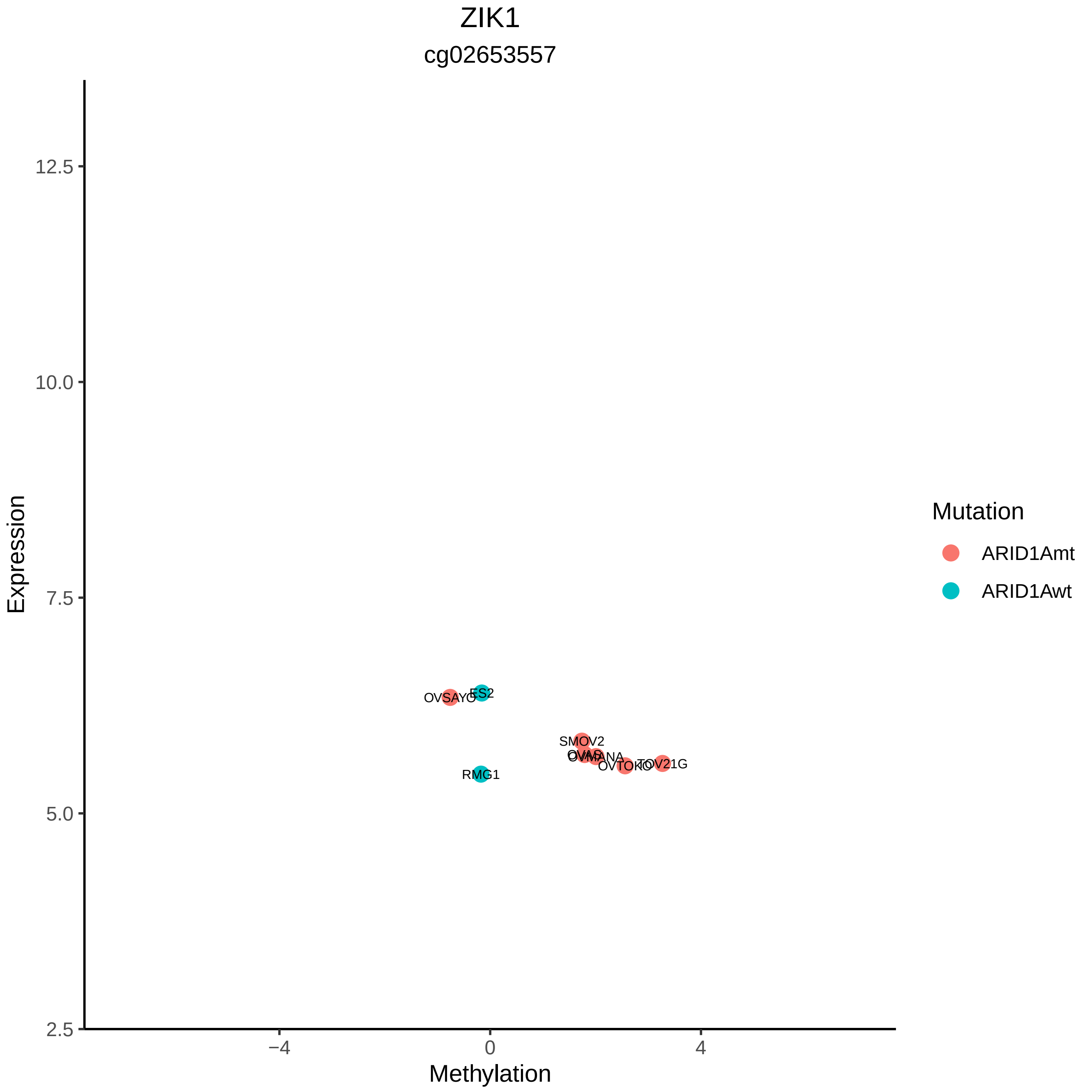
ZIK1
cg02653557
Expression
Methylation
12.5
10.0
7.5
5.0
2.5
−4
0
4
OVSAYO
ES2
SMOV2
OVMANA
OVTOKO
TOV21G
RMG1
Mutation
ARID1Amt
ARID1Awt

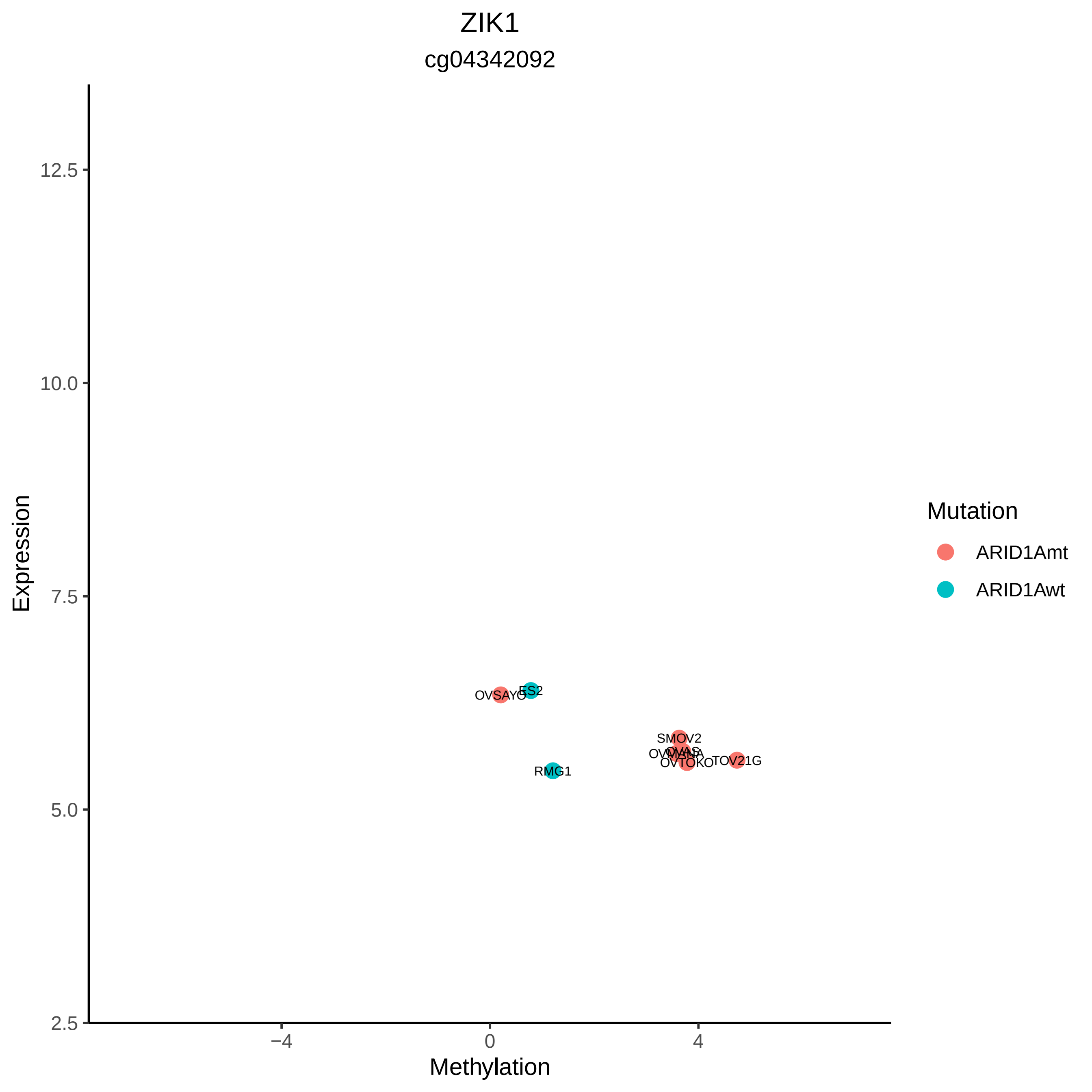
ZIK1
cg04342092
Expression
Methylation
12.5
10.0
7.5
5.0
2.5
−4
0
4
OVSAYO
ES2
SMOV2
OVMANA
OVTOKO
TOV21G
RMG1
Mutation
ARID1Amt
ARID1Awt

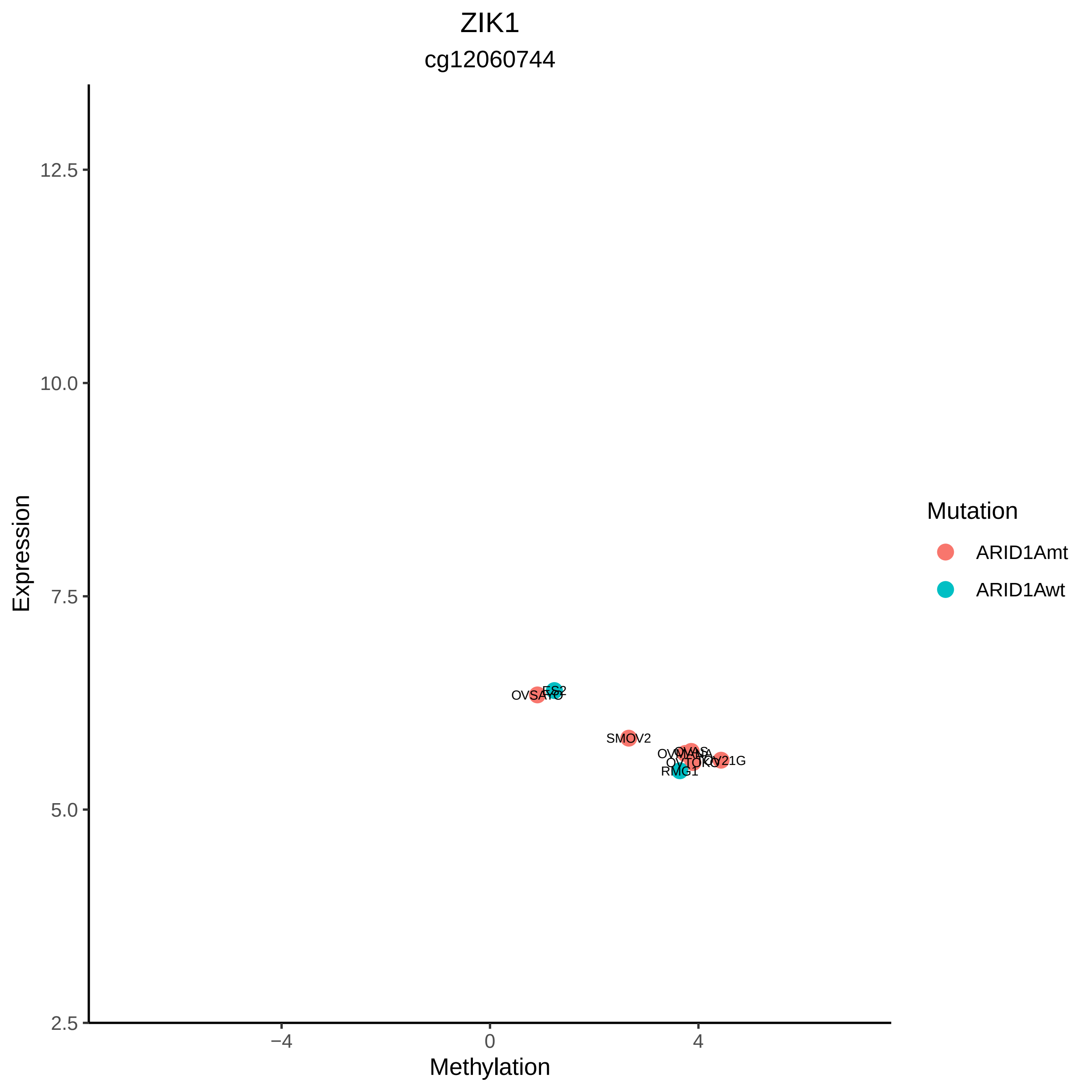

ZIK1
cg12060744
Expression
12.5
10.0
7.5
5.0
2.5
−4
0
4
Methylation
ES2
OVSAYO
SMOV2
RMG1
Mutation
ARID1Amt
ARID1Awt

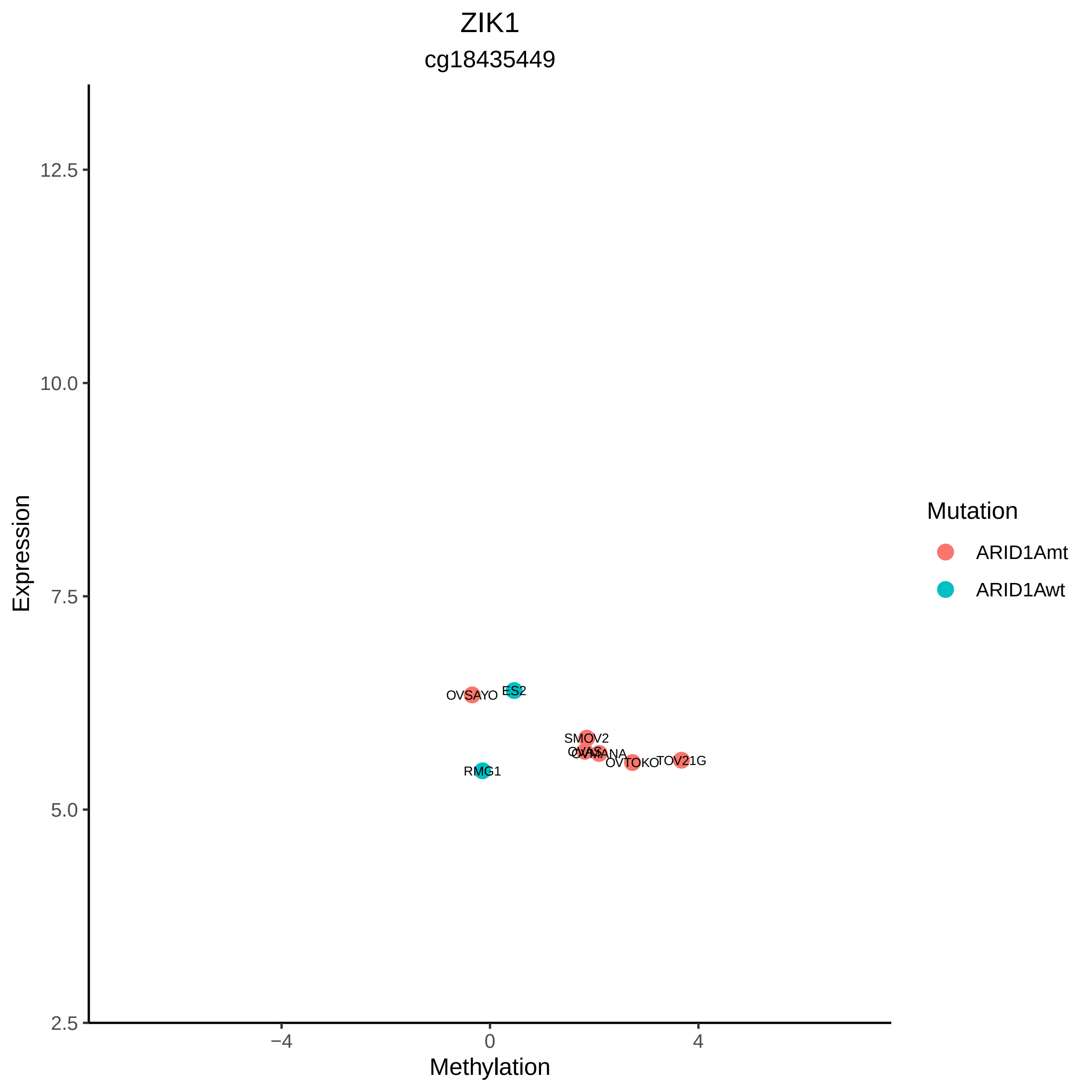
ZIK1
cg18435449
Expression
Methylation
12.5
10.0
7.5
5.0
2.5
−4
0
4
OVSAYO
ES2
SMOV2
OVAS
OVMANA
OVTOKO
TOV21G
RMG1
Mutation
ARID1Amt
ARID1Awt

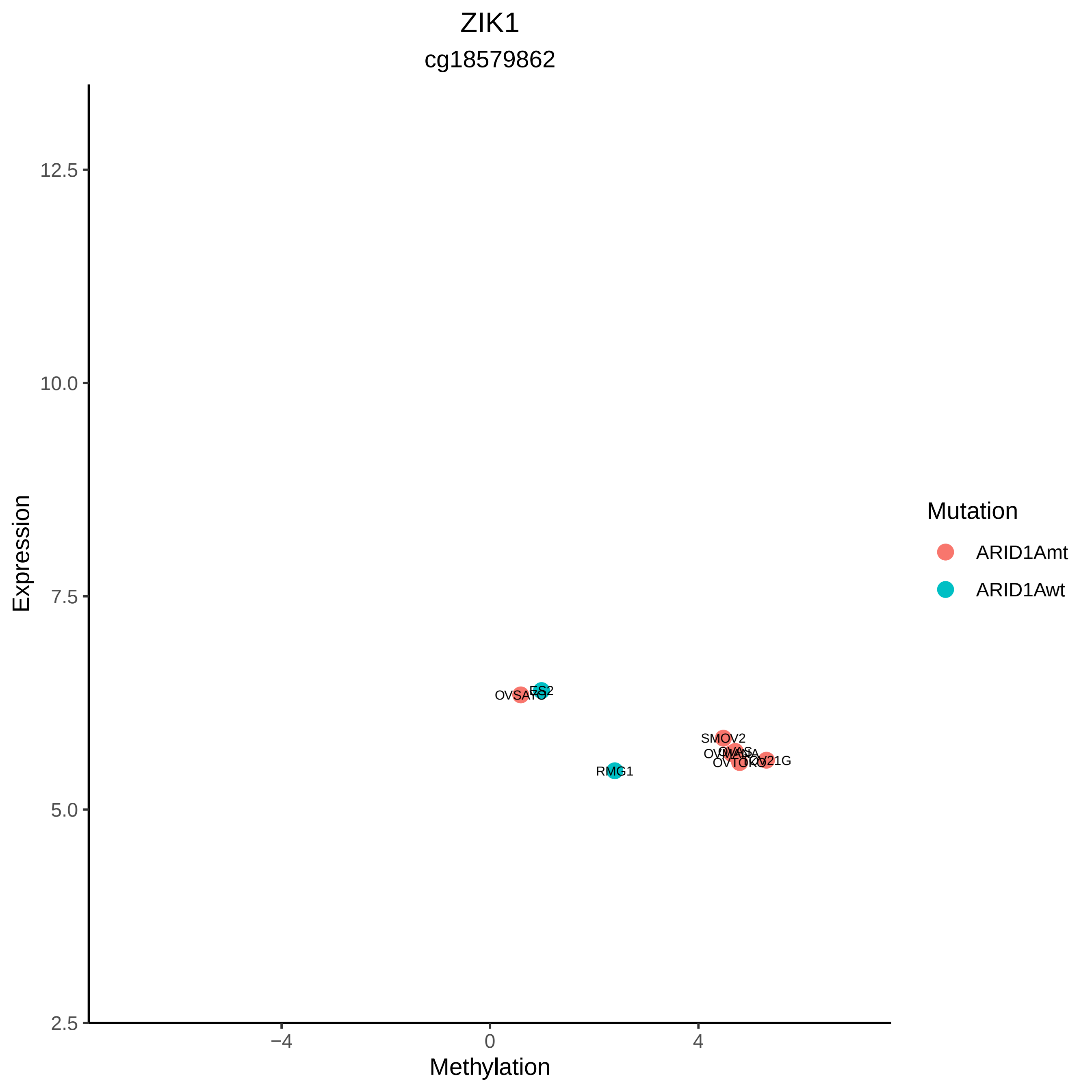

ZIK1
cg18579862
Expression
Methylation
12.5
10.0
7.5
5.0
2.5
−4
0
4
OVSAYO
ES2
RMG1
SMOV2
OVTOKO
Mutation
ARID1Amt
ARID1Awt

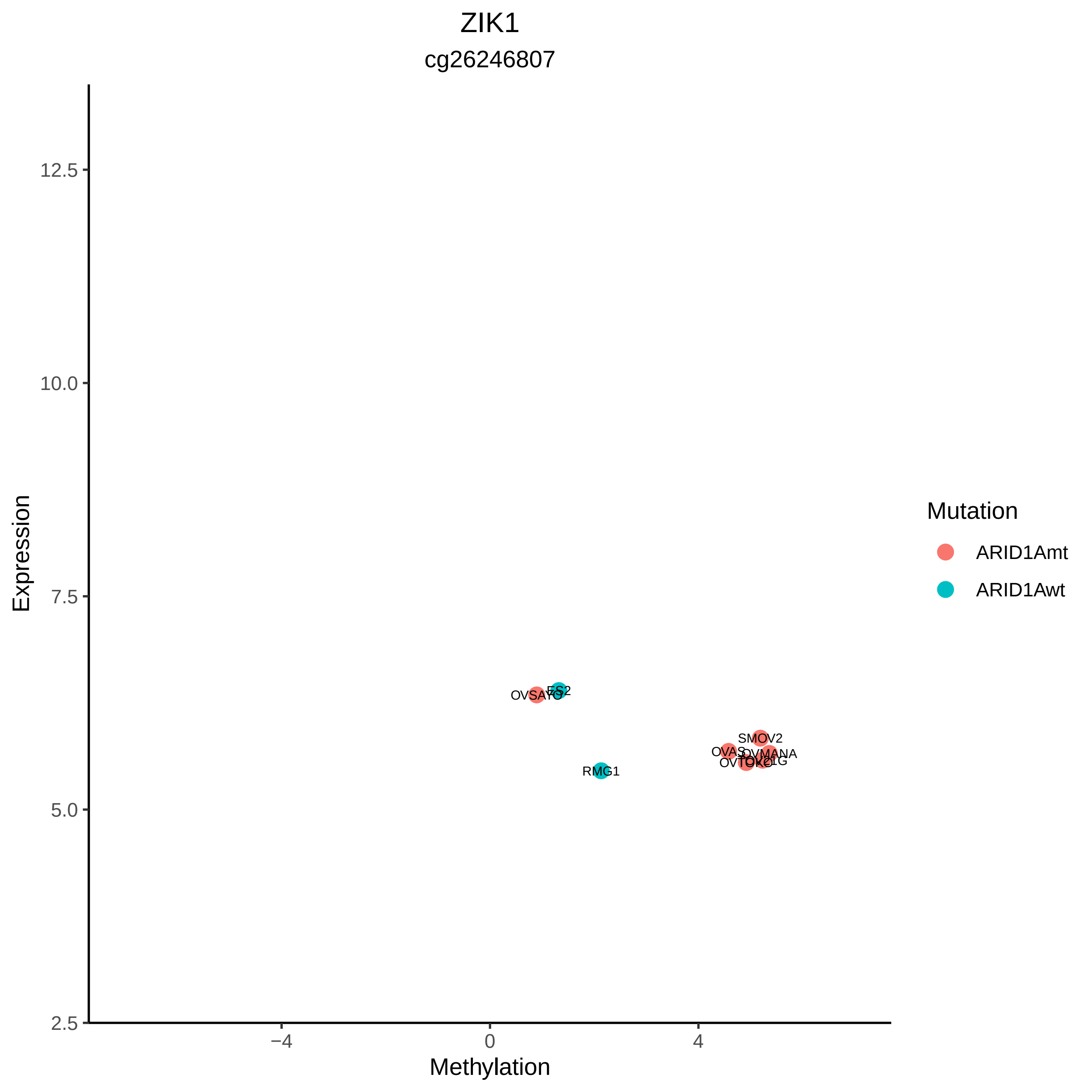
ZIK1
cg26246807
Expression
Methylation
12.5
10.0
7.5
5.0
2.5
−4
0
4
OVSAYO
ES2
RMG1
SMOV2
OVAS
OVMANA
OVTOKO
TOV21G
Mutation
ARID1Amt
ARID1Awt
