# Supplementary material for: Genome-wide DNA methylation in relation to ARID1A deficiency in ovarian clear cell carcinoma
Source: J Transl Med. 2024 Jun 10;22:556. doi: 10.1186/s12967-024-05311-7 (PMC11163774; doi:10.1186/s12967-024-05311-7)
Supplement: Supplementary file 2 — Supplementary material 2. [file 12967_2024_5311_MOESM2_ESM.docx]

**Supplementary methods**

**Data processing of Infinium MethylationEPIC BeadChip arrays**

R package "minfi" was used to process raw IDAT data from Infinium MethylationEPIC BeadChip arrays [1]. Raw iDAT files were imported and processed as described in Bioconductor [2]. Dye bias correction was done via "singleNoob" method of "minfi" package. Normalization was also done according to Bioconductor. Next, as described in "minfi" package and before [3], the methylated intensity (denoted by ***M***) and the unmethylated intensity (denoted by ***U***) of each CpG site were used to determine its β-value (β=***M***/(***M***+***U***+100) and M-value (M-value=log(***M***/***U***)), both representing the methylation level of the CpG site.

Poor performing CpG probes including (1) probes that failed in one or more samples (n=13,224), (2) probes that were potentially cross-hybridizing or overlapping genetic variants (n=151,690) [4,5], and (3) probes with unspecific alignment to human genome (n=7,951) were removed from the analysis [6]. Finally, M-values and β-values of 692,994 CpG sites were obtained. For the duplicated samples (ES2, ES2*^ARID1A-/-^*, OVCA429 and OVCA429*^ARID1A-/-^*), average β-value and M-values were calculated.

**Hierarchical clustering**

To analyze the hierarchical clustering, first, Spearman correlation coefficients (Spearman coefficients) matrix was generated using β-values of all 692,994 CpGs sites in OCCC tumor and cell lines. Next, based on the Spearman coefficients matrix, Euclidean distance between each two samples was determined. At last, using Euclidean distance, OCCC samples were unsupervised hierarchically clustered using “complete” method as described in "pheatmap" R package [7]. Fisher exact test was used to determine the significance of clinical features and genetic mutations in relation to the clustering. Multiple linear regression test was done using “arm” R package [8].

**Identification of differential methylated (DM) CpGs located in gene promoters or gene-bodies**

OCCC samples were classified into 4 sample sets (“OCCC tumor”, “OCCC cell lines”, “ES2*^ARID1A-/-^* and ES2” and “OVCA429*^ARID1A-/-^* and OVCA429”). Since β-value and M-value of CpGs both have their own advantages in discovering differential methylated (DM) CpGs [9], 3 cut-offs based on β-value and M-value were used collectively to identify DM CpGs (Supplementary Table 3).

Cut-off 1 and Cut-off 2 were both based on M-value, Methylation log_2_FC and differential methylation significance (p-value) of each CpG between *ARID1A*mt or *ARID1A*ko samples and *ARID1A*wt samples were calculated using “limma” package [10].

Cut-off 3 was based on absolute Δβ and was calculated as followed:

absolute Δ =| - |,


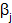

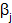

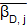

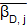

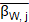

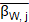


where represented the average of *ARID1A*mt/ko samples at j^th^ CpG site and represented the average of *ARID1A*wt samples at j^th^ CpG site.


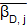

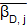

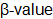

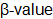

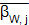

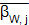

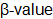

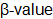


For each sample set, CpGs that satisfied at least two out of three corresponding cut-offs were identified as DM CpGs. Overlapping DM CpGs derived from all sample sets were visualized by Venn diagram using R package “VennDiagram” [11]. Based on the Venn diagram, common DM CpGs present in at least two sample sets were identified and used for subsequent analysis. DM CpGs that gained methylation in two sample sets but lost methylation in the other two sample sets were regarded as ambiguous DM CpGs and were therefore removed from the list. At last, the common DM CpGs without ambiguity were further annotated to gene transcripts, based on “Infinium MethylationEPIC v1.0 B4 Manifest“. Considering methylation of CGIs that are located in promoter (TSS200, TSS1500, 1stExon and 5’UTR) and gene-body regions may closely relate to gene expression [12], DM CpGs located in promoter or gene-body CGIs were selected for further analysis.

**Expression of genes with DM CpGs located in their promoters or gene-bodies**

Two OCCC datasets from GEO [13], GSE16570 (10 OCCC cell lines: ES2, KK, OVAS, OVISE, OVMANA, OVSAYO, OVTOKO, RMG1, SMOV2 and TOV21G) and GSE29175 (14 OCCC cell lines: OVCA429, JHOC5, JHOC7, JHOC8, JHOC9, KOC5C, KOC7C, OVISE, OVTOKO, RMG1, RMG2, RMG5, TAYA and TOV21G) [14,15] were used to identify genes whose expression may be epigenetically regulated. First, logit transformed expression data from these two datasets was combined into one file (24 samples of which RMG1, OVISE, OVTOKO and TOV21G were present in both datasets) using R base function “merge”. Genes tested in either of the datasets were included in the output of merge function (all=TRUE). However, a batch effect mainly due to different sources of samples was detected. To preserve characters relating to *ARID1A*mt, during correction, information relating to *ARID1A*mt status of all 24 OCCC cell lines was integrated into a design matrix. Next, the design matrix, combined expression file and the batch vector that indicating the source of each sample were submitted to “removeBatchEffect” function of “limma” package. After batch effect correction (Supplementary Figure 2), average expression was taken for the duplicate samples (RMG1, OVISE, OVTOKO and TOV21G). Thereby, a batch effect corrected logit transformed expression profile containing 20 OCCC cell lines was obtained, of which 11 were also analyzed in our study by MethylationEPIC BeadChip arrays.

Based on the batch effect corrected GEO data and Infinium MethylationEPIC BeadChip arrays of these 11 cell lines, the correlation between DNA methylation of all common DM CpGs located in CGIs of promoter and of gene-body and expression of corresponding genes were further analyzed. First, methylation log_2_FC (11 cell lines) of each DM CpG between *ARID1A*mt samples versus *ARID1A*wt samples was calculated using “limma” package [10]. Next, expression log_2_FC (GEO, 11 cell lines) of each gene between *ARID1A*mt vs *ARID1A*wt OCCC was calculated with "limma" method [10]. The positive expression log_2_FC indicated the upregulated expression of this gene in *ARID1A*mt OCCC. The negative expression log_2_FC indicated the downregulated expression of this gene in *ARID1A*mt OCCC. At last, Spearman coefficients between M-value of DM CpGs and expression of their targeted genes (M-E Spearman coefficients (11 cell lines)) were determined.

**Pre-ranked gene set enrichment analysis (pre-ranked GSEA) of genes**

Pre-ranked GSEA requires a list of genes, where genes are sorted based on user-supplied rank-scores. DM CpGs that were commonly identified in at least 2 out of “OCCC tumor”, “OCCC cell lines”, “ES2*^ARID1A-/-^* and ES2” and “OVCA429 *^ARID1A-/-^* and OVCA429” sample sets (n of DM CpGs=3627), and their target genes (n of genes=2004, Supplementary Table 4) were analyzed. We did three separate analysis: methylation based pre-ranked GSEA, expression based pre-ranked GSEA and M-E Spearman coefficients based pre-ranked GSEA. Rank-scores to GSEA software for each pre-ranked GSEA, were calculated based on the following steps:

1. Let *i* =1, …, ***I*** denote genes included in the analysis.
2. Let CpG_1_, …, CpG_j_ represent the DM CpGs located in the promoter or gene-body CGIs of gene *i*.
3. Let CpG_k_ (k∈{*1, …, j*}) represent the k^th^ DM CpG which had the largest absolute methylation log_2_FC (11 cell lines) or absolute M-E Spearman coefficients (11 cell lines) among CpG_1_, …, CpG_j_.
4. Rank-score_i_ (methylation based pre-ranked GSEA) = methylation log_2_FC_k_
5. Rank-score_i_ (expression based pre-ranked GSEA) = expression log_2_FC_i_
6. Rank-score_i_ (M-E Spearman coefficients based pre-ranked GSEA) = M-E Spearman coefficient_k_

Genes were ranked based on their rank-scores and submitted to 3 separate pre-ranked GSEA analysis. In the pre-ranked GSEA, classic scoring scheme and gene sets “Hallmark gene sets v7.4”, “Curated gene sets v7.4”, “Biological Process subsets of gene ontology v7.4” and “Molecular Function subsets of gene ontology v7.4”, “Oncogenic signature gene sets v7.4” were selected for the analysis [16,17]. Finally, leading-edge genes of significantly enriched gene sets (false discovery rate (FDR) ≤ 0.25, absolute normalized enrichment score (|NES|) ≥ 2) from all 3 pre-ranked GSEA lists were identified [18].

**Expression-based in-silico validation and potential clinical relevance of identified genes**

The expression changes between *ARID1A*mt vs *ARID1A*wt of identified genes (n=2004) were further verified based on expression-based in-silico validation. In the step of expression-based in-silico validation, another two OCCC expression profiles were used. The first expression profile (Expression 21Q2 Public, CCLE database, Supplementary Table 2) was downloaded from DepMap website [19]. The 12 OCCC cell lines included in the first expression profile were the same as the ones included in the gene dependency data. The second expression profile was derived from the unused data of 9 OCCC cell lines from whole batch effect corrected GEO data. Using these two OCCC expression profiles, two expression log_2_FCs (expression log_2_FC (CCLE, 12 cell lines) and expression log_2_FC (GEO, 9 cell lines)) were calculated with "limma" method [10], respectively. At last, expression log_2_FCs (GEO, 11 cell lines) between *ARID1A*mt vs *ARID1A*wt of identified genes (n=2004) were compared to expression log_2_FC (CCLE, 12 cell lines) and expression log_2_FC (GEO, 9 cell lines). For a certain gene from the 2004 identified genes, only when its expression log_2_FC (GEO, 11 cell lines), expression log_2_FC (CCLE, 12 cell lines) and expression log_2_FC (GEO, 9 cell lines) all indicated the same direction of the expression change between *ARID1A*mt vs *ARID1A*wt OCCC, this gene passed the expression-based in-silico validation.

Moreover, methylation of primary OCCC tumors was also taken into account to obtain genes with more potential clinical potentials. Genes that differentially methylated in primary OCCC were considered as of more potential clinical relevance.

**The selection criteria of DM gene candidates and the significantly enriched gene-sets**

After pre-ranked GSEA analysis and expression-based in-silico validation, the identified DM leading-edge genes with consistent *ARID1A*-related methylation alterations in primary tumor and cell lines were selected. In addition, the selected gene-sets should consist of at least 2 DM leading-edge genes.

**Visualization of candidate genes and the enriched gene sets.**

Genes that passed the expression-based in-silico validation and showed more potential clinical relevance were regarded as candidates. A two-dimensional hierarchical unsupervised clustered heatmap constructed by R package “complexheatmap” [20] was used to visualize these candidate genes and their enriched gene sets. The distance between every two components in columns (gene candidates) or rows (gene sets) was calculated based on Spearman coefficients. At the end “ward.D2” method was used to cluster columns (gene candidates) and rows (gene sets) of the heatmap according to the corresponding distance. The additional information of genes (gene methylation log_2_FC, gene dependency and gene expression log_2_FC) and pathways (enrichment FDR of pathways) were added as columns annotations and row annotations, respectively.

UCSC accustom tracks were used to visualize methylation of genes. First, the genome locations of all CpGs targeting the selected genes were obtained by aligning the source sequences of CpG probes to Gh37/h19 with UCSC BLAT web tool [21]. Next, based on the genome locations of the CpGs targeting the selected genes and their Methylation log_2_FC in *ARID1A*mt/ko primary OCCC and cell lines vs *ARID1A*wt primary OCCC and cell lines, BED files were made. These BED files were subsequently uploaded to UCSC genome browser [22] and four accustom tracks, namely “Methylation in primary tumor”, “Methylation in cell lines”, “Methylation in ES2*^ARID1A-/-^*” and “Methylation in OVCA429*^ARID1A-/-^*”, were constructed. More details are described at the UCSC instruction site [23].

Utilizing the UCSC accustom tracks, CpG level methylation consistency between primary OCCC and cell line samples were evaluated (Supplementary Table 10). Within a certain region of a selected gene, CpGs that showed consistent methylation change in primary tumor, cell lines and at least one of the *ARID1A*ko models were given weight of 2. CpGs that showed consistent methylation change in primary tumor and any of cell lines and the *ARID1A*ko models were given weight of 1. When the methylation change in cell lines or *ARID1A*ko models was opposite to the methylation change in primary tumor, the weights of the CpGs are 0. At last, the weights of all the CpGs inside the focused region were summed up and used to determine the consistency score of the whole focused region within the gene.

**Bisulfite sequencing PCR (BSP)**

To validate the Infinium MethylationEPIC BeadChip arrays, BSP was performed. Sodium bisulfite treatment of isolated genomic DNA (1 µg/sample) was performed according to the recommendations of the EZ DNA methylation kit (Zymo Research). Primers for 13 genes were designed using MethPrimer and Zymor Research. Regions of interest were amplified by primers including the array probe sequences and the size of the amplicon should be between 200-500 bps (Supplementary Table 11). PCR was performed as described previously (Lendvai Epigen). PCR products were gel purified using the QIAquick Gel Extraction kit (Qiagen) according to the manufacturer’s instructions. Each amplified DNA sample was sent in for sequencing according to manufacturer’s instruction (Eurofins) using the specific reverse primers.

BSP methylation average ratio was calculated based on the weighted average of all CpG sites located in the focused region. The weights of methylated CpG sites, hemi-methylated CpG sites and unmethylated CpG sites were 1, 0.5 and 0, respectively. The missed CpG sites were excluded from the calculation. Next, the average β-values of CpG probes of Infinium MethylationEPIC BeadChip arrays located in the focused region was calculated. The consistency between the average β-values Infinium MethylationEPIC BeadChip arrays and BSP methylation average ratio was interpreted using spearman correlation.

**Quantitative reverse transcription PCR (RT-qPCR)**

For global demethylation, cells at 40–50% confluency were treated with demethylating agent 5-aza-2′-deoxycytidine (DAC) at a final concentration of 1 μM for 72h. After 72h, cells were harvested and processed for RNA isolation. RNA was isolated using a RNeasy mini kit (Qiagen) following the kit handbook. The quantity and integrity of isolated RNA were analyzed using a Nanodrop and electrophoresis on agarose gel. cDNA synthesis was done by RNase H+ reverse transcriptase with 1 μg total RNA using an iScript cDNA synthesis kit (BioRad, Hercules, CA, USA) according to the manufacturer’s instructions. RT-qPCR was performed in an ABI PRISM 7500HT Sequence Detector (Applied Biosystems, Foster City, CA, USA) with the following assays on demand: TRIP6 (Hs00377979_m1), TMEM101 (Hs00260720_m1), BCOR (Hs00372378_m1) and GAPDH (Hs02758991_g1) (ThermoFisher Scientific). The following cycling conditions were applied for amplification: 5 min at 95°C, and 40 two-step cycles of 15 s at 95°C and 25 s at 60°C. The threshold cycles (Ct) were extracted, and relative gene expression (2^-ΔCt^) was analyzed after normalizing for GAPDH, a house-keeping gene. Spearman correlation between 2^-ΔCt^ and gene expression from OCCC expression profiles was next calculated to determine the association between RT-qPCR and OCCC expression profiles.

**Reference**

1. Aryee MJ, Jaffe AE, Corrada-Bravo H, Ladd-Acosta C, Feinberg AP, Hansen KD, et al. Minfi: a flexible and comprehensive Bioconductor package for the analysis of Infinium DNA methylation microarrays. Bioinformatics. 2014 May 15;30(10):1363–9.

2. A cross-package Bioconductor workflow for analysing methylation array data.

3. Tomar T, de Jong S, Alkema NG, Hoekman RL, Meersma GJ, Klip HG, et al. Genome-wide methylation profiling of ovarian cancer patient-derived xenografts treated with the demethylating agent decitabine identifies novel epigenetically regulated genes and pathways. Genome Med. 2016 Oct 20;8(1).

4. van Iterson M. Quality control, probe/sample filtering and normalization of Infinium HumanMethylation450 BeadChip data: “The Leiden Approach.” 2016 Sep 29;

5. Pidsley R, Zotenko E, Peters TJ, Lawrence MG, Risbridger GP, Molloy P, et al. Critical evaluation of the Illumina MethylationEPIC BeadChip microarray for whole-genome DNA methylation profiling. Genome Biology 2016 17:1. 2016 Oct 7;17(1):1–17.

6. GitHub - sirselim/illumina450k_filtering: A collection of resources to filter “bad” probes from the Illumina 450k and EPIC methylation arrays.

7. CRAN - Package pheatmap.

8. Du P, Zhang X, Huang CC, Jafari N, Kibbe WA, Hou L, et al. Comparison of Beta-value and M-value methods for quantifying methylation levels by microarray analysis. BMC Bioinformatics 2010 11:1. 2010 Nov 30;11(1):1–9.

9. Ritchie ME, Phipson B, Wu D, Hu Y, Law CW, Shi W, et al. limma powers differential expression analyses for RNA-sequencing and microarray studies. Nucleic Acids Res. 2015 Apr 20;43(7):e47–e47.

10. CRAN - Package VennDiagram.

11. Arechederra M, Daian F, Yim A, Bazai SK, Richelme S, Dono R, et al. Hypermethylation of gene body CpG islands predicts high dosage of functional oncogenes in liver cancer. Nature Communications 2018 9:1. 2018 Aug 8;9(1):1–16.

12. Home - GEO - NCBI.

13. AK N, CJ C, Z Y, H Z, PH G, JG R, et al. A link between mir-100 and FRAP1/mTOR in clear cell ovarian cancer. Mol Endocrinol. 2010 Feb;24(2):447–63.

14. Yamaguchi K, Mandai M, Oura T, Matsumura N, Hamanishi J, Baba T, et al. Identification of an ovarian clear cell carcinoma gene signature that reflects inherent disease biology and the carcinogenic processes. Oncogene. 2010 Mar;29(12):1741–52.

15. Subramanian A, Tamayo P, Mootha VK, Mukherjee S, Ebert BL, Gillette MA, et al. Gene set enrichment analysis: A knowledge-based approach for interpreting genome-wide expression profiles. Proceedings of the National Academy of Sciences. 2005 Oct 25;102(43):15545–50.

16. Mootha VK, Lindgren CM, Eriksson KF, Subramanian A, Sihag S, Lehar J, et al. PGC-1α-responsive genes involved in oxidative phosphorylation are coordinately downregulated in human diabetes. Nature Genetics 2003 34:3. 2003 Jun 15;34(3):267–73.

17. D’Haene E, Jacobs EZ, Volders PJ, De Meyer T, Menten B, Vergult S. Identification of long non-coding RNAs involved in neuronal development and intellectual disability. Scientific Reports 2016 6:1. 2016 Jun 20;6(1):1–12.

18. DepMap: The Cancer Dependency Map Project at Broad Institute.

19. Z G, R E, M S. Complex heatmaps reveal patterns and correlations in multidimensional genomic data. Bioinformatics. 2016 Sep 15;32(18):2847–9.

20. Kent WJ. BLAT--the BLAST-like alignment tool. Genome Res. 2002 Apr 1;12(4):656–64.

21. Kent WJ, Sugnet CW, Furey TS, Roskin KM, Pringle TH, Zahler AM, et al. The human genome browser at UCSC. Genome Res. 2002 Jun 1;12(6):996–1006.

22. Raney BJ, Dreszer TR, Barber GP, Clawson H, Fujita PA, Wang T, et al. Track data hubs enable visualization of user-defined genome-wide annotations on the UCSC Genome Browser. Bioinformatics. 2014 Apr 1;30(7):1003–5.
