# Supplementary material for: Genome-wide DNA methylation in relation to ARID1A deficiency in ovarian clear cell carcinoma
Source: J Transl Med. 2024 Jun 10;22:556. doi: 10.1186/s12967-024-05311-7 (PMC11163774; doi:10.1186/s12967-024-05311-7)
Supplement: Supplementary file 3 — Supplementary material 3: Figure 1: Approach to identify ARID1A related DM genes with potential clinical value in OCCC for treatment. Figure 2: Batch effect correction of 2 OCCC expression profiles from GEO database. Figure 3: Hierarchical clustering analysis of OCCC tumors and cell lines on methylation level. Figure 4: One to one comparison of all individual CpGs between ARID1A deficient OCCC vs ARID1Awt OCCC across all the chromosomes. Figure 5: DNA methylation and gene expression of TRIP6 in ARID1A deficient OCCCs vs ARID1Awt OCCCs. Figure 6: DNA methylation and gene expression of TMEM101 in ARID1A deficient OCCCs vs ARID1Awt OCCCs. Figure 7: DNA methylation and gene expression of BCOR in ARID1A deficient OCCCs vs ARID1Awt OCCCs. Figure 8: DNA methylation and gene expression of ZIK1 in ARID1A deficient OCCCs vs ARID1Awt OCCCs. Figure 9: DNA methylation and gene expression of PCDHA1 in ARID1A deficient OCCCs vs ARID1Awt OCCCs. [file 12967_2024_5311_MOESM3_ESM.pdf]

Genome-wide DNA methylation in relation to *ARID1A* deficiency in ovarian clear cell carcinoma

Shang Li[1], Gert Jan Meersma[1,2], Jolanta Kupryjanczyk[3], Steven de Jong[1#], G. Bea A. Wisman.[2#]

1 Department of Medical Oncology, Cancer Research Center Groningen, University Medical Center Groningen, University of Groningen, Hanzeplein 1, 9713 GZ, Groningen, the Netherlands

2. Department of Gynecologic Oncology, Cancer Research Center Groningen, University Medical Center Groningen, University of Groningen, Hanzeplein 1, 9713 GZ, Groningen, the Netherlands

3. Department of Pathology, Maria Sklodowska-Curie National Research Institute of Oncology, Roentgena 5, 02-781, Warsaw, Poland

# Correspondence: s.de.jong@umcg.nl / g.b.a.wisman@umcg.nl

Steven de Jong and G. Bea A. Wisman jointly supervised the work.

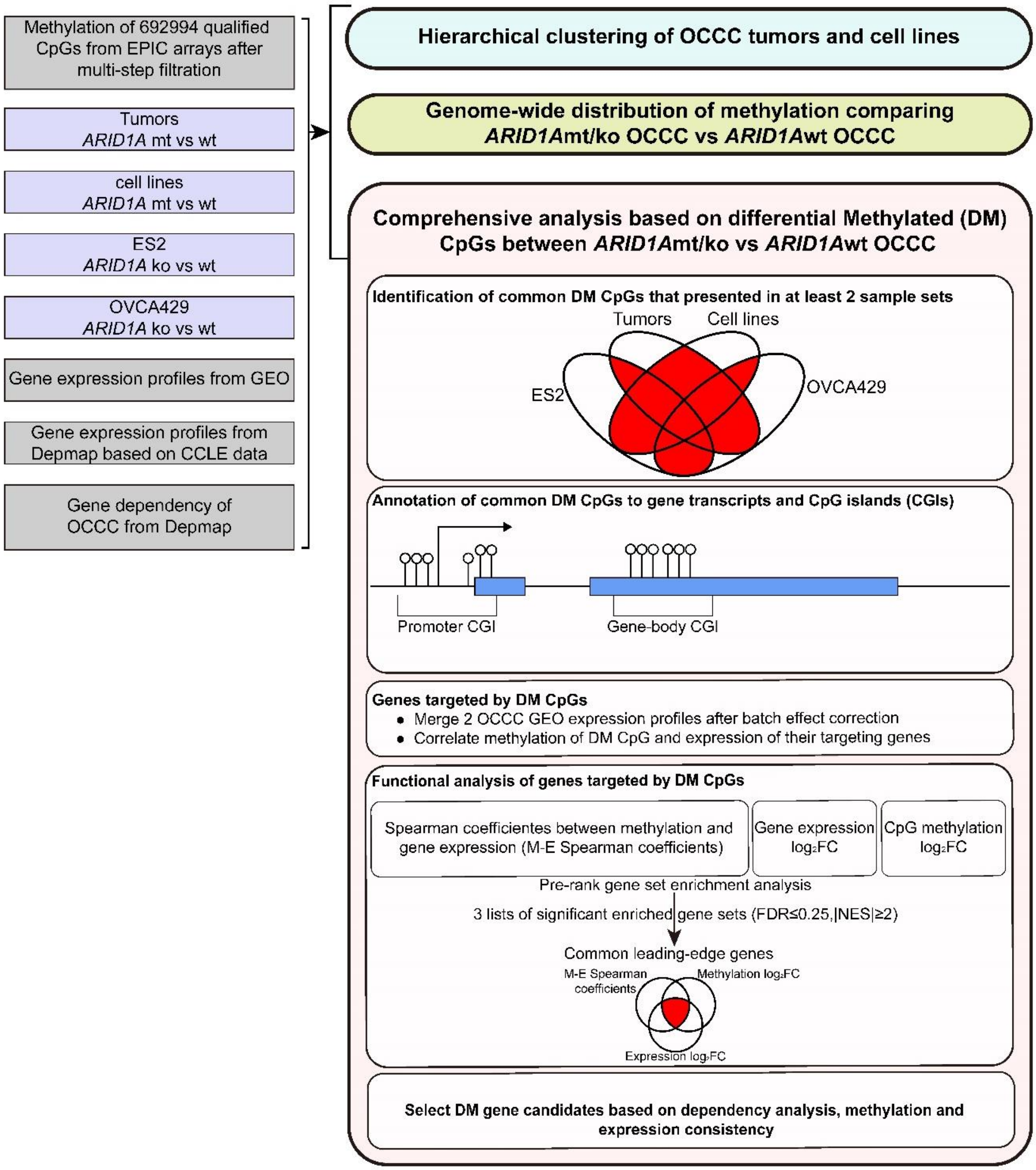

Supplementary Figure 1: Approach to identify *ARID1A* related DM genes with potential clinical value in OCCC for treatment.

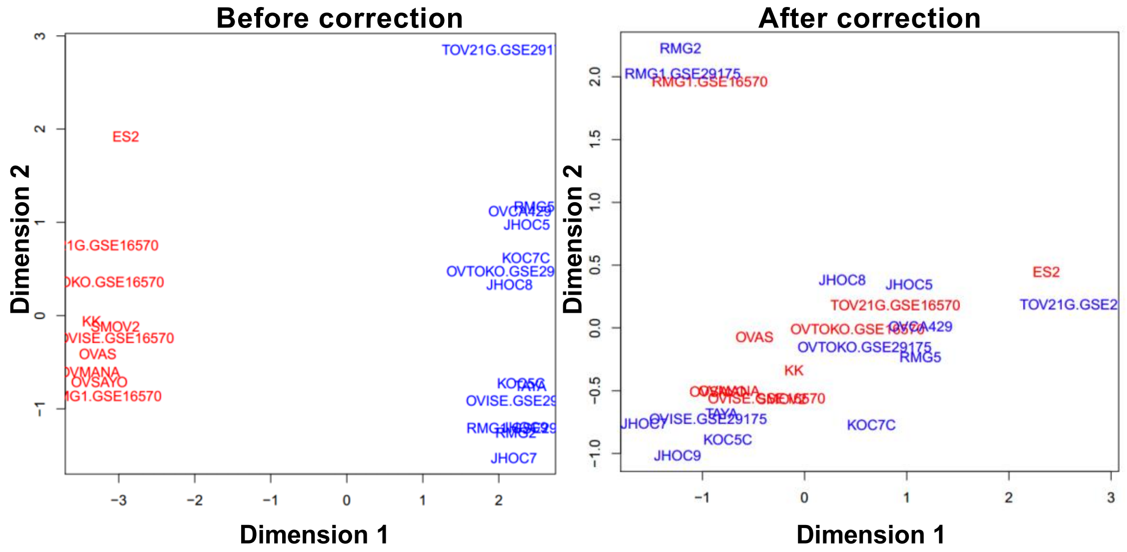

Supplementary Figure 2: Batch effect correction of 2 OCCC expression profiles from GEO database. Principal component analysis bi-plot of before (left) and after (right) batch effect correction. Red and blue colored text represent samples from GSE16570 and GSE29175, respectively.

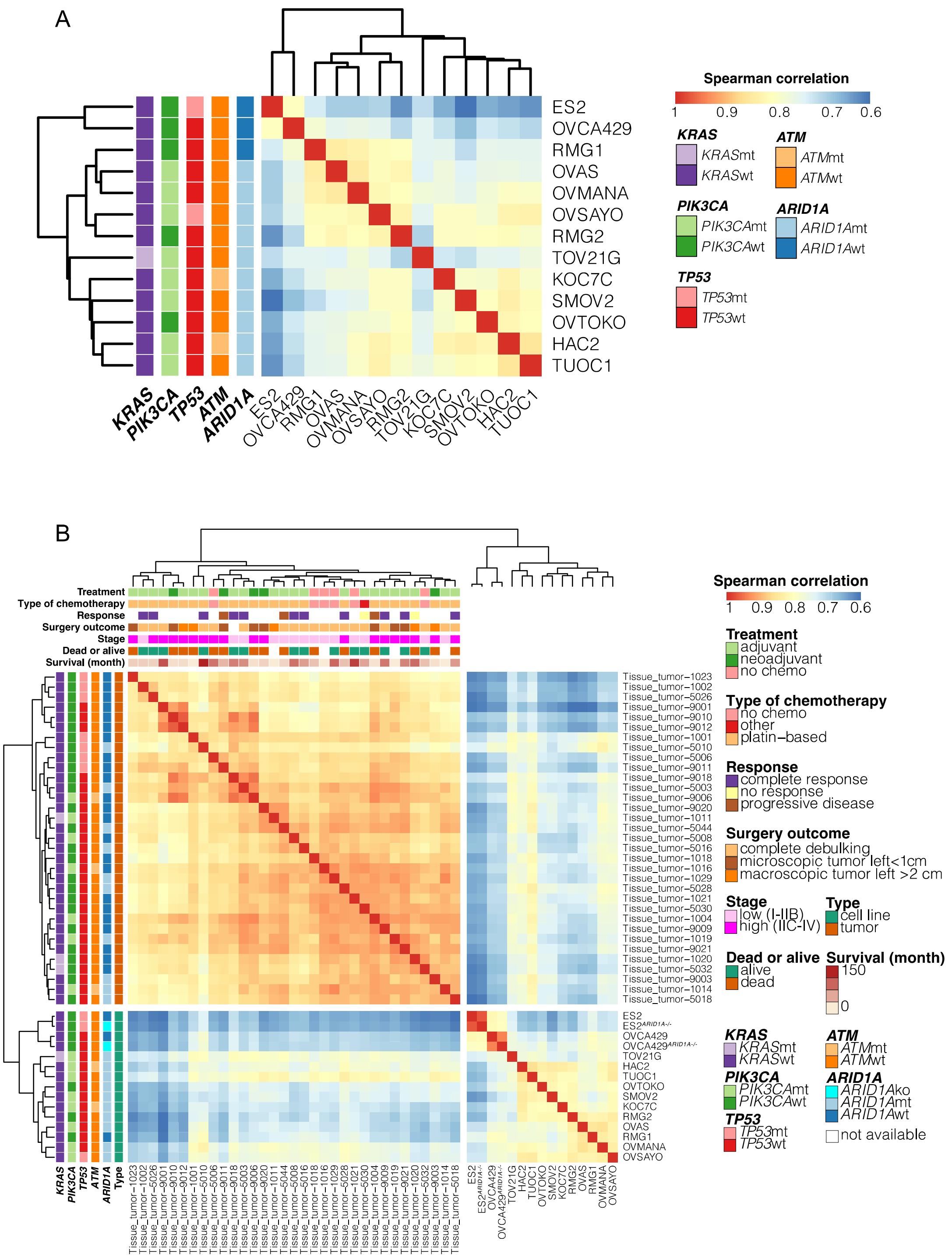

Supplementary Figure 3: Hierarchical clustering analysis of OCCC tumors and cell lines on methylation level. A) Unsupervised gain hierarchical clustering of OCCC cell lines based on β-values of 692,994 CpGs. The genetic mutations of OCCC cell lines are indicated. B) Unsupervised two-dimensional hierarchical clustering of OCCC tumors and cell lines based on β-values of 692,994 CpGs. The clinical data of OCCC tumors and genetic mutations of OCCC tumors and cell lines are indicated.

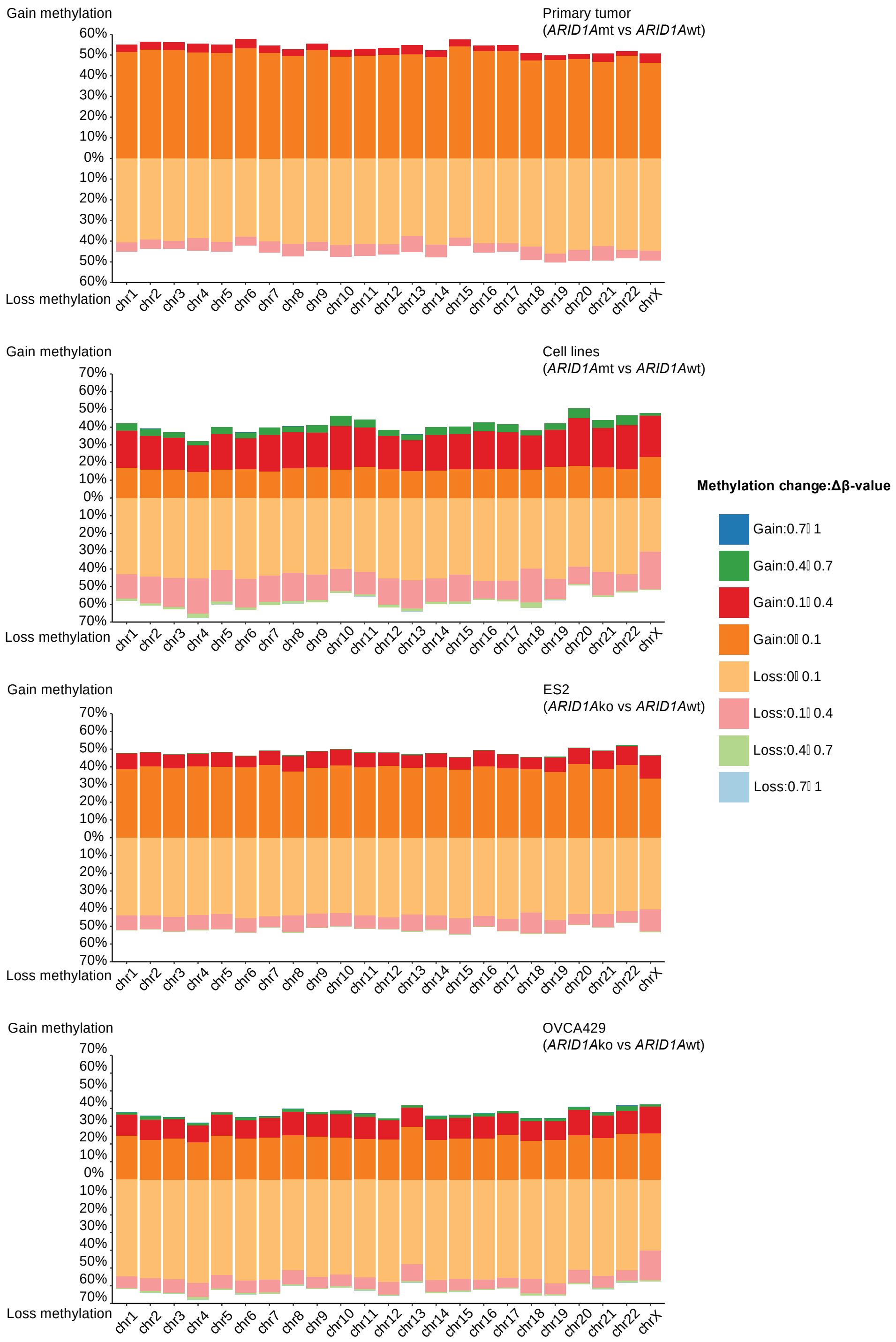

Supplementary Figure 4: One to one comparison of all individual CpGs between *ARID1A* deficient OCCC vs *ARID1A*wt OCCC across all the chromosomes. CpGs located in each chromosome are grouped based on the whether and how much their methylation β-value changed (Δβ-value) in *ARID1A* deficient OCCC comparing to *ARID1A*wt OCCC (8 groups: Gain:0.7-1, Gain:0.4-0.7, Gain:0.1-0.4, Gain:0-0.1, Loss:0-0.1, Loss:0.1-0.4, Loss:0.4-0.7, Loss:0.7-1). The size of components within each stacked bar indicate the percentage of these 8 groups within each chromosome.

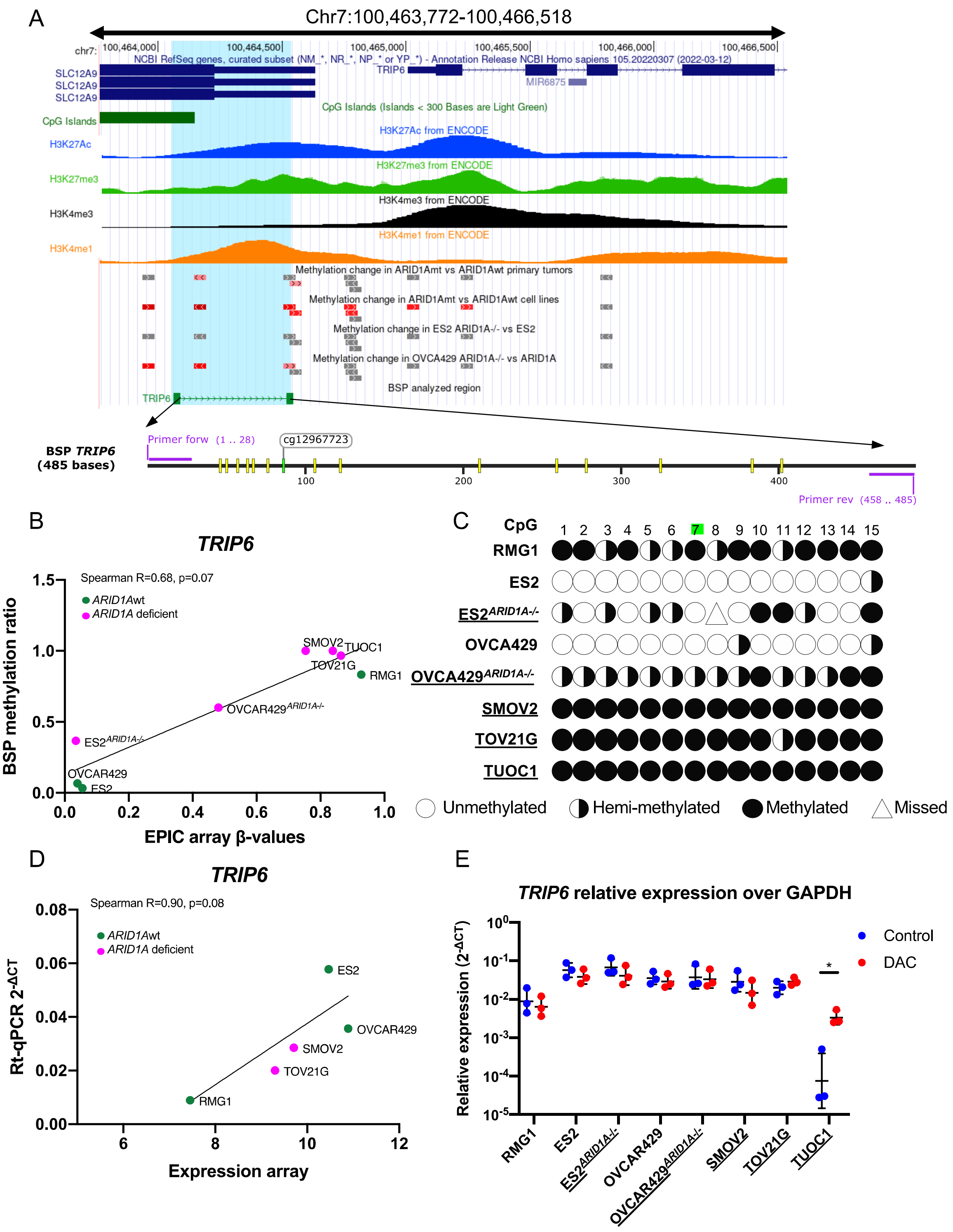

Supplementary Figure 5: DNA methylation and gene expression of *TRIP6* in *ARID1A* deficient OCCCs vs *ARID1A*wt OCCCs. A) DNA methylation of *TRIP6* promoter in OCCC cell lines. UCSC genome browser (GRCh37/hg19) representation of the genomic organization of *TRIP6*. The thick solid blocks indicate the coding regions, the thinner blocks indicate the 5' and 3'UTRs, blue lines indicate introns and arrows indicate the direction of gene transcription. The CGIs are represented as horizontal green bars. H3K27me3 (green), H3K27Ac (blue), H3K4me3

(black), H3K4me1 (orange) data from ENCODE project depict histone modification status as peaks. CpGs gaining methylation (red), losing methylation (blue), insignificant (gray) in *ARID1A* deficient vs *ARID1A*wt OCCC are represented as horizontal solid bars. BSP PCR product is indicated by solid boxes (primers) and green line (analyzed sequence). The BSP-analyzed region shaded in light blue is presented below with CpG located in the BSP-analyzed region depicted as yellow bars. The labeled and green CpGs are mutually analyzed by Infinium MethylationEPIC BeadChip arrays and BSP. B) *TRIP6* BSP methylation ratio vs average β-value from Infinium MethylationEPIC BeadChip array in *ARID1A*mt (pink) and *ARID1A*wt (green) OCCC cells. The black solid line represents the regression line. C) BSP result of *TRIP6* in OCCC cells. CpG sites located in the BSP-analyzed region are numbered and showed. CpG mutually analyzed by Infinium MethylationEPIC BeadChip arrays and BSP are specified with green color. Empty circles represent unmethylated CpGs, black circles represent methylated CpGs, half black circles represent hemi-methylated CpGs and empty triangles represent missed CpGs. *ARID1A* deficient cells are underlined. D) *TRIP6* relative gene expression based on RT-qPCR vs publicly available expression profiles of *ARID1A*mt (pink) and *ARID1A*wt (green) OCCC cells. The black solid line represents the regression line. E) RT-qPCR result of *TRIP6* in OCCC cells with (red) or without (blue) DAC treatment. *ARID1A* deficient cells are underlined. Statistical significance of Student T-test is notified as * $p<0.05$.

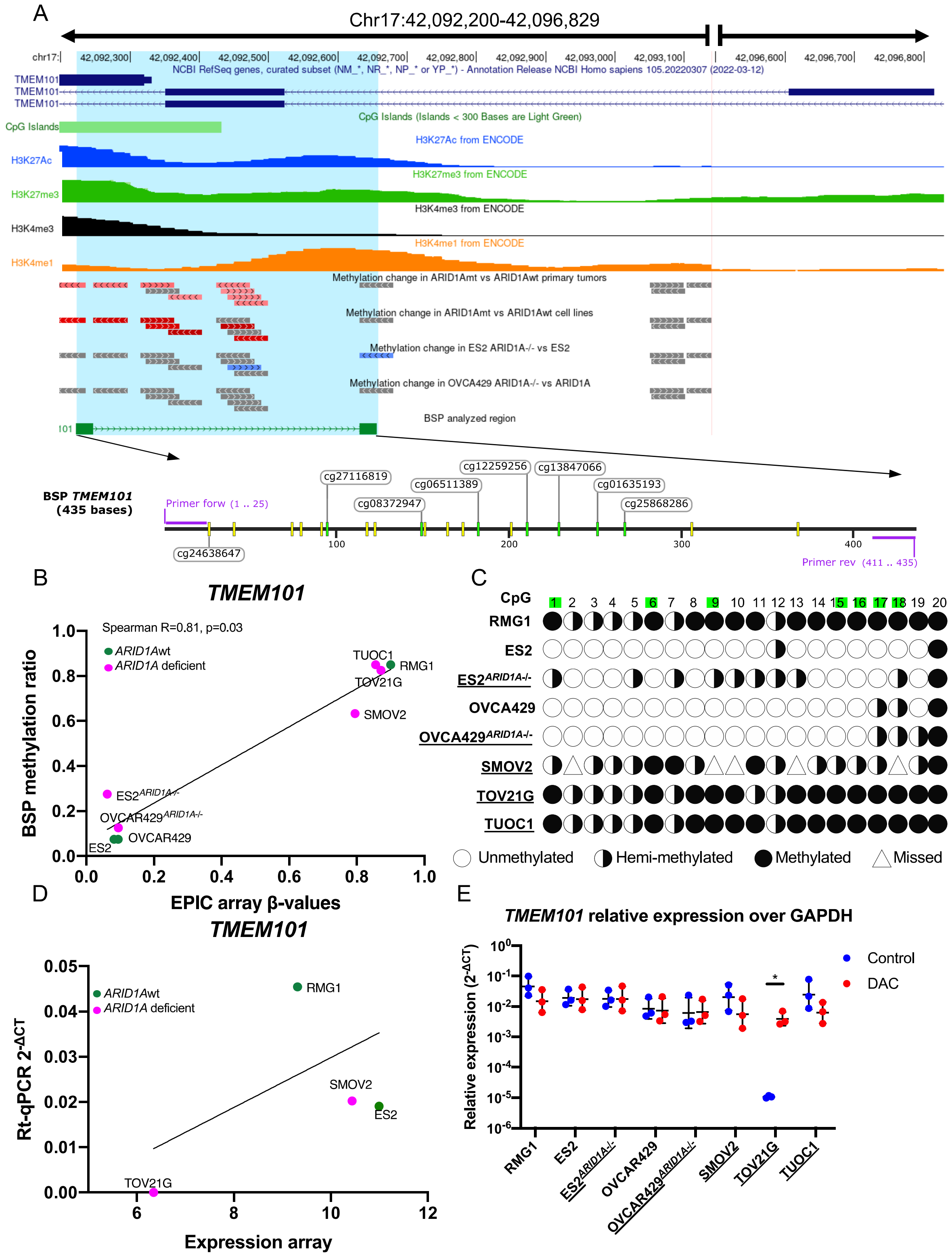

Supplementary Figure 6: DNA methylation and gene expression of *TMEM101* in *ARID1A* deficient OCCCs vs *ARID1A*wt OCCCs. A) DNA methylation of *TMEM101* promoter in OCCC cell lines. UCSC genome browser (GRCh37/hg19) representation of the genomic organization of *TMEM101*. The thick solid blocks indicate the coding regions, the thinner blocks indicate the 5'

and 3'UTRs, blue lines indicate introns and arrows indicate the direction of gene transcription. The CGIs are represented as horizontal green bars. H3K27me3 (green), H3K27Ac (blue), H3K4me3 (black), H3K4me1 (orange) data from ENCODE project depict histone modification status as peaks. CpGs gaining methylation (red), losing methylation (blue), insignificant (gray) in *ARID1A* deficient vs *ARID1A*wt OCCC are represented as horizontal solid bars. BSP PCR product is indicated by solid boxes (primers) and green line (analyzed sequence). The BSP-analyzed region shaded in light blue is presented below with CpG located in the BSP-analyzed region depicted as yellow bars. The labeled and green CpGs are mutually analyzed by Infinium MethylationEPIC BeadChip arrays and BSP. B) *TMEM101* BSP methylation ratio vs average β-value from Infinium MethylationEPIC BeadChip array in *ARID1A*mt (pink) and *ARID1A*wt (green) OCCC cells. The black solid line represents the regression line. C) BSP result of *TMEM101* in OCCC cells. CpG sites located in the BSP-analyzed region are numbered and showed. CpG mutually analyzed by Infinium MethylationEPIC BeadChip arrays and BSP are specified with green color. Empty circles represent unmethylated CpGs, black circles represent methylated CpGs, half black circles represent hemi-methylated CpGs and empty triangles represent missed CpGs. *ARID1A* deficient cells are underlined. D) *TMEM101* relative gene expression based on RT-qPCR vs publicly available expression profiles of *ARID1A*mt (pink) and *ARID1A*wt (green) OCCC cells. The black solid line represents the regression line. E) RT-qPCR result of *TMEM101* in OCCC cells with (red) or without (blue) DAC treatment. *ARID1A* deficient cells are underlined. Statistical significance of Student T-test is notified as * $p<0.05$.

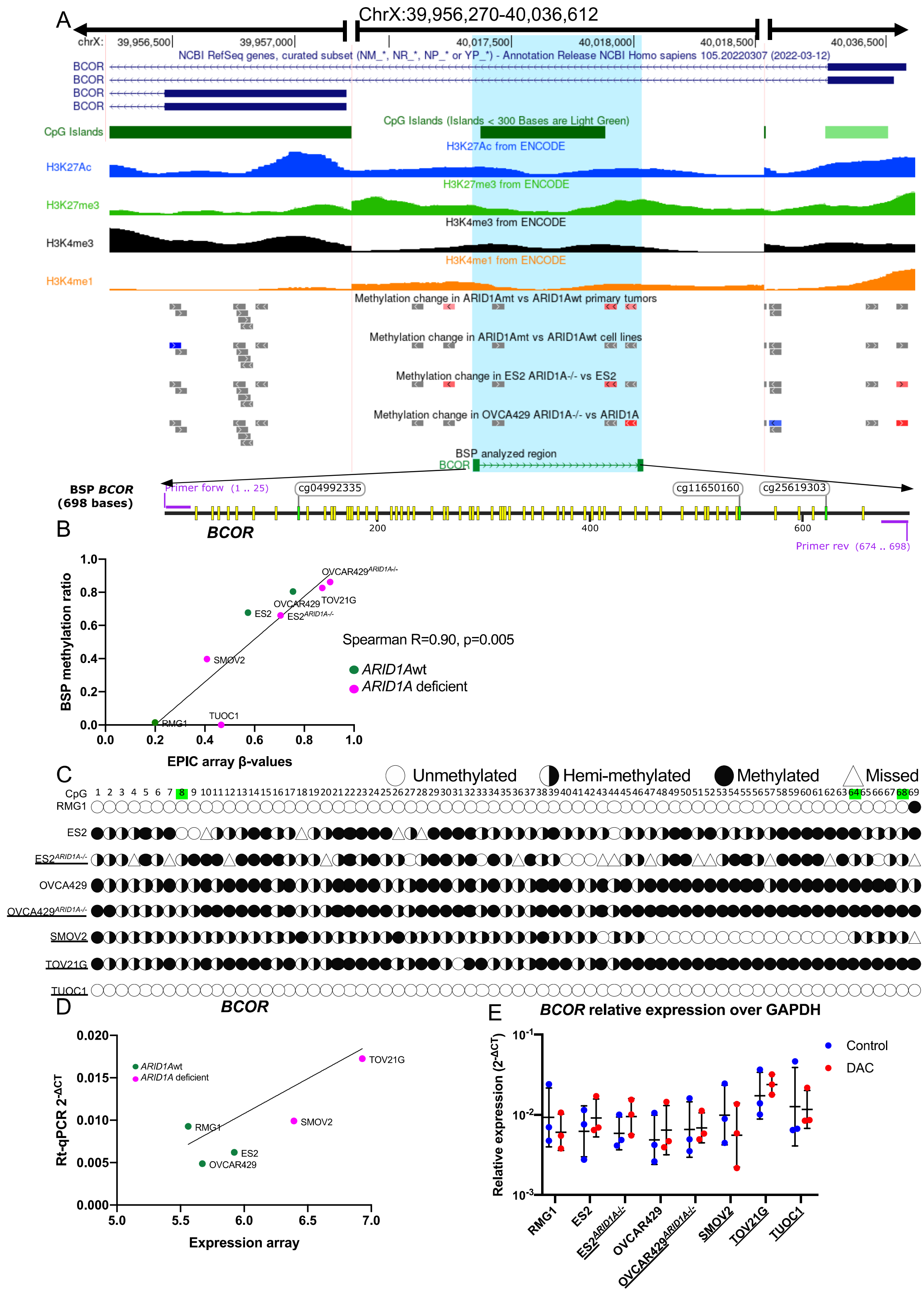

Supplementary Figure 7: DNA methylation and gene expression of *BCOR* in *ARID1A* deficient OCCCs vs *ARID1A*wt OCCCs. A) DNA methylation of *BCOR* promoter in OCCC cell

lines. UCSC genome browser (GRCh37/hg19) representation of the genomic organization of *BCOR*. The thick solid blocks indicate the coding regions, the thinner blocks indicate the 5' and 3'UTRs, blue lines indicate introns and arrows indicate the direction of gene transcription. The CGIs are represented as horizontal greenbars. H3K27me3 (green), H3K27Ac (blue), H3K4me3 (black), H3K4me1 (orange) data from ENCODE project depict histone modification status as peaks. CpGs gaining methylation (red), losing methylation (blue), insignificant (gray) in *ARID1A* deficient vs *ARID1A*wt OCCC are represented as horizontal solid bars. BSP PCR product is indicated by solid boxes (primers) and green line (analyzed sequence). The BSP-analyzed region shaded in light blue is presented below with CpG located in the BSP-analyzed region depicted as yellow bars. The labeled and green CpGs are mutually analyzed by Infinium MethylationEPIC BeadChip arrays and BSP. B) *BCOR* BSP methylation ratio vs average β-value from Infinium MethylationEPIC BeadChip array in *ARID1A*mt (pink) and *ARID1A*wt (green) OCCC cells. The black solid line represents the regression line. C) BSP result of *BCOR* in OCCC cells. CpG sites located in the BSP-analyzed region are numbered and showed. CpG mutually analyzed by Infinium MethylationEPIC BeadChip arrays and BSP are specified with green color. Empty circles represent unmethylated CpGs, black circles represent methylated CpGs, half black circles represent hemi-methylated CpGs and empty triangles represent missed CpGs. *ARID1A* deficient cells are underlined. D) *BCOR* relative gene expression based on RT-qPCR vs publicly available expression profiles of *ARID1A*mt (pink) and *ARID1A*wt (green) OCCC cells. The black solid line represents the regression line. E) RT-qPCR result of *BCOR* in OCCC cells with (red) or without (blue) DAC treatment. *ARID1A* deficient cells are underlined. Statistical significance of Student T-test is notified as * $p<0.05$.

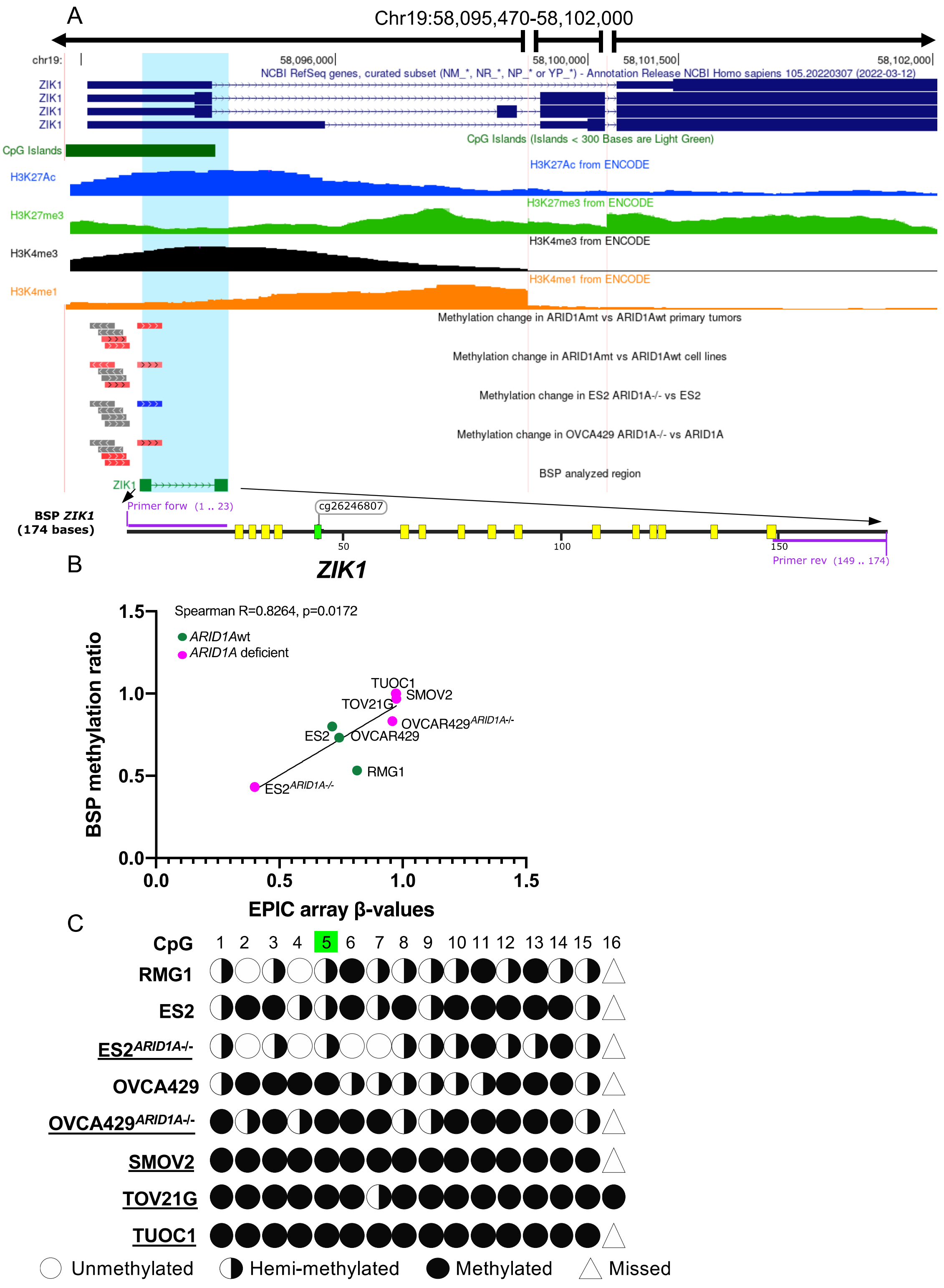

Supplementary Figure 8: DNA methylation and gene expression of *ZIK1* in *ARID1A* deficient OCCCs vs *ARID1A*wt OCCCs. A) DNA methylation of *ZIK1* promoter in *ARID1A* deficient OCCC. UCSC genome browser (GRCh37/hg19) representation of the genomic organization of *ZIK1*. The thick solid blocks indicate the coding regions, the thinner blocks indicate the 5' and

3'UTRs, blue lines indicate introns and arrows indicate the direction of gene transcription. The CGIs are represented as horizontal greenbars. H3K27me3 (green), H3K27Ac (blue), H3K4me3 (black), H3K4me1 (orange) data from ENCODE project depict histone modification status as peaks. CpGs gaining methylation (red), losing methylation (blue), insignificant (gray) in *ARID1A* deficient vs *ARID1A*wt OCCC are represented as horizontal solid bars. BSP PCR product is indicated by solid boxes (primers) and green line (analyzed sequence). The BSP-analyzed region shaded in light blue is presented below with CpG located in the BSP-analyzed region depicted as yellow bars. The labeled and green CpGs are mutually analyzed by Infinium MethylationEPIC BeadChip arrays and BSP. B) BSP methylation ratio vs average β-value of *ZIK1* from Infinium MethylationEPIC BeadChip array in *ARID1A*mt (pink) and *ARID1A*wt (green) OCCC cells. The black solid line represents the regression line. C) BSP results of *ZIK1* in OCCC cell lines. CpG sites located in the BSP-analyzed region are numbered and showed. CpG sites that are mutually analyzed by Infinium MethylationEPIC BeadChip arrays and BSP are specified with green color. Empty circles represent unmethylated CpGs, black circles represent methylated CpGs, half black circles represent hemi-methylated CpGs and empty triangles represent misses CpGs. Labels of *ARID1A* deficient cells are underlined.

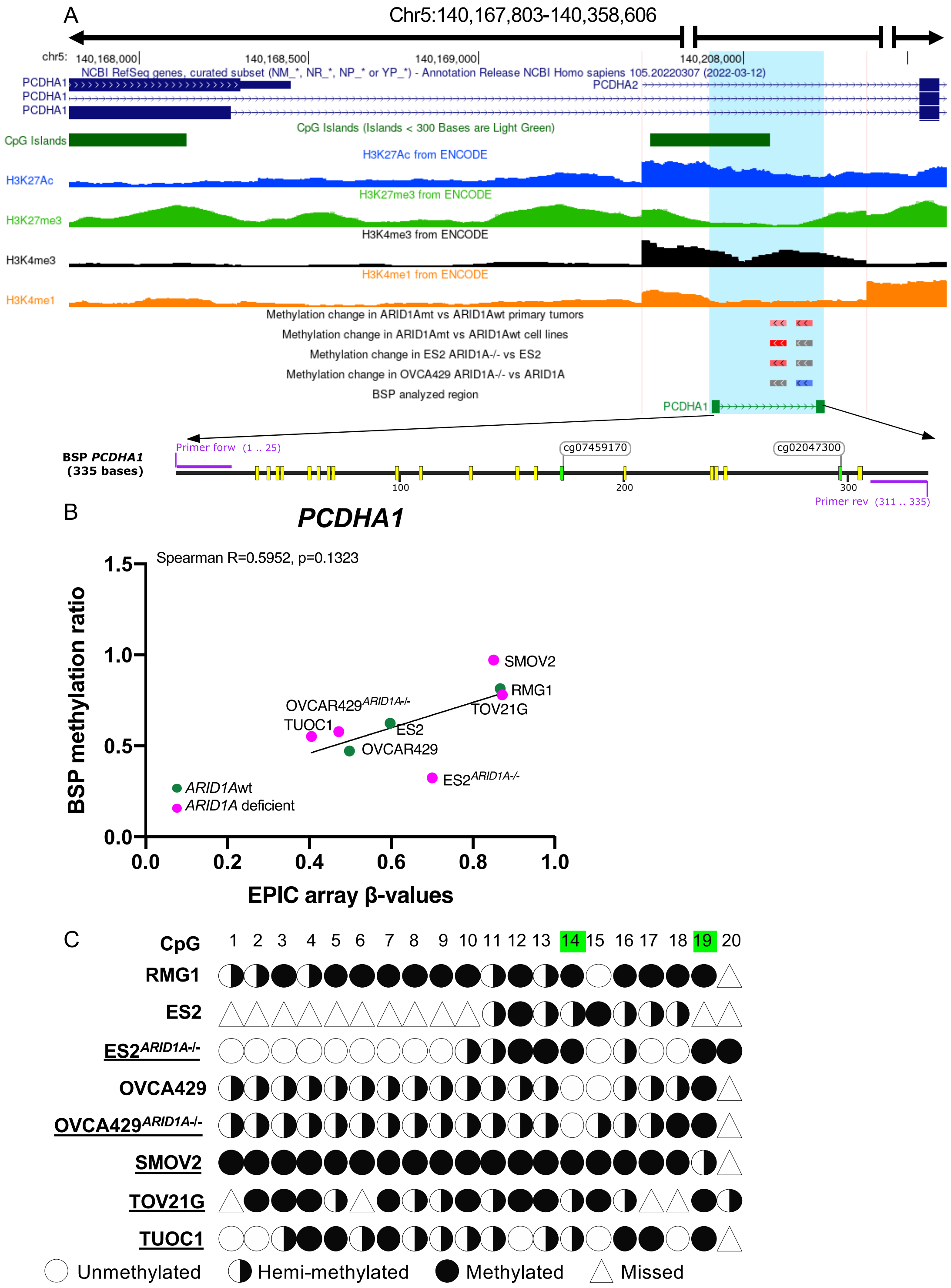

Supplementary Figure 9: DNA methylation and gene expression of *PCDHA1* in *ARID1A* deficient OCCCs vs *ARID1A*wt OCCCs. A) DNA methylation of *PCDHA1* promoter in *ARID1A* deficient OCCC. UCSC genome browser (GRCh37/hg19) representation of the genomic organization of *PCDHA1*. The thick solid blocks indicate the coding regions, the thinner blocks indicate the 5' and 3'UTRs, blue lines indicate introns and arrows indicate the direction of gene

transcription. The CGIs are represented as horizontal greenbars. H3K27me3 (green), H3K27Ac (blue), H3K4me3 (black), H3K4me1 (orange) data from ENCODE project depict histone modification status as peaks. CpGs gaining methylation (red), losing methylation (blue), insignificant (gray) in *ARID1A* deficient vs *ARID1A*wt OCCC are represented as horizontal solid bars. BSP PCR product is indicated by solid boxes (primers) and green line (analyzed sequence). The BSP-analyzed region shaded in light blue is presented below with CpG located in the BSP-analyzed region depicted as yellow bars. The labeled and green CpGs are mutually analyzed by Infinium MethylationEPIC BeadChip arrays and BSP. B) BSP methylation ratio vs average β-value of *PCDHA1* from Infinium MethylationEPIC BeadChip array in *ARID1A*mt (pink) and *ARID1A*wt (green) OCCC cells. The black solid line represents the regression line. C) BSP results of *PCDHA1* in OCCC cell lines. CpG sites located in the BSP-analyzed region are numbered and showed. CpG sites that are mutually analyzed by Infinium MethylationEPIC BeadChip arrays and BSP are specified with green color. Empty circles represent unmethylated CpGs, black circles represent methylated CpGs, half black circles represent hemi-methylated CpGs and empty triangles represent misses CpGs. Labels of *ARID1A* deficient cells are underlined.
